# Catalytic Upcycling of Polyolefins

**DOI:** 10.1021/acs.chemrev.3c00943

**Published:** 2024-08-16

**Authors:** Jiakai Sun, Jinhu Dong, Lijun Gao, Yu-Quan Zhao, Hyunjin Moon, Susannah L. Scott

**Affiliations:** 1 Department of Chemistry and Biochemistry, University of California, Santa Barbara, California 93106-9510, United States; 2 Department of Chemical Engineering, University of California, Santa Barbara, California 93106-5080, United States

## Abstract

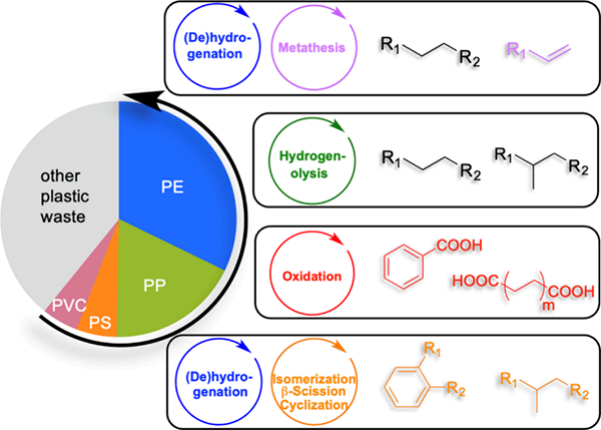

The large production volumes of commodity polyolefins (specifically, polyethylene, polypropylene, polystyrene, and poly(vinyl chloride)), in conjunction with their low unit values and multitude of short-term uses, have resulted in a significant and pressing waste management challenge. Only a small fraction of these polyolefins is currently mechanically recycled, with the rest being incinerated, accumulating in landfills, or leaking into the natural environment. Since polyolefins are energy-rich materials, there is considerable interest in recouping some of their chemical value while simultaneously motivating more responsible end-of-life management. An emerging strategy is catalytic depolymerization, in which a portion of the C–C bonds in the polyolefin backbone is broken with the assistance of a catalyst and, in some cases, additional small molecule reagents. When the products are small molecules or materials with higher value in their own right, or as chemical feedstocks, the process is called upcycling. This review summarizes recent progress for four major catalytic upcycling strategies: hydrogenolysis, (hydro)cracking, tandem processes involving metathesis, and selective oxidation. Key considerations include macromolecular reaction mechanisms relative to small molecule mechanisms, catalyst design for macromolecular transformations, and the effect of process conditions on product selectivity. Metrics for describing polyolefin upcycling are critically evaluated, and an outlook for future advances is described.

## 1 General Introduction

### 1.1 Catalytic Upcycling Strategies for Polyolefins

Plastics are unquestionably one of the most important technologies of the last century. They have transformed sectors as diverse as health care, food safety, textiles, electronics, transportation, machinery, and construction, and have made a vast array of consumer goods more broadly accessible to people in all sectors of society. Since the early days of mass production, the global production of plastics has soared, from less than 2 million metric tonnes (MMT) in the early 1950s to nearly 450 MMT today.^1,2^ On our current trajectory, it is possible that by 2050, the world will be producing 1100 MMT plastics each year.^3^ By 2050, the plastics industry is predicted to consume as much of 20 % of remaining oil production, while generating 15% of annual carbon emissions.^4^

The commodity polyolefins (POs) are polymers of simple alkenes or substituted alkenes, namely, ethylene (PE), propylene (PP), vinyl chloride (PVC) and styrene (PS). These four POs represent the majority of modern plastics. In 2021, PE accounted for approx. 27 % of plastic production, including low-density polyethylene (LDPE) and high-density polyethylene (HDPE), while PP constituted about 20 %, PVC about 13 %, and PS about 5 %.^5^ Thus POs represent two-thirds of all plastic production and a similar fraction of the plastic waste.^487^

This immense manufacturing scale, combined with the low cost of POs, has led to a proliferation of short-term uses and a corresponding surge in waste, with an estimated 353 MMT plastic waste generated in 2019 alone.^2^ Globally, approx. 49 % ends up in landfills. About 19 % is incinerated, which can achieve a modicum of energy recovery but which also releases CO_2_ and (potentially) toxic organic compounds into the environment. Conventional recycling (9 %) has not thus far proven to be a viable solution to address these issues.^7^ In addition, ca. 22 % of the waste is not responsibly managed, leading to leakage into the natural environment including the rivers and oceans. This leakage contributes to microplastics and nanoplastics in water, causing widespread public concerns about health impacts. (Note however that an early and highly publicized estimate of the amounts of these microscopic plastic particles that humans ingest has now been shown to be many orders of magnitude too high,^9^ providing an important cautionary tale^8,10^ about hasty and/or unreproducible science.^11^)

**Figure 1:**
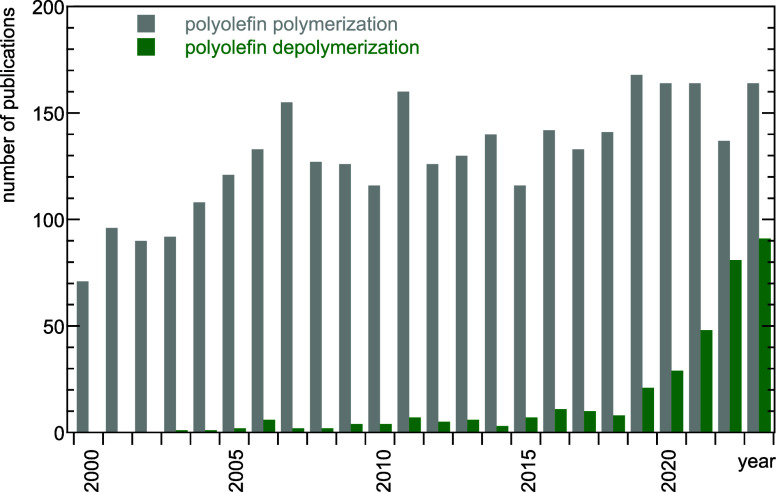
Comparison of the number of catalysis papers published from 2000 to 2023 on “alkene polymerization” (grey; search terms: catalytic polymerization AND polyethylene OR polypropylene OR polystyrene OR poly(vinyl chloride)) vs. “polyolefin depolymerization” (green; search terms: catalytic depolymerization AND polyethylene OR polypropylene OR polystyrene OR poly(vinyl chloride)). Data from Web of Science.

Clearly, the challenge has inspired researchers’ interest. Figure 1 compares the number of catalysis papers published from 2000 to 2023 on alkene polymerization to those published on polyolefin depolymerization. While the number of reports on alkene polymerization has increased slowly over the last two decades, work on polyolefin depolymerization has surged recently. In this rapidly developing field, advances will depend crucially on the development of robust protocols and benchmarks for evaluating catalysts. These topics will be discussed in sections 1.2 and 1.3.

Plastic waste can be recycled mechanically, chemically, or via hybrid chemomechanical processes.^12−14^ Mechanical recycling involves remanufacturing using waste to make new products, without disassembling or otherwise modifying the polymer chains. This approach is most effective for recycling material of known composition and high purity (i.e., industrial scrap), rather than post-consumer waste. Even then, it usually compromises the mechanical properties (e.g., strength, toughness) of the original polymer due to inadvertent chemical changes, as well as the presence of (often incompletely known) impurities. In contrast, chemical recycling requires at least partial depolymerization into monomers and/or oligomers, which can be repolymerized or repurposed after appropriate chemical purification.^15^ This approach is most readily applied to heteroatom-containing polymers like polyesters, such as polyethylene terephthalate (PET), with hydrolysable ester linkages.^16^ It is not generally applicable to polyolefins, whose backbones consist solely of strong and relatively inert C–C bonds.^7^

Uncatalyzed depolymerization of POs to their monomers can, in principle, be achieved at elevated temperatures. These reactions are thermodynamically spontaneous above the polymer ceiling temperature (*T*_c_).^17^ However, POs tend to have high *T*_c_ values which make their thermal depolymerization unselective. In short, they pyrolyze before reaching their ceiling temperatures, resulting in complex and low-value mixtures of gases, low-quality liquids, and coke.^18^ For example, PE has a *T*_c_ of 610 °C, and alkenes including ethylene formed by its partial or full depolymerization above this temperature tend to convert spontaneously to aromatics.^19,20^ Therefore, PE is rarely chemically recycled. Even if the side-reactions could be suppressed, for example, by reactive separation of ethylene as it forms, thermal PE depolymerization would still be highly energy intensive due to the need to reinvest the large heat of polymerization (108 kJ/mol).^21^ Similar considerations apply to the depolymerization of PP (*T*_c_ = 466 °C),^22^ which is generally unselective (although high selectivity to propylene has been reported using a plasma-based approach).^23^ PS (*T*_c_ = 397 °C) also undergoes unselective thermal depolymerization.^24^ PVC has the lowest ceiling temperature of the major polyolefins (*T*_c_ = 350 °C), but it tends to undergo dehydrochlorination in the temperature range 250–320 °C rather than depolymerization, so that vinyl chloride is not formed.^25^ These considerations have been discussed in recent reviews.^17,26^

**Scheme 1:**
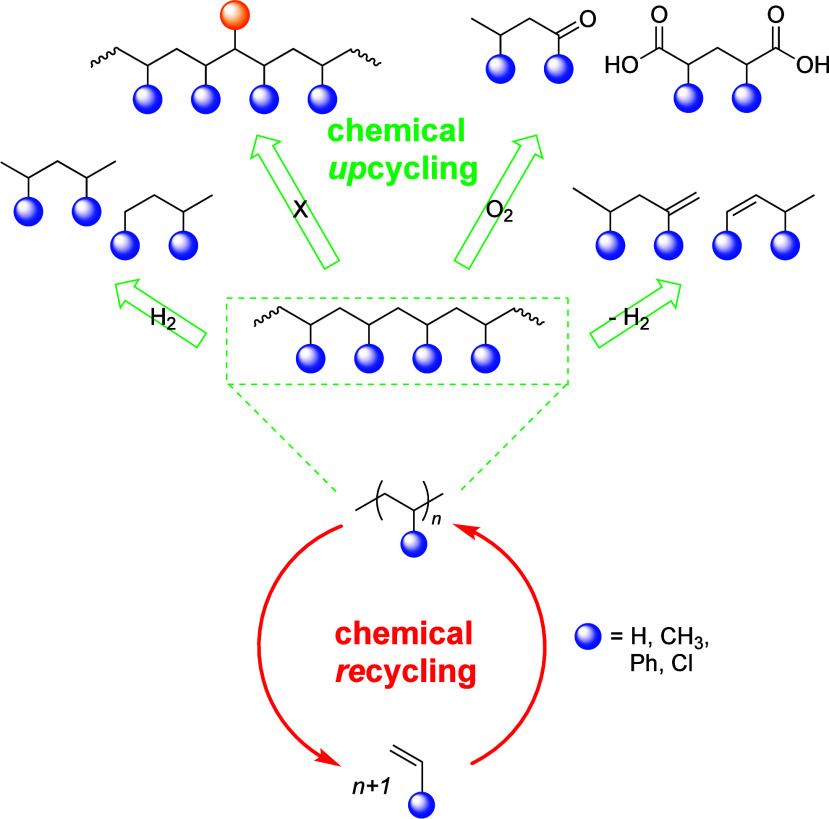
Comparison of Chemical Recycling and Upcycling of Polyolefins

Upcycling is a possible alternative for managing polyolefin waste.^15,27^ In contrast to recycling, upcycling is defined as conversion to a product with a higher value than the virgin material. For polymers, it can also include conversion to a product with higher value than the constituent monomer(s). The aim of PO upcycling is to selectively cleave specific C–C bonds, converting them into building blocks that can be used as feedstocks for chemical manufacturing. It requires a suitable catalyst, and often involves the use of co-reactants. Scheme 1 illustrates the key differences between chemical recycling and upcycling. Chemical *re*cycling necessitates breaking a large fraction of the C–C bonds in the PO backbone, while chemical *up*cycling targets a smaller fraction of these bonds. This selective cleavage reduces the energy requirement and tends to make the thermodynamics of upcycling reactions more more favorable.

The major PO upcycling strategies are summarized and compared in Table 1. In polyolefin hydrogenolysis, direct, metal-mediated cleavage of internal C–C bonds occurs in the presence of H_2_, capping the new chain ends with H. When hydrogenolysis breaks a terminal C–C bond, one product is methane, whose low value does not qualify it as upcycling. Catalytic cracking involves the formation of carbocations (carbenium and/or carbonium) that undergo spontaneous C–C bond scission. Although hydrocracking is performed in the presence of H_2_, alkene intermediates are still formed. They react with acid sites to form carbenium ions. C–X (X = H, Cl) bond cleavage also generates reactive sites. For example, polymer dehydrogenation to give a C=C site allows for chain cleavage via alkene metathesis, or by protonation to give a carbenium ion that is susceptible to chain cleavage by β-scission. Direct carbenium ion formation in saturated polyolefins (i.e., without first forming a C=C bond) can be achieved by heterolytic C–H bond cleavage, as occurs in hydride abstraction by a Lewis acid. Carbocation formation can also result from the protonation of a saturated hydrocarbon chain, generating a five-coordinate carbonium ion whose protolytic cracking leads to a carbenium ion.

**Table 1:** Comparison of Major Polyolefin Upcycling Processes

reaction	coreagents	catalyst types	C–C bond cleavage mechanism(s)	product types	potential applications
hydrogenolysis	H_2_	metal; metal hydride	homolytic bond cleavage with M–C bond formation; β-alkyl elimination	*n-*alkanes; *iso*-alkanes	lubricants; waxes; solvents
catalytic cracking	none	acid	β-scission of carbenium ion; protolytic cracking of carbonium ion	alkenes; *iso*-alkanes; cycloalkanes	new polyolefins; solvents
hydrocracking	H_2_	bifunctional metal + acid	β-scission of carbenium ion	*iso*-alkanes	lubricants; waxes; solvents
internal hydrogen redistribution	none	bifunctional metal + acid	β-scission of carbenium ion	aromatics; alkanes	anionic surfactants; lubricants
tandem cracking/alkylation	*iso*-alkane	acid	β-scission of carbenium ion	*iso*-alkanes	lubricants; waxes; solvents
alkane metathesis	*n*-alkane	metal alkylidene	alkenolysis	*n*-alkanes	lubricants
tandem isomerization/metathesis	ethylene	metal alkylidene	ethenolysis	propylene	polypropylene
partial oxidation	O_2_; H_2_O_2_	transition metal ions	β-scission of alkoxy radical	oxygenates	polyesters; non-ionic surfactants

Alkyl radicals are formed by homolytic C–H bond cleavage, when H atoms are abstracted by reactive oxygen species. Trapping of alkyl radicals by O_2_ leads eventually to alkoxy radicals that undergo β-scission. In the presence of excess oxidant, the polymer can be converted to small molecule oxygenates, or combusted to CO_x_. Obviously, the latter is not considered an upcycling reaction. Installing other functional groups without breaking the C–C bonds of the polymer backbone, a process already practiced with virgin polymers where it is called post-polymerization modification, can be classified as upcycling when it converts a post-consumer plastic into a new polymer with higher value.

Several reviews summarizing aspects of recent progress in the upcycling of POs focus on process strategy^28−35^ or product targets.^36−41^ This review attempts to present a comprehensive and current overview of *catalytic* upcycling of POs, with emphasis on catalyst design and catalytic mechanisms (these are mutually instructive). It is organized into four sections, each dedicated to a different major category of upcycling: hydrogenolysis, (hydro)cracking, tandem reactions, and selective oxidation. We concentrate on reactions that achieve C–C bond cleavage, in alignment with the primary focus of the review. Consequently, there is only limited discussion of post-polymerization modification strategies, in the section on selective polyolefin oxidation.

### 1.2 Useful Definitions in Polyolefin Upcycling

Catalytic transformations of POs usually involve a complex series of reactions. For example, C–C bond cleavage can occur on the main polymer chain or a side-chain, as well as on any of the many hydrocarbon fragments generated during the reaction. Many catalysis terms used in small molecule reactions are still useful, but can benefit from being precisely defined when they are used to describe polymer-based reactions. In addition, new terms are needed (see section 1.2.2 below) to more clearly describe the extent of reaction as well as characteristics of product classes when polymers are deconstructed. In the reports of catalytic PO reactions described later in this review, there are many instances of incomplete or inadequate polymer specification and/or product analysis. While we can only describe information provided in published reports, we offer the following guidance for future reports.

#### 1.2.1 Relevant Terms

##### 1.2.1.1 Polyolefin Conversion

The dimensionless *conversion* (*X*) is the fractional extent to which a reactant has been transformed into products. In small molecule reactions, a molar basis is usually implied. However, molar conversion is difficult to specify for reactions involving macromolecules. In polymer upcycling, conversion is therefore commonly (but not universally) defined instead as the ratio of the mass (*m*) of polymer reacted to the initial charge of polymer, eq 1. Authors should affirm the mass basis for this calculation in their reports.
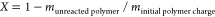
1

Nevertheless, the operational definition in eq 1 is still unsatisfactory. It is virtually impossible to determine whether a polymer chain present at the end of an experiment is truly unchanged, or arises from the cleavage of an even longer chain, since the molecular weights of reactants and products overlap for at least some time during the depolymerization process. Furthermore, each polymer chain undergoes many chain cleavage events to become a small molecule product. It is almost never specified (or even possible to define) how many such events—one, ten, one hundred...—constitute the threshold for considering a chain “converted”. Consequently, PO conversion is commonly calculated as the ratio between the mass of insoluble hydrocarbons and the initial PO charge, eq 2.
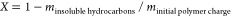
2

The general assumption (usually unstated) is that insoluble hydrocarbons (or the total mass of insoluble solids less the known mass of solid catalyst) represent unreacted PO exclusively. Clearly, this will often not be true. Insoluble hydrocarbons also include partially depolymerized material (slightly lower molecular weight), cross-linked material (higher molecular weight), and coke/coke precursors. Unreacted polymer should have the same molecular weight and molecular weight distribution (requiring specification of any two of *M*_n_, *M*_w_, and *Đ*) as the original polymer, and, where relevant (e.g., PP and PS), the same stereoregularity. More complete characterization of the hydrocarbon solids is generally desirable, although the low solubility of POs in general, and challenges in accessing high temperature gel permeation chromatography (GPC) for PO molecular weight analysis, complicate this goal. TGA has been used as a crude measure of the amount of unreacted polymer, via the weight loss that occurs at temperatures characteristic of the decomposition of the virgin polymer.^42−44^

##### 1.2.1.2 Yields of Upcycling Products

The *yield* (*Y*) refers to the amount of product obtained in a chemical reaction, often expressed as the fraction of the theoretical maximum amount that could be obtained. Yields may be assessed *in situ* (e.g., GC or NMR yields) or determined after purification (isolated yield), and are reported on either a molar or mass basis. Molar yields (in mol%) are common in molecular chemistry, where they conveniently reflect the reaction stoichiometry. However, chemicals are priced on a mass basis, so mass yields (in wt%) are more directly relevant to the economic viability of a process. It is essential that authors specify clearly which type of yield they are reporting.

For reactions involving macromolecules, where there is typically a large number of similar products, the mass yield is usually straightforward to calculate. The yield of any one type of product or, more often, group of products, is expressed in terms of its mass (*m*) relative to the initial polymer charge, eq 3.
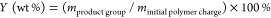
3

Even when products are complex and incompletely characterized mixtures with significant molecular weight dispersity, this operational definition is still easy to implement. Molar yields are less common. When available, they are usually calculated relative to the number of moles of monomer present in the initial charge of polymer. However, the hydrocarbon products are not always related in a simple way to the original monomer structure of the PO. Furthermore, Eq 3 is less useful for some types of upcycling reactions, including alkane metathesis and partial oxidation, where the total product yield may exceed 100 wt% by a significant amount because of the mass of co-reactants (e.g., alkanes or O_2_) incorporated into the products.

For alkane metathesis and partial oxidation, the carbon mass yield is a more useful metric because it reflects the efficiency of carbon incorporation from the polymer into the products. The carbon mass yield of one type of products (or group of products) is expressed in the terms of their polymer-derived carbon mass (*m*) relative to the carbon mass of the initial polymer charge, eq 4.
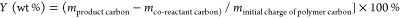
4Obviously, when some carbon in the products is derived from a co-reactant instead of the polymer, that carbon should not be counted in the carbon mass yield (although it is challenging to distinguish carbons based on their origin). We note that eq 4 is not useful in hydrogenolysis and hydrocracking, because the co-reactant (H_2_) does not introduce additional carbon, and the wt% carbon present in the products is very similar to that in the polymer.

Given these complications, yields in PO reactions are often represented operationally by phase yields: i.e., gases, liquids, solids. The definitions of each phase can overlap, depending on the recovery method used for each product phase type. For example, C_4_–C_6_ hydrocarbons may appear in the gas or liquid phases, or be distributed between them, depending on the conditions (e.g., temperature, pressure). The distinction between liquid and solid is temperature-dependent for heavier hydrocarbons. Definitions of the liquid phase that are based on solubility depend on the choice of extracting solvent (e.g., CH_2_Cl_2_) and its temperature, as well as the nature of the hydrocarbon products (e.g., oxygenates may be more soluble than hydrocarbons without heteroatoms). Sometimes, a portion of the unreacted polymer is soluble, resulting in an apparent non-zero initial conversion. In all cases, the experimental methods should describe the product recovery and phase definitions precisely.^44^

Regardless of the method chosen for reporting yields, it is important to assess and report the *mass balance*, i.e., the sum of the masses for all products compared to the initial masses of reactants (polymer and, where relevant, non-polymer co-reagents). In some reports, the lack of recovery of one of the product phases (typically, gases or solids) has been erroneously reported as a mass balance of exactly 100 %, assuming that the unrecovered phase represents all of the missing mass.^45−47^ At a minimum, such assumptions must be stated explicitly. The true mass balance reveals whether major species are missing in the product analysis. A common problem that leads to a poor mass balance is the loss of volatile liquids (principally, C_5_–C_7_ hydrocarbons), which are not fully vaporized when the gas phase is analyzed, but evaporate when the reactor is depressurized prior to opening.^44^

In order to reduce the loss of volatile liquids during product collection, gas and vapor products can be analyzed by expansion into an evacuated Schlenk tube or gas bag, to ensure quantitative gas collection and sampling. If the reactor pressure is higher than ambient, volatile liquids can be trapped by passing the headspace gases through a liquid absorbent, such as CS_2_. The quantity of products recovered in the absorbent can be measured by GC, using an internal standard. The reactor can also be cooled prior to opening, to ensure that volatile liquids remain condensed. Some volatiles may condense in the lines and valves of the reactor; failing to recover them will result in a poor mass balance. These parts should be rinsed thoroughly with a solvent (such as CHCl_3_ or CS_2_) to recover trapped hydrocarbons. Use of a glass liner inside the reactor can aid in the recovery of non-volatile solids, and has the added benefit of preventing contamination of the reacting polymer by metals or other contaminants from the reactor.

##### 1.2.1.3 Selectivities to Upcycling Products

In molecular reactions, the *selectivity* (*S*) refers to the molar ratio of products formed from a given reactant. Here too, defining selectivity is not straightforward for macromolecular reactions, since product mixtures are often complex, consisting of multiple product types with overlapping molecular weight distributions. A convenient operational definition for selectivity is the mass ratio relating one product type (i.e., group of products) to all products, eq 5. It is essential to specify the mass basis and indicate what the total mass refers to. For example, CH_4_ selectivity has been reported as either the mass of methane relative to the mass of all products, or the mass of methane relative to all gas products.
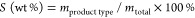
5Clearly, the selectivity is less meaningful when the mass balance is poor (i.e., when a significant fraction of the polymer mass is not taken accounted for). This situation is unfortunately rather common in PO reactions. Just as in molecular transformations, it is essential to compare selectivities for different catalysts and/or different reaction conditions at similar levels of conversion. Comparisons at constant reaction times are potentially misleading, since selectivity can vary greatly as a function of conversion.

Selectivity for a particular type of product, such as the selectivity for aromatic compounds among all hydrocarbon products, or the selectivity for hydrocarbons within a certain molecular weight range, such as gasoline-range compounds (C_5_–C_12_), is frequently reported. Another commonly reported selectivity is based on phase yields (gas, liquid, solid products). As described in section 1.2.1.2, this requires a clear description of the recovery method for each phase, since some products can appear in different phases, or in more than one phase, depending on the recovery method.

#### 1.2.2 Adapting Metrics to Describe Polyolefin Upcycling

##### 1.2.2.1 Number of C–C Bond Scission Events

**Figure 2:**
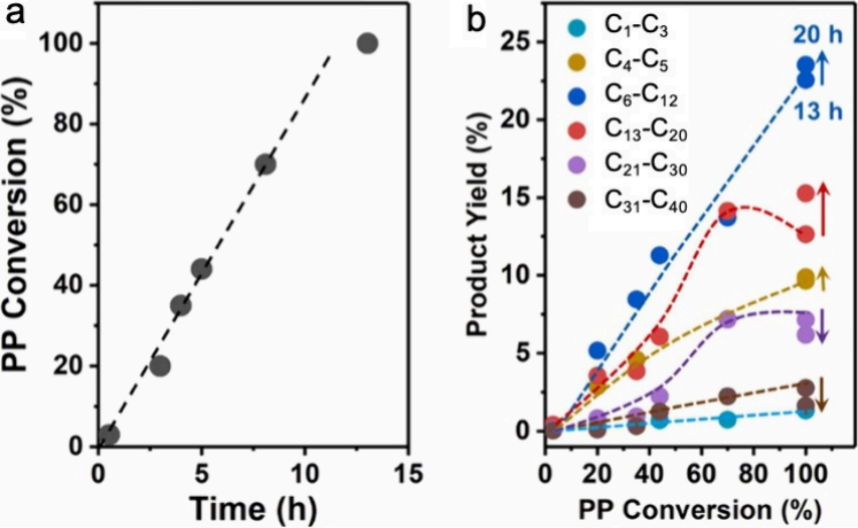
Time-dependence of PP hydrocracking: (a) increase in PP conversion with time; and (b) variations in the yields of various hydrocarbon product groups with conversion. Dual data points at 100 % PP conversion were measured at two reaction times (13 and 20 h), where the colored arrows represent increasing reaction time. Reproduced with permission from ref (48). Copyright 2023, Elsevier.

In small molecule reactions, the product yield is often related directly to the extent of reaction. The relationship is more complex for partial depolymerization of macromolecules. For example, in the random depolymerization of POs, the mass yield of intermediate-length hydrocarbon chains (for example, C_13_–C_20_, as shown in red in Figure 2) increases initially, then decreases as the reaction proceeds. This non-monotonic behavior occurs when POs break first into relatively long-chain hydrocarbons, which are then further converted into shorter chains. Thus, the yield of hydrocarbons in any particular molecular weight range is non-monotonic with respect to the extent of reaction, and its use in activity comparisons can be misleading.

Simply combining mass yields for different product types does not resolve the problem, if one still assumes that the total mass yield (equal to the PO conversion only when the mass balance closes) increases until the reaction is complete. When the total product yield (or PO conversion) reaches 100 %, depolymerization usually continues, as intermediate products undergo further depolymerization. After this point, the total product yield (or PO conversion) remains constant at 100 %, and is no longer meaningful for describing the extent of reaction.

**Scheme 2:**
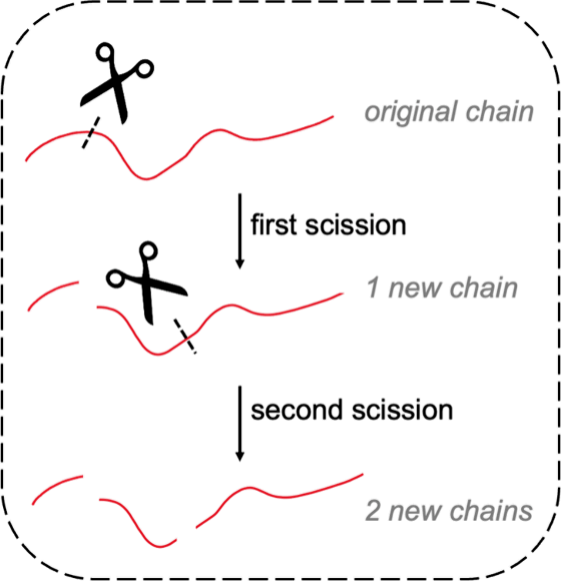
Each C–C Bond Scission Event Generates One New Hydrocarbon Chain, Making the Number of Events Equal to the Number of New Chains

An alternative, and potentially more precise, metric for assessing the extent of depolymerization of polyolefins is the *number of C–C bond scission events*.^49−51^ Each such event generates one new hydrocarbon chain (Scheme 2), and the total number of such events (*n*_C–C scission_) is given by eq 6:
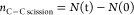
6where *N*(0) and *N*(t) represent the total number of polymer chains (including chain fragments) present in a batch reactor at time 0 and time *t*, respectively. This metric is also valid for other types of polymers, including condensation polymers such as PET.

**Figure 3:**
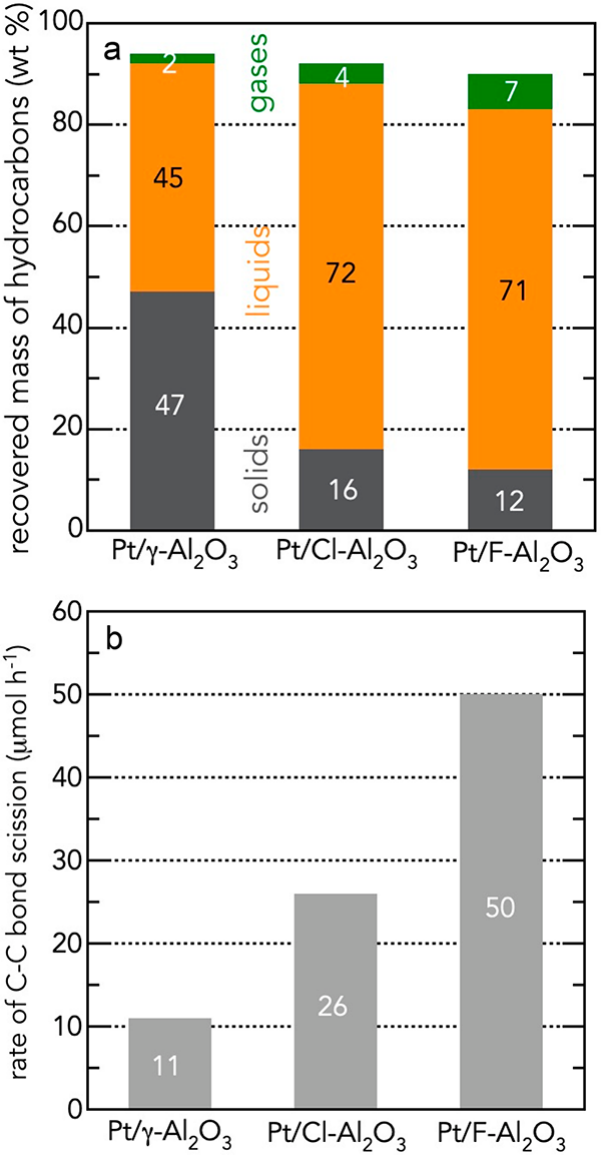
Activity comparison of three Pt-based catalysts for PE depolymerization: (a) by mass fractions of hydrocarbons recovered from a batch reactor: green, gases; orange, liquids soluble in hot CH_2_Cl_2_; black, organic residues insoluble in hot CH_2_Cl_2_; and (b) by average rate of C–C bond scission (defined as *n*_C–Cscission_/*t*). Adapted with permission from ref (50). Copyright 2023, Elsevier.

For depolymerizations, *n*_C–Cscission_ gives more insight into intrinsic catalytic activity than either conversion or product yield. In the example in Figure 3a, the use of liquid yields makes Pt/γ-Al_2_O_3_ appear to be much less active as a catalyst for polyethylene depolymerization than either Pt/Cl-Al_2_O_3_ and Pt/F-Al_2_O_3_, while the latter two catalysts have similar activities. However, Figure 3b reveals that the difference in the average rates of C–C bond scission (*n*_C–Cscission_/*t*, where *t* = 8 h in this case) is about a factor of two between Pt/Cl-Al_2_O_3_ and Pt/F-Al_2_O_3_, similar to the difference between Pt/γ-Al_2_O_3_ and Pt/Cl-Al_2_O_3_. Misleading conclusions from liquid yields arise because the operational definitions for unreacted solids and liquids include large molecular weight ranges which fail to represent the number of catalytic events accurately (see also section 1.2.1.3).

In hydrogenolysis or hydrocracking of saturated POs to produce saturated alkanes, *H_2_consumption* is an independent assessment of the extent of depolymerization. Since H_2_ consumption occurs concurrently with C–C bond scission, both metrics should yield the same information. Furthermore, it may be operationally easier to monitor the evolution of H_2_ pressure. However, when H_2_ redistribution processes such as dehydrogenation, cyclization, and aromatization of the polymer chains occur in parallel with hydrogenolysis and hydrocracking, the relationship between C–C bond cleavage and H_2_ consumption is more complex.^49,50^ To correct for these side-reactions, it is necessary to characterize and quantify the unsaturated hydrocarbon products,^52^ for example, using a combination of NMR, IR and CHN analysis. Alternatively, the degree of unsaturation can be ascertained indirectly by measuring the H_2_ required to fully saturate the hydrocarbons. Consequently, the *number of C–C bond scission events* is a less ambiguous metric.

In oxidative depolymerization, the number of C–C bond scission events can also be calculated if eq 6 is modified to allow *N*(t) to represent the total number of molecules of hydrocarbon oxygenates as well as CO and CO_2_. Alkane metathesis involves only chain length redistribution, so the total number of chains does not change and *n*_C–C scission_ does not reflect the extent of depolymerization.

##### 1.2.2.2 Degree of C–C Bond Scission

The number-average *degree of polymerization* (DP_n_) of a polymer is the number-average number of monomer subunits in a collection of polymer chains. It can be calculated as *M*_n_/*M*_0_, where *M*_n_ is the number-average molecular weight of the polymer chains (measured, for example, by GPC), and *M*_0_ is the monomer molecular weight.^53^ Thus pure monomer has DP_n_ = 1, while a polymer with an average of 1000 monomer subunits per chain has DP_n_ = 1000. An equivalent definition for DP_n_ is *N*(0)/*N*(t), where *N*(0) is the initial charge of monomer to a reactor, and *N*(*t*) is the total number of chains (including unreacted monomers and oligomers) present after addition polymerization proceeds for time *t*. Eqs 7 and 8 relate DP_n_ to the fractional extent of polymerization, *p*.
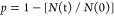
7
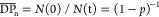
8

Analogously, depolymerization that forms monomer and oligomers of the monomer subunits (effectively, the reverse of addition polymerization) has a fractional extent of depolymerization *q* and a number-average *degree of depolymerization* (DD_n_) as defined in eqs 9 and 10, where *N*(0) is now the initial number of polymer chains (with a number-average number of monomer subunits DP_n_), and *N*(t) is the total number of chains (including unreacted polymer chains and oligomers) present after depolymerization proceeds for time *t*. Normalization by DP_*n*_(0) keeps both values bounded between 0 and 1.
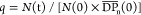
9
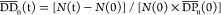
10

Since PO chain scission may generate hydrocarbons other than monomer subunits and oligomers of monomer subunits, DD_n_ and *q* may not be useful as quantitative metrics for PO depolymerization (although they may be appropriate for condensation polymers such as polylactic acid (PLA), which do tend to depolymerize selectively to their constituent monomers). A more general approach allows for products other than monomers by considering all C–C bonds *in the polymer backbone* to be potentially cleavable. For a PO chain, in which each monomer subunit contributes two C–C bonds to the polymer backbone, the maximum possible number of such cleavage events is (2 DP_n_–1). Consequently, we define the number-average *degree of scission* (DS_n_) in eq 11:
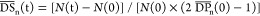
11

**Figure 4:**
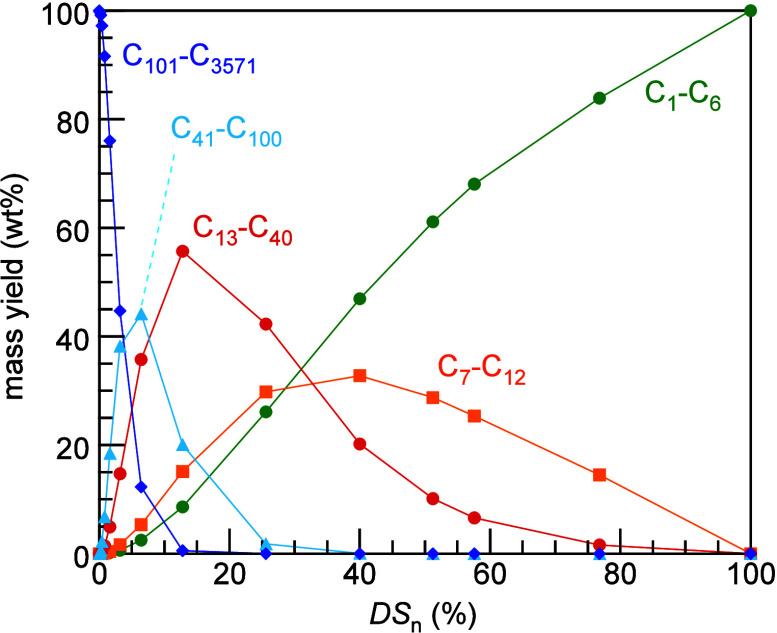
Model of the mass distribution of hydrocarbon product groups resulting from the random hydrogenolysis of a collection of 2,000 linear and atomically-precise PE chains C_3571_H_7144_ (*M* = 50,000 g/mol) as a function of the degree of scission (DS_n_). Assuming that all C–C bonds have an equal chance of undergoing hydrogenolysis implies that the likelihood of a chain being cut is proportional to its length. The mass of H_2_ incorporated was not included in the product mass yields.

Predicted mass distributions for PE chains undergoing random hydrogenolysis are modeled as a function of DS_n_(t) in Figure 4. When DS = 0, the reactor contains only the starting polymer chains; hydrogenolysis of linear PE converts all chains to CH_4_ when DS_n_ = 1.

Finally, an even more general approach acknowledges the possibility of side-chain C–C bond cleavage as well as polymer backbone cleavage. If the number of side-chains is small relative to the degree of polymerization, the effect may be neglected. However, this is clearly not the case for highly branched polyolefins such as PP.

##### 1.2.2.3 Molecular Weight and Dispersity

Due to the broad product distributions that typically result from PO hydrogenolysis, statistical methods are necessary to compare distributions arising from different experiments. Metrics from polymer science such as the number-averaged molecular weight (*M*_n_), weight-averaged molecular weight (*M*_w_), and dispersity (*Đ* = *M*_w_/*M*_n_) can also be used to describe hydrocarbon product distributions obtained in depolymerization reactions.

Inferring catalytic activity from molecular weight distributions must be done cautiously. If all hydrocarbons, including the initial polymer, are included in the product molecular weight distribution, *M*_n_ is inversely proportional to *n*_C–C scission_ (or DS_n_), eq 12.
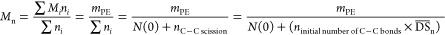
12

**Figure 5:**
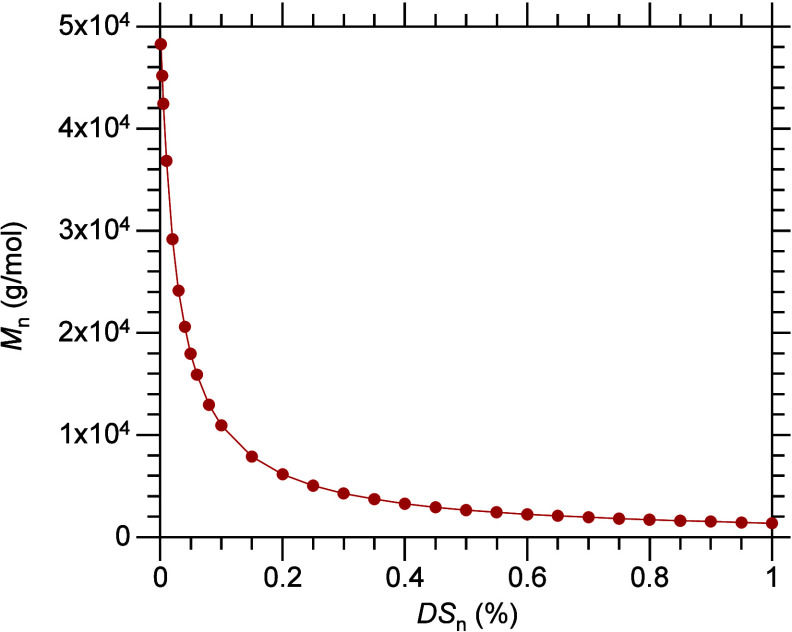
Predicted evolution of the number-averaged molecular weight (*M*_n_) as a function of the average degree of scission (DS_n_), starting from 2,000 chains of a monodisperse PE with a precise *M* value of 50,000 g/mol (i.e., C_3571_H_7144_). The mass of H_2_ incorporated via hydrogenolysis is not included in the product mass.

Initially, *M*_n_ decreases rapidly, but the rate of decrease then slows abruptly. This behavior has been reported in a few studies.^49,54^ The evolution of *M*_n_ for long-chain alkanes undergoing hydrogenolysis is shown as a function of DS_n_ in Figure 5. The highly non-linear change in *M*_n_ with respect to DS_n_ means that the rate of change in average molecular weight slows dramatically as reaction proceeds. This behavior does not indicate catalyst deactivation, nor selectivity for depolymerization products in a particular molecular weight range.

### 1.3 Characterization in Polyolefin Upcycling

#### 1.3.1 Polyolefins as Reactants

For molecular compounds, the name, purity and vendor information usually suffice as descriptors. However, this is not the case for macromolecular materials, whose structures and molecular weights are generally not precise. Providing only the name of a polymer, such as PE or PP, provides information on the empirical chemical composition but is inadequate to predict the behavior of a material in a depolymerization reaction. Slightly more specific designations, such as LDPE, indicate the grade of polymer and imply something about the branch frequency. However, other important properties including the average chain length, the chain length distribution, the type and frequency of branches, and where appropriate, the stereoregularity (tacticity), all require further specification. Many of these properties influence reactivity in depolymerization (see, for example, sections 2.2.2, 3.5.2, 3.5.3, and 5.2.2). To make polymer upcycling reports more reproducible,^55−57^ information beyond the average chain length should be sought and reported. Researchers are encouraged to consult standard works on polymer characterization.^58,59^ Here, we briefly discuss the characterization and specification of macromolecular chain length because this value is used in calculating the number of C–C bond scission events (see section 1.2.2.1).

GPC is a widely used technique to assess macromolecular chain lengths. It requires a calibration curve made using polymer standards, whose molecular weights can range from a few hundred to several million g/mol. The measured elution time of a polymer is related to its molecular weight and molecular weight distribution, using a calibration curve.^60^ Two of the following three values must be reported: the number-average molecular weight *M*_n_, the weight-average molecular weight *M*_w_, and the dispersity *Đ = M*_w_/*M*_n_ (see section 1.2.2.3). A problem specific to POs is the low solubility of high molecular weight PE and PP chains, which precludes their analysis by room-temperature GPC (typically performed in a solvent like THF or CHCl_3_). In such cases, less widely available high-temperature GPC, performed using high-boiling solvents like 1,2,4-trichlorobenzene or decalin, is required.

#### 1.3.2 Polyolefin Upcycling Products

Quantitative analysis is even more challenging for PO upcycling products, because (1) the potential range of molecular weights is very wide, from ca. 10^6^ g/mol for high molecular weight PE, down to ca. 10^2^ g/mol for small molecule products, (2) the number of hydrocarbon product types (e.g., alkanes, alkenes, cycloalkanes, alkadienes, aromatics) as well as oxygenates (e.g., alcohols, aldehydes, ketones, carboxylic acids, dicarboxylic acids) is considerable, and (3) many isomers are possible for each carbon number of a particular product type (branched alkanes, substituted aromatics, etc.).

Gas chromatography (GC) is a convenient way to separate low molecular weight hydrocarbons (from C_1_ up to C_∼40_). Qualitative identification and quantitative analysis can be achieved using electron ionization mass spectrometry (EI-MS) and/or flame ionization detection (FID). Response factors in EI-MS depend on the hydrocarbon type and the number of carbons, requiring calibration curves. In FID analysis, common types of hydrocarbons (e.g., alkanes, alkenes, cycloalkanes, and aromatics) can be assumed to have the same per-carbon response factors.^61^ However, this assumption should not be extended to other types of molecules (e.g., oxygenates). The yield of heavy alkanes (C_*n*_, where *n* > 40) extracted into a solvent (such as CHCl_3_, CH_2_Cl_2_, CS_2_, etc.) may be underestimated in GC analysis due to measurement limitations of the instrument (column, injection temperature, etc). When the mixture contains a large number of isomers with the same or similar carbon numbers, molecular identification can be problematic due to insufficient resolution and/or separation. In such cases, adding a second separation dimension may be helpful (e.g., 2D GC×GC),^62^ although the technique is not yet widely available and requires extensive calibration.

EI is a hard ionization method that induces extensive fragmentation during hydrocarbon analysis. In contrast, techniques that use soft ionization methods, e.g., matrix-assisted laser desorption ionization-time of flight mass spectrometry (MALDI-TOF-MS), field desorption mass spectrometry (FD-MS), and atmospheric pressure chemical ionization mass spectrometry (APCI-MS), can provide more information about the hydrocarbon types present in mixtures, as well as the molecular weight distributions for heavier hydrocarbons (MALDI-TOF: up to 150,000 g/mol, FD-MS: up to 12,000 g/mol, and APCI-MS: up to 1,500 g/mol). However, isomers with the same molecular weight are not separated, and product families belonging to the same mass series overlap (e.g., *n*-alkanes and alkylnaphthalenes). Furthermore, these techniques are generally only semi-quantitative, because compounds with lower molecular weights may be preferentially removed when the sample is prepared under vacuum, while compounds with higher molecular weights may not be sufficiently soluble and/or volatile.

For oligomers (C_>40_) and partially converted polymers, GPC can be used to assess the molecular weight distribution. This technique is typically considered reliable for compounds with molecular weights above ca. 10^3^ (i.e., C_>100_), where the refractive index becomes essentially independent of molecular weight.^63^ In a typical polymer upcycling process, waxy oligomers (C_∼40_ to C_∼100_) and even heavy liquids (C_∼10_ to C_∼40_) are also present. They may not be properly assessed by GPC, influencing the accuracy of the average molecular weight determination for the entire sample.

Dispersity or *Đ* values (i.e., *M*_w_/*M*_n_) obtained from GPC measurements are subject to the same limitations. In a non-living polymerization process, the value of *Đ* increases with molecular weight. Conversely, *Đ* decreases in a depolymerization process (although an initial increase may be observed before the decreasing trend is established).^49,54^ Since GPC measurements have a low molecular weight cutoff (C_∼10_) due to overlap with the solvent peak, measured *Đ* values can be underestimated. *Đ* values obtained from GPC measurements should be used with cto compare and make claims about product distributions requires care.

To obtain accurate molecular weights from GPC measurements, polymer standards must have a chemical structure similar to that of the macromolecules being analyzed. When their chemical structures differ, the Mark–Houwink equation can be used to interconvert molecular weights measured with different standards.^60^ For example, linear alkane waxes generated via PE hydrogenolysis can be analyzed by GPC using common PS calibration standards, but the results must be adjusted to obtain values that correspond to calibration with PE standards. When upcycling generates products with chemical properties that are very different from the original polymer (e.g., PE conversion to long-chain alkylaromatics, or PE oxidation to oxygenated waxes), changes in the hydrodynamic volumes of the products relative to the standards affect retention times and make the extracted molecular weight values less reliable.

Solution-state ^1^H and ^13^C nuclear magnetic resonance (NMR) spectroscopy can provide complementary information about the types of hydrocarbons present in product mixtures. These methods provide only an overall assessment, since different hydrocarbon types often exhibit overlapping signals (especially ^1^H signals, due to their small chemical shift range). They are also limited to compounds with sufficient solubility. The larger chemical shift range for ^13^C relative to ^1^H can be useful, but sensitivity can be an issue due to the low natural abundance (0.1 at%) of ^13^C. Increased sensitivity can be achieved with ^13^C isotope labeling, but requires additional expense and/or synthetic effort. To ensure NMR information is quantitative, the relaxation delay used in the NMR data acquisition needs to be long enough to allow for complete spin-lattice relaxation. The appropriate delay can be ascertained using inversion recovery measurements.^64^ Alternatively, a paramagnetic relaxation agent such as Cr(acac)_3_ can reduce spin-lattice relaxation times and improve the measurement efficiency.^65^ Some characterization of poorly soluble or insoluble solids can be achieved using solid-state magic-angle spinning (MAS) NMR, although the resolution is typically much lower than for solution-state NMR. Melt NMR requires higher temperatures, but may provide better sensitivity since there is no solvent to dilute the polymer and products.^66^*Operando* MAS-NMR equipment is not currently compatible with the elevated temperatures typically required in depolymerization experiments (>250 °C),^67^ but this technique may be useful in lower temperature studies, and higher temperature compatibility may become available in the future.

## 2 Catalytic Hydrogenolysis of Polyolefins

### 2.1 Introduction to Alkane Hydrogenolysis

Hydrogenolysis refers to C–C bond scission in the presence of H_2_. Among all the strategies reported to upcycle POs, hydrogenolysis is one of the simplest, both chemically and operationally. It leads directly to saturated hydrocarbons, which may find uses as lubricants, waxes, and solvents, depending on the molecular weight distribution and branching level.^30,51,68^ It can also generate fuels (e.g., jet, diesel, gasoline, and naphtha). This section will include the latter processes, since they are often claimed as targets of PO hydrogenolysis although they do not technically qualify as upcycling (see section 1.1). Hydrogenolysis is thermodynamically accessible at relatively mild temperatures (<300 °C). Nevertheless, the process consumes considerable amounts of energy-intensive H_2_, currently produced most efficiently by steam reforming of methane.

In contrast to hydrocracking, which occurs when a catalyst also contains strong acid sites (see section 3), pure hydrogenolysis is generally conducted in the absence of significant catalyst acidity, resulting in little skeletal isomerization of the hydrocarbon as the reaction proceeds. Methane is not a typical hydrocracking product (since formation of CH_3_^+^ is thermodynamically highly unfavorable),^69,70^ but PO hydrogenolysis can produce considerable amounts of methane via terminal C–C bond cleavage and/or removal of methyl branches. Research to-date has focused on improving hydrogenolysis activity, narrowing the product molecular weight distribution and limiting the formation of methane and other light alkanes by regulating the size of the metal nanoparticles and/or the nature of the support material, as well as by using confinement effects.

**Scheme 3:**
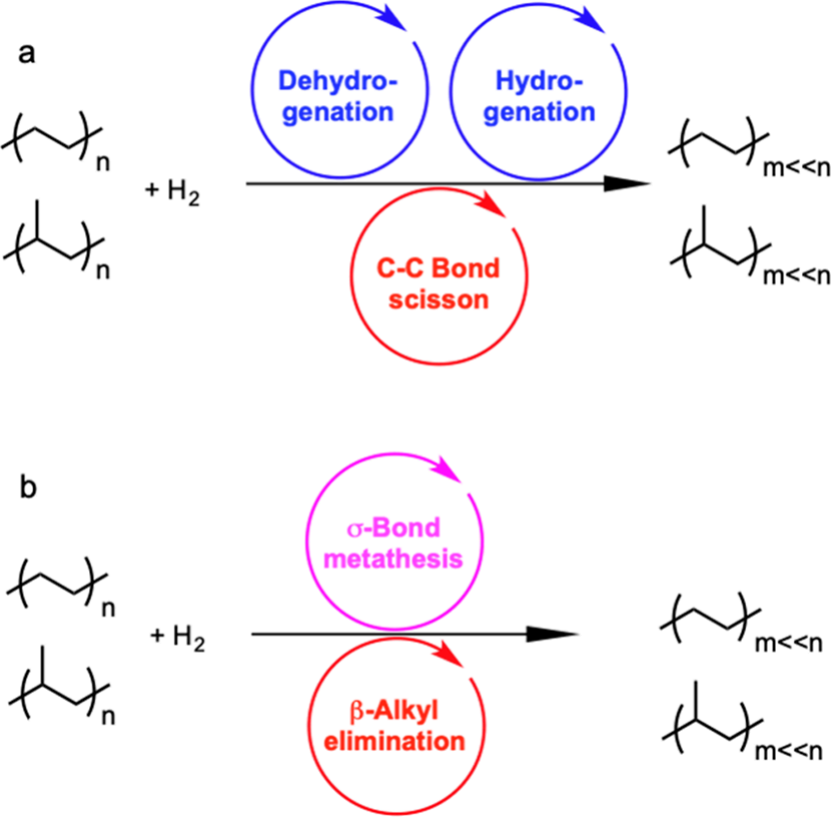
Principal Steps in PE and PP Hydrogenolysis Catalyzed by (a) Late Transition Metal Catalysts; and (b) Early Transition Metal Hydride Catalysts

In most studies, the catalysts investigated for PO hydrogenolysis are solids consisting of metal nanoparticles dispersed on non-acidic supports.^18^ Such catalysts are considered to be monofunctional. The most efficient catalysts are generally based on the noble metals Ru and Pt. However, there are also reports of non-noble metal catalysts, typically first-row transition metals in their metallic states (e.g., Co, Ni). The mechanism of hydrogenolysis by metal catalysts involves activation of aliphatic C–H bonds, dehydrogenation, and C–C bond scission in the adsorbed hydrocarbon intermediates. Adsorbed H atoms react with the adsorbed hydrocarbons to generate shorter alkanes. The overall reaction is depicted in Scheme 3a.

In addition, early transition metals in high oxidation states (e.g., hydrides of Zr, Ta, and Hf) catalyze PO hydrogenolysis under low H_2_ pressures (<2 bar). The general mechanism involves C–H bond activation by σ-bond metathesis followed by β-alkyl elimination, Scheme 3b. Since the metal nanoparticle-catalyzed process is more common in published research to-date, the mechanistic discussion will focus on it in the next section. Early transition metal hydride-catalyzed hydrogenolysis is discussed in section 2.3.5 below.

#### 2.1.1 Mechanism of C–C Bond Cleavage

Prior to the recent explosion of interest in catalytic hydrogenolysis of POs, metal-catalyzed hydrogenolysis of shorter saturated hydrocarbons had been widely investigated due to its relevance in the cracking of heavy hydrocarbons derived from crude oil to make fuels, and its importance as an undesired side-reaction in light alkane dehydrogenation.^71−73^ When accompanied by C–H bond formation, C–C bond cleavage is exothermic, and strongly favored thermodynamically over a wide range of reaction conditions.

**Scheme 4:**
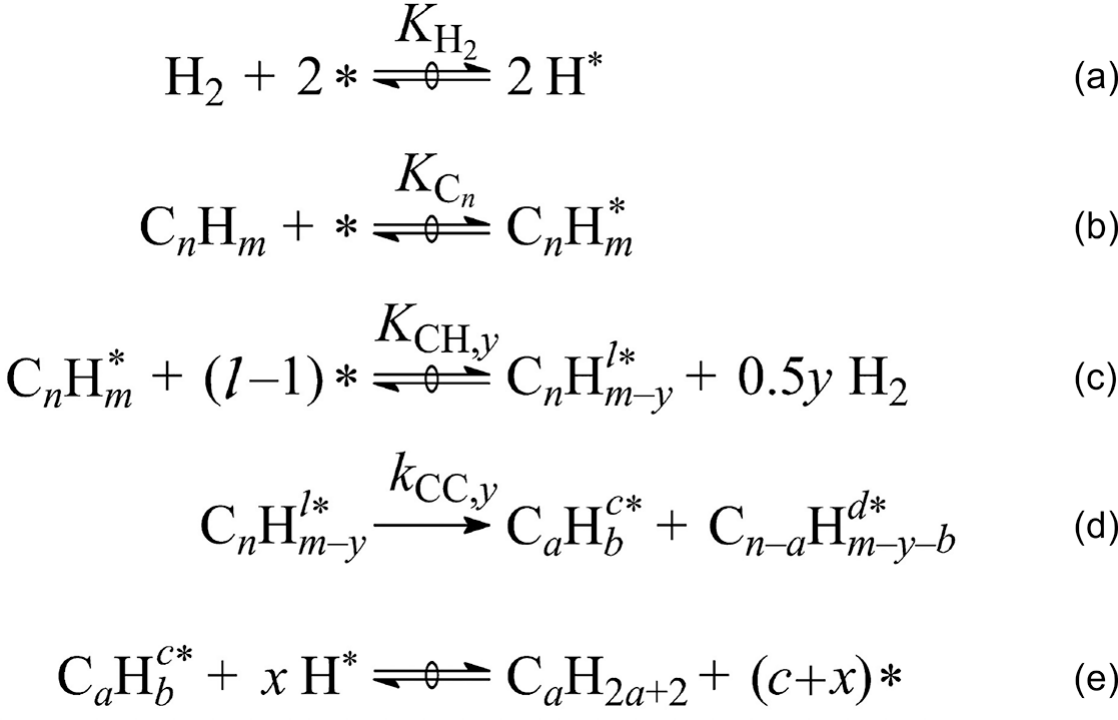
Elementary Steps and Intermediates in Alkane Hydrogenolysis Catalyzed by Metal Surfaces All steps except for (d) are considered to be quasi-equilibrated. * denotes a vacant surface site; l* indicates that the adsorbate occupies *l* surface sites. *K_x_* and *k_y_* are equilibrium and rate constants, respectively, for individual steps; *a*/*b* indicate numbers of C/H atoms, respectively, in the cleaved fragments. Reproduced with permission from ref (75). Copyright 2016, American Chemical Society.

The microkinetic model in Scheme 4 was developed for small alkane hydrogenolysis based on kinetic measurements combined with computational studies.^74,75^ The first, quasi-equilibrated steps are H_2_ dissociation on adjacent vacant metal sites to form adsorbed H (H^*^, step a) and alkane adsorption (step b). A number of subsequent, quasi-equilibrated dehydrogenation steps (grouped in step c) generates a pool of partially dehydrogenated adsorbed intermediates (C*_n_*H^l*^_m–y_, representing alkyls, alkylidenes, etc., where *l* is the number of surface sites needed to adsorb the intermediate).^76^ Some of these intermediates can be difficult to distinguish spectroscopically due to their low abundance on H*-covered surfaces, but their nature can be inferred indirectly from experimental studies and theoretical predictions. For example, in ethane hydrogenolysis on Pt(211), lowering the H* coverage on Pt causes the dominant surface species to change from CH_3_CH_2_* to CH_3_CH* (where * represents metal–carbon bonding of unspecified bond order).^77^ C–C bond cleavage may occur in any of the intermediates to generate two shorter hydrocarbon fragments (step d); this step is generally rate-determining. Finally, alkanes are released when adsorbed hydrocarbons combine with adsorbed H (step e). Competitive adsorption of H reduces the coverage of adsorbed dehydrogenated intermediates, thus the rate of C–C bond cleavage is inhibited by high H_2_ pressures.^77^

This general mechanism is consistent with the experimental kinetics of ethane hydrogenolysis, as well as DFT calculations that predict C_2_H_6_ undergoes quasi-equilibrated dehydrogenation, forming multiple dehydrogenated intermediates.^78^ For example, experiments verified that C–C bond cleavage in an *CHCH* intermediate dominates the observed rate of ethane hydrogenolysis catalyzed by Ir/SiO_2_ (7 nm nanoparticles, 18 bar H_2_, 320 °C).^74^

While the elementary steps are general for metal-catalyzed hydrogenolysis, the overall reaction orders depend on the identity of the metal, the H_2_ pressure, the alkane, and the temperature, which together determine the extent of dehydrogenation in adsorbed hydrocarbons.^79^ For example, from 150 to 350 °C the reaction order in ethane is +1.0 for various M/silica catalysts (M = Co, Ni, Ru, Rh, Pd, Os, Ir, Pt), while the reaction order in H_2_ is strongly negative due to competitive adsorption of H* and hydrocarbons.^80−82^ The reported orders for H_2_ are −3.0 for Ir (0.7 nm nanoparticles), −2.3 for Pt (0.6 nm nanoparticles), −3.1 for Rh (0.9 nm nanoparticles), and −3.0 for Ru (1.0 nm nanoparticles).^71,72,83,84^ However, at very low H* coverages, the reaction order in H_2_ may become positive even for noble metals.^51,76^ At a H* coverage of ca. 1 monolayer, the most abundant surface hydrocarbons depend on the identity of the metal: for Group 8–9 metals, the surface is covered mainly by deeply dehydrogenated species (e.g., *CHCH*), while multiple species (e.g., *CHCH* and CH_3_CH*) coexist on Group 10 metal surfaces, and Group 11 metal surfaces are occupied mainly by the relatively saturated alkyls (e.g., CH_3_CH_2_*).^83^

#### 2.1.2 Hydrogenolysis of Model Alkanes

Studies of small molecule hydrogenolysis can provide more detailed insight into the mechanism of PO hydrogenolysis, by simplifying reaction networks and product analysis, compared to experiments with the actual POs. Hydrocarbons that have been used recently for the purpose of understanding PO hydrogenolysis include those with very small carbon numbers, such as *n*-butane,^85^*n*-hexane, and 2-methylpentane,^86−88^ as well as those with intermediate carbon numbers, such as *n*-hexadecane,^88^*n*-octadecane,^46^*n*-eicosane (C_20_),^89^*n*-hexacosane (C_26_),^90^ and squalane (C_30_).^88,91,92^ The choice of hydrocarbon chain length is significant. As chain length increases (up to C_10_), the strength of *n*-alkane adsorption on a metal surface (e.g., Pt(111)), increases approx. linearly with carbon number.^93^ For longer hydrocarbons, the dependence of the adsorption strength (based on the Gibbs’ energy difference between gas phase and adsorbed forms of the *n*-alkane) on chain length deviates from linearity, becoming sub-linear for C_*n*>12_ due to significant contributions from the conformational variations of long-chain alkanes.^94^

DFT calculations based on the microkinetic model in Scheme 4 predict that C–C bond cleavage in C_2_–C_10_ linear alkanes catalyzed by Ir(111) occurs via α,β-bound intermediates RC*–C*R (R = H or C_*y*_H_2*y*+1_), formed by removal of H atoms from adjacent C* sites.^75^ The activation enthalpies are lower and activation entropies higher for the cleavage of longer chains relative to shorter chains, such that the experimental rate of cleavage for *n*-decane catalyzed by 0.7 nm Ir clusters is ca. 10^8^ times higher than for ethane at 300 °C and H_2_/alkane = 5. Since internal C–C bond cleavage leads to two long hydrocarbon fragments and a corresponding increase in entropy, the thermodynamic preference for internal C–C bond cleavage over terminal C–C bond cleavage increases with chain length.

The hydrocarbon product distribution in the ring-opening hydrogenolysis of methylcyclopentane catalyzed by Pt/Al_2_O_3_ at 300 °C was reported to be particle size-dependent. For Pt nanoparticles below 1.5 nm, *n*-hexane was formed preferentially relative to 2-methylpentane or 3-methylpentane.^87^ The estimated abundance of corner atoms on Pt nanoparticles of various sizes was found to correlate well with the *n*-hexane yield. Metallacyclobutane intermediates which form *n*-hexane by C(2°)–C(2°) bond cleavage are stabilized on corner sites, providing an example of how catalyst structure influences the location of C–C bond cleavage.

The complexity of describing alkane hydrogenolysis for a wide range of hydrocarbon chain lengths and reaction conditions can be illustrated with an example.^95^ Ru/C (5 wt% Ru) catalyzed the hydrogenolysis of *n*-octadecane under 30 bar H_2_ at 240 °C in a batch reactor. Over the course of 14 h, the carbon numbers of the products shifted gradually from C_8_–C_17_ to lower values, and finally to CH_4_, suggesting random cleavage of both internal and terminal C–C bonds.^46^ However, hydrogenolysis of *n*-eicosane catalyzed by Ru/TiO_2_ (1.5 wt% Ru) in a continuous flow reactor under 1 bar H_2_ at 200 °C gave C_1_ and C_15_ alkanes as the most abundant products, reflecting a preference for terminal chain cleavage. Hydrogenolysis of *n*-hexacosane catalyzed by Ni/SiO_2_ under 30 bar H_2_ at 300 °C also favored terminal chain cleavage, with the most abundant products initially being C_1_ and C_25_ alkanes.

Branches located along the main alkane chain (*aka* the “backbone”) affect the rates of C–C bond hydrogenolysis. DFT calculations involving the surfaces of noble metal (e.g., Ir, Rh, Ru, and Pt) predict that intermediates are formed by activation of vicinal C–H bonds, and are cleaved in metallacyclic α,δ- or α,β,γ-bound transition states.^86,96^ Consequently, activation barriers are larger when the C–C bond being cleaved involves at least one sterically-hindered tertiary carbon, i.e., C(3°)–C(*x*°), compared to C(2°)–C(1°) or C(2°)–C(2°) bonds.

**Figure 6:**
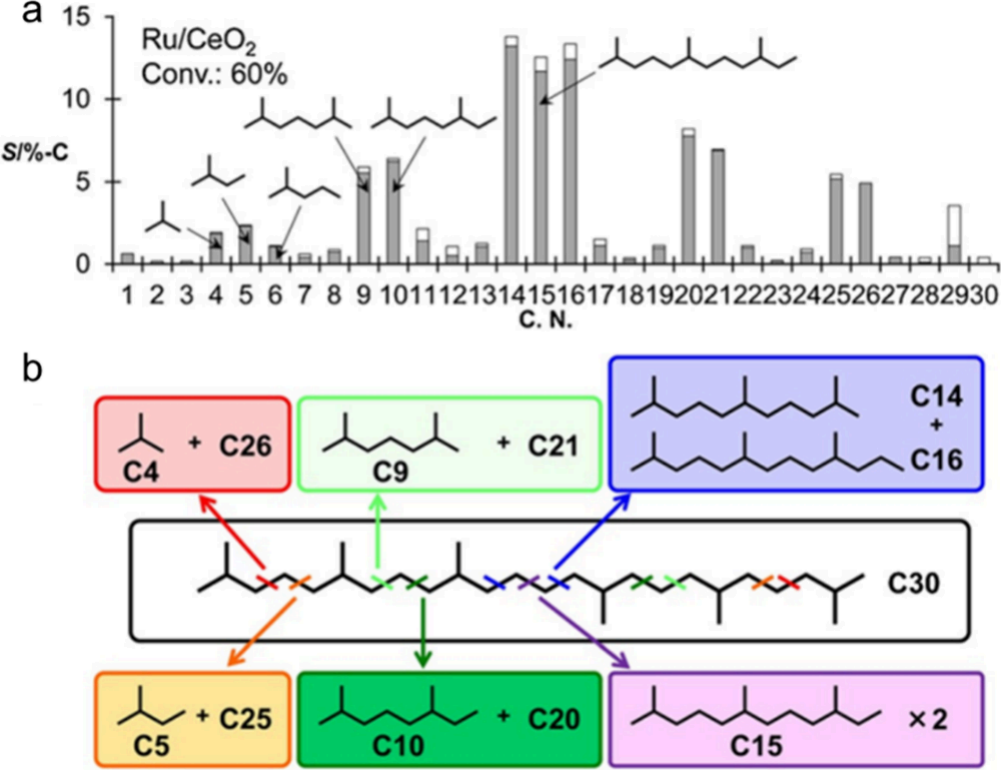
Results of squalane hydrogenolysis: (a) product selectivity; and (b) proposed C–C bond cleavage positions, based on product analysis. Reaction conditions: 4.23 g squalane, 50 mg Ru/CeO_2_, 60 bar H_2_, 240 °C, 6 h, batch reactor. In part (a), gray and white bars represent yields of dominant products and other isomers, respectively. Products with 4, 5, 9, 10, 14, 16, 20, 21, 25, or 26 carbons result from C(2°)–C(2°) bond cleavage, while products with 1, 3, 6, 8, 11, 13, 17, 19, 22, 24, 27, or 29 carbons result from C(3°)–C(*x*°) bond cleavage. Reproduced with permission from ref (91). Copyright 2016, Wiley.

The regioselectivity of C–C bond cleavage in alkane hydrogenolysis was studied using squalane, a methyl-branched isomer of C_30_H_62_. Ru/CeO_2_ (5 wt% Ru, as ca. 1.5 nm nanoparticles) was chosen as the catalyst, due to its high preference for non-terminal C–C bond cleavage in *n*-hexadecane (in contrast to Ir/C, Pd/C, Ru/C, and Ru/SiO_2_).^91^Figure 6a shows the various squalane fragments obtained under 60 bar H_2_ at 240 °C in 6 h. All possible C(2°)–C(2°) cleavage positions are shown in Figure 6b. The experimental product distribution was deemed to arise due to selective cleavage of C(2°)–C(2°) bonds between methyl branches, without isomerization. *P*_H_2__ also plays a role in the regioselectivity of C–C bond hydrogenolysis. At similar conversions, the preference for cleavage of internal C(2°)–C(2°) bonds in squalane, *n*-hexadecane, and *n*-hexane increased with *P*_H2_ from 20 to 60 bar.^92^

In general, there are three kinds of hydrogenolysis sites: (1) internal C(2°)–C(2°) bonds, which are relatively sterically unhindered, (2) sterically-hindered C(3°)–C(1°) bonds, and (3) terminal C(2°)–C(1°) bonds. When a site (3) cleavage occurs successively, methane is produced selectively. Relative to site (1), cleavage at either sites (2) or (3) generates lower-value small molecules, and shows more negative reaction orders in H_2_. Higher *P*_H2_ (e.g., 60 bar) suppresses cleavage at sites (2) and (3) as well as undesired side-reactions, at the expense of the overall hydrogenolysis rate. Incorporating a second metal (e.g., V) into a supported Ru catalyst produced a similar effect (i.e., suppression of methane production and overall hydrogenolysis activity),^89^ presumably by modulating the relative abundances of surface intermediates.

### 2.2 Hydrogenolysis of Polyolefins

#### 2.2.1 General Mechanism

**Scheme 5:**
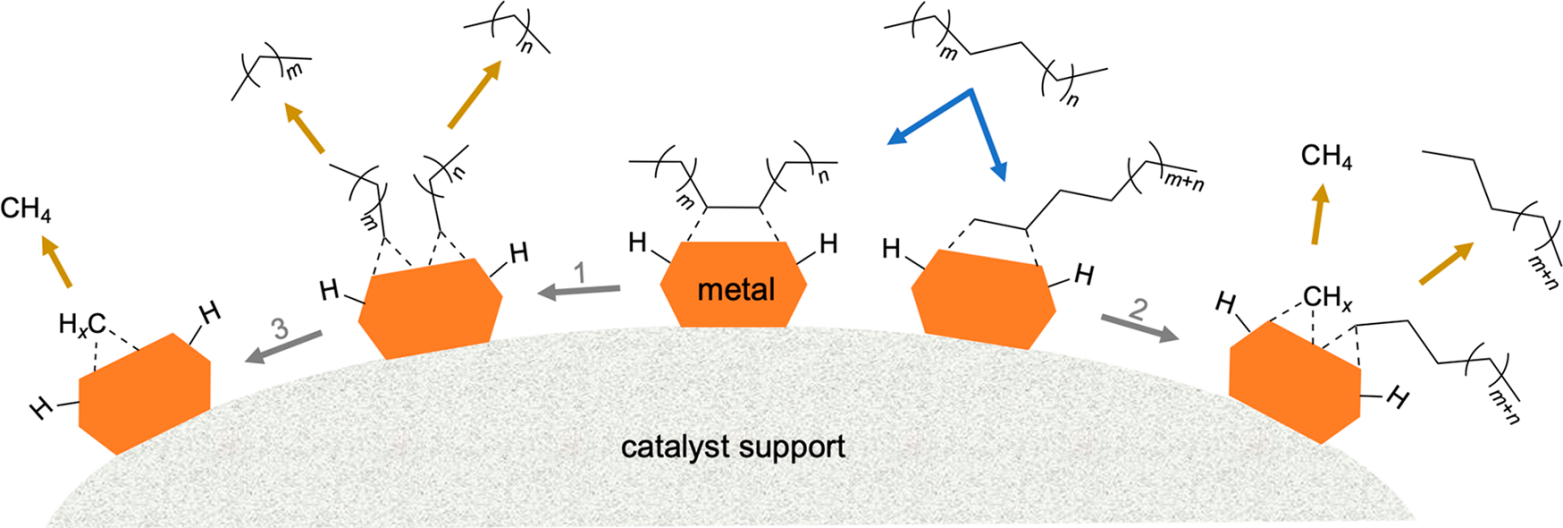
General Mechanism for PO Hydrogenolysis Catalyzed by a Metal Surface, with Types of C–C Bond Scissions Classified As: (1) Internal Scission (Type 1); (2) Terminal Scission (Type 2); and (3) Successive Scission (Type 3, Referring to Multiple Scission Events for a Particular Adsorbed Species) “Bonds” between the metal surface and polymer fragments (indicated by dashed lines) are shown for illustration purposes, and are not intended to be chemically precise.

A general mechanism for PO hydrogenolysis catalyzed by a metal surface is depicted in Scheme 5, based on our understanding of small alkane hydrogenolysis.^75^ Polymer dehydrogenation can occur at either internal or terminal sites, resulting in various adsorbed species. Type 1 scission leads to internal chain cleavage, producing two shorter chain fragments. Type 2 scission leads to terminal C–C bond cleavage, resulting in demethylation. Both of these scenarios involve a single chain cleavage event. However, if an adsorbed intermediate remains on the surface, subsequent cleavage, called Type 3 scission, can occur. The ultimate product of this reaction is low-value methane. To limit hydrogenolysis products to a narrow molecular weight range and generate more valuable products (e.g., lubricants, C_15_–C_50_; or fuels: gasoline, C_5_–C_12_; diesel, C_9_–C_22_), avoiding the production of light alkanes, Type 1 scission must be promoted while Types 2 and 3 scissions must be suppressed.^97−101^

The much higher molecular weights of PO macromolecules relative to small molecule alkanes (C_2_–C_10_) affect the kinetics of hydrogenolysis. As noted in section 2.1.2, adsorption energies for alkanes on metal surfaces increase with chain length,^93^ due to increased van der Waals interactions.^51^ The stronger interactions between longer chains and metal sites alter the competition with adsorbed H atoms. Reaction orders with respect to H_2_ are less negative for longer alkanes compared to light alkanes, since the higher stability of adsorbed long-chain intermediates can result in hydrogenation and/or desorption being rate-determining, instead of C–C bond cleavage. Kinetic simulations predict that when adsorbed intermediates are abundant, hydrogenolysis should become zeroth-order in alkane and first-order in H_2_.^102^ Some experimental studies report that reaction orders in H_2_ are only slightly negative for PO hydrogenolysis.^46,103^

**Scheme 6:**
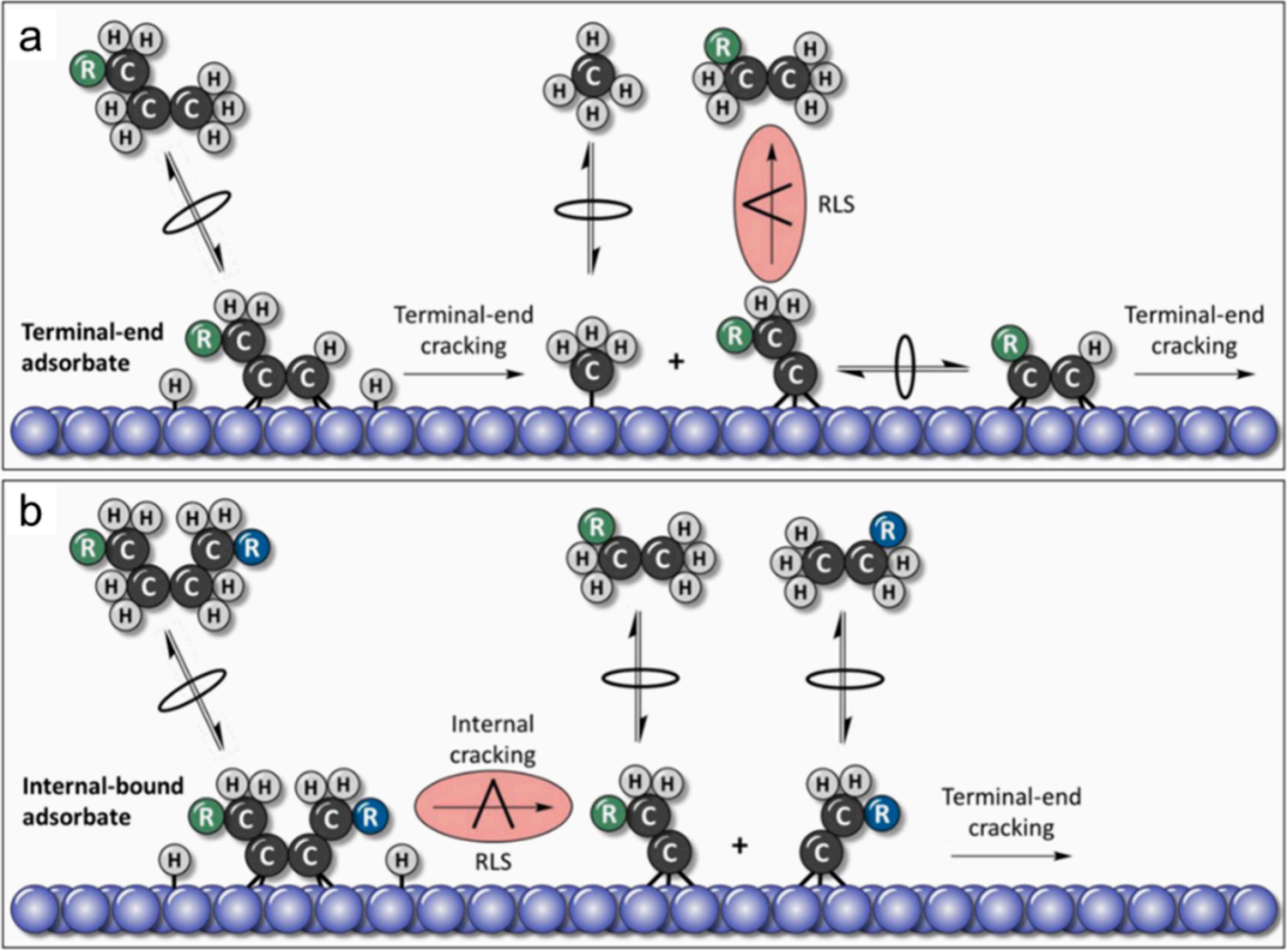
Location-Dependent Kinetics of PE Hydrogenolysis, Catalyzed by Ni/SiO_2_: (a) Cleavage of a Terminal C–C Bond, with Rate-Determining Desorption; and (b) Cleavage of an Internal C–C Bond, with Rate-Determining C–C Bond Cleavage Adapted with permission from ref (90). Copyright 2023, Elsevier.

Hydrogenolysis of *n*-hexacosane (C_26_) catalyzed by Ni/SiO_2_ (15 wt% Ni) is consistent with the mechanism pictured in Scheme 5. The products are dominated by methane, but the yield of *n*-pentacosane (C_25_) is just 20 % of the methane yield (300 °C, 30 bar H_2_, 2 h). The proposed reaction mechanism is comprised of multiple scission events involving terminal (Scheme 6a) and internal carbons (Scheme 6b). The product molecular weight distribution was simulated using stochastic modeling,^104^ assuming (1) terminal C–C bond cleavage is 25× faster than the internal C–C bond cleavage (consequently, the rate-limiting steps are desorption and C–C bond scission for terminal and internal cracking, respectively), based on observations of *n*-hexane hydrogenolysis catalyzed by Ni/SiO_2_,^105^ and (2) the rate of alkane desorption is inversely proportional to its chain length.^93,94^ All three pathways: internal C–C bond cleavage, terminal C–C bond cleavage, and successive terminal C–C bond cleavage, must be included for the model to simulate the hydrogenolysis product distribution well, supporting the simultaneous occurrence of various chain scission mechanisms. Compared to *n*C_26_, even a short PE chain (*M*_w_ = 4,000 g/mol, *Đ* unspecified) had a much higher (10×) abundance of internal C–C bonds and a higher probability of adsorbing on the Ni surface via one of its internal C–C bonds. Nevertheless, at similar conversions, hydrogenolysis of this PE generated approx. 4× more methane than *n*C_26_, implying that successive C–C bond scission occurs and produces methane until the shrinking hydrocarbon chain becomes short enough to desorb.^90^

#### 2.2.2 Effect of Polyolefin Microstructure

POs differ in their molecular weights and molecular weight distributions, branch lengths, branch frequencies, and stereoregularities, leading to differences in melting points, viscosities, diffusivities, and mobilities. It is important to understand how these factors impact hydrogenolysis kinetics, as well as the hydrocarbon product distribution. PP contains a large number of methyl branches, and a backbone comprised of C(3°)–C(2°) bonds, compared to mostly C(2°)–C(2°) bonds in the backbone of PE. Hydrogenolysis will therefore generate more *iso*-alkanes from PP than from PE.^106^

The effect of branching on PO hydrogenolysis reactivity is complex and not yet well-understood. While the C(3°)–C(2°) bonds in PP have lower bond dissociation energies than the C(2°)-C(2°) bonds in PE,^107^ some studies report that PP is more reactive than PE. For example, isotactic polypropylene (iPP, *M*_n_ = 12,000 g/mol, *Đ* unspecified) was almost fully converted in 2 h by a Ni/SiO_2_ (15 wt% Ni) catalyst at 300 °C under 20 bar H_2_, while PE (*M*_n_ = 4,000 g/mol, *Đ* unspecified) was only 65 % converted in the same time. The increased reactivity of PP was attributed to the presence of tertiary carbons on the backbone, and their ability to undergo hydrogenolysis by activation of weaker C(3°)-H bonds.^90^ The high extent of hydrogenolysis catalyzed by PtRe/SiO_2_ for poly(ethylene-*alt*-propylene) (PEP, *M*_n_ = 127,000 g/mol, *Đ* = 1.04), relative to linear PE (*M*_n_ = 128,000 g/mol, *Đ* = 3.3) after 17 h at 170 °C under 30 bar H_2_, was also attributed to the abundance of tertiary carbons due to short-chain branching in the PEP.^108^ However, this comparison did not take into account the large difference in melt viscosities of the two polymers.^109^

In contrast, other studies report higher hydrogenolysis reactivity for PE relative to PP. For instance, “full” conversion of iPP (*M*_w_ = 12,000 g/mol, *M*_n_ = 5,000 g/mol) catalyzed by Ru/CeO_2_ (5 wt% Ru) was achieved after 72 h at 240 °C under 60 bar H_2_, while HDPE (*M*_w_ = 64,000 g/mol, *M*_n_ = 12,000 g/mol) required just 10 h. The difference was ascribed to steric hindrance due to the highly branched structure of PP.^110^ A similar conclusion was made for hydrogenolysis of the two polymers catalyzed by Ru/TiO_2_.^111^

The effect of PE microstructure on hydrogenolysis catalyzed by Pt/SrTiO_3_ (10 wt% Pt, 2 nm Pt nanoparticles) was investigated.^112^ After 72 h at 300 °C under 12 bar H_2_, the conversion was higher than 99 % for initial *M*_n_ values from 7,600 to 51,000 g/mol. The liquid hydrocarbon products (i.e., those soluble in *n*-hexane) had essentially indistinguishable molecular weights (*M*_n_ = 550 g/mol), dispersities (*Đ* ∼ 1.4) and branching frequencies (160–180 per 1000 C). However, LLDPE with hexyl branches gave a higher gas yield (C_1_–C_8_) than LLDPE with ethyl branches, presumably due to preferential C–C bond scission of the branches, compared to the backbone.^112^

**Figure 7:**
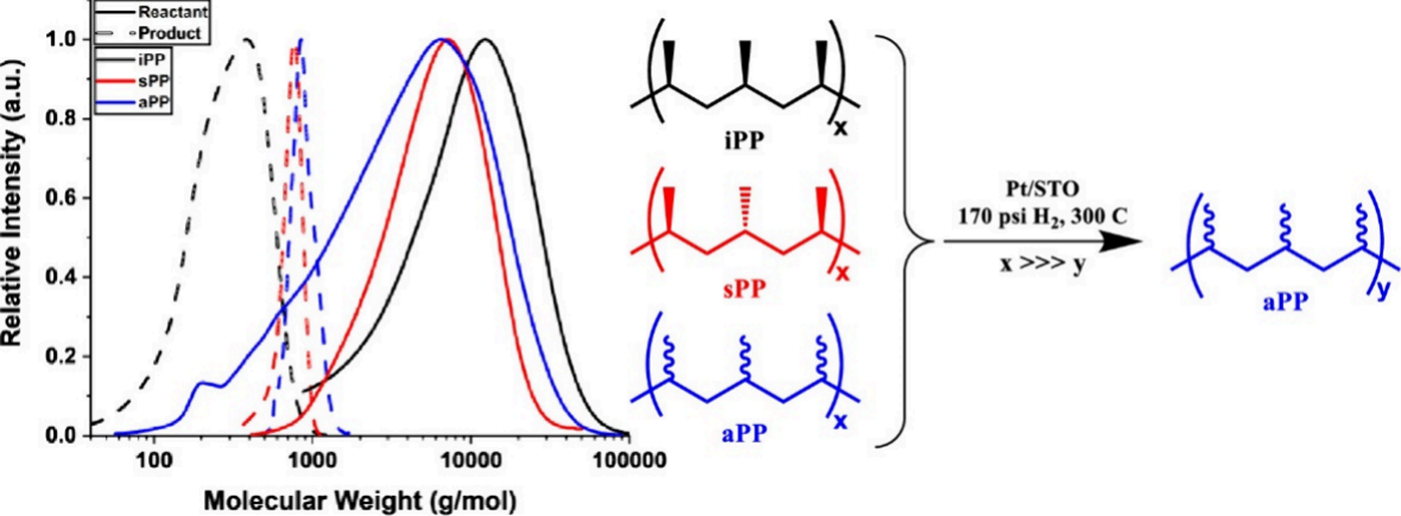
GPC molecular weight distributions for various polymers (solid lines) and their hydrogenolysis products (dashed lines) derived from atactic (aPP), isotactic (iPP), and syndiotactic polypropylene (sPP). Reaction conditions: Pt/SrTiO_3_ (10 wt% Pt), 300 °C, 170 psi H_2_, 72 h. Reprinted with permission from ref (112). Copyright 2022, American Chemical Society.

Striking variations in results were found for PP hydrogenolysis, depending on its stereochemistry. Under the same reaction conditions, atactic (aPP, *M*_n_ = ca. 1,600 g/mol, *Đ* = 4.2), isotactic (iPP, *M*_n_ = ca. 6,000 g/mol, *Đ* = 2.2), and syndiotactic polypropylenes (sPP, *M*_n_ = ca. 4,250 g/mol, *Đ* = 1.7) gave liquid products with significantly different molecular weights (Figure 7). Specifically, iPP underwent much more extensive chain scission (down to *M*_n_ ∼ 250 g/mol, *Đ* = 1.4) compared to aPP and sPP, both of which gave heavier liquid products (*M*_n_ ∼ 800 g/mol, *Đ* = 1.1). The authors suggested that iPP chains tend to adopt helical conformations due to the identical stereochemistry of adjacent methyl branches, leading to lower chain mobility and stronger interactions with the catalyst surface. Presumably, more strongly adsorbed intermediates undergo more extensive reaction. Racemization of stereocenters and skeletal rearrangements were evident in the ^13^C{^1^H} NMR spectrum of the liquid products, but not the polymer.^112^ The rate of racemization in the small-molecule products was inferred to be much slower than the rate of PP hydrogenolysis.

**Figure 8:**
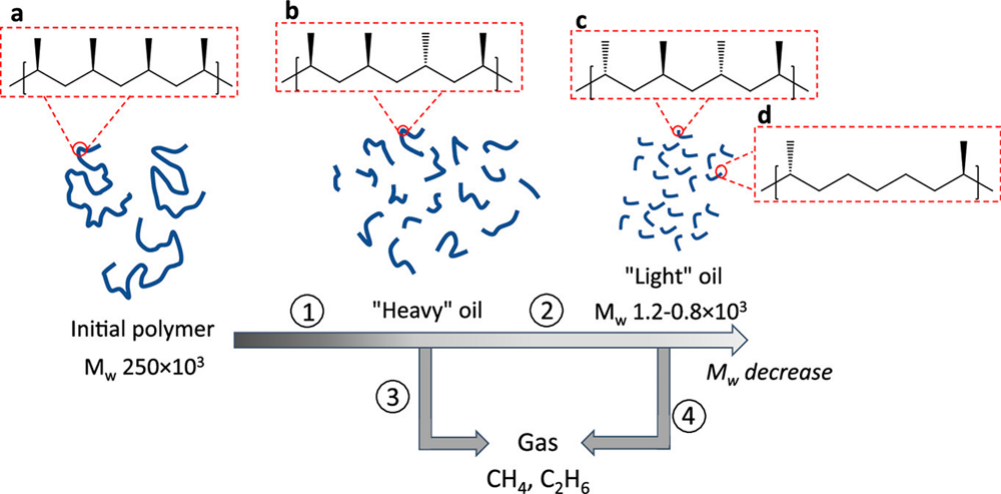
Progress of iPP hydrogenolysis, catalyzed by Ru/TiO_2_: (a) initial polymer; (b) heavy oil, with lower stereoregularity than the initial polymer; (c) light oil, with even lower stereoregularity; and (d) light oil, with loss of CH_3_ branches due to demethylation. Reprinted with permission from ref (113). Copyright 2021, American Chemical Society.

Catalysts based on a variety of transition metals and metal oxide supports were screened in the hydrogenolysis of iPP (*M*_w_ 250,000 g/mol, *M*_n_ 67,000 g/mol) at 250 °C under 30 bar H_2_.^113^ After 16 h, Ru/TiO_2_ (5 wt% Ru) gave the lowest yield of light alkanes and the highest fraction of liquid oil (66 %), consisting mostly of lubricant-range hydrocarbons with a fairly narrow molecular weight distribution. The time dependence of the product distribution was investigated. Hydrogenolysis initially produced a heavier oil, which was subsequently converted to a lighter oil. Gases were secondary products, arising from the heavy oil at early reaction times as well as from the light oil at later reaction times. Gradual down-shifting of the molecular weight distribution indicated that hydrogenolysis occurred through multiple adsorption–desorption events, resulting in the progression shown in Figure 8. To examine the effect of feedstock variability, iPP (*M*_w_ = 12,000 g/mol, *M*_n_ = ca. 5,000 g/mol), aPP (*M*_w_ and *M*_n_ not specified), and post-consumer PP bags were tested. All gave similar product molecular weight distributions. The lubricant properties (kinematic viscosities and pour points) of the hydrogenolysis products derived from iPP were deemed comparable to those of standard commercial products.^113^

^1^H and ^13^C NMR chemical shifts characteristic of CH, CH_2_, and CH_3_ groups revealed that the PP chains lost their high isotacticity as the reaction proceeded, although stereoregularity decayed relatively slowly, over 20 h. It was completely absent in the oil products. Replacement of H_2_ by D_2_ had little effect on the amount of solid residue, indicating that the rate of iPP consumption was unaffected by the isotopic substitution. However, D_2_ caused the molecular weight of liquid oil to increase, implying that hydrogenation/dehydrogenation steps became rate-determining in heavy oil hydrogenolysis.

**Figure 9:**
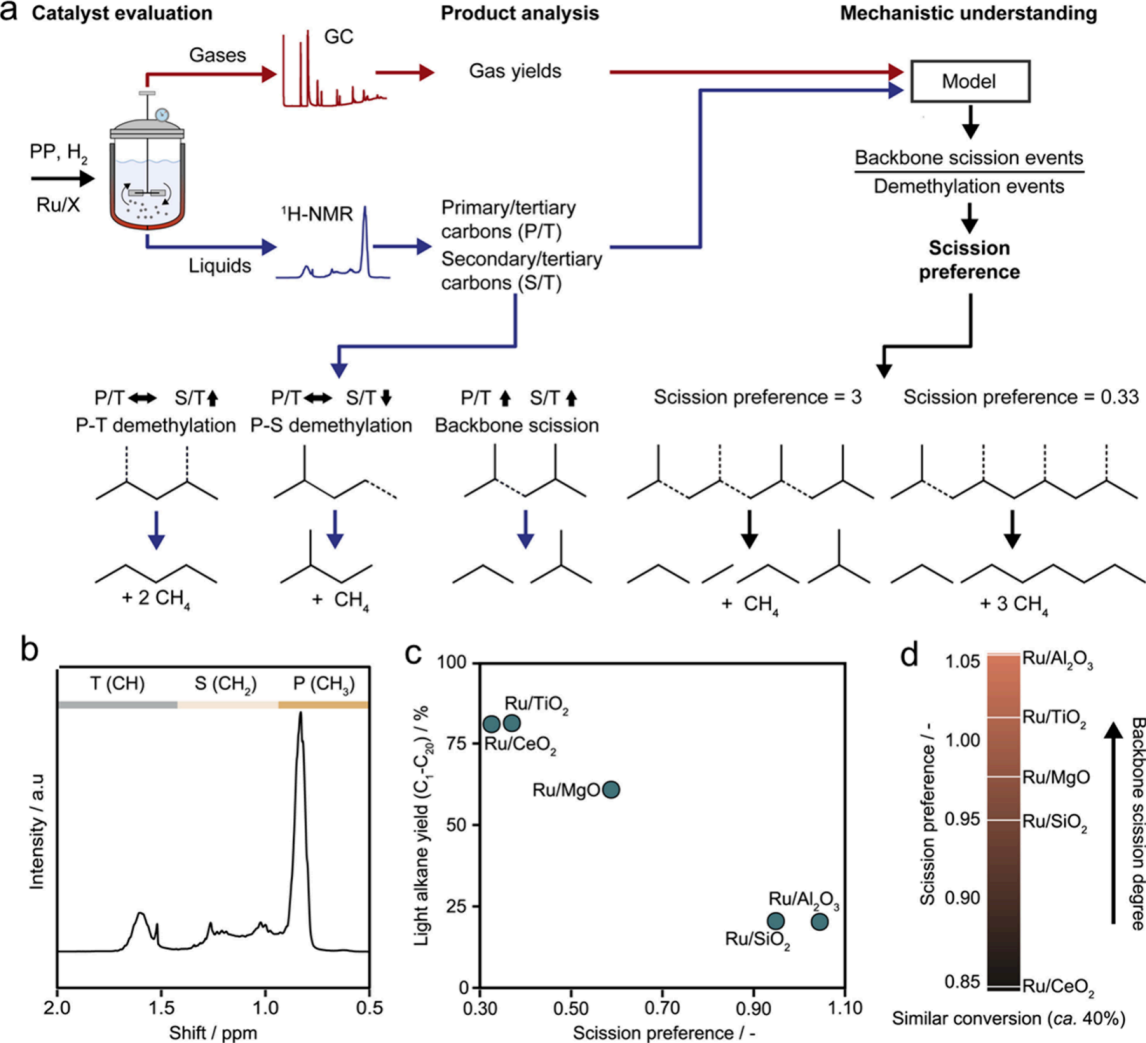
(a) Workflow to evaluate scission preferences in catalytic PP hydrogenolysis. Bottom left: Relationship between the ratios of different level carbon atoms and cleavage mechanisms. Bottom right: scission preference for demethylation vs. backbone scission. P: primary, S: secondary, T: tertiary. (b) ^1^H NMR spectrum of a depolymerization product mixture obtained with Ru/TiO_2_ (5 wt% Ru). (c) Dependence of the light alkane yield on scission preference for various Ru-based catalysts. (d) Comparison of scission preferences for various Ru-based catalysts (C_n>45_ was classified as unreacted solid residue). Reaction conditions: 240 °C, 20 bar H_2_, 4 h. Adapted with permission from ref (115). Copyright 2023, Elsevier.

Despite much work on PO hydrogenolysis, comparisons between catalysts have been limited mostly to relative crude assessments of polymer conversion and product selectivity.^114^ A standardized metric for describing selectivity in C–C scission was proposed to remedy this problem.^115^ During PP hydrogenolysis, demethylation leads to undesired methane, while backbone scission yields more valuable hydrocarbon chains. Backbone scission requires cleavage of C(2°)–C(3°) bonds ( S–T scission), leading to progressive backbone shortening. Backbone demethylation, including cleavage of C(1°)–C(3°) bonds (P–T demethylation) and C(1°)–C(2°) bonds (P–S demethylation), results in methane formation without changing the chain length of the backbone. The scission preference (SP) is defined as the ratio between the rates of backbone scission and backbone demethylation. The assessment protocol is shown in Figure 9.

This approach was used in a study of PP hydrogenolysis (*M*_w_ = 12,000 g/mol, *Đ* unspecified), involving a series of Ru catalysts (5 wt%, dispersed on: TiO_2_, α-Al_2_O_3_, CeO_2_, MgO, or SiO_2_) at 240 °C under 20 bar H_2_. After 4 h, Ru/TiO_2_ and Ru/CeO_2_ gave complete conversion and similar product distributions (methane > C_2+_ gases > liquids), while Ru/MgO, Ru/SiO_2_, and Ru/α-Al_2_O_3_ all gave lower conversions, and a different product distribution (methane > liquids > C_2+_ gases).^115^ Demethylation was inferred to be favored at high PP conversion, and light alkane yields (C_1_–C_20_) were inversely correlated with SP (Figure 9c). SP values, compared at constant (40 %) conversion, were lower for Ru/CeO_2_ (0.30) and Ru/TiO_2_ (0.38) than for Ru/SiO_2_ (0.95) and Ru/Al_2_O_3_ (1.06), showing the greater preference of the first two catalysts for demethylation (Figure 9d). Ru/α-Al_2_O_3_ is the most selective catalyst, while Ru/CeO_2_ is the least. The decrease in SP with reaction time reflects an increasing propensity for demethylation, possibly because the activation barrier for backbone cleavage is higher for shorter chains. Increased basicity appears to favor low SP values, suggesting that selective catalysts have weakly basic supports. The SP approach is currently limited to non-acidic catalysts, since the model assumes no skeletal rearrangement of the polymer backbone (such as occurs with metal–acid bifunctional catalysts).^69^

### 2.3 Hydrogenolysis Catalysts

#### 2.3.1 Ru-Based Catalysts

Ru is by far the most widely investigated active metal in PO hydrogenolysis, where it is usually deployed in the form of supported nanoparticles. Various oxide supports have been explored, including TiO_2_,^111,113,116^ CeO_2_,^106,110,117−120^ ZrO_2_,^103,121,122^ SiO_2_,^123,124^ and carbon.^46,125−128^ Typical conditions are reaction temperatures of 200 to 250 °C and H_2_ pressures from 20 to 60 bar. The target hydrocarbons are generally liquids in the range C_5_-C_45_, which can be used as fuels, lubricants, waxes, and solvents with minimal separation.

The hydrogenolysis activity of Ru/C was compared to a series of transition metal catalysts in the reaction of a PE model compound, *n*-octadecane (*n*C_18_).^46^ After 14 h at 250 °C under 50 bar H_2_, Pt/γ-Al_2_O_3_ (1 wt% Pt), unsupported NiO, and Ni/C (5 wt% Ni) showed little activity, while unsupported Co_3_O_4_ and Rh/C (5 wt% Rh) showed moderate activity (*n*C_18_ conversion < 46 mol%). However, Ru/C (5 wt% Ru) achieved full conversion of *n*C_18_ at 250 °C. Even at 200 °C, conversion was still 92 mol%. The *n*-alkane product distribution evolved with time, from C_8_–C_17_ after 2 h to C_1_–C_7_ after 16 h. The shift suggests that both terminal and internal C–C bond cleavage was occurring. A similar conclusion emerged from a study of the hydrogenolysis of iPP (*M*_w_ = 250,000 g/mol, *M*_n_ = 67,000 g/mol) catalyzed by a series of transition metals (Pd, Rh, Ir, Ni, Pt, Ru), all supported on TiO_2_, at 250 °C under 30 bar H_2_. Ru/TiO_2_ gave >90 wt% conversion while all other metal/TiO_2_ catalysts showed <10 wt% conversion.^113^

**Figure 10:**
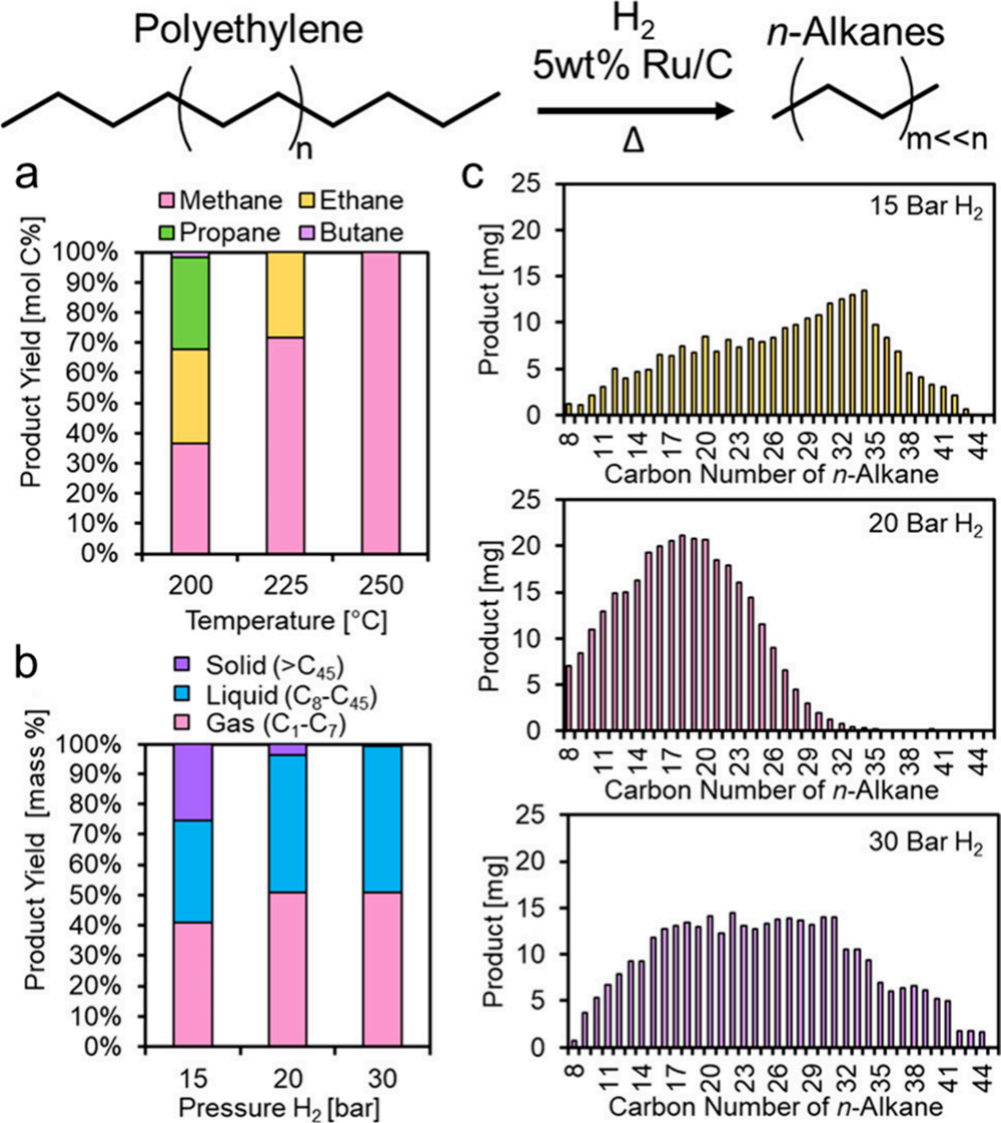
Effect on yields in PE hydrogenolysis of (a) temperature; and (b) *P*_H2_. Reaction conditions: 16 h, 30 bar H_2_, 100 mg PE (*M*_w_ = 4,000 g/mol, *M*_n_ = 1,700 g/mol), 25 mg Ru/C (5 wt % Ru). (c) Dependence of the carbon number distribution in liquid products on *P*_H2_. Reaction conditions: 200 °C, 16 h, 700 mg PE, 25 mg Ru/C. Reprinted with permission from ref (46). Copyright 2021, American Chemical Society.

The effects of temperature and *P*_H2_ on the hydrogenolysis of a low molecular weight PE catalyzed by Ru/C are shown in Figure 10.^46^ After 16 h at 200 °C and a mass ratio PE:catalyst = 4, PE was converted exclusively to gases (Figure 10a). Upon increasing the mass ratio to 28, the main products were liquids (approx. 50 wt%). Carbon number analysis showed the majority to be C_8_-C_30_ range alkanes for *P*_H2_ = 20 bar (Figure 10c). Increasing *P*_H2_ to 30 bar inhibited C–C bond scission (Figure 10b). Although the Ru nanoparticle size increased slightly from 1.6 nm in the fresh catalyst to 2.1 nm in the used catalyst, the gas and liquid product distributions were virtually the same in three consecutive hydrogenolysis reactions. PEs with different molecular weights, branching frequencies, and additives were tested under the same conditions. In general, only gases and liquid products (C_8_–C_25_) were observed.

The same Ru/C catalyst was used to convert iPP (*M*_w_ = 340,000 g/mol, *M*_n_ = 97,000 g/mol).^128^ After 8 h at 250 °C under 40 bar H_2_, the reaction of iPP (700 mg) catalyzed by Ru/C (50 mg, 5 wt% Ru) gave a maximum yield of liquid products (40 wt%), as a mixture of linear and branched alkanes centered at C_12_–C_15_, with the gas products being principally methane. Here the optimum *P*_H2_ was different, possibly due to altered interactions between the polymer and the catalyst and/or changes in the behavior of molten iPP relative to PE. Under similar conditions, hydrogenolysis of iPP (*M*_w_ = 250,000 g/mol, *M*_n_ = 67,000 g/mol) catalyzed by Ru/TiO_2_ (5 wt% Ru) gave liquid products in 66 wt% yield at 94 wt% iPP conversion after 8 h.^113^

Ru/CeO_2_ was reported to catalyze the hydrogenolysis of POs, including used plastics, to liquid fuels (C_5_–C_21_) and waxes (C_22_–C_45_).^110^ Ru/CeO_2_ (100 mg, 5 wt% Ru) converted 76 wt% of a low molecular weight PE (*M*_w_ = 1,700 g/mol, *M*_n_ = 4,000 g/mol) in 5 h in a autoclave at 240 °C under 60 bar H_2_, while other M/CeO_2_ catalysts (M = Ir, Rh, Pt, Pd, Cu, Co, Ni) with the same metal content (5 wt%) gave no essentially conversion. Structural characterization confirmed the presence of small nanoparticles (<1.5 nm, except for 6 nm in the case of Pd/CeO_2_) in the metallic state for all catalysts, ruling out possible effects of variable metal oxidation state and nanoparticle size on activity.

**Figure 11:**
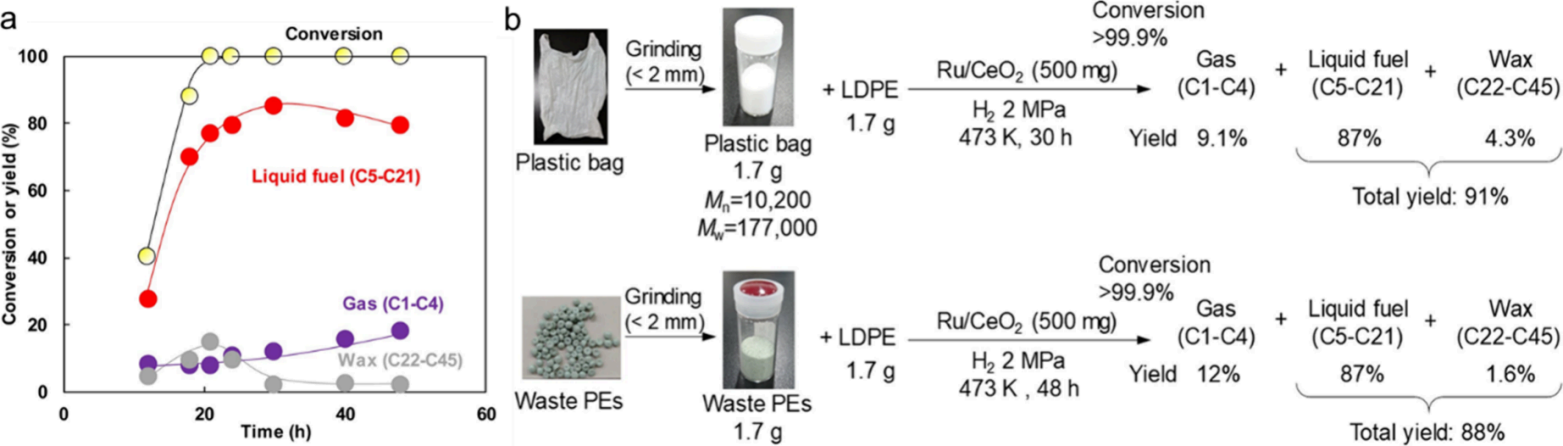
(a) Time-dependence of PE conversion and product yields in hydrogenolysis catalyzed by Ru/CeO_2_. Reaction conditions: 500 mg Ru/CeO_2_ (5 wt% Ru), 3.4 g PE (*M*_n_ = 1,700 g/mol, *M*_w_ = 4,000 g/mol), 20 bar H_2_, 200 °C. (b) Hydrogenolysis of post-consumer plastics catalyzed by Ru/CeO_2_. Adapted with permission from ref (110). Copyright 2021, Elsevier.

The time-dependence of hydrogenolysis is shown for the optimized reaction conditions (20 bar H_2_, 200 °C, 500 mg Ru/CeO_2_, 3.4 g PE) in Figure 11a. PE was fully converted in 20 h to linear alkanes. The maximum yields of waxes (C_22_–C_45_) and liquid fuels (C_5_–C_21_) were achieved at 21 and 30 h, respectively. The highest combined yield of waxes (15 wt%) and liquid fuels (77 wt%) was obtained after 21 h. When either a commercial plastic bag (PE, *M*_n_ = 10,000 g/mol, *M*_w_ = 177,000 g/mol) or waste PE was mixed with the PE, similar combined yields (ca. 90 %) of waxes and liquid fuels were obtained (Figure 11b).^110^

The hydrogenolysis of iPP (*M*_w_ = 250,000 g/mol, *M*_n_ = 67,000 g/mol) and of PE (*M*_w_ = 4,000 g/mol, *M*_n_ = 1,300 g/mol) was examined with various metal catalysts.^127^ The carbon balance reported for all reactions (unfortunately, not always done in hydrogenolysis studies) exceeded 80 wt% in most experiments. Under typical reaction conditions (250 °C, 30 bar H_2_, 18 h, 1 g PO, 100 mg catalyst), Ru/C was more effective than Rh/C, Pt/C, Pd/C, Ir/C, or Ni/C (all containing 5 wt% metal, as approx. 2.5 nm nanoparticles). The Ru-based catalyst converted iPP and PE completely to gases. Rh/C was less active than Ru/C and gave moderate conversion (30 % for iPP and 50 % for PE), while all other catalysts showed very low activity, with solid residues accounting for >99 % iPP and >93 % PE. The observed activity order matches that described above (Ru > Rh ≫ other metals).^46^ Hydrogenolysis studies are rare for Rh-based catalysts compared to Pt-based catalysts,^129^ perhaps because of the widespread use of Pt-based hydrocracking catalysts in the petrochemical industry. Control experiments with PVC showed a strong detrimental effect of chloride on activity and a change in regioselectivity (to CH_4_ and branched alkanes), indicating a need to remove Cl from plastic waste and/or to develop more Cl-tolerant catalysts.^127^

**Figure 12:**
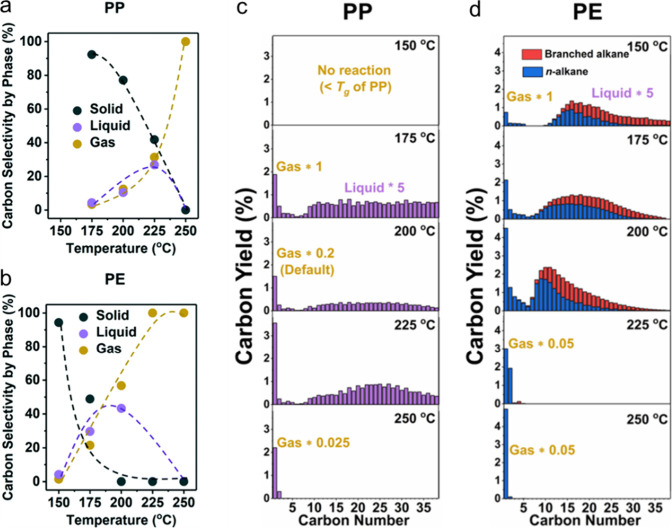
Temperature dependence of the hydrogenolysis of iPP (*M*_w_ = 250,000 g/mol, *M*_n_ = 67,000 g/mol) and PE (*M*_w_ = 4,000 g/mol, *M*_n_ = 1,300 g/mol) catalyzed by Ru/C: (a, b) selectivity for various product classes; and (c, d) carbon number distributions in the range C_1_–C_38_ (gas product amounts are multiplied by 0.2, except where noted). Reaction conditions: 30 bar H_2_, 18 h, 1 g iPP or PE, 100 mg Ru/C (5 wt% Ru). Adapted with permission from ref (127). Copyright 2022, Royal Society of Chemistry.

The onset of PO hydrogenolysis catalyzed by Ru/C occurred at 150 °C for PE and 175 °C for iPP,^127^ consistent with the difference in their melting temperatures. Maximum yields of liquid alkanes were generated at 200 and 225 °C for PE and iPP, respectively (Figure 12a, b). At these temperatures, mixtures of liquid and gas alkanes were obtained. Over time, the liquid and gas yields increased monotonically, up to 20 h for both polyolefins. The carbon number distribution in the liquid products was roughly constant prior to 100 % solid conversion, then the liquid yield started to decrease. This behavior suggests preferential adsorption and subsequent cleavage of chains longer than the liquid alkane products, due to the size dependence of alkane adsorption energies which inhibits secondary hydrogenolysis of intermediate-length products (C_<45_). A significant amount of CH_4_ was generated by cleavage of methyl branches (Figure 12c). This is undesirable in iPP hydrogenolysis, since high quality fuels require branched alkanes. Still, considering the C_10_ products as an example, branched alkanes constituted 50 and 100 % of the C_10_ isomers derived from PE and iPP, respectively. The lower fraction of branched products from PE was attributed to a lower rate of isomerization, compared to C–C bond cleavage. Selectivities to CH_4_ and branched alkanes were constant prior to complete solid conversion, consistent with the idea that intermediate-length hydrogenolysis products cannot readily access the metal surface when long polymer chains are present.

At 225 °C, iPP conversion increased monotonically with H_2_ pressure from 5 to 60 bar, then started to decline above 60 bar.^127^ At 175 °C and 45 bar H_2_, PE conversion was a maximum. The H_2_ effect was explained as competitive adsorption of H_2_ and alkanes, with branched chains adsorbing less strongly than linear chains, and higher H_2_ pressures being required at higher temperatures to reach the same H coverage. More importantly, higher H_2_ pressures caused the CH_4_ selectivity to decrease monotonically for both POs, consistent with the mechanism discussed in section 2.1.2.^91^ C–C bond scission in adsorbed hydrocarbons requires adjacent unoccupied metal sites where H atoms can adsorb. Terminal C–C bond cleavage is therefore inhibited at higher H_2_ pressures since the dehydrogenation of methyl groups is hindered more than dehydrogenation of less H-rich sites at higher H* coverages.

For iPP, cleavage can occur between either C (3°)–C(1°) or C(3°)–C(2°) bonds. The former are more abundant and lead to CH_4_ formation at lower H_2_ pressures, while the latter are favored by higher H_2_ pressures because they require the removal of fewer H atoms to achieve full dehydrogenation of the adsorbed hydrocarbons. For PE, cleavage of C(2°)–C(2°) bonds requires more dehydrogenation than cleavage of C(3°)–C(2°) bonds, the latter not being favored on surfaces with high H* coverages. Therefore, higher H_2_ pressures favor detachment of methyl branches, resulting in more linear alkane products. At 175 °C and 82 bar H_2_, PE gave a liquid yield of 57 wt% with a CH_4_ selectivity of just 3 wt%, while iPP gave a liquid yield of 47 wt% with a CH_4_ selectivity of 30 wt% under the same conditions. Overall, low temperatures and high H_2_ pressures maximized the yield of liquid products and minimized the methane yield from Ru/C-catalyzed PO reactions.

In summary, Ru-based catalysts show higher activity in PO hydrogenolysis relative to other transition metal catalysts, but controlling the product distribution and inhibiting the formation of low value alkanes, particularly methane, remain as challenges.

#### 2.3.2 Pt-Based Catalysts

**Figure 13:**
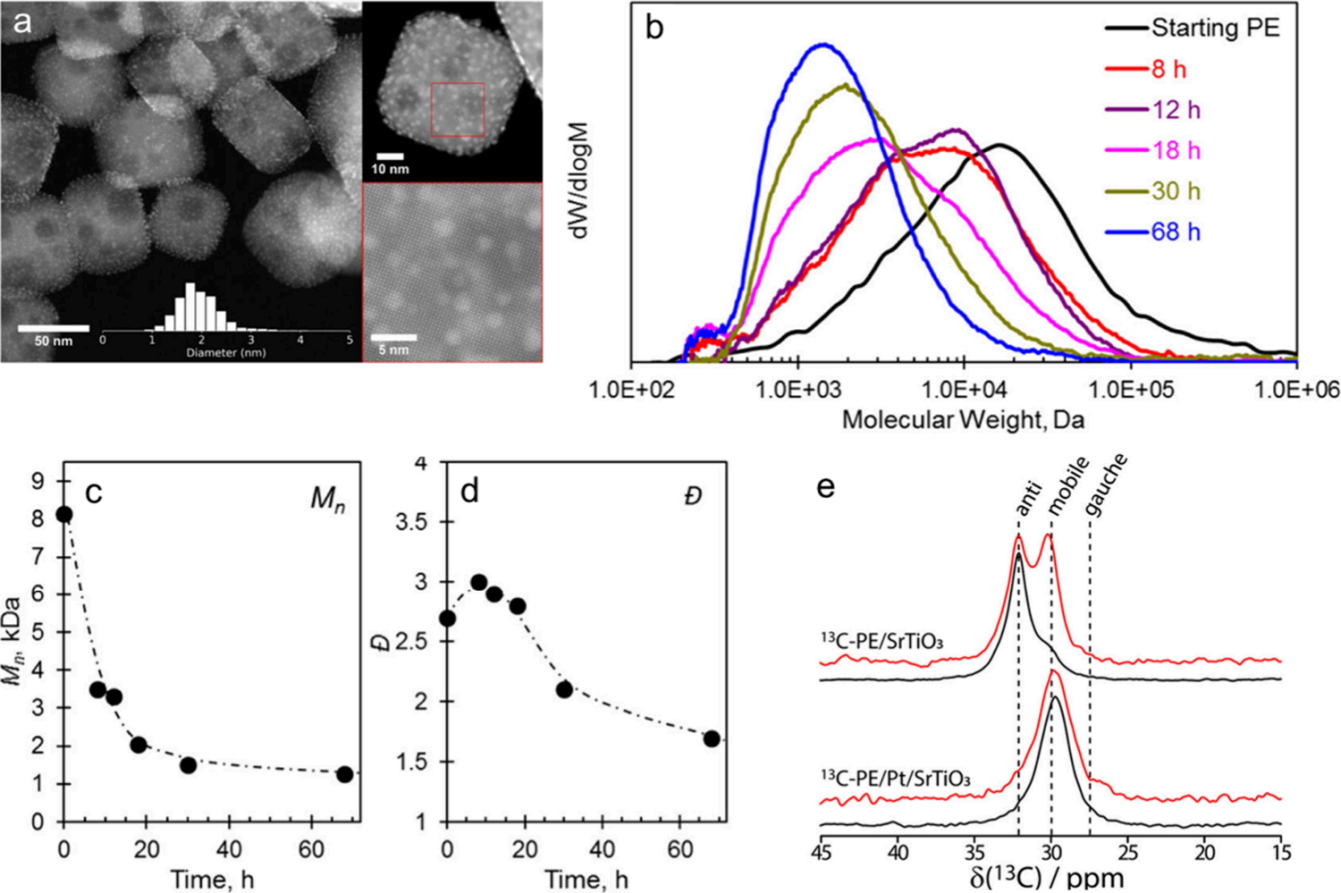
(a) Electron micrographs of 5c-Pt/SrTiO_3_ (5 wt% Pt), including the Pt nanoparticle size distribution. Time-dependent analyses of products from catalytic PE hydrogenolysis: (b) molecular weight distribution; (c) number-average molecular weight (*M*_n_); and (d) dispersity (*Đ*). Reaction conditions: 12 bar H_2_, 300 °C, PE (3 g, *M*_n_ = 8,150 g/mol, *Đ=* 2.7), 5c-Pt/SrTiO_3_ (5 wt% Pt, 160 mg). (e) Characterization of ^13^C-enriched PE adsorbed on SrTiO_3_ and Pt/SrTiO_3_ by ^13^C MAS NMR (red) and CP-MAS NMR (black). Adapted with permission from ref (54). Copyright 2019, American Chemical Society.

Pt-catalyzed hydrogenolysis of POs usually requires 10–20 bar H_2_ and 250 to 300 °C. Reported supports for Pt include SiO_2_,^130−132^ SrTiO_3_,^54,112,133,134^ and activated carbon.^135^ Pt/SrTiO_3_ was used in an early example of converting PE to value-added products in the presence of H_2_. Five cycles of atomic layer deposition (ALD, using MeCpPtMe_3_ as the Pt source) dispersed on SrTiO_3_ nanocuboids (average size: 65 nm, exposing (100) facets) gave a catalyst called 5c-Pt/SrTiO_3_ (5 wt% Pt), with Pt nanoparticles of ca. 2 nm average size (Figure 13a).^54^ The close lattice match between SrTiO_3_(100) and *fcc*-Pt provides interfacial epitaxial stabilization.^136,137^ High-throughput screening of the hydrogenolysis of PE (50 mg, *M*_n_ = 8,150 g/mol, *M*_w_ = 22,150 g/mol, *Đ* = 2.7) catalyzed by 5c-Pt/SrTiO_3_ (22 mg, containing 1.1 mg Pt) in a 96 h test identified the optimal reaction conditions as 12 bar H_2_ and 300 °C. The PE was converted to liquid products (42 wt%, *M*_n_ = 590 g/mol, *M*_w_ = 625 g/mol, *Đ* = 1.1) consisting of a mixture of linear alkanes (10 methyl branches/1000 C). The narrow dispersity could make them useful as lubricants and waxes. Similar products (*M*_n_ = 590–990 g/mol) were achieved starting from PEs of different molecular weights (*M*_n_ = 8,150–15,800 g/mol) as well as a post-consumer PE bag (*M*_n_ = 33,000 g/mol, *Đ* = 3.5). The hydrogenolysis of iPP (*M*_n_ = 6,000 g/mol, *Đ* = 2.2) catalyzed by Pt/SrTiO_3_ (10 wt% Pt) gave liquid products (*M*_n_ = 250 g/mol, *Đ* = 1.4) with broader dispersity.^108^

*M*_n_ and *Đ* for the hydrogenolysis products shifted to lower values as the reaction progressed (Figure 13b–d), showing that heavier chains undergo hydrogenolysis preferentially relative to lighter chains.^54^ The interactions between the hydrocarbon chains and the surface were studied using solid-state ^13^C MAS NMR of a ^13^C-enriched PE (*M*_n_ = 132,000 kg/mol, *Đ =* 3.2). Three conformations of adsorbed polymer were observed: anti (δ = 32.9 ppm), gauche (δ = 27.5 ppm), and mobile (no preferred conformation, δ = 30 ppm), Figure 13e. The disappearance of the signal for the anti-conformation after Pt deposition on SrTiO_3_ suggests that Pt nanoparticles dramatically enhance PE mobility and suppress its interactions with the SrTiO_3_ surface. Theoretical modeling also predicted that hydrocarbon molecules interact more strongly with Pt(100) and Pt(111) surfaces than with the TiO_2_-terminated surface of SrTiO_3_, by aligning their C–C bonds in an all-anti conformation parallel to the metal surface. Therefore, the hydrogenolysis activity of Pt/SrTiO_3_ was attributed to favorable adsorption of long PE chains on Pt sites rather than on SrTiO_3_. Comparison of Pt/SrTiO_3_ and a commercial Pt/γ-Al_2_O_3_ (1 wt% Pt, 1.6 nm nanoparticles) revealed that the SrTiO_3_ support also influences the outcome of the reaction, by suppressing the formation of light hydrocarbons (C_1_–C_8_) and producing liquids with a narrower dispersity (*Đ =* 2.4 vs. 6.6) even when the product *M*_n_ values are similar (1,500 g/mol).^54^

The lubricant properties of the liquid products derived from the hydrogenolysis of HDPE (*M*_w_ = 38,000 g/mol, *M*_n_ = 5,400 g/mol) catalyzed by Pt/SrTiO_3_ were investigated and compared to those of synthetic base oils (*aka* polyalphaolefins, or PAOs) and petroleum-based lubricants (Group III mineral oils). Better tribological performance (friction coefficient, wear scar and viscosity) was observed for the plastic-derived lubricants. Specifically, the addition of 20–30 wt% plastic-derived lubricants to PAOs reduced friction by approx. 9 % at 100 °C.^133^ Converting waste plastics to products such as high-performance lubricants, with higher value than fuels, was suggested as a strategy to offset the feedstock cost. A conceptual design for a facility to convert PE to lubricants was presented, using extrapolated lab-scale performance data (60–90 % lubricant yields). In the case of the highest yield, the production cost for plastic-derived lubricants was estimated to be $1.8/gal, compared to a market price above $6 per gal for Group III grade and PAO lubricants. The major expense was the cost of feedstock (waste HDPE). Replacing methane-derived H_2_ with green H_2_ was projected to increase the lubricant production cost by up to 13%.^134^

**Figure 14:**
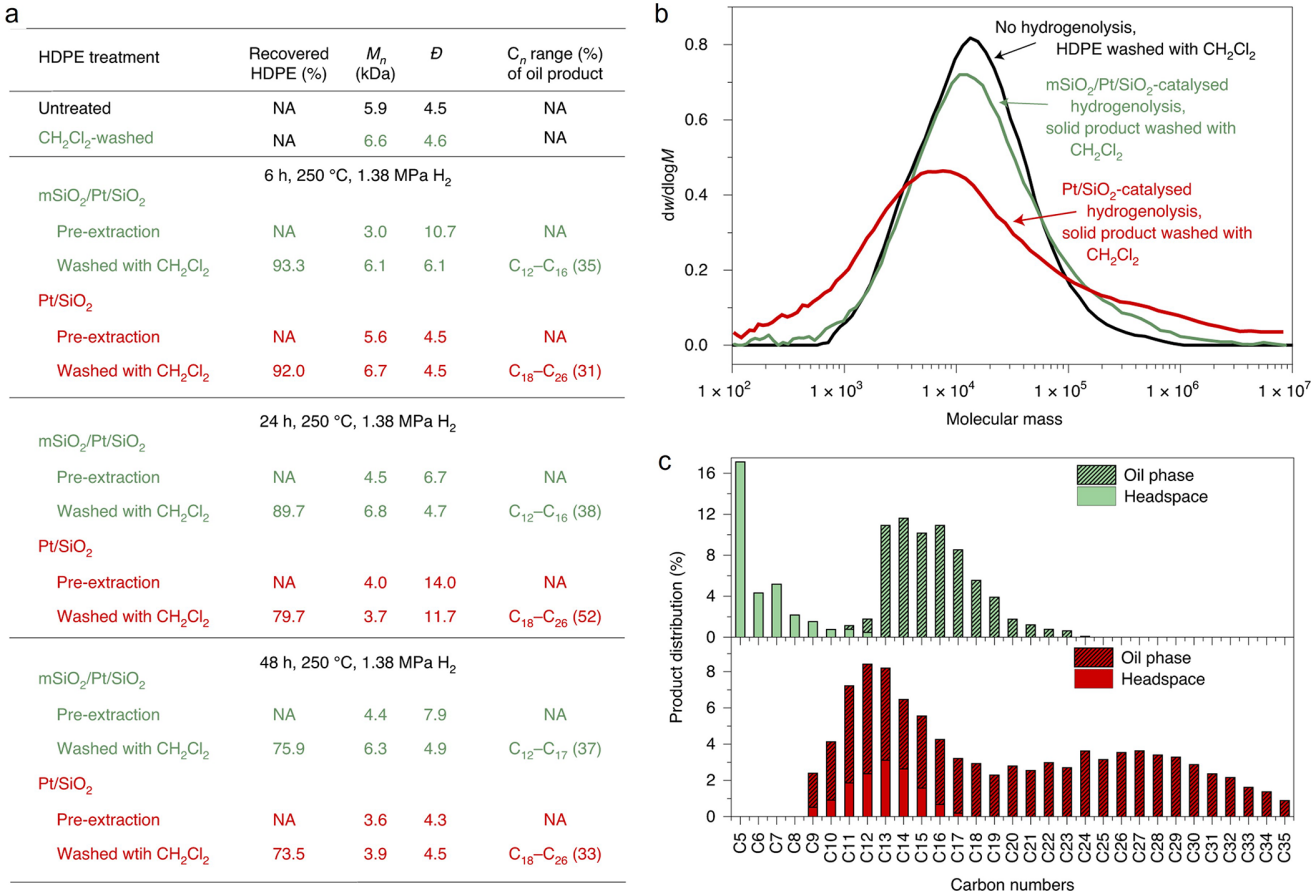
Comparison of hydrogenolysis performance for mSiO_2_/Pt/SiO_2_ and Pt/SiO_2_: (a) product distributions; (b) GPC analysis of starting HDPE (black) and insoluble residues remaining after 24 h; and (c) carbon number distributions of light hydrocarbons after 6 h. Reaction conditions: 250 °C, 14 bar H_2_. Reproduced with permission from ref (130). Copyright 2020, Springer Nature.

Diesel fuel and lubricant-range alkanes were also made by hydrogenolysis of PE-based single-use shopping bags using Pt nanoparticles (diameter 3.2 ± 0.5 nm), located at the bottom of silica mesopores.^130^ The nanoparticles were deposited onto a nonporous silica core (diameter 127 nm), then covered by a shell of mesoporous silica (110 nm-thick, 2.4 nm diameter pores directed radially).^130^ Hydrogenolysis of HDPE (*M*_n_ = 5,900 g/mol, *Đ =* 4.5) catalyzed by mSiO_2_/Pt/SiO_2_ (1 mg Pt per 100 g HDPE) was performed at 250 °C under 13.8 bar H_2_. Pt nanoparticles supported on the nonporous SiO_2_ without the mSiO_2_ shell (Pt/SiO_2_) served as a control to examine the effect of the mesopores. The performance of the two catalysts was distinguished by the amount of the “unreacted polymer residue” (defined as the solid remaining after washing with CH_2_Cl_2_ to remove C_<40_), Figure 14a. For mSiO_2_/Pt/SiO_2_, the unreacted polymer residue remaining after 24 h had the same properties (*M*_n_ and *Đ*) as the starting polymer, while Pt/SiO_2_ gave a material with a lower molecular weight at a similar polymer conversion (Figure 14b). The carbon number distributions in the liquid products at similar polymer conversions (ca. 7 %) were also quite different for the two catalysts, Figure 14c. For mSiO_2_/Pt/SiO_2_, the product distribution was centered at C_12_–C_16_ (diesel-range), accounting for 40 wt% of total products. At full conversion of HDPE, the yield of oil products (C_9_–C_15_) was 50 wt%.

Inspired by the high hydrogenolysis activity of Ru/C,^46,113^ Pt supported on an activated carbon (AC1, B.E.T. surface area 3,000 m^2^/g) was explored in the hydrogenolysis of PP (*M*_w_ = 12,000 g/mol, *Đ* and tacticity unspecified).^135^ Pt/AC1 was prepared by incipient wetness impregnation of pre-synthesized Pt nanoparticles,^138^ to give a material with a Pt loading of 5 wt% and an average Pt nanoparticle size of 2 nm after removal of the capping agent. At 300 °C under 15 bar H_2_ and with a catalyst/PP ratio of 1:10, the selectivity to liquid products (C_5_–C_45_) was 100 % after 24 h. Among the liquid products, motor oil-range hydrocarbons (C_21_–C_45_), diesel-range hydrocarbons (C_12_–C_20_), and gasoline-range hydrocarbons (C_5_–C_12_) contributed 77, 20 and 3 wt%, respectively. Lighter hydrocarbons were undetectable, even at full conversion.

#### 2.3.3 Catalysts Based on Non-Noble Transition Metals

Although noble metals (Ru, Rh, Pt) are currently the most active metal catalysts known for PO hydrogenolysis, Earth-abundant metals have also been explored, often at higher metal loadings. We note that metal cost and availability are likely to be impacted by changing demand during the energy transition,^139^ making the value of justifications based solely on these factors difficult to evaluate. The performance of some non-noble transition metal catalysts in PO hydrogenolysis is summarized below.

Many older investigations of small alkane hydrogenolysis catalyzed by Ni have generated knowledge relevant to PO hydrogenolysis. A kinetic study of ethane hydrogenolysis catalyzed by Ni/SiO_2_ showed that the rate depends on the degree of H surface coverage, because reversible H_2_ adsorption competes with the irreversible adsorption of ethane.^140^ Hydrogenolysis of *n*-hexane and its isomers, catalyzed by Ni/SiO_2_, led to successive demethylation with only a small extent of internal bond scission, due to the favorable formation of 1,2-diadsorbed species arising from primary alkane adsorption.^105^

**Figure 15:**
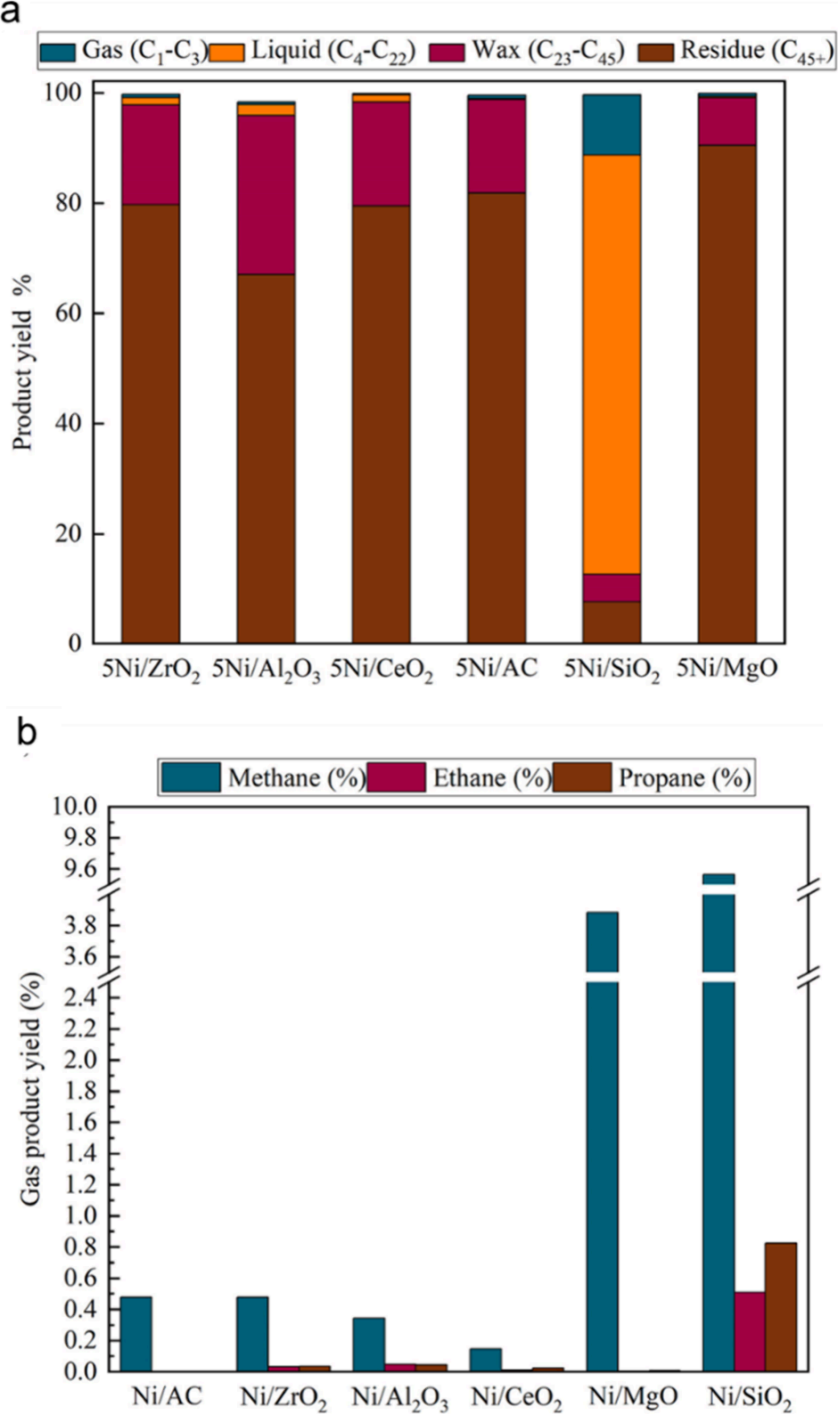
Comparison of PE hydrogenolysis by various supported Ni catalysts: (a) Product yields for various broad product categories; and (b) compositional analysis of the light gases. Reaction conditions: 1.5 g PE (*M*_w_ = ∼4,000 g/mol, *M*_n_ = ∼1,700 g/mol), 0.3 g catalyst (5 wt% Ni), 280 °C, 30 bar H_2_, 4 h. Reproduced with permission from ref (141). Copyright 2022, Elsevier.

Ni nanoparticles dispersed on various supports (ZrO_2_, Al_2_O_3_, CeO_2_, activated carbon, SiO_2_, MgO, all with 5 wt% Ni) were synthesized by a deposition-precipitation method. Ni/SiO_2_ showed the best activity in hydrogenolysis of PE (*M*_w_ = ∼4000 g/mol, *M*_n_ = ∼1700 g/mol) at 280 °C under 30 bar H_2_, Figure 15. After 4 h, this catalyst gave the highest yield of liquid (C_4_-C_22_) products (75 wt%) and the least polymer residue. Among the other catalysts, the most efficient gave only a 30 wt% yield of waxes (C_23_-C_45_).^141^ For Ni/SiO_2_, the liquid yield reached a maximum of 81 wt% with an average carbon number of C_15_ after 8 h. The methane yields increased from 10 to 36 wt% between reaction times of 4 and 12 h.

**Figure 16:**
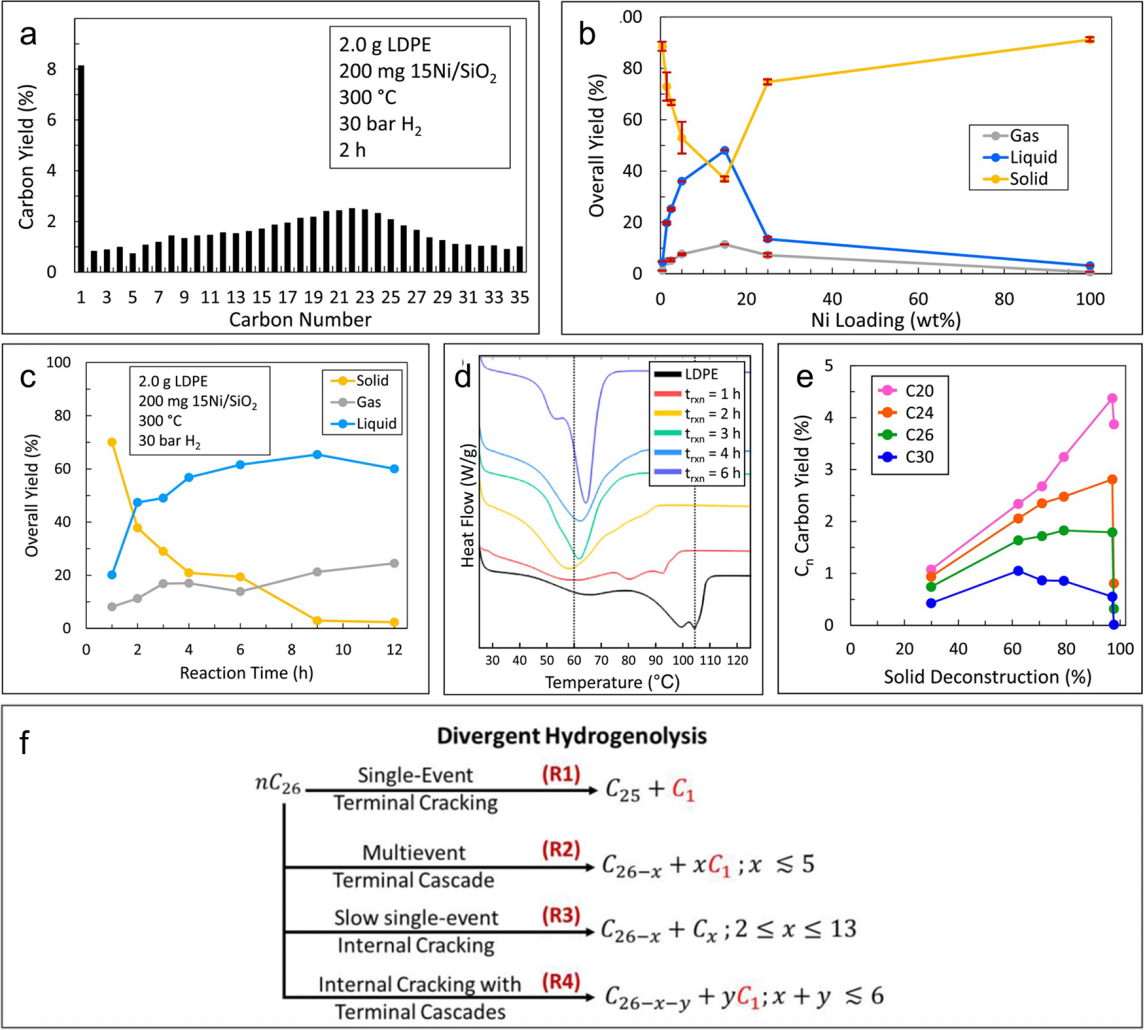
PE hydrogenolysis catalyzed by Ni/SiO_2_: (a) distribution of light alkanes, for 15 wt% Ni; and (b) broad categories of product yields at 2 h, as a function of Ni loading. Time dependence observed with 15 wt% Ni catalyst, for: (c) yields in broad product categories; and (d) DSC analyses of solid residues. (e) Independence of alkane selectivities until high PE conversion. Reaction conditions: 2.0 g PE (*M*_w_ ∼ 4,000 g/mol, *Đ* unspecified), 200 mg Ni/SiO_2_, 300 °C, 30 bar H_2_. (f) Proposed hydrogenolysis pathways. Adapted with permission from ref (90). Copyright 2022, Elsevier.

A study of the reaction catalyzed by Ni/SiO_2_ revealed the origins of product selectivity.^90^ In the hydrogenolysis of PE (*M*_w_ ∼ 4,000 g/mol, *Đ* unspecified) at 300 °C under 30 bar H_2_, the optimum liquid yield was 57 wt% (max. at C_22_) after 2 h when the catalyst had a Ni loading of 15 wt% (Figure 16a,b). The liquid yield changed little after 3 h, although the residual solid became more crystalline as it continued to convert (Figure 16c,d). The disappearance of tertiary carbons (initially, 1.5 branches per 100 CH_2_ groups) in the solid residue according to ^13^C NMR suggested that short-chain branches are the initial locations for hydrogenolysis. Competitive adsorption of residual polymer and hydrogenolysis products favored longer chains, accounting for the stable selectivities in the light alkane products (C_1_–C_35_) between 1 and 6 h. Only after the PE conversion exceeded 95 wt% were heavier products (C_20+_) transformed into lighter alkanes (C_2_–C_19_) by secondary hydrogenolysis (Figure 16e).

The catalytic performance of Ni/SiO_2_ was promising: for full deconstruction of PE, the hourly rates of production of liquid hydrocarbons, normalized by total catalyst mass, were ca. 0.7 g_PE_/g_cat_/h for Ni/SiO_2_, 0.15–0.25 g_PE_/g_cat_/h for Pt-based catalysts, and 0.5–12 g_PE_/g_cat_/h for Ru-based catalysts.^51^ In general, hydrogenolysis rates are controlled by the product of surface H and hydrocarbon coverages, but the optimal H_2_ pressure is metal-sensitive. Therefore the reaction conditions must be optimized for each metal.^46,110,113^ Similar to the Ru-based catalysts, Ni/SiO_2_ (15 wt%) showed maximum activity with 30 bar H_2_, suggesting that the metal surface is mostly covered by adsorbed hydrocarbons at lower pressures, and by adsorbed H at higher H_2_ pressures.

Kinetic modeling of the hydrogenolysis of *n*-hexacosane (*n*C_26_H_54_) catalyzed by Ni/SiO_2_ led to a proposal for different types of C–C bond cleavage events, Figure 16f. Longer chains may undergo a single terminal C–C bond cleavage (path R1) or multiple terminal C–C bond scissions (path R2). Slow internal C–C bond cleavage (path R3) can be followed by multiple terminal C–C bond scissions (path R4). By adjusting the individual rates of the four reaction pathways, the predicted product molecular weight distribution was matched with experimental results.^51^

Nickel aluminate, prepared by coprecipitation and activated by H_2_ reduction at 350 °C, was active in the hydrogenolysis of PE (*M*_w_ = 4,000 g/mol, *Đ* unspecified) at 300 °C and 30 bar H_2_.^142^ The polymer conversion was ∼45 and 100 wt% after reaction times of 2 and 10 h, respectively. The methane selectivity was <5 wt%, which represents one of the lowest methane yields achieved with a Ni-based catalyst (see section 2.4.2).

Ni/SiO_2_ (15 wt% Ni, prepared by wet impregnation) was also reported to be effective for deconstructing PS (*M*_w_ = 97,000 g/mol, *Đ* unspecified) at 300 °C under 70 bar H_2_. After 6 h, aliphatic oils were obtained with a favorable pour point (−30 °C), oxidation onset temperature (184 °C) and viscosity index (130), similar to those of high-quality lubricants (high-grade Group IV oils). Time-dependent hydrogenolysis experiments with PS and the model compound 1,2-diphenylmethane showed that hydrogenation of aromatic rings occurs 10^3^× faster than aliphatic C–C bond cleavage.

**Figure 17:**
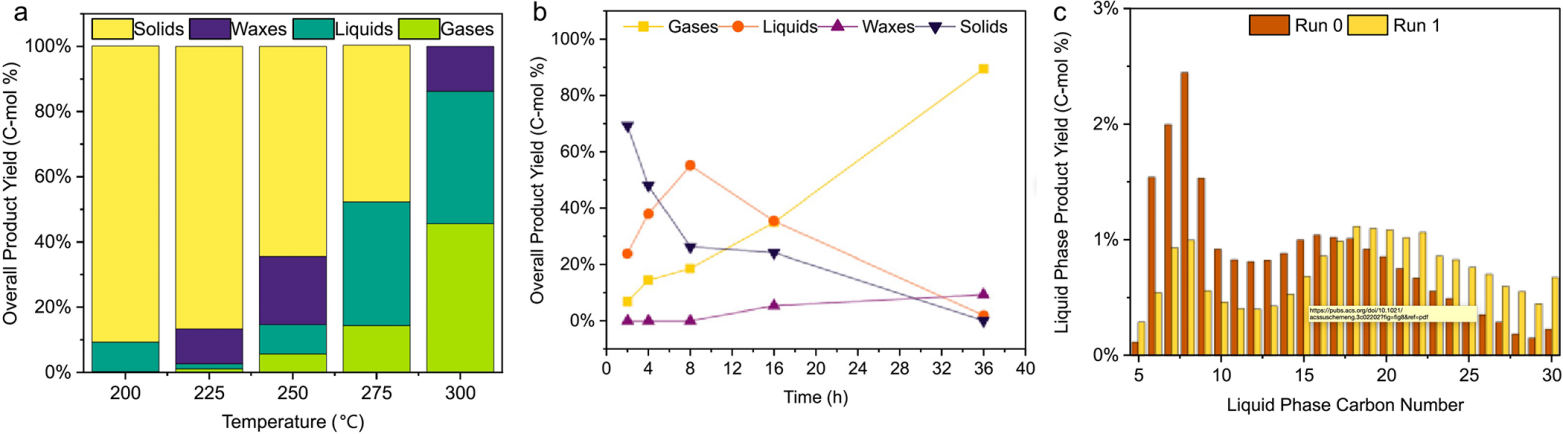
For PE hydrogenolysis catalyzed by Co/SiO_2_, the effect on product distribution of (a) temperature (all at 4 h reaction time); and (b) reaction time (all at 275 °C). (c) Comparison of distributions for liquid-phase products from fresh and reactivated catalysts, in reactions conducted at 300 °C for 4 h. The reactivated catalyst was calcined at 450 °C for 5 h in air between run 0 and run 1. Reaction conditions: 1.0 g PE (*M*_w_ = 4,000 g/mol, *Đ* unspecified), 0.10 g Co/SiO_2_ (5 wt% Co), 30 bar H_2_, 200 rpm. Adapted with permission from ref (143). Copyright 2023, American Chemical Society.

Co is another non-noble metal capable of promoting PO hydrogenolysis. Co/SiO_2_ (5 wt% Co) was used to convert PE (*M*_w_ = 4,000 g/mol, *Đ* unspecified) at temperatures from 200 to 300 °C, pressures from 20 to 40 bar H_2_, and reaction times from 2 to 36 h (Figure 17).^143^ The products consisted of gases (C_1_–C_5_ alkanes), liquids (C_5_–C_30+_ alkanes, as detected by GC), waxes (C_30+_ oligomers not detected by GC) and solid residues. Figure 17a shows that the yield of solid residue decreased from 87 % (as C-mol%) at 225 °C to zero at 300 °C. The maximum liquid selectivity was obtained at 275 °C, with an average liquid-phase carbon number of ca. 22. The full product distribution was monitored as a function of time at 275 °C (Figure 17b). The solid yield decreased monotonically while the gas yield increased. The highest yield of liquid products (55 C-mol%, ranging from C_5_ to C_30_) was obtained at 275 °C and 30 bar H_2_ after a reaction time of 8 h. The liquid selectivity was ca. 75 % during the first 8 h of reaction but declined to 2 % after 36 h. Interestingly, the average carbon number in the liquid phase did not shift, but remained between 22 and 24 over the full reaction time of 36 h. Recycling of the used Co/SiO_2_ catalyst was explored. The recovered catalyst was not active unless regenerated prior to reuse. The loss of activity was attributed to carbon deposition. After calcination at 450 °C for 5 h in air, the regenerated catalyst gave a similar product distribution, Figure 17c.

In the hydrogenolysis of small hydrocarbons, each C–C bond cleavage event can be classified as either terminal or non-terminal; for POs, subsequent C–C bond cleavage in internally-cleaved PO fragments is called sequential nonterminal cleavage, as discussed in section 2.1.1. To explain the initially constant average carbon number in the liquid product distribution, an additional mechanism was proposed.^143^ The authors described this mechanism as “tandem” nonterminal cleavage (Note that this differs from the accepted use of the term tandem, which refers to sequential, coupled catalytic reactions that occur via mechanistically distinct processes).^144^ A PO chain was suggested to adsorb on multiple, nearby metal nanoparticles where it undergoes quasi-simultaneous scission to oligomer-length (C_2_ to C_30_) segments. This type of “tandem” non-terminal cleavage was proposed to dominate during the first 4 h of reaction. However, if the nanoparticle size or interparticle distance exceeded certain threshold values, individual PO chains were proposed to adsorb at multiple adjacent sites on the same nanoparticle where they underwent “tandem” bond cleavage, producing predominantly light gases as products. The authors attempted to validate this idea by comparing the hydrogenolysis performance of unsupported Co generated by reduction of bulk Co_3_O_4_ with that of Co/SiO_2_. However, the comparison was not made at comparable PE conversions,^143^ so the conclusion was not fully convincing.

CoAl_2_O_4_ spinels were reported to convert PE (*M*_w_ = 3,000 kg/mol, *Đ* unspecified) into gasoline and jet-fuel range *n*-alkanes in a two-step cascade fixed-bed reactor. PE was delivered to the first reactor by a high-pressure solid sampler, for pyrolysis at 500 °C with a catalyst-to-feedstock (C/F) mass ratio of 2. Hydrogenolysis was performed in the second reactor at 300 °C under 2 bar H_2_.^145^ With a contact time of 20 s, the yield of C_5_–C_12_ (gasoline-range) *n*-alkanes was 86 wt%. The spinel catalysts showed higher activity than physical mixtures of γ-Al_2_O_3_ and Co_3_O_4_, as well as 3× and 5× increases in the yields of C_5_–C_12_ (gasoline range) and C_8_–C_16_ (jet-fuel range) products, respectively. Thus, Co active sites derived from the spinel facilitated alkane cleavage. The product distribution varied with the Co/Al ratio in the spinel, with low ratios (e.g., 0.5) favoring deep cracking, relatively narrow carbon number distributions (C_5_–C_10_), and higher gas yields.^145^

#### 2.3.4 Catalysts Based on Metal Oxides

**Figure 18:**
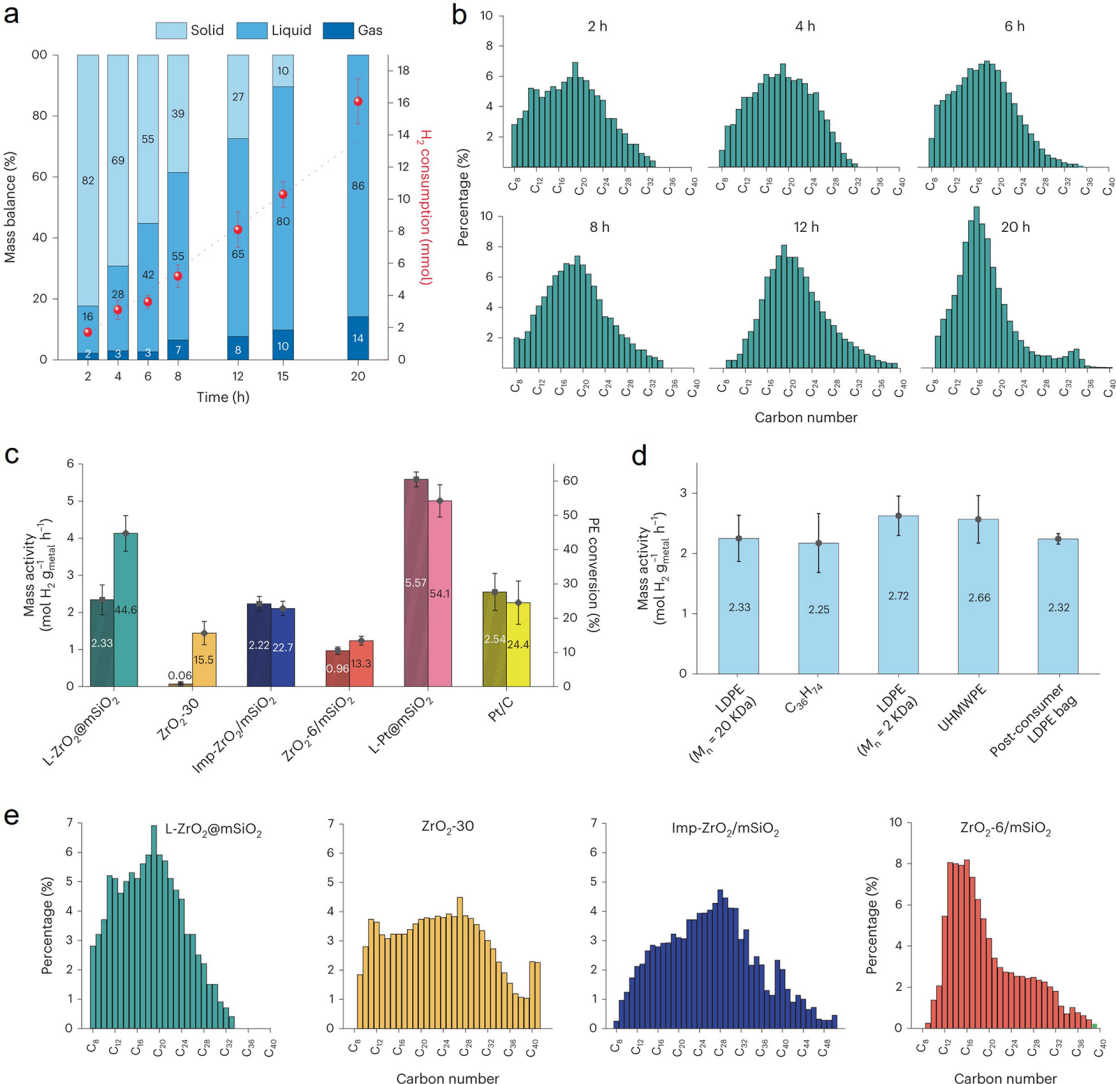
PE hydrogenolysis catalyzed by various ZrO_2_-based catalysts: (a) time-dependent mass yields for various product categories and H_2_ consumption in the reaction catalyzed by L-ZrO_2_@mSiO_2_, and (b) distribution of liquid products for the same catalyst. (c) Comparison of activities for several ZrO_2_-based catalysts in C–C bond cleavage (darker bars, derived from the rate of H_2_ consumption) and PE conversion (lighter bars) after 6 h at 300 °C. Reaction conditions: PE: *M*_n_ = 20,000 g/mol, *M*_w_ = 90,000 g/mol, *Đ* = 4.8, 300 °C. (d) Comparison of C–C bond cleavage activities (at 300 °C, averaged over 6 h) for different PEs. (e) Distribution of liquid products produced by ZrO_2_-based catalysts with various architectures, at constant H_2_ consumption. Reproduced with permission from ref (146). Copyright 2023, Springer Nature.

Metal oxides have been investigated less often as catalysts for PO hydrogenolysis. Amorphous ZrO_2_ nanoparticles of diameter (3.0 ± 0.5) nm were sandwiched between platelets of mesoporous silica of thickness 35 nm, to give L-ZrO_2_@mSiO_2_ (4.7 wt% ZrO_2_, where L indicates that ZrO_2_ is located in the SiO_2_ mesopores).^146^ The confinement provided by the mesopores, in addition to the covalent Si–O–Zr anchoring, was suggested to limit the growth and crystallization of the nanoparticles. The ability of the catalyst to effect hydrogenolysis of PE (*M*_w_ = 90,000 g/mol, *Đ* = 4.8) was studied at 300 °C under 10 bar H_2_. The time-dependent yields of gases (C_1_–C_9_), liquids (C_8_-C_50_) and polymer residue (C_>50_) are shown in Figure 18a,b. Trace metal contaminants were ruled out as catalytically active species. Within 20 h, products in the range C_8_–C_50_ showed a narrow, Gaussian-type distribution centered at C_18_, with C_9_–C_27_ representing >90 mol% of all liquid products, regardless of reaction time. After 20 h, the product distribution shifted to shorter chain lengths. L-ZrO_2_@mSiO_2_ had an activity (evaluated based on the rate of H_2_ consumption) comparable to that of Pt/C (7.1 wt% Pt), and higher than several other ZrO_2_-based catalysts, Figure 18c. The other ZrO_2_-based catalysts gave broader, flat or multimodal product distributions (Figure 18e). The rate of C–C bond cleavage was constant for PEs with different chain lengths (from C_36_ to *M*_n_ = 20,000 g/mol) and degrees of branching (Figure 18d), even though the chains likely enter the pores and access the active sites at different rates.

According to *in situ* DRIFTS, H_2_ undergoes heterolytic dissociation on the ZrO_2_ surface, generating both Zr–H and O–H bonds.^147^ Low-coordinate Zr ions present in amorphous ZrO_2_ nanoparticles were modeled with DFT calculations as Zr adatoms on the (−111) surface of monoclinic ZrO_2_. The initial reaction of a model compound, *n*-hexane, at these sites involves C–H bond activation by 1,2-addition across a Zr–O bond. The reaction is more favorable than σ-bond metathesis with evolution of H_2_ at a [ZrH] site. C–C bond cleavage via β-propyl elimination gives propene (which is hydrogenated by [ZrH]) and a [Zr-propyl] site. The latter eliminates propane by proton transfer from the proximal hydroxyl group, rather than by the σ-bond metathesis mechanism proposed for supported organozirconium catalysts (see section 2.3.5). A bifunctional mechanism (i.e., heterolytic activation of H_2_ and alkanes with formation of alkenes, followed by acid-catalyzed C=C bond cleavage via a carbenium ion intermediate) was not investigated. Nevertheless, it is intriguing that an air-stable catalyst for PO hydrogenolysis can be made directly from ZrO_2_, without the need for synthesis and grafting of a molecular organozirconium complex.^146,148,149^

Nb_2_O_5_ rich in surface oxygen vacancies was also reported to activate H_2_ by both heterolytic and homolytic cleavage, forming surface hydrides.^150^ The catalyst is effective for the hydrogenolysis of hydrocarbons, converting phenylcyclohexane (a model compound) to benzene in 46 wt% yield and methylcyclopentane in 31 wt% yield, at 98 % conversion. Polystyrene was converted (52 wt%) to arenes (benzene, cumene, ethylbenzene) in a total yield of 48 wt% in 12 h at 280 °C under 5 bar H_2_. These examples showcase the high selectivity of Nb_2_O_5_ towards the cleavage of C_sp2_-C_sp3_ bonds.

#### 2.3.5 Catalysts Based on Supported Organometallic Compounds

Early transition metal hydride complexes represent another type of PO hydrogenolysis catalyst.^149^ Usually prepared by supporting an organometallic precursor on an oxide,^151^ they operate under relatively mild conditions (T≤ 200 °C, *P*_H2_ ≤ 2 bar). The earliest report dates from the 1990s, and described the degradation of PE and PP to a range of shorter-chain saturated alkanes. The catalyst was a highly electrophilic silica-supported Zr(IV) hydride, [(≡SiO)_3_ZrH], operating at moderate temperature (<150 °C) and low *P*_H2_ (<1 atm).^149^ It was made by depositing a molecular complex, Zr(CH_2_^t^Bu)_4_, onto a dry silica surface, followed by exposure to H_2_ at 150 °C to remove the neopentyl ligands and install hydride ligands. A proposed mechanism involving β-alkyl transfer to Zr was described as the microscopic reverse of the alkene insertion step in Ziegler-Natta polymerization. This study was therefore the first to suggest that Ziegler–Natta catalysts for alkene polymerization can be repurposed to depolymerize waste plastic into alkanes in the presence of H_2_. While direct depolymerization to alkenes is not thermodynamically favorable under the reaction conditions, the use of H_2_ to saturate the C=C double bonds renders the overall reaction accessible despite the moderate temperature.

When the [ZrH] sites are supported on acidic silica-alumina instead of silica, and used to catalyze the hydrogenolysis of alkanes under 1 atm H_2_ at 150 °C, the product molecular weight distribution shifted to lower carbon numbers and consisted mainly of light alkanes after 62 h. Experiments with LDPE (*M*_n_ = 125,000 g/mol, *Đ* unspecified) and iPP (*M*_n_ = 250,000 g/mol, *Đ* unspecified) gave similar results.^149^ Other silica-supported metal hydrides also catalyzed alkane hydrogenolysis, with rates that depend on the metal ion (Ti > Zr > Hf, Ta > W).^152^

**Scheme 7:**
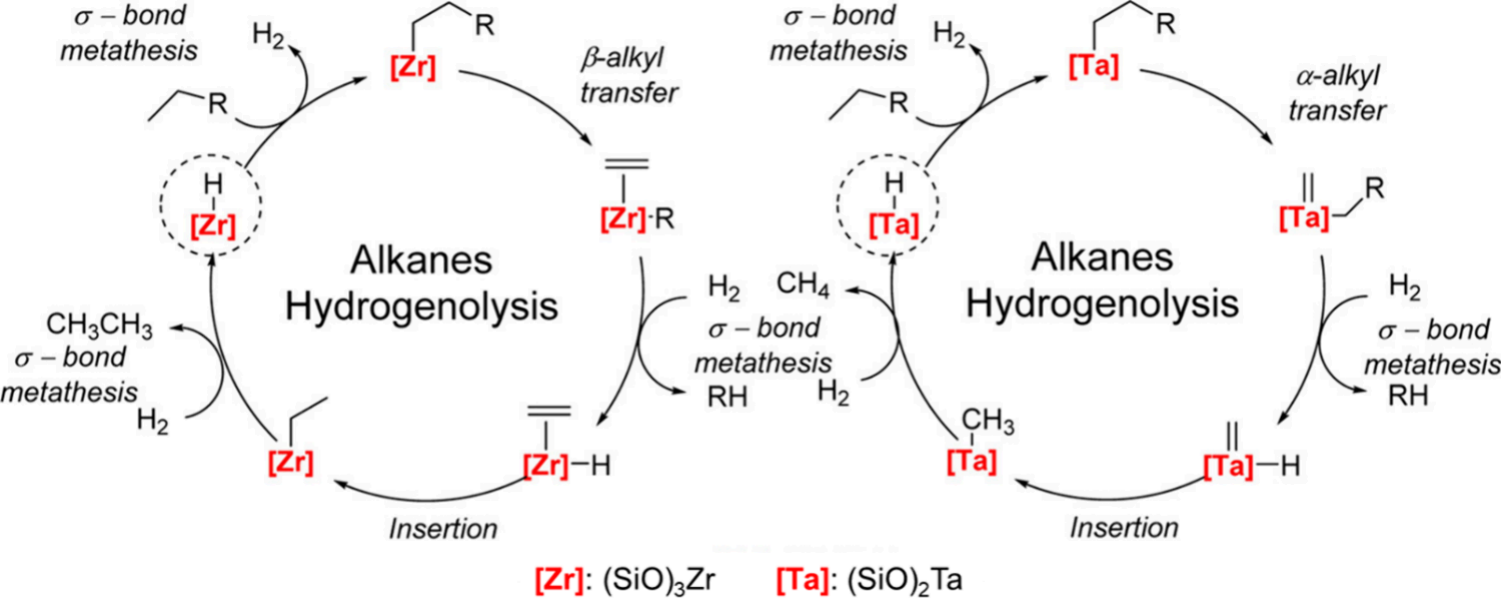
Comparison of Proposed Mechanisms for Alkane Hydrogenolysis Catalyzed by Zr(IV) Hydrides (Involving β-Alkyl Transfer, Left) and Ta(V) Hydrides (Involving α-Alkyl Transfer, Right) Reprinted with permission from ref (151). Copyright 2016, American Chemical Society.

A DFT study of the hydrogenolysis mechanism, using propane as a model compound and silica-supported [ZrH] as the catalyst, predicted the rate-limiting step to be β-methyl transfer to Zr, with an activation energy of 122 kJ/mol.^153^ Therefore, β-alkyl transfer was proposed to be the key step in C–C bond cleavage catalyzed by silica-supported group 4 metal hydrides (Ti, Zr, Hf). However, the C–C cleavage mechanism can vary depending on the metal: group V and VI metal hydrides have been suggested to perform C–C bond scission by α-alkyl transfer.^151^ The two hydrogenolysis pathways are compared in Scheme 7.

C–H bonds in both molecular and macromolecular alkanes are proposed to be activated by σ-bond metathesis, regardless of the metal ion. According to a DFT study of reactions catalyzed by [(≡SiO)_3_ZrH], this type of C–H activation occurs fastest at methyl branches (preferred by 8 kJ mol^–1^).^154^ C–C bond cleavage occurs in a metal ion-dependent fashion via either α-alkyl or β-alkyl elimination. The latter produces an olefin, while the former generates an alkylidene, both of which can be subsequently hydrogenated to alkanes.^151^ β-Alkyl transfer from the alkyl ligand to the metal favors the elimination of smaller methyl groups over larger alkyl groups (by ca. 4 kJ mol^–1^).^154^ Finally, cleavage of Zr–C bonds by σ-bond metathesis is preferred at primary carbons vs. secondary carbons (by ca. 4 kJ mol^–1^). These insights explain differences in the degradation rates and products obtained from specific polymers. For example, PP degradation produces large amounts of undesirable methane by β-methyl elimination. At the same time, β-H elimination suppresses the chain walking that interconverts primary, secondary and tertiary Zr–C sites. Therefore, β-R elimination (where R > CH_3_) is necessary to achieve significant shortening of the main PP chain.^154^ Tuning the relative rates of β-alkyl and β-H elimination is an important goal in the design of metal hydride catalysts.

Electrophilic *d*^0^ metal hydride catalysts with charge-neutral active sites currently have limited practical appeal due to their very low hydrogenolysis rates. More reactive cationic Zr sites were obtained by supporting Zr(CH_2_^t^Bu)_4_ on a highly Bro̷nsted acidic sulfated alumina,^148^ in which surface sulfate sites behave as very weakly basic, non-coordinating anions. Cationic [ZrH]^+^ sites were found to be at least 200× more active than the charge-neutral [ZrH] sites present on silica-alumina.^149^ The former were reported to catalyze the hydrogenolysis of several POs (namely, PE, iPP, a polyethylene-*co*-1-octene (PECO) copolymer, and post-consumer PE) under conditions as mild as 90 °C and 0.5 bar H_2_. Activity in the hydrogenolysis of PE (*M*_n_ = 11,620 g/mol, *Đ* unspecified) to light hydrocarbons was estimated to be 4,000 mol_CH2_ mol_Zr_^–1^ h^–1^ at 200 °C under 2 bar H_2_.

**Figure 19:**
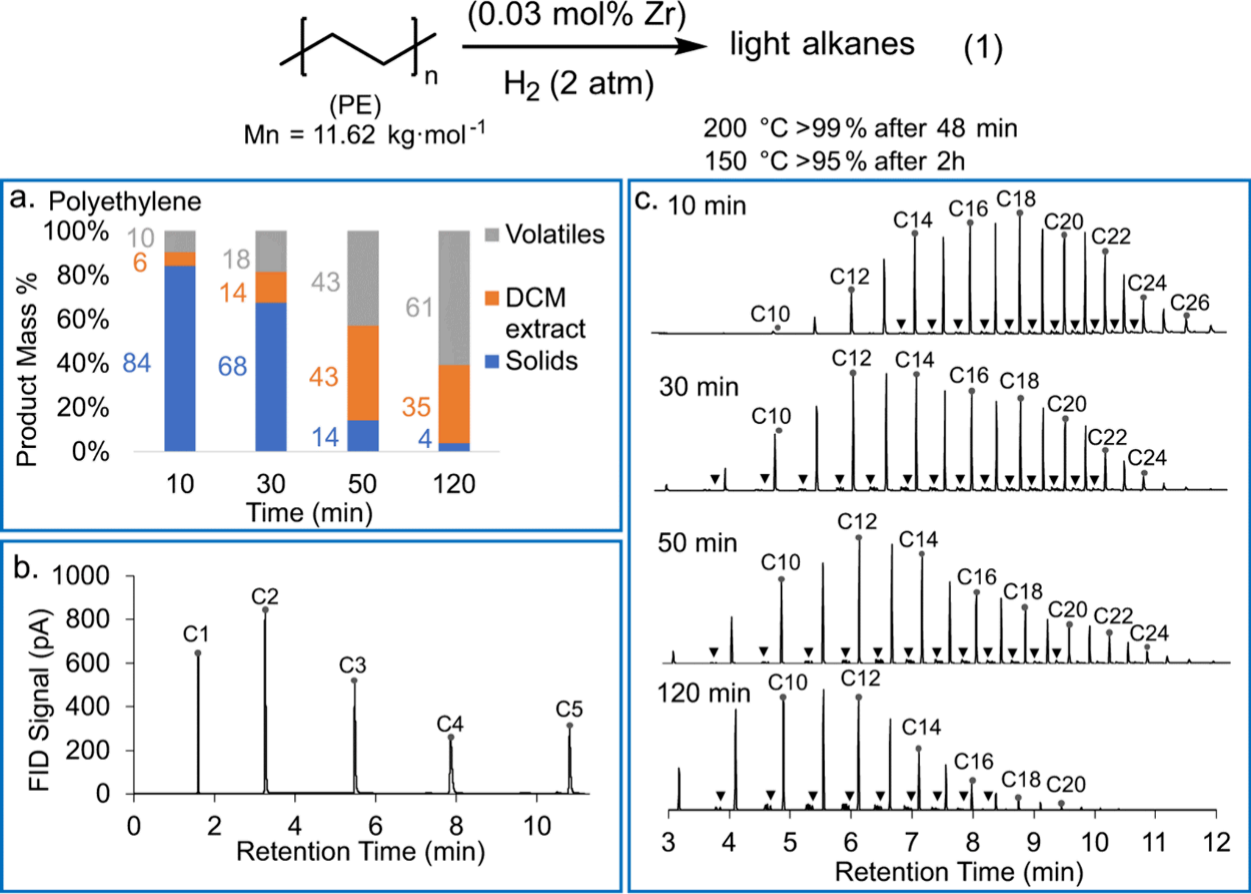
Results of PE hydrogenolysis catalyzed by ZrNp_2_/sulfated alumina (0.03 mol% Zr) at 150 °C under 2.0 bar H_2_: (a) time-dependent product distributions; (b) GC/FID analysis of volatile products after 30 min; and (c) GC/MS analysis of soluble products extracted into CH_2_Cl_2_, after various reaction times. Linear and branched alkanes are indicated with circles and triangles, respectively. Reprinted with permission from ref (148). Copyright 2022, Springer Nature.

The results of PE hydrogenolysis are shown in Figure 19: after 2 h, the yield of volatile and liquid products was 96 %, with PE residue (*M*_n_ < 0.86 kg mol^–1^) comprising the rest. The average chain length of the CH_2_Cl_2_-soluble products declined from C_18_ at 10 min to C_11_ at 2 h (Figure 19c). Hydrogenolysis of iPP (*M*_n_ = 36,000 g mol^-1^, *Đ* unspecified) converted the polymer to low-molecular-weight products (C_<30_, 68 wt%) and C_1_–C_6_ volatiles (28 wt%) under 2 bar H_2_ in just 1 h. Kinetic studies using *n*-hexadecane (C_16_H_34_) as a model for PE yielded the power rate law *k*[Zr]^1^[H_2_]^0^[C_16_]^0^, which implies that the rate-limiting step does not involve direct H_2_ attack on Zr. Two mechanistic scenarios were investigated: C–C bond scission via β-alkyl transfer with a DFT-calculated barrier of 110 kJ/mol was suggested to be rate-limiting, and much faster than C–C bond scission by σ-bond metathesis, whose 320 kJ/mol barrier is prohibitive.^148^

A comparison of hydrogenolysis activity and stability was undertaken for three supported group-IV organometallic catalysts (MNp_2_/sulfated alumina, where M = Ti, Zr, Hf, and Np = neopentyl) with various pre- and post-consumer POs.^155^ The rates of C–C bond cleavage, normalized by mol metal, decreased in the order Zr > Hf > Ti, while the order of catalyst thermal stability was Hf ≈ Zr > Ti. These trends are consistent with the DFT calculated-energy barriers for β-alkyl elimination and the relative thermal stabilities of MNp_4_, respectively.

Cationic [TaH]^+^ sites made by supporting Ta(=CH^t^Bu)(CH_2_^t^Bu)_3_ on sulfated alumina were similarly reported to be more active in hydrogenolysis than neutral [TaH] sites supported on silica.^156^ At 150 °C, the rate of smaller hydrocarbon production from *n*-C_14_H_30_ was 235 mol/mol_Ta_ for [TaH]^+^, compared to 59 mol/mol_Ta_ for [TaH]. In the hydrogenolysis of HDPE (*M*_n_ = 2,500 g/mol; *Đ* = 3.6) catalyzed by [TaH]^+^ under 1 bar H_2_, the yield of C_1_-C_28_ products (with a distribution centered at C_18_) was 30 wt% after 4 h. Interestingly, the residual polymer had a much higher molecular weight (*M*_n_ = 8,500 g/mol; *Đ* = 1.5) than the original polymer, possibly due to alkane metathesis catalyzed by the same active sites (see section 4.3.6 below).^156^ A [TaH]^+^ catalyst made by the same synthetic approach was reported to have a hydrogenolysis activity for PE (*M*_n_ = 8,300 g/mol, *Đ* unspecified) of 9,800 (CH_2_ units)·mol_Ta_^–1^·h^–1^ at 200 °C under 17 atm H_2_. The cationic Ta sites were thermally more stable than the Zr analogs, and were not deactivated by typical PO additives.^157^

Hydrogenolysis of iPP (*M*_n_ = 13,300 g/mol; *Đ* = 2.4; frequency of isotactic pentads *mmmm* = 94 %) was investigated using [TaH]^+^ supported on sulfated alumina.^158^ At 200 °C under 1 bar H_2_, 80 wt% of the polymer was converted to a mixture of branched alkanes in 24 h, with product yields of 70 wt% in the range C_11_–C_30_ and CH_4_ yields as low as 3.5 wt%. Under identical conditions, a much smaller amount of HDPE (*M*_n_ = 4,900 g/mol; *Đ* = 3.1), 27 wt%, was converted to light alkanes, presumably due to less facile β-alkyl elimination in the sterically crowded [TaR]^+^ sites.^159^^13^C{^1^H} NMR analysis indicated complete loss of tacticity in the branched alkane products, while tacticity was largely conserved in the residual iPP (*mmmm* = 93%). Cleavage of iPP in the presence of D_2_ gave similar alkane mixtures with deuterium incorporated into all possible positions (1°, 2°, 3°), but the residual iPP contained ten times more deuterated methyls (CH_3–*x*_D_*x*_) than deuterated methylenes (CH_2–*x*_D_*x*_) groups and negligible deuterated methines (CD). Thus [TaH]^+^ sites do not activate methine groups.^158^

**Scheme 8:**
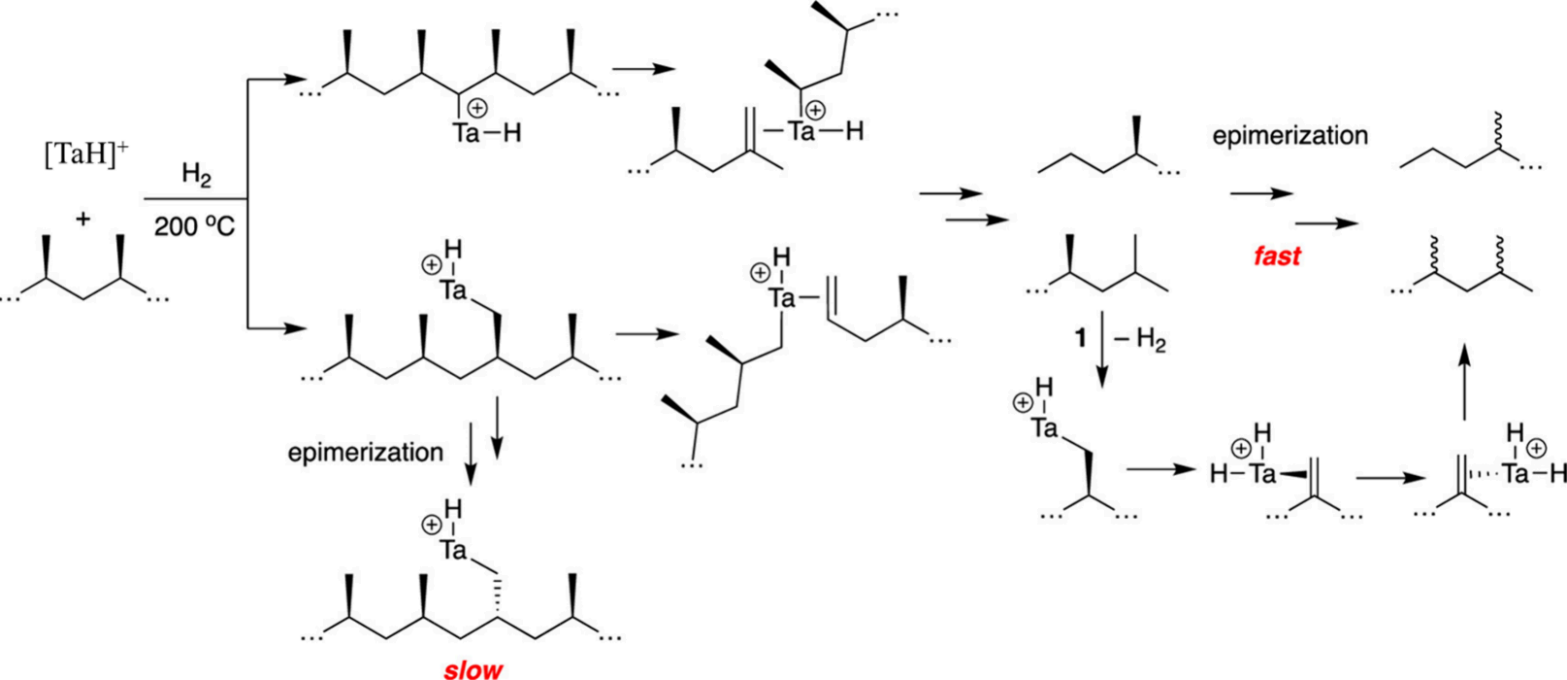
Proposed Mechanism for iPP Hydrogenolysis Catalyzed by a Cationic Tantalum Hydride Supported on Sulfated Alumina Reprinted with permission from ref (158). Copyright 2023, American Chemical Society.

The results are consistent with the iPP hydrogenolysis mechanism proposed in Scheme 8. Secondary and tertiary C–H bonds in iPP are activated preferentially by [TaH]^+^, forming [TaR]^+^. Since chain-walking is slower than β-alkyl elimination, iPP tacticity is preserved. However, epimerization in the small molecule alkanes is fast relative to iPP hydrogenolysis by β-alkyl elimination, presumably due to the greater flexibility of shorter chains which allows them to adopt the necessary conformations. Scheme 8 shows that [TaR]^+^ epimerizes via a classical β-H elimination mechanism, followed by unselective alkene insertion.^160^ Consequently, the stereoregularity changes during iPP hydrogenolysis depending on the relative rates of β-alkyl elimination and epimerization, which vary with the alkyl chain length in the [TaR]^+^ sites.^158^ Degradation of the same iPP catalyzed by cationic [HfH]^+^ sites (derived from the reaction of [HfCH_3_]^+^ sites with H_2_) under identical conditions gave a better yield of liquid hydrocarbons (83 wt%, *M*_n_ = 290 g/mol), although the products were also atactic.^161^

### 2.4 Structure–Performance Relationships

Aiming to narrow the hydrogenolysis product distribution and suppress methane formation, a large number of studies have explored catalyst structure–performance relationships. Strategies include controlling the active metal (e.g., size, morphology, oxidation state, promoters), support effects (e.g., metal–support interactions, pore architecture), and optimization of reaction conditions (e.g., reactor configuration, solvent addition).

#### 2.4.1 Active Metal State

Varying the metal loading in catalysts based on supported nanoparticles can alter the average nanoparticle size and size distribution, resulting in changes in the relative abundances of surface and interface sites. In methylcyclopentane hydrogenolysis, C–C bond scission on small (<1.5 nm) Pt nanoparticles rich in undercoordinated corner sites was suggested to explain the preferential formation of *n*-hexane, via a metallacyclobutane intermediate.^87^ Pt/SrTiO_3_ prepared with different nanoparticle sizes (1.2, 2.3, and 2.9 nm) and a constant total mass of Pt was used to catalyze the hydrogenolysis of PE (*M*_n_ = 8,150 g/mol, *Đ* = 2.7). The smallest nanoparticles converted PE completely to gas products, while larger nanoparticles formed liquid products in the lubricant-range (average *M*_n_ = 800 g/mol, *Đ* = 1.1).^54^ The higher fraction of undercoordinated Pt sites in the smallest nanoparticles was suggested to promote hydrogenolysis, maximize the liquid product yield, and narrow the dispersity.^162^ A similar phenomenon was reported for Pt/C (5 wt% Pt) with different nanoparticle sizes (2.5–58 nm) in the hydrogenolysis of iPP (*M*_w_ = 12,000 g/mol, *Đ* unspecified), where the average carbon number in the products decreased from C_>45_ (58 nm nanoparticles) to C_13_ (2.5 nm nanoparticles).^135^

A semi-quantitative assessment of the hydrogenolysis rate for PE (*M*_n_ = 20,000 g/mol, *M*_w_ = 90,000 g/mol) based on the mass of wax products (C_<45_) revealed that the rate per surface metal atom for 5 nm Pt nanoparticles was ca. 50 % of the rate for 1.7 nm Pt nanoparticles. This finding was presented as evidence for the structure-sensitivity of PE hydrogenolysis on Pt.^131^ For comparison, ethane hydrogenolysis is much more sensitive to Pt nanoparticle size in Pt/SBA-15 (0.6 wt% Pt), with turnover frequencies increasing 2 orders of magnitude for nanoparticles from 1 to 10 nm (375 °C, H_2_/C_2_H_6_ = 10).^163^

Since one C–C bond scission event consumes one H_2_ molecule, the hydrogenolysis rate can be assessed by monitoring the rate of H_2_ consumption (assuming no other side-reactions consume or generate H_2_ – see section 1.2.2.1). Although this simple approach has not yet been widely used in evaluating hydrogenolysis activity, it has the potential to avoid large errors introduced by simply weighing complex product mixtures (liquids, solids) and by incomplete product recovery. Using this method, the rate of hydrogenolysis of PE (*M*_n_ = 1,700 g/mol, *M*_w_ = 4,000 g/mol) catalyzed by Ru/CeO_2_, normalized per surface Ru, decreased monotonically as the Ru nanoparticle size increased from 1 to 10 nm. For comparison, the yield of gas products (C_1_–C_4_) showed little dependence on nanoparticle size at similar PE conversions (30 to 50 %).^121^ However, the monotonic relationship was no longer valid when the nanoparticle size reached the sub-nm level.

**Figure 20:**
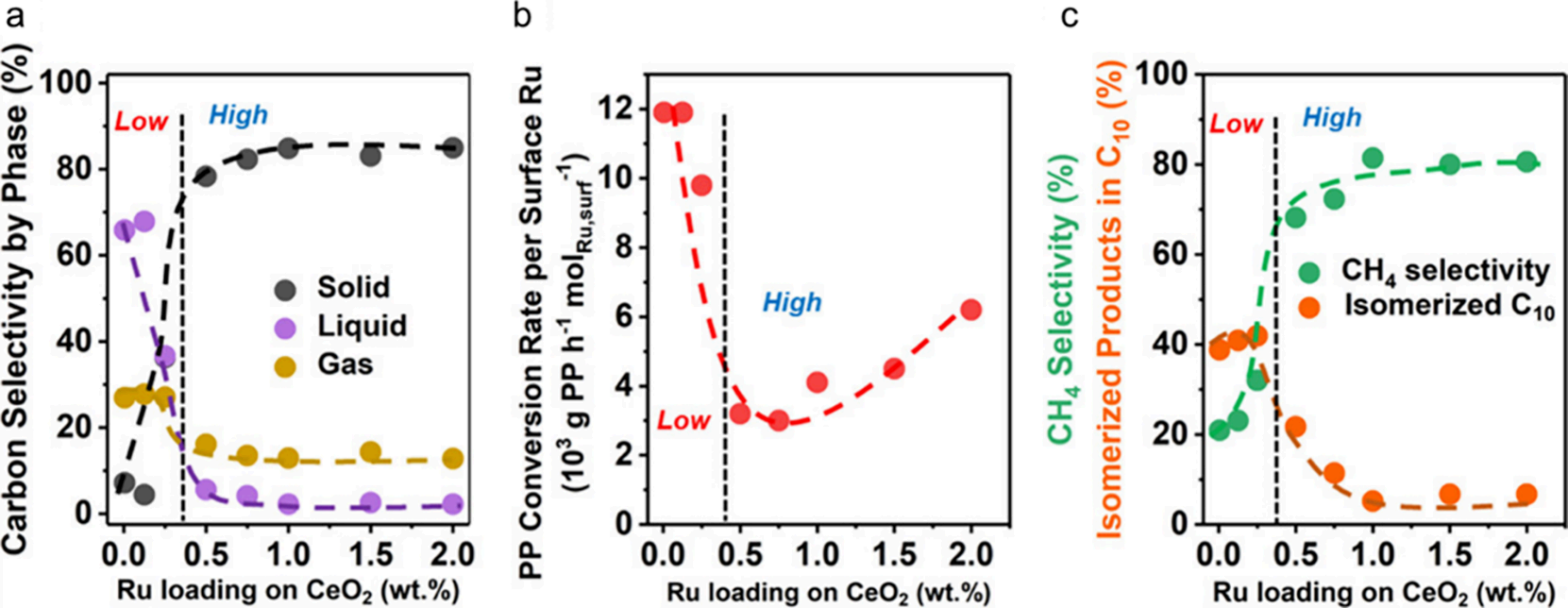
Dependence on Ru loading of iPP hydrogenolysis catalyzed by Ru/CeO_2_: (a) overall product phase selectivity; (b) rate of conversion, normalized by surface Ru atoms; and (c) selectivity to CH_4_ and C_10_ isomers. Reaction conditions: 1 g iPP (*M*_w_ ∼250,000 g/mol, *M*_n_ = ∼67,000 g/mol), 0.5 mg Ru, 260 °C, 30 bar H_2_, 18 h. Reproduced with permission from ref (106). Copyright 2022, American Chemical Society.

A series of Ru/CeO_2_ catalysts with 0.05 to 2.0 wt% Ru was tested in the hydrogenolysis of iPP (*M*_w_ = 250,000 g/mol, *M*_n_ = 67,000 g/mol). As shown in Figure 20a, when the Ru loading was higher than 0.5 wt% (average Ru nanoparticle size: ca. 1.5 nm), the amount of solid residue was <25 % and depended little on the Ru loading. In contrast, when the Ru loading was ≤0.25 wt% (average Ru nanoparticle size < 1.5 nm), the non-solid fraction increased abruptly, reaching 95 % for 0.125 wt% Ru. The rate of iPP hydrogenolysis (estimated from the amount of solid residue remaining) was normalized by the number of surface Ru atoms to obtain the intrinsic catalytic activity. It decreased with nanoparticle size for high Ru loadings (>0.5 wt%), then increased abruptly as the particle size decreased further (at low loading, ≤0.25 wt%), Figure 20b. The rate normalized by surface Ru was a maximum at 11 g/(h·g_Ru_) for the lowest Ru loading. Low loadings also favored branched liquid alkanes relative to CH_4_, whereas methane selectivity was high for catalysts with high Ru loadings (Figure 20c). The selectivity trends also held when compared at similar conversions, and when the reactant was PE or *n*-C_16_H_34_. This dependence on Ru loading suggests a significant difference in the mechanism for Ru loadings above and below ca. 0.5 wt%.^106^

Further structural characterization revealed more information about the origin of the change in reactivity as the Ru loading passed through this threshold. Scanning transmission electron microscopy (STEM) images showed that Ru/CeO_2_ (0.125 wt% Ru) consisted of sub-nm particles (<0.8 nm), possibly present as disordered two-dimensional rafts. Extended X-ray absorption fine structure (EXAFS) analysis confirmed that the metallic nanoparticles were crystalline at high Ru loadings, but they became highly disordered at low Ru loadings (<0.25 wt%), with no well-defined scattering paths beyond the nearest neighbors. The threshold for this Ru structural transition coincided with the change in hydrogenolysis performance. However, the authors suggested that Ru particle size was not the cause of the observed changes in catalytic behavior, despite the clear correlation between structure and size. X-ray Absorption Near-Edge Structure (XANES) and X-ray photoelectron spectroscopy (XPS) suggested that Ru became more cationic as the nanoparticle size decreased. Therefore, catalytic behavior was correlated with both oxidation state and structural ordering in the Ru-based catalysts. This behavior differs from previous reports, where smaller metal particles showed lower TOFs and higher selectivities to CH_4_ and branched alkanes.^121^

Ru/CeO_2_ catalysts containing either 0.125 or 2 wt% Ru were analyzed for how changes in H coverage upon raising the H_2_ pressure from 5 to 60 bar affect the hydrogenolysis rate. For the lower Ru loading, the rate of iPP hydrogenolysis increased with increasing H_2_ pressure initially, then decreased above 30 bar. For the higher Ru loading the rate increased monotonically with H_2_ pressure. The negative order in H_2_ of the catalyst with lower loading was attributed to a higher surface coverage of atomic H, relative to adsorbed hydrocarbons. Higher H coverage on the catalyst with low Ru loading was confirmed by H_2_ chemisorption measurements. The lower CH_4_ selectivity of this catalyst is consistent with this higher H coverage, since the inability to accommodate more surface H by dehydrogenation of the polymer chain favors internal C–C bond cleavage.^106^

Eliminating metal clusters/ensembles by creating atomically-dispersed metal catalysts could limit the interaction of PO chains with multiple sites and thereby suppress the successive fragmentation of a single chain that leads to methane formation.^117^ According to DFT calculations on *n*-hexane hydrogenolysis catalyzed by Ru single sites and trimers, internal cleavage should be favored and methane formation suppressed at atomically-dispersed Ru sites. Ru/CeO_2_ catalysts with various Ru loadings (from 0.2 to 5 wt%, labeled xRu/CeO_2_ where *x* indicates the Ru content in wt%) were synthesized by a wet impregnation method. 0.2Ru/CeO_2_ was suggested to have isolated Ru sites and an oxidation state of Ru^4+^ after H_2_ reduction at 300 °C, while 2Ru/CeO_2_ had mainly metallic Ru nanoparticles. Using *n*-hexadecane as a model compound, hydrogenolysis was conducted at 250 °C under 20 bar H_2_ for 8 h, catalyzed by Ru/CeO_2_. While both catalysts converted 100 % of the C_18_, the 2Ru/CeO_2_ catalyst gave methane in 87 % yield, while 0.2Ru/CeO_2_ produced only 3 % methane (the average carbon number in the products was C_8_).^117^

**Figure 21:**
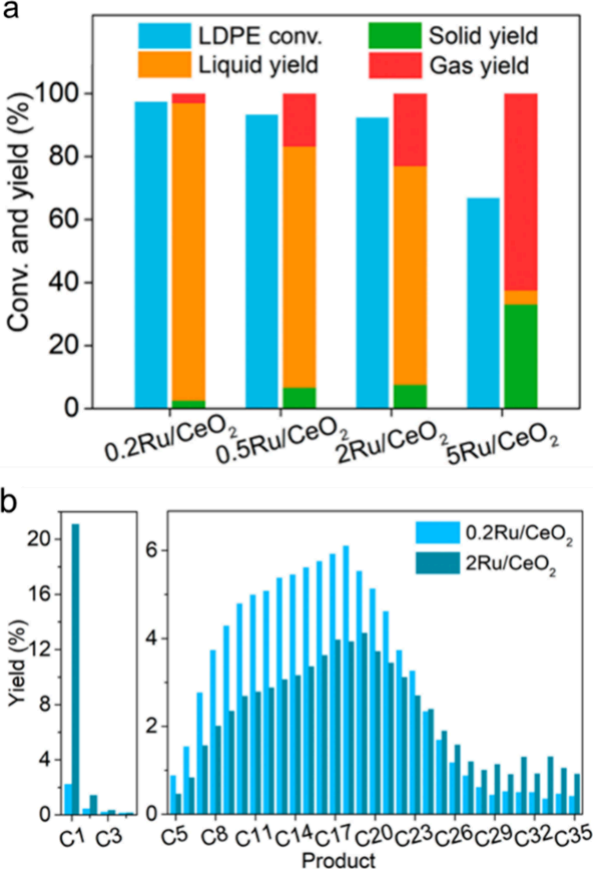
Comparison of PE hydrogenolysis catalyzed by Ru/CeO_2_ with various Ru loadings: (a) conversion and product phase yields; and (b) carbon number distribution. Reaction conditions: 250 °C, 6 h, 20 bar H_2_, PE (*M*_w_= 4,000 g/mol, *Đ* unspecified), constant PE/Ru mass ratio of 2000. Adapted with permission from ref (117). Copyright 2023, AAAS.

The effect of Ru loading was also investigated in the hydrogenolysis of PE (*M*_w_= 4,000 g/mol, *Đ* unspecified), while keeping the PE/Ru ratio constant.^117^ The results are shown in Figure 21. The catalyst with the lowest Ru loading gave the least PE residue (2.5 wt%), although the amount was only slightly higher (5 wt%) for the catalyst with the highest loading. The catalysts with 0.2 and 2 wt% Ru both gave liquids with carbon numbers centered at C_16_–C_18_, but their methane yields differed significantly (2 vs. 21 %). The gas yield of 0.2Ru/CeO_2_ is very low among reported Ru-based hydrogenolysis catalysts. This catalyst also performed successfully with commercial post-consumer plastics, including PE bags, HDPE bottles, and PP bottles, which contained a variety of additives and impurities. Liquid yields were over 80 wt% and gas yields were below 10 wt% at solid conversions above 90 %. However, another study using an atomically-dispersed Ru catalyst supported on CoAlO_x_ to convert pyrolysis intermediates (produced at 520 °C under 1.5 bar H_2_ from mixed plastic waste containing PP, PE, PET) via hydrogenolysis at 350 °C and 1.5 bar H_2_ reported that methane was the only hydrocarbon product, in >99 wt% yield.^164^ The differences in reported selectivities suggest the need for more investigation of the behavior of highly-dispersed catalysts.

An influence of metal oxidation state on methane formation in the hydrogenolysis of PE (*M*_w_= 4,000 g/mol, *M*_n_ = 1,700 g/mol) was reported for Ru/CeO_2_, where cationic Ru favored internal C–C bond cleavage over terminal C–C bond cleavage. The effect was attributed to the higher electron density at internal C(2°) sites compared to terminal C(1°) sites.^119^ In another study, Ru/C (5 wt% Ru) was modified using ethylene glycol (EG).^165^ Both Ru/C and Ru/C-EG had ca. 2 nm Ru nanoparticles. A single reduction peak at ca. 150 °C, attributed to Ru reduction, was observed in the TPR of Ru/C, while in the presence of EG (ca. 35 mol% relative to total Ru atoms), an additional reduction peak at ca. 300 °C appeared, suggesting that EG stabilizes cationic Ru. In the hydrogenolysis of PE (1.0 g, *M*_w_= 4,000 g/mol, *M*_n_ = 1,700 g/mol) at 240 °C under 2 MPa H_2_, Ru/C-EG showed nearly 5× higher solid conversion compared to Ru/C (91 vs. 20 %). A similar increase was reported in the rate of *n*-hexadecane hydrogenolysis, although Ru/C and Ru/C-EG showed the same activity for propylene hydrogenation. The authors concluded that the rate of C–C bond cleavage (step d in Scheme 4) determines hydrogenolysis activity. According to *in situ* XPS under 0.2 mbar H_2_ at 300 °C, the Ru^δ+^/Ru^0^ atomic ratio was 1.4 for Ru/C-EG, and 0.7 for Ru/C. A DFT model predicted that the EG-covered surface facilitates electron transfer from the C–C bond of EG to Ru^δ+^, resulting in an increased rate of C–C bond scission in PE. Ru^0^ sites were deemed responsible for both C–H bond activation and hydrogenation of adsorbed alkyl groups.^165^

Ni-based catalysts made by reduction of a nickel aluminate (NiAl-T, where T is the reduction temperature) also showed a structure-methane selectivity relationship.^142^ Reduction led to exsolution of Ni nanoparticles, which catalyzed hydrogenolysis of PE (*M*_w_ = 4,000 g/mol, *Đ* unspecified) at 300 °C under 30 bar H_2_. After 2 h, NiAl-250 exhibited no activity, while catalysts reduced at higher temperatures all gave ca. 45 % conversion. However, the methane selectivities (among the C_<35_ products) varied significantly, from 5 to 18 to 33 % for NiAl-350, NiAl-450, and NiAl-550, respectively. There was no correlation between Ni nanoparticle size (evaluated post-reaction) and methane selectivity.

**Figure 22:**
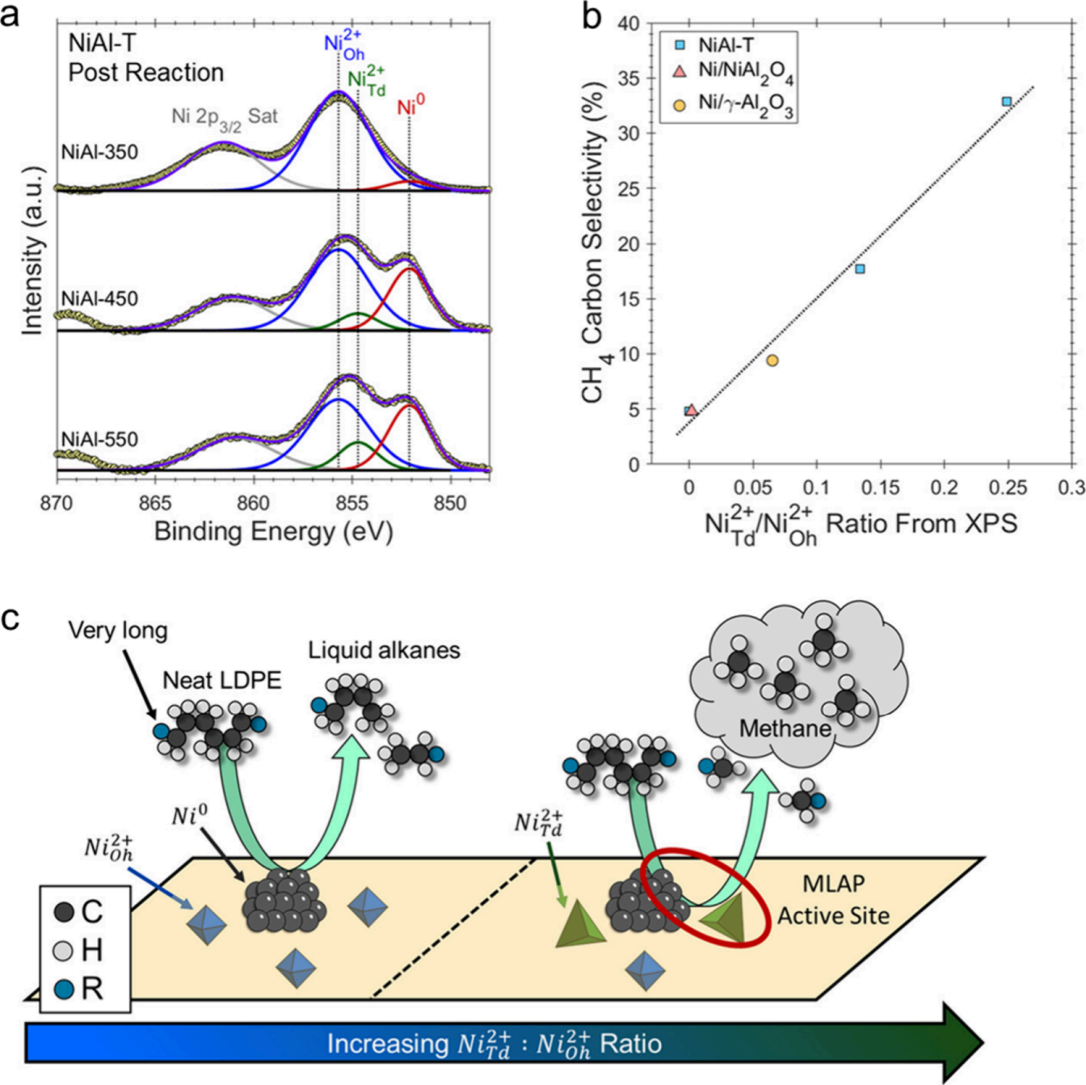
(a) Post-reaction air-free XPS spectra of reduced nickel aluminate (NiAl-T) catalysts, in the Ni 2*p* region. (b) Correlation between methane selectivity and the ratio of tetrahedral to octahedral Ni sites. (c) Schematic illustration showing methane production promoted by Ni_Td_^2+^. Reaction conditions: 2.0 g PE (*M*_w_ = 4,000 g/mol, *Đ* unspecified), 200 mg catalyst, 300 °C, 30 bar H_2_. Adapted with permission from ref (142). Copyright 2023, American Chemical Society.

The post-reaction oxidation state of Ni (after 14 h) was assessed by XPS, Figure 22a. The Ni 2*p*_3/2_ peak was deconvoluted into three contributions: Ni_Oh_^2+^, Ni_Td_^2+^, and Ni^0^. The Ni_Td_^2+^/Ni_Oh_^2+^ ratio was linearly correlated with CH_4_ selectivity in PE hydrogenolysis (Figure 22b). A comparison of Lewis acidity (evaluated by IR of adsorbed pyridine) with the fraction of Ni_Td_^2+^ implied that these sites are the source of Lewis acidity (notably, inactive NiAl-250 had no Lewis acidity). The linear trend was suggested to arise due to metal nanoparticle-Lewis acid (Ni_Td_^2+^) pairs, Figure 22c. However, there was no molecular-level explanation for how these paired sites might enhance hydrogenolysis.^142^

#### 2.4.2 Promoter and Support Effects

The performance of metal catalysts in PO hydrogenolysis differs widely depending on the nature of the support.^166^ For example, screening of various metal-support combinations led to the identification of Ru supported on ZrO_2_ (among SiO_2_, γ-Al_2_O_3_, and Nb_2_O_5_)^121^ and Ni supported on SiO_2_ (among AC, ZrO_2_, γ-Al_2_O_3_, and CeO_2_)^141^ as the best hydrogenolysis catalysts for the respective metals. The presence of promoters can also alter the geometric and electronic properties of the metal, as well as the metal-support interface.

Most research has focused on controlling hydrogenolysis product selectivity. For example, addition of V to Ru/SiO_2_ (5 wt% Ru) improved regioselectivity in squalane hydrogenolysis (240 °C, 60 bar H_2_), although at the expense of activity. Methane formation was suppressed, resulting in enhanced selectivity for products in the range C_14_–C_16_ for V/Ru = 0.25.^88^ Bimetallic alloys RuM_3_/CeO_2_ (M = Fe, Co, Ni, 1 wt% Ru, 1.7 wt% M) were shown to tailor the product selectivity in hydrogenolysis of PE (1.0 g, *M*_w_ = 4,000 g/mol, *Đ* unspecified) under 18 bar H_2_ at 250 °C, at the expense of catalytic activity. Comparison of the product distributions for Ru/CeO_2_ and RuCo_3_/CeO_2_ at similar PE conversions showed that the latter favors longer-chain hydrocarbons (C_7_–C_25_). The effect was attributed to weaker interactions of the hydrocarbons with the RuM_3_ alloy.^167^

The effect of highly-dispersed WO_x_ clusters on Ru/ZrO_2_ (5 wt%, Ru–Zr) was investigated in the hydrogenolysis of LDPE (*M*_w_ = 76,000 g/mol, *Đ* unspecified).^103^ Under mild reaction conditions (250 °C, 50 bar H_2_, 2 h), the presence of 25 wt% WO_3_ (Ru-WZr) had a significant effect on the product distribution for a LDPE/catalyst mass ratio = 40. At similar conversions (60-70 %), the methane yield decreased from 16 to 5 %. Although the gasoline-range selectivity (C_5_–C_12_) remained relatively constant (ca. 25 %), the fraction of jet fuel (C_8_-C_16_) increased from 33 to 42 %, the diesel fuel fraction (C_9_-C_22_) increased from 47 to 66 %, and the wax/lubricant base-oil yield (C_20_-C_35_) increased from 22 to 35 %. Overall, the presence of WO_x_ shifted the carbon distribution to larger, value-added alkanes. Hydrogenolysis of single-use plastics using this catalyst gave a similar enhancement in product selectivity.

The amorphous WO_x_ clusters were ca. 1 nm in size, and partially covered the Ru nanoparticles (ca. 5 nm). Ru-WZr had negligible isomerization activity, suggesting the absence of hydrocracking. Although the Bro̷nsted acidity of WZr was weak, the C–C bond scission rate was high. In the hydrogenolysis of *n*-hexane, Ru–Zr and Ru-WZr catalysts gave identical TOFs for C–C bond scission (molecules of *n*-hexane consumed per surface Ru site per s), implying that the electronic properties of Ru were not affected by the presence of WO_x_.^103^ In LDPE hydrogenolysis catalyzed by Ru–WZr, methane was the major product in the first hour. The non-solid yield increased abruptly after 1.5 h. Higher H_2_ pressure was proposed to promote the rate-limiting hydrogenation of alkyl surface intermediates and their desorption, and to suppress methane formation.

**Scheme 9:**
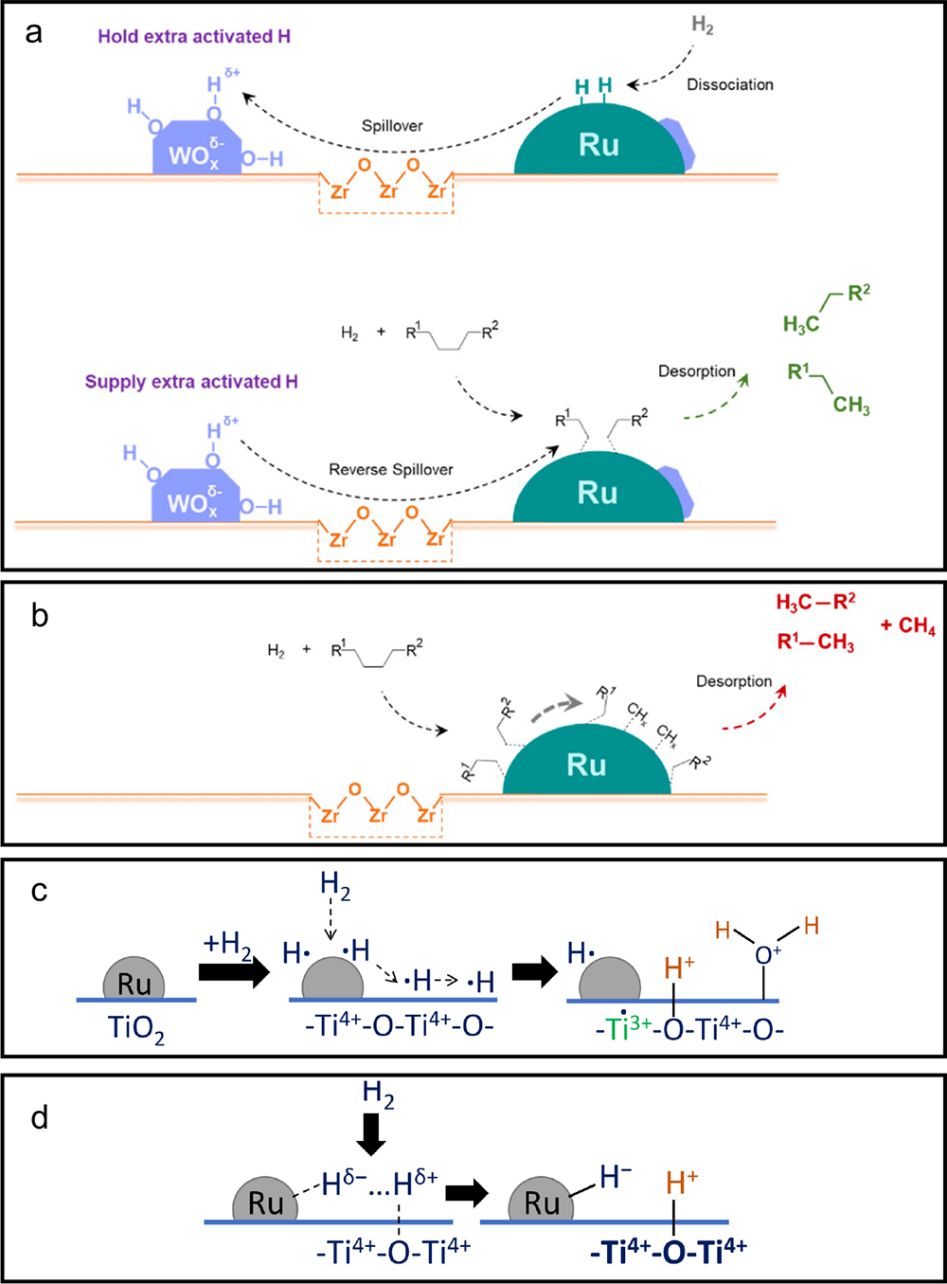
Polyolefin Hydrogenolysis Promoted by Hydrogen Spillover: Comparison of Reaction Pathways for (a) WO_x_-Modified Ru/ZrO_2_; and (b) Ru/ZrO_2_. (c) Hydrogen Spillover; and (d) H_2_ Dissociation, Both for Ru/TiO_2_ Panels a and b: Adapted with permission from ref (103). Copyright 2021, American Chemical Society. Panels c and d: Adapted with permission from ref (116). Copyright 2022, Springer Nature.

*In situ* transmission IR of Ru–WZr revealed the formation of WO–H bonds under H_2_, and their disappearance under He. Based on H_2_–TPR and TPD, Ru–WZr experienced H_2_ spillover, with H_2_ dissociation on Ru and H diffusion to WO_x_, where it encountered Lewis basic oxygen atoms (W=O or W–O–W). The proposed effect is illustrated in Scheme 9a,b, where highly mobile H formed by reversible spillover promotes hydrogenation and desorption of alkyl intermediates. The study suggested that methane production can be suppressed by increasing the H_2_ storage capacity of Ru-based catalysts.^103^ A further study revealed that V and Mo oxides are also effective in inhibiting methane production, since they are reducible and can supply hydrogen to Ru through reverse hydrogen spillover.^122^

A hydrogen spillover effect was also observed in Ru/TiO_2_, where it resulted in enhanced hydrogenolysis activity towards iPP (*M*_w_ = 250,000 g/mol, *M*_n_ = 67,000 g/mol).^116^ Ru supported on anatase TiO_2_ was synthesized by an impregnation method, using aqueous NH_3_ to control pH. The catalysts were labeled Ru/TiO_2_-x, where x indicates the pH. IR of adsorbed CO showed that Ru/TiO_2_-8 had more Lewis acidic five-coordinate Ti^4+^ sites and a lower density of surface TiOH groups than Ru/TiO_2_-1. In Ru/TiO_2_-8, the ν(CO) vibration in Ru^δ+^(CO)_*m*_ species shifted to lower wavenumbers (from 2077 to 2059 cm^-1^), due to the presence of a TiO_x_ overlayer. During H_2_-TPD, hydrogen spillover increased the H_2_ uptake. Partial reduction of TiO_2_ resulted in TiOH groups (Scheme 9c, d). IR and Raman spectroscopies showed that reduction of Ru/TiO_2_-1 generated mainly localized Ti^3+^ states, whereas reduction of Ru/TiO_2_-8 resulted in higher H coverage and produced delocalized electrons in the conduction band.

In iPP hydrogenolysis (250 °C, 30 bar H_2_, polymer/catalyst mass ratio = 20), Ru/TiO_2_-8 gave 4× more liquid yield per h and a lower average molecular weight in the liquid products than Ru/TiO_2_-1. Both gave similar methane yields, up to 10 wt%, at long reaction times. For Ru/TiO_2_-1, the rate was faster at higher H_2_ pressure (50 vs. 30 bar), but still not faster than Ru/TiO_2_-8. A kinetic isotope effect of 5.8 was observed for Ru-TiO_2_-1, but not for Ru-TiO_2_-8. The difference was attributed to the higher H coverage on Ru/TiO_2_-8, which made the rate less sensitive to *P*_H2_. Lower H coverage on Ru/TiO_2_-1 made dehydrogenation more kinetically significant, accounting for slower polymer deconstruction in D_2_.^116^

Creating structures that store more hydrogen therefore appears to be key to more efficient hydrogenolysis catalysts. In a related example, installing a TiO_x_ capping layer on Ru via the strong metal-support interaction facilitated hydrogenation/desorption of alkyl intermediates and led to a doubling of the propylene hydrogenation rate relative to the uncapped Ru catalyst.^111^ Alloying a Co nanoparticle catalyst derived from a CoAl-layered double hydroxide with Ni also enhanced H_2_ spillover, leading to improved hydrogenation/desorption of alkyl intermediates and significantly reducing CH_4_ production.^168^

#### 2.4.3 Catalyst Architecture

Other components of heterogeneous catalyst structures can assist in adsorbing and/or constraining polymer chains, altering their interactions with the metal active sites, and thereby influencing the outcome of hydrogenolysis.^169^ Several studies have discussed the role of polymer-support interactions in hydrogenolysis. When the catalyst consists of metal nanoparticles supported on an oxide, the interactions of polymer chains with metal nanoparticles are typically stronger than with the oxide support. For example, ^13^C-enriched PE (*M*_n_ = 132,000 kg/mol, *Đ=* 3.2) interacted predominantly with Pt in polymer–Pt/SrTiO_3_ mixtures, as discussed in section 2.3.2.^54^

When metals are supported on activated carbons (ACs), hydrocarbon interactions with the support must also be considered.^135^ The adsorption of *n*-heptane on ACs (1300–3000 m^2^/g) with various oxygen contents (3–8 at%) was studied by TPD. The adsorption capacity for *n*-heptane was proportional to the micropore volume (which correlates with surface area), while the adsorption strength was inversely proportional to the surface O content.^135^ For comparison, amorphous SiO_2_ and γ-Al_2_O_3_ adsorbed no *n*-heptane. The activity of Pt nanoparticles dispersed on various supports (all 5 wt% Pt) was compared in the hydrogenolysis of PP (*M*_w_ = 12,000 g/mol, *Đ* unspecified, no information on tacticity) at 300 °C under 15 bar H_2_. Conversions estimated from the amount of solid residue (C_>45_) remaining after 24 h were >90 % for a Pt/AC, but <15 % for Pt/SiO_2_ and Pt/Al_2_O_3_. The carbon support, with the highest adsorption capacity for *n*-heptane, therefore showed the highest liquid yield (>90 %, C_6_–C_45_, mostly C_21_–C_45_).

**Figure 23:**
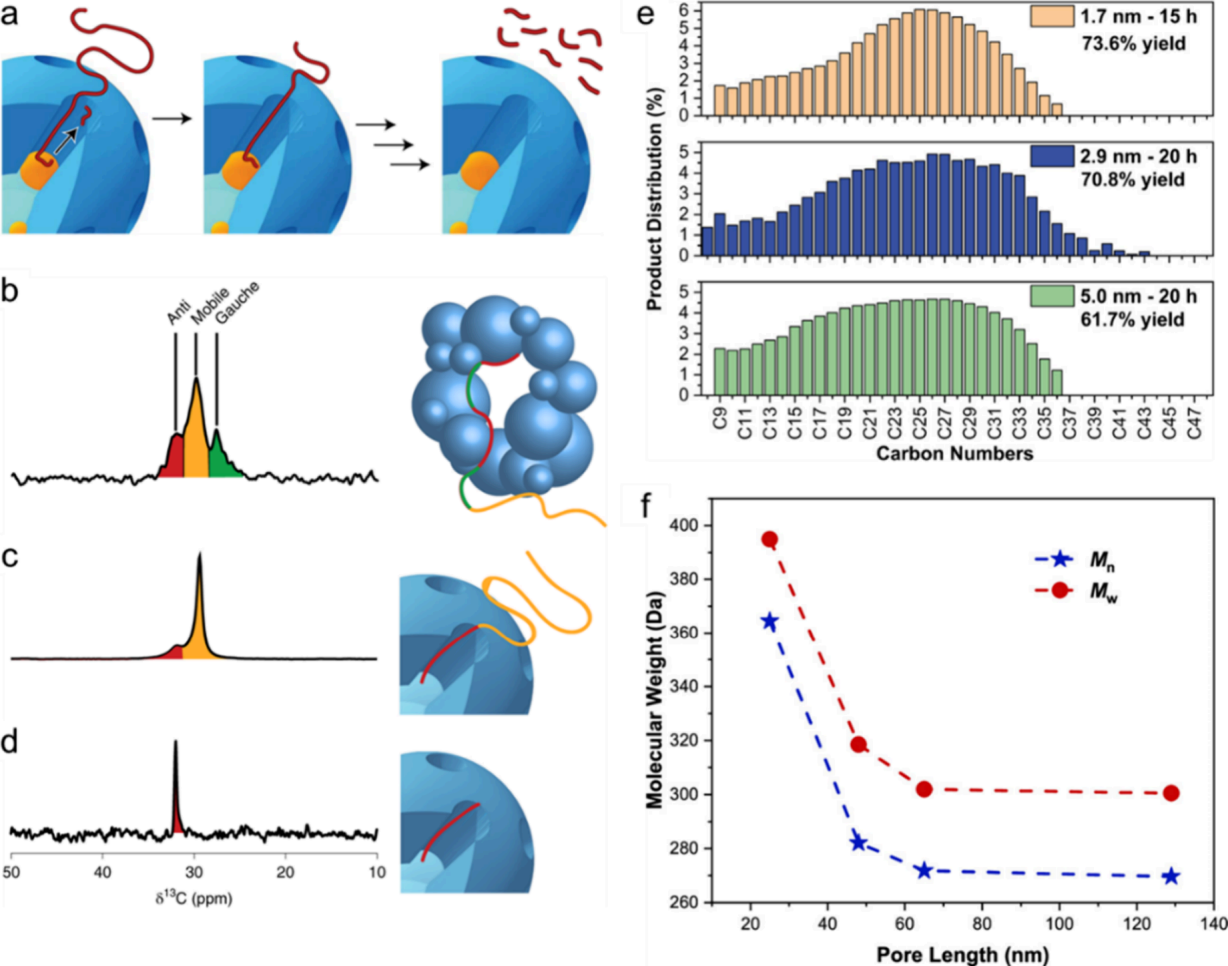
(a) Schematic of the proposed “processive” mechanism for PE hydrogenolysis catalyzed by a mSiO_2_/Pt/SiO_2_, with Pt nanoparticles (orange) located at the bottom of nanopores in the mesoporous SiO_2_ (mSiO_2_) shell. ^13^C MAS NMR spectra of ^13^C-enriched PE (*M*_n_ = 130,000 g/mol, *Đ=* 3.2) adsorbed onto (b) silica gel; or (c) mSiO_2_. (d) ^13^C MAS NMR spectra of unlabeled PE (*M*_n_ = 7,000 g/mol, *Đ* unspecified) on mSiO_2_. Proposed polymer conformations are indicated to the right of the spectra. Adapted with permission from ref (130). Copyright 2020, Springer Nature. (e) Product distributions for wax (C_<45_) recovered at times corresponding to similar LDPE conversions (ca. 80 %) from reactions catalyzed by mSiO_2_/Pt/SiO_2_ with various Pt nanoparticle sizes. Reaction conditions: LDPE (*M*_n_ = 20,000 g/mol, *M*_w_ = 90,000 g/mol), 300 °C, 8.9 bar H_2_. Adapted with permission from ref (131). Copyright 2022, American Chemical Society. (f) Dependence of the average molecular weight of the wax product on the mesopore length of the mSiO_2_/Pt/SiO_2_ catalyst. Reaction conditions: PE (*M*_n_ = 2,800 g/mol, *M*_w_ = 5,000 g/mol), 300 °C, 8.9 bar H_2_, 6 h. Adapted with permission from ref (171). Copyright 2023, American Chemical Society.

Catalytic C–C bond cleavage in PO chains typically generates a range of hydrocarbon chain lengths. Random cleavage should result if the environment around the Pt nanoparticles allows unrestricted access to any C–C bond on the external surface of a folded polymer chain. In contrast, enzymes such as cellulase and exonuclease^170^ deconstruct biomacromolecules “processively” by threading each chain into a channel inside which C–X (X = O, N) bond cleavage takes place at a precise location along the chain. A hierarchical inorganic catalyst (mSiO_2_/Pt/SiO_2_) was designed to mimic this behavior (Figure 23a).^130^ Its architecture consists of a nonporous silica nanoparticle core (diameter 127 nm), on which Pt nanoparticles (3.2 nm) were deposited. The system was then encapsulated in a mesoporous silica shell (mSiO_2_, 110 nm thick, with 2.4 nm pores oriented radially relative to the core).

The silica mesopores were suggested to induce the unfolding and elongation of long PE chains in order to maximize their van der Waals interactions with the pore walls. Interactions between HDPE and silica were characterized using ^13^C solid-state NMR. Figure 23b,c compares the interactions of PE (*M*_n_ = 130,000 g/mol, *Đ* = 3.2, 99 % ^13^C-enrichment) with a nonordered, mesoporous silica gel (Davisil, B.J.H. pore size: 12 nm, B.E.T. surface: 480 m^2^/g) and mSiO_2_ (B.J.H. pore size: 1.5 nm, 200 nm long; B.E.T. surface: 1420 m^2^/g). Anti-conformers were preferred over gauche conformers, suggesting that mSiO_2_ induces adsorbed PE chains to adopt an all-zig-zag anti-conformation, oriented along the pore axis. This interpretation is consistent with the single resonance observed for the anti-conformer of natural-abundance PE (*M*_n_ = 7,000 g/mol, *Đ* unspecified) in mSiO_2_ (Figure 23d). 2D exchange spectroscopy (EXSY) showed that ca. 70 % of the PE was adsorbed and remained inside the pores at temperatures up to 114 °C, while a lower molecular alkane (*n*-C_20_H_42_) simply desorbed, presumably because it had fewer interactions with the pore walls. Thus, mSiO_2_ mimics enzyme behavior in the sense of spontaneously incorporating the polyolefin (Figure 23a).

Hydrogenolysis of HDPE (*M*_n_ = 5,900 g/mol, *Đ* = 4.5) catalyzed by Pt (1 mg per 100 g HDPE) was performed under 13.8 bar H_2_ at 250 °C.^130^ Pt nanoparticles supported on non-porous SiO_2_ nanospheres (Pt/SiO_2_) with the same Pt nanoparticle size and Pt loading, but without the mSiO_2_ shell, served as a control. The activities of mSiO_2_/Pt/SiO_2_ and Pt/SiO_2_ were similar (based on recovered HDPE: 93 vs. 92 % after 6 h, and 76 vs 74 % after 48 h). However, mSiO_2_/Pt/SiO_2_ left the molecular weight of the unreacted HDPE largely unchanged, while the HDPE that did react was completely converted to small molecules. The carbon distribution (C_11_–C_24_, centered at C_14_ with few chains longer than C_20_) was largely independent of conversion (up to 25 %). In contrast, Pt/SiO_2_ caused changes in the polymer characteristics (molecular weight and dispersity) as the conversion increased, and the hydrocarbon product distribution was broader, with carbon numbers from C_9_ to C_36_. Thus mSiO_2_/Pt/SiO_2_ appears to retain long polymer chains in the catalyst pores until they are short enough to escape, as depicted in Figure 23a. If so, modifications to the tri-layer architecture (nanoparticle size, mesopore diameter and length, etc.) should lead to different product distributions.

The effect of Pt nanoparticle size on activity and selectivity in PE hydrogenolysis was investigated.^131^ A series of catalysts mSiO_2_/Pt-X/SiO_2_ (where X is the average Pt nanoparticle diameter: 1.7, 2.9, or 5.0 nm) was prepared. The number of active sites was estimated using ethylene hydrogenation as a probe reaction,^163^ and the catalyst weight was adjusted to ensure a constant number of active sites. Activities in hydrogenolysis of PE (*M*_n_ = 20,000 g/mol, *Đ* = 4.5, 300 °C, 8.9 bar H_2_) were compared via the amounts of gas and liquid formed per unit time (mass transfer limitations were ruled out by showing that mSiO_2_/Pt/SiO_2_ and Pt/SiO_2_ exhibited the same activity). Smaller Pt nanoparticles were more active, consistent with the reported structure sensitivity of metal catalysts in hydrogenolysis.^24^ A kinetic model involving competitive adsorption and cleavage for two lumped populations of hydrocarbons (representing long and short chains) was refined to the data to obtain apparent rate constants for the three catalysts. The difference in rate constants for C–C bond cleavage was a modest factor of two between the 1.7 and 5 nm Pt nanoparticles.

Selectivity appeared to be independent of Pt nanoparticle size, because yields of gases (C_1_–C_9_ in the reactor headspace) were similar (ca. 10–12 wt%) for all mSiO_2_/Pt-X/SiO_2_ catalysts, despite very different polymer conversions (from 25 to 70 wt%). Specifically, the yields of volatile hydrocarbons were constant while the fractions of extractable liquids increased with conversion, until all unextractable solids (polymer residue) were consumed. After that time, over-hydrogenolysis caused the volatile fraction to increase dramatically, and the wax fraction to decrease, regardless of Pt nanoparticle size.^131^ The mean chain lengths and carbon number distributions were similar for the waxes generated by all three Pt catalysts over a wide range of conversions, Figure 23e. Therefore, the wax carbon number distribution was deemed to be a consequence of the silica architecture, rather than the Pt nanoparticle size. This type of catalyst architecture represents an interesting strategy to evaluate C–C bond cleavage activity separately from chain length selectivity.

The influence of pore properties was explored by combining *in-situ* MAS NMR with molecular simulations. The majority of the polymer chains underwent Pt-catalyzed dehydrogenation-hydrogenation, rather than C–C bond cleavage. However, longer pores (with longer residence times) did result in an increased ratio of hydrogenolysis to dehydrogenation events.^172^Figure 23f shows the average molecular weight of wax generated from hydrogenolysis of PE (*M*_n_ = 2,800 g/mol, *M*_w_ = 5,000 g/mol) at 300 °C under 8.9 bar H_2_ after 6 h for various X-mSiO_2_/Pt/SiO_2_ catalysts (X denotes the pore length). The wax shifted to a lower average weight and lower dispersity until the values reached a plateau, implying that the product distribution is pore-length-dependent.^171^

In a subsequent design, the “dead-end” pores were replaced by open-channel pores (with Pt nanoparticles located between mSiO_2_ and mesoporous MCM-48).^132^ Small-molecule alkane products were proposed to exit the pores more rapidly in this configuration, resulting in a narrower product distribution. Hydrogenolysis of LDPE (M_n_ = 20,000 g/mol, M_w_ = 91,000 g/mol) at 300 °C under 21 bar H_2_ gave an average carbon number of C_28_ after 12 h, with selectivities of 93 and 1 % to wax products and methane, respectively.

A similar catalyst architecture was constructed with amorphous ZrO_2_ nanoparticles (3.0 ± 0.5 nm) as the hydrogenolysis active sites, instead of Pt nanoparticles. The ZrO_2_ nanoparticles were sandwiched between mesoporous silica platelets (pore size: ca. 3.4 nm, lateral dimensions ranging from hundreds of nm to a few mm, thickness ca. 5 nm). Hydrogenolysis of PE (M_n_ = 20,000 g/mol, *Đ* = 4.8) was conducted at 300 °C under 10 bar H_2_. The activity of zirconia was comparable to that of Pt when the H_2_ consumption rate was normalized by the metal (i.e., Zr or Pt) loading. The hydrocarbon product distribution was centered at C_18_. PE chains were proposed to translocate from the bulk through the radial mesopores of the SiO_2_ platelets and undergo cleavage on the ZrO_2_ nanoparticles, following a “processive” mechanism similar to that proposed for mSiO_2_/Pt/SiO_2_.^131^

Although the above examples illustrate how to construct layered porous architectures starting from the hydrogenolysis active sites (Pt or ZrO_2_ nanoparticles) in a “bottom-up” approach, it is also possible to use a “top-down” approach in which active sites are introduced into a pre-synthesized porous material. Ru@SBA-15 (1.2 wt% Ru, average silica pore size 9.1 nm) was prepared with Ru nanoparticles (ca. 3 nm) located exclusively inside the mesopore channels of the silica, for comparison with Ru/SBA-15 with similar Ru loading but with nanoparticles located exclusively outside the channels.^124^ In the hydrogenolysis of PE (*M*_w_ = 4000 g/mol, *Mn* unspecified) at 230 °C under 20 bar H_2_, Ru@SBA-15 and Ru/SBA-15 gave polymer conversions of 90 and 44 %, respectively, after 5 h. At the same solid conversion (ca. 55 %), the product distributions were identical, showing that Ru confinement in the channels enhanced catalytic activity, but exerted little effect on product selectivity (in contrast to the observations made with the mSiO_2_/Pt/SiO_2_ catalyst).^124^

**Figure 24:**
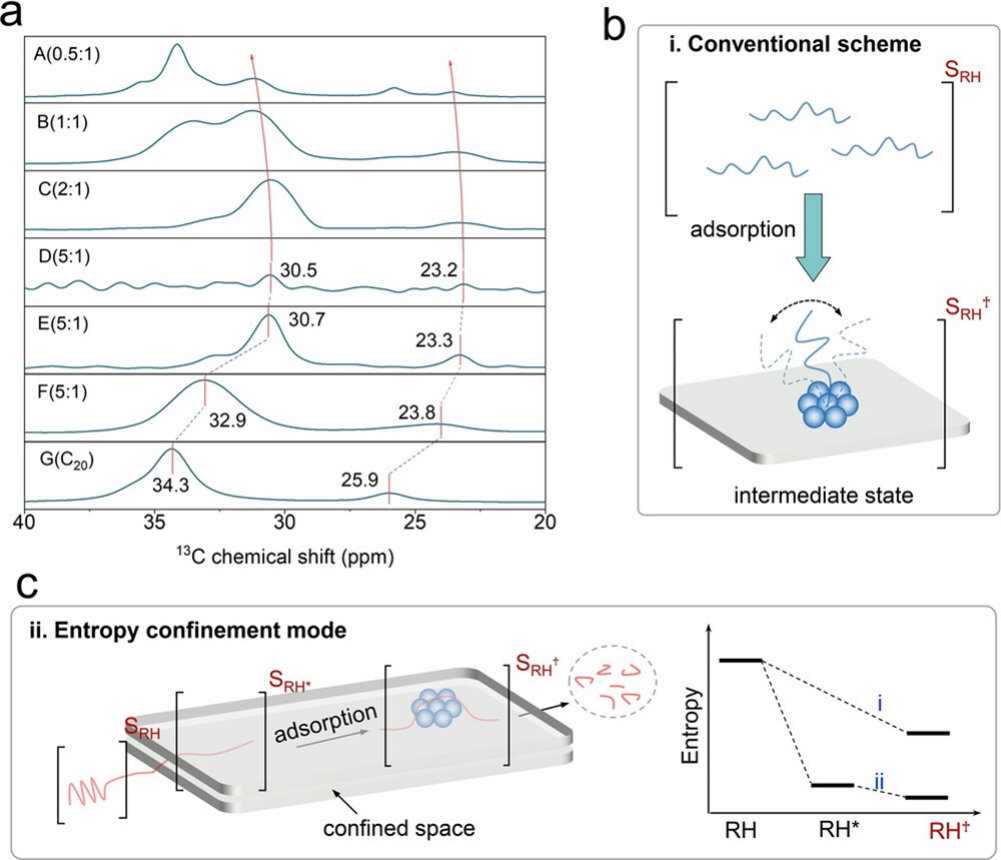
Polymer-support interactions: (a) ^13^C MAS NMR spectra of C_20_ adsorbed in various catalysts (A-D: Ru@SBA-15, with different catalyst/C_20_ ratios as indicated; E: Ru/SBA-15; F: Ru/SiO_2_; G: pure C_20_. Schematic representations of (b) conventional polymer chain adsorption on Ru/SBA-15; and (c) spatial confinement of adsorbed polymer chains and the corresponding entropy reduction for Ru@SBA-15. Reproduced with permission from ref (124). Copyright 2023, Wiley.

Polymer-catalyst interactions were again investigated using ^13^C MAS-NMR spectroscopy, with *n*-eicosane (C_20_) as a probe molecule. Two signals for its methylene groups are shown (G) in Figure 24a. Upon mixing with a catalyst, both signals shift. At the same catalyst/C_20_ ratio, the signal for C_20_ interacting with Ru@SBA-15 (D) has a slightly lower chemical shift and weaker signal intensity than with Ru/SBA-15 (E), indicating more stable adsorbed polymer chains on Ru@SBA-15. C_20_ located in the mesopore channels had a lower entropy than C_20_ adsorbed on a flat surface, Figure 24b,c. According to DFT, forming the transition state results in lower entropy due to chain confinement at the active site. The more that mesopore confinement reduced chain entropy prior to reaction, the less the entropy decreased during reaction. Consequently, confinement of polymer chains reduced the barrier for hydrogenolysis, and improved the activity.^124^ Another study of the hydrogenolysis of PE (*M*_w_ = 4,000 g/mol, *M*_n_ = 1,700 g/mol, 250 °C, 30 bar H_2_) catalyzed by Ru@SBA-15 (ca. 1.4 nm, 5 wt% Ru) attributed the higher reaction rate to improved interaction between polymer chains/intermediates and Ru nanoparticles located inside the SBA-15 channels.^173^

**Figure 25:**
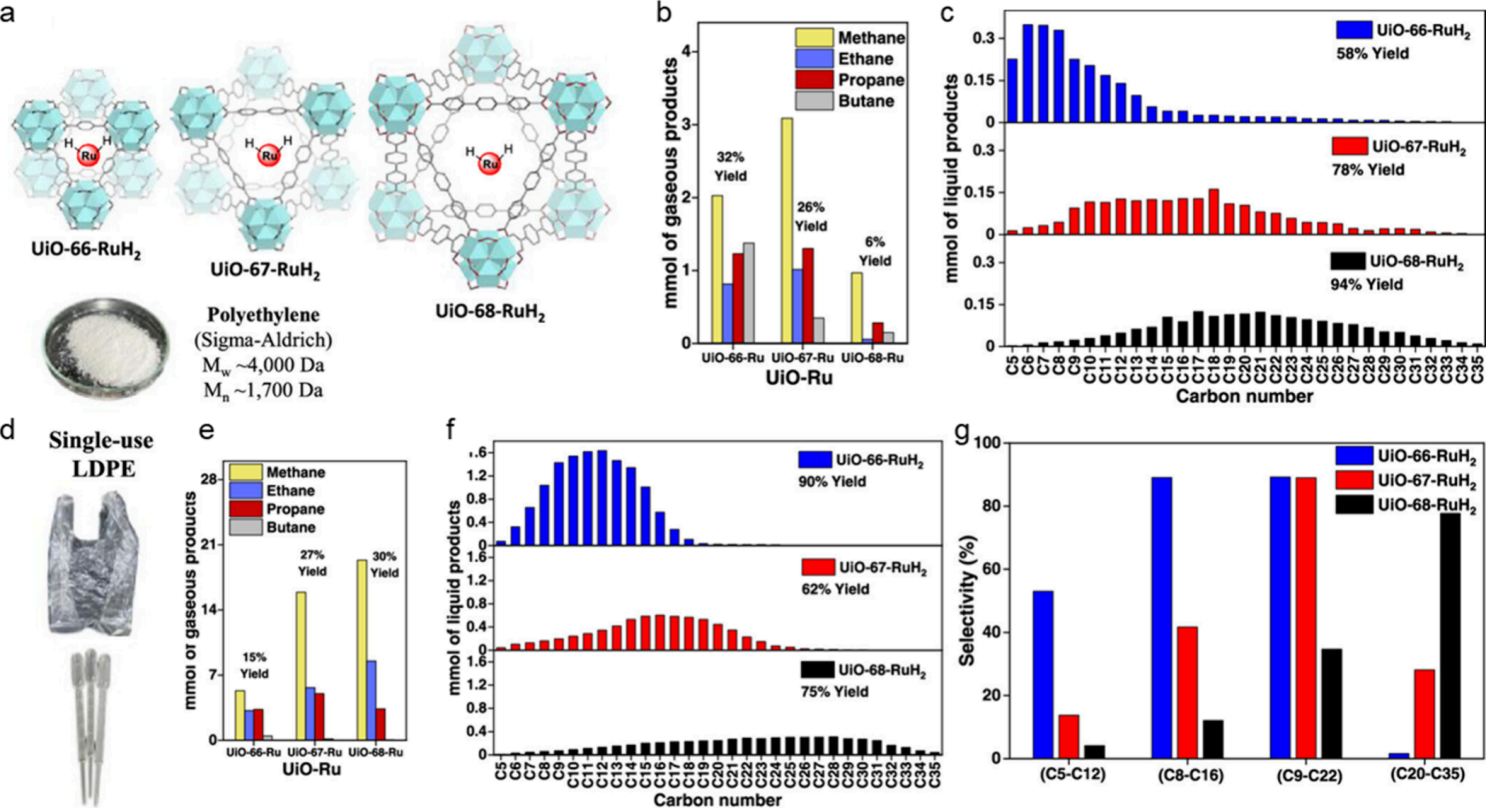
(a) Structures of UiO-6X-RuH_2_ catalysts. Distributions of (b) gas; and (c) liquid products, generated by hydrogenolysis of commercial PE catalyzed by UiO-6X-RuH_2_ catalysts with different pore sizes. Distributions of (e) gas; and (f) liquid products, generated by hydrogenolysis of (d) post-consumer single-use LDPE plastic bags. (g) Selectivity comparison for liquid products in several alkane ranges. Reaction conditions: polymer (600 mg), Ru (4 mmol), 200 °C, 35 bar H_2_, 72 h for commercial PE (*M*_w_ = 4,000 g/mol, *M*_n_ = 1,700 g/mol) and 20 h for the LDPE bag. Reproduced with permission from ref (175). Copyright 2023, American Chemical Society.

Zr-UiO-66 metal–organic frameworks (MOFs) consist of Zr_6_O_4_(OH)_4_ nodes connected by dicarboxylate linkers, with a pore size of 5.1 Å.^174^ Single-site UiO-66-RuCl_2_ was prepared by immobilizing RuCl_3_ in the pores by its reaction with OH groups, then activating with NaEt_3_BH to give neutral, covalently-linked RuH_2_ sites (Figure 25a).^175^ The resulting UiO-66-RuH_2_ (30 wt% Ru) was active in the hydrogenolysis of PE (*M*_w_ = 4,000 g/mol, *M*_n_ = 1,700 g/mol) at 200 °C under 35 bar H_2_. Gas products (C_1_–C_4_) and liquid products (C_5_–C_35_) were formed in 32 and 58 wt% yields (Figure 25b,c), respectively. The pore size was tuned by varying the UiO support (Figure 25a), giving 5.1, 6.8, and 8.1 Å pores for UiO-66-RuH_2_, UiO-67-RuH_2_, and UiO-68-RuH_2_, respectively. Figure 25c shows that the liquid products shifted gradually from a lower and narrower carbon number range (with UiO-66-RuH_2_) to a higher and broader carbon number range (with UiO-68-RuH_2_) as the pore size increased. In particular, the yield of gases declined from 32 to 6 % for the catalyst with the largest pore size. The most abundant C_n_ values in the liquid products were C_5_–C_12_ (66 %), for UiO-66-RuH_2_, C_8_–C_22_ (74 %) for UiO-67-RuH_2_, and C_20_–C_35_ (68 %) for UiO-68-RuH_2_ (Figure 25g).

Similar trends were observed when the same series of catalysts was used to convert post-consumer single-use plastic grocery bags (LDPE, *M*_w_ = ca. 150,000 g/mol, *M*_n_ unspecified, Figure 25d). The average product molecular weight was C_10_ for UiO-66-RuH_2_, C_15_ for UiO-67-RuH_2_, and C_26_ for UiO-67-RuH_2_ (Figure 25e, f). The influence of pore size on product distribution is reminiscent of the “shape-selective catalysis” often invoked with zeolites. While there is no fundamental explanation for this phenomenon in PO hydrogenolysis as yet,^175^ investigating the interactions between molecular alkanes and various pores may shed light on its origin.

#### 2.4.4 Solvent Effects

In addition to efforts to improve intrinsic catalyst activity, process optimization of PO hydrogenolysis is needed to address practical challenges such as high viscosity, contamination, and scale-up. Large PO macromolecules have very high melt viscosities, which vary with molecular weight and branching. Severe mass transport limitations can impede access of the polymer to catalyst active sites.^109^ Vigorous stirring can help to mitigate external mass transport limitations and increase polymer-catalyst contact. This strategy is commonly applied in batch reactors, which represent most of the studies described above (although stirring efficiency is not often verified, and is likely poor in highly viscous polymer melts). Alternatively, the presence of solvent can dramatically improve mass as well as heat transfer. This approach is rarely reported in PO hydrogenolysis, presumably because few solvents are less reactive than PE and PP. However, formation of liquid-range products means that most reactions generate their own, PO-derived solvents as they proceed. The often-unacknowledged consequence is that mass and heat transport limitations vary considerably over the course of the reaction, complicating efforts to measure and compare intrinsic reactivities.

Polymer solubility depends on molecular weight. Even in the absence of solvent, chemically identical polymer chains with very different molecular weights can undergo phase separation. Interfacial adsorption enthalpies and entropies also depend on molecular weight. Thus, the composition of a depolymerizing mixture in a catalyst pore or near a catalytic interface will be non-uniform, and methods are needed to measure and describe these effects. Replica-exchange molecular dynamics simulations predicted that competitive adsorption of short-chain alkanes and long PO chains on Ru will result in a different distribution of three common polymer conformations (trains, loops, and tails), and a preference for the adsorption of longer chains. The simulations suggest that adsorption can be tuned by dissolving the PO in a light alkane solvent.^176^

The effect of *n*-hexane on hydrogenolysis of HDPE (water jug strips) catalyzed by Ru/C (5 wt% Ru) was explored.^125^ At 220 °C under 20 bar H_2_, the polymer was converted to liquids (61 wt% jet-fuel-range, 14 wt % diesel-range) in 1 h. When the temperature exceeded 240 °C, the product distribution shifted abruptly away from shorter chain (C_<23_) hydrocarbons towards longer chains (C_23_–C_38_). This result was attributed to the appearance of supercritical *n*-hexane (critical *T* = 234.5 °C), in which HDPE has very low solubility. Consistent with results in solvent-free systems, H_2_ pressures above 30 bar inhibited hydrogenolysis.

**Figure 26:**
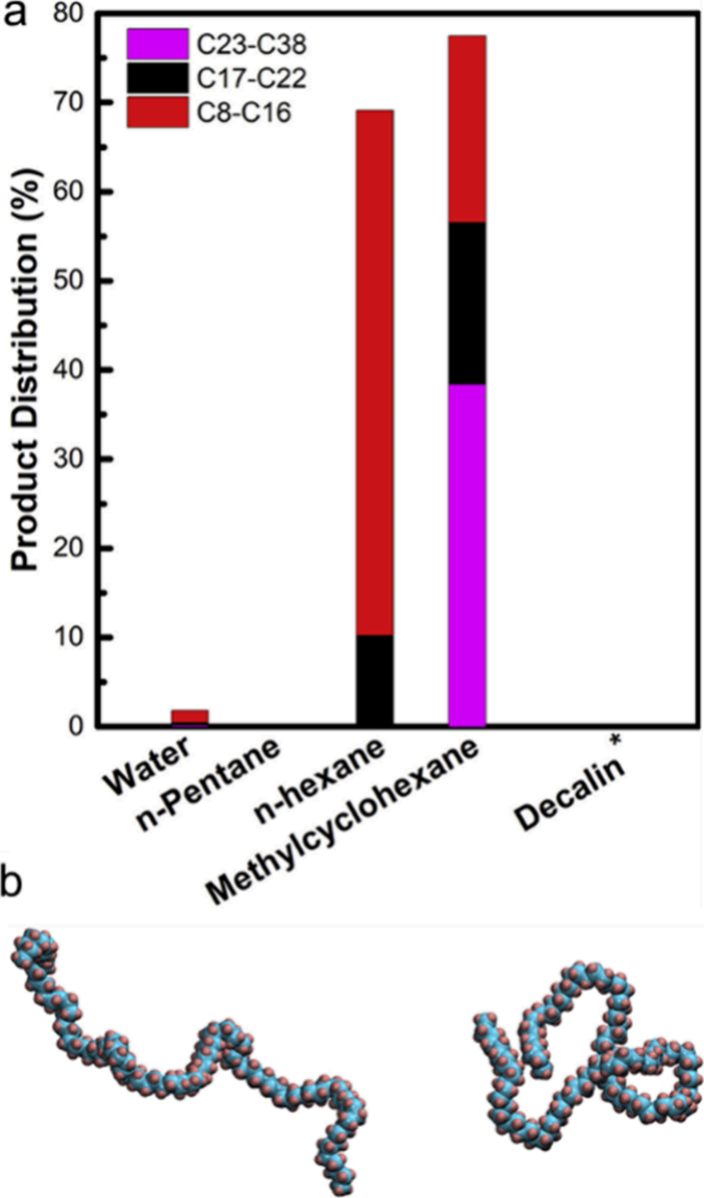
(a) Product yields from HDPE hydrogenolysis in various solvents. Reaction conditions: 0.1 g HDPE (post-consumer water jugs), 25 mL solvent, 220 °C, 20 bar H_2_, 1 h. (b) NVT (substance, volume, and temperature) simulations of PE conformations in decalin (left), and hexane (right). Adapted with permission from ref (125). Copyright 2021, Elsevier.

Three other organic solvents as well as water were explored in HDPE depolymerizations conducted at 220 °C.^125^ Product distributions are shown in Figure 26a. Water was associated with a very low depolymerization rate, since HDPE is insoluble. The optimal performance was obtained in *n*-hexane, while other non-polar solvents gave very different results. For *n*-pentane, whose critical temperature (196.5 °C) is higher than the reaction temperature, little depolymerization took place. Under reaction conditions in water and *n*-pentane, the HDPE melted and became spherical particles, rather than being solvated. In methylcyclohexane, more depolymerization took place although the products contained more of the longer chain (C_23_–C_38_) hydrocarbons than when the reaction was conducted in *n*-hexane.

Curiously, no reaction took place in decalin, although it is considered to be a good solvent for PE. Molecular dynamics (MD) simulations showed that PE adopts an extended conformation in decalin, relative to the compact conformation in *n*-hexane, Figure 26b.^125^ The PE end-to-end length decorrelation rate is inversely correlated with the affinity of PE for the solvent, and PE showed the slowest decorrelation in decalin. The stability of this solvated PE, arising from the increased entropy of the unfolded chains, was suggested to prevent interactions with the catalyst surface and account for the very slow hydrogenolysis kinetics in decalin. This result demonstrates that too strong an affinity of the polymer for the solvent also slows depolymerization.^125^

#### 2.4.5 Reactor Design

The many different kinds of reactors used for PO hydrogenolysis make it difficult to compare intrinsic kinetics, even when the catalysts are similar, due to differences in heat and mass transfer characteristics, temperature profiles, headspace volumes, stirring modes, etc.

**Figure 27:**
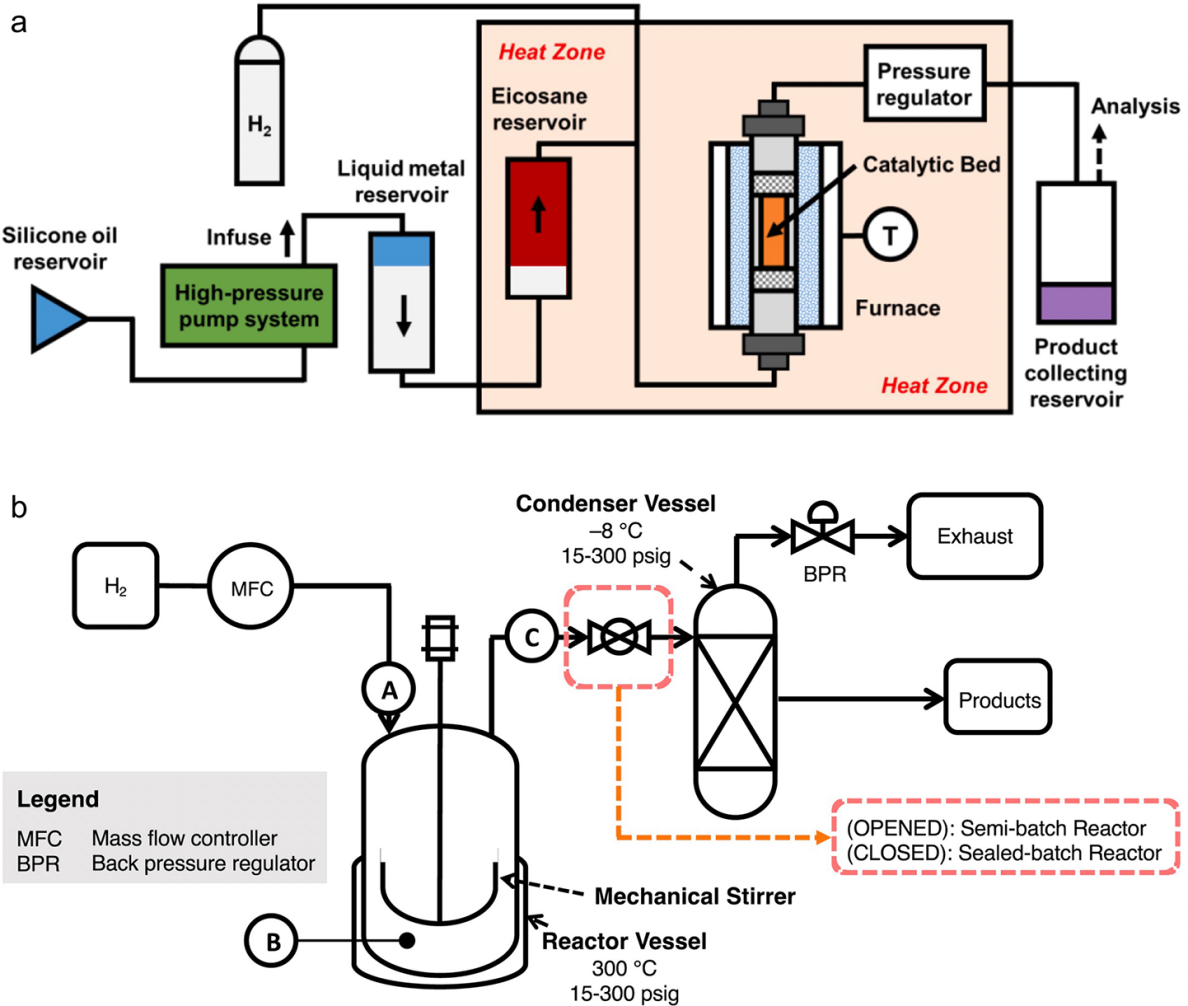
(a) Schematic of the continuous flow reactor system used to study intrinsic PO hydrogenolysis kinetics. Reproduced with permission from ref (89). Copyright 2023 Elsevier. (b) Schematic of reactor configuration for reactive separation during PO hydrogenolysis. Reproduced with permission from ref (177). Copyright 2024, American Chemical Society.

A continuous micro-flow reactor was designed to study the kinetics of PO and model compounds undergoing hydrogenolysis.^89^Figure 27a shows the stainless-steel reactor equipped with a system to feed heavy hydrocarbons or polymer melts to the reactor under pressure. The reactant is pushed into the reactor by an immiscible liquid metal (e.g., GaInSn). This system has several advantages over the batch reactors commonly used to study PO hydrogenolysis, because it provides: (1) steady-state rate measurements; (2) observation of processes occurring at early reaction times; and (3) information about catalyst stability.

PO hydrogenolysis can lead to undesired low-value light gases, including methane. While much effort has been made to control the extent of hydrogenolysis via mechanistic understanding and catalyst design, simple physical removal of middle-range distillates from the reactor may prevent their cleavage to light gases.^178^ A reactive separation by vaporization of volatile liquid products was achieved in the configuration shown in Figure 27b.^177^ A reactor and condenser connected via a stainless-steel tube were held at very different temperatures while H_2_ (5 vol% in Ar, 20.4 bar, 100 mL/min) flowed through the system. After reaction, the products in the reactor and condenser vessels were recovered separately.

The reactor was loaded with 10 g PE (*M*_n_ = 2,800 Da, *M*_w_ = 4,000 Da) and 0.10 g Pt/SiO_2_ (0.085 wt% Pt) and heated to 300 °C, while the condenser was cooled to −8 °C. After 20 h, the liquid products recovered from the condenser (1.6 g, 16 wt% yield) were mostly C_10_–C_20_ hydrocarbons (a mixture of alkanes, alkenes, and aromatics), while the liquid products recovered from the reactor (1.5 g, 15 wt% yield) were mostly C_20_–C_40_. The reactor also contained 6.4 g solid residue (64 wt%). Light gases (C_<9_, 0.6 g, 6 wt% yield) were removed via the exhaust. For comparison, hydrogenolysis without the open connection between the reactor and the condenser (i.e., sealed reactor, with a single charge of H_2_) gave similar low PE conversion (63 wt% solid residue) but a broader range of liquid products (2 g, 20 wt%, C_10_–C_40_) and more gases (1.7 g, 17 wt%). The semibatch configuration gave slightly higher yields of both liquid and wax products and a much lower yield of light gases. The alkene contributions to the liquid products recovered from the condenser, semi-continuous reactor and sealed reactor were 46, 27, and 22 mol%, respectively, indicating that semi-continuous operation prevented alkene hydrogenation.^177^

Although the product selectivity was shifted by ca. 10 wt% towards value-added middle-range liquids by the reactive separation strategy, there are significant limitations to scaling up this approach. The energy required to maintain the temperature difference between the reactor and condenser includes the need to cool the exiting gases, then heat the H_2_ for recycle to the reactor. More energy is required to separate and recompress the unreacted H_2_, which remains 90 % unconverted in the process. While less energy may be required for hydrocarbon product separation relative to the batch process, the overall energy efficiency has yet to be compared for the full processes.

Another approach used a two-stage fixed-bed reactor system,^145^ the first stage being a (hydro)pyrolysis step, with the second being either a hydrogenolysis or hydrocracking step, depending on the catalyst and target products. Primary products (alkane and alkene mixtures) formed in the first reactor were sent via the vapor phase directly to the second reactor.^164^ Compared to direct hydrogenolysis of the polymer, conducted in a single step, the mass transport limitations due to the high viscosity of the polymer melt were eliminated. The non-catalytic pyrolysis step in the first reactor can also be regarded as a pretreatment to remove heteroatom poisons before they reach the catalyst in the second reactor. For example, HCl formed from Cl-containing waste plastics (e.g., PE/PP contaminated with PVC) was trapped by a magnesia-alumina (Mg_3_AlO_4.5_) bed located between the two reactors, generating a Cl-free product suitable for hydrogenolysis in the second reactor with a Cl-sensitive catalyst.^179^

### 2.5 Conclusions and Perspectives

This section reviewed PO hydrogenolysis research involving monofunctional metal or metal hydride catalysts. All require large amounts of H_2_ as an energy-rich coreagent to cleave C–C bonds and shorten polymer chains. The result is an alkane mixture that can be used as a fuel (gasoline, diesel, or jet), as fine chemicals (lubricants, waxes), or as a recycled form of naphtha to produce other feedstocks, including alkenes destined for repolymerization.

Experimental and theoretical studies of small alkane hydrogenolysis catalyzed by metals were summarized to provide a foundation for understanding hydrogenolysis kinetics and mechanisms for polymers. The common elementary steps are alkane dehydrogenation, adsorption of unsaturated hydrocarbons, C–C bond scission, hydrogenation, and alkane desorption. Studies of short-chain alkane hydrogenolysis shed light on possible C–C bond cleavage events in PO macromolecules, where the preference between internal and terminal C–C bond cleavage determines the product molecular weight distribution. The effect of polymer type, branching frequency, stereoregularity, and *P*_H2_ on PO hydrogenolysis were discussed.

Most PO hydrogenolysis catalysts belong to one of four broad categories, for which representative examples and catalyst performance were presented. Among them, Ru-based catalysts show the highest intrinsic activity, but require special effort to control the formation of methane. Pt-based catalysts work at slightly higher temperatures than is typical for Ru-based catalysts, but show better selectivity to hydrocarbons in desirable molecular weight ranges (e.g., fuel or lubricants). First-row transition metal catalysts have attracted some interest because of their lower initial cost, but remain challenged on methane suppression. Early transition metal hydride catalysts operate by a different mechanism than metal catalysts. The key step, β-alkyl elimination, has been described as the microscopic reverse of alkene insertion in coordination polymerization. These emerging catalysts deconstruct polymers at pressures as low as 2 bar H_2_ and temperatures below 200 °C, and can be quite fast when the active sites are cationic.

Finally, catalyst structure-performance relationships in PO hydrogenolysis were reviewed. Reactivity is influenced by the morphology and chemical state of the metal active sites, the choice of support and the metal–support interaction, the presence of promoters, the pore architecture and the ability of various size molecules to access active sites, polymer interactions with the support, solvent effects, reactor configurations, etc. All of these aspects can affect the breadth of the hydrocarbon production distribution and the tendency for methane formation.

Although PO hydrogenolysis has been the subject of perhaps the largest effort in PO upcycling in recent years, there are still many challenges to overcome. The high viscosity of molten macromolecules creates significant barriers to mass transport and polymer–catalyst contact. Experimental test parameters must be varied and reproducibility established before data can be assumed to represent intrinsic catalyst reactivity. In particular, the effect of catalyst particle size and reactor size/stirring should be investigated further. More effort needs to be made to characterize solid residues, which are usually assumed to be unreacted polymer but which may contain chains modified in various ways as well as insoluble non-polymer products. Comparisons of the rate and amount of small molecule hydrocarbon production with the rate and amount of H_2_ uptake are important consistency checks.

Catalyst stability and reusability remain major challenges in the hydrogenolysis of waste plastics, due to contamination by components intrinsic to the plastic (such as additives and fillers), the presence of multiple types and grades of plastics (including those containing Cl and O), and the presence of non-plastic waste which can introduce metals, as well as N, S, etc.). Comparisons of intrinsic catalyst activity or selectivity are therefore insufficient for practical applications, which must consider catalyst sensitivity to impurities. Their removal from plastic waste comes at a cost that increases according the needed level of purity. Impurities can likewise be removed from spent catalysts during regeneration, at a cost that depends on the nature of the deactivation.

Finally, the source of H_2_ is a critical consideration. Green H_2_, not derived from or dependent on fossil fuels, will need to be available at scale, at a reasonable energy cost and with a low CO_2_ footprint to improve the appeal of PO upcycling by hydrogenolysis.

## 3 Catalytic Cracking and Hydrocracking of Polyolefins

### 3.1 Key Concepts

According to the IUPAC Goldbook, cracking refers to thermal or catalytic decomposition of a hydrocarbon into chemical species of lower molecular weight.^53^ Because chemical feedstocks typically have much smaller carbon numbers than polymers, cracking is a strategy to generate recycled chemical feedstocks from POs.

**Scheme 10:**
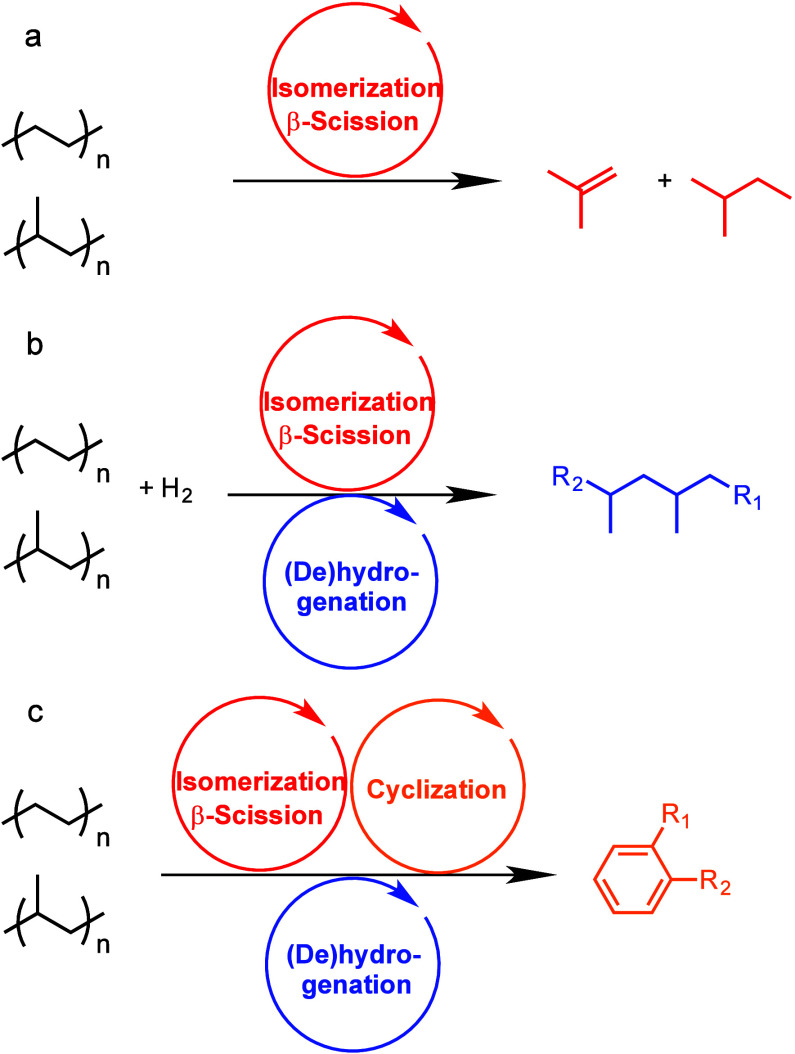
Depiction of Overall Processes for (a) Catalytic Cracking; (b) Hydrocracking; and (c) Hydrogen Redistribution, with their Component Catalytic Reactions

Thermal cracking of POs in the absence of O_2_ at high temperatures (>500 °C), also known as pyrolysis, generates *n*-alkanes, 1-alkenes, and alkadienes.^20^ In the absence of a catalyst, the endothermic reaction is proposed to follow a radical mechanism. Pyrolysis without a catalyst is not the focus here; other reviews have discussed the impact of reaction conditions and reactors on the product distribution and reaction mechanisms, as well as the technical challenges.^20,180−185^ Catalytic cracking shares many similarities with pyrolysis, but differs in the use of a acid catalyst (Scheme 10a).^186^ The catalytic cracking of POs typically requires lower temperatures (but still >400 °C) compared to pyrolysis, and it generates a mixture of *iso*-alkanes, alkenes, cycloalkanes, and aromatics.^187−191^

Hydrocracking is a form of catalytic cracking that occurs in presence of a high pressure of H_2_. In addition to an acid component, the catalyst must contain a component with a (de)hydrogenation function (Scheme 10b).^53^*Iso*-alkanes are formed in competing acid-catalyzed isomerizations. Since hydrocracking is exothermic, it can be performed at even lower temperatures than catalytic cracking. The lower energy requirements and simpler product distributions have attracted interest for polymer upcycling. The following section focuses primarily on the (relatively) low temperature hydrocracking of POs, although some efforts in the area of catalytic cracking will also be discussed.

In addition to catalytic cracking and hydrocracking, POs can be converted, in the absence of external H_2_ or under low *P*_H2_, and in the presence of acidic, and especially bifunctional catalysts, into higher-value aromatics by hydrogen redistribution (Scheme 10c). This strategy will be discussed in section 3.6.

#### 3.1.1 Key Definitions

In *hydrocracking*, the net reaction is *hydrogenolysis*, in which H_2_ is added across a C–C or carbon–heteroatom (C–X) single bond, resulting in its cleavage.^53^ In the literature, *hydrocracking* and *hydrogenolysis* are sometimes used interchangeably to refer to the overall cleavage of C–C single bonds.^69^ However, in hydrocracking, the catalyst is bifunctional (metal/acid) and C–C bond cleavage is usually achieved via an initial dehydrogenation step to form an alkene, whose protonation leads to a highly reactive carbenium ion. Mechanistically, hydrogenolysis refers specifically to the C–C bond cleavage that occurs when an alkane interacts directly with a hydrogen-covered metal surface.^69^ In the following sections, we will adopt the mechanism-based definitions for the hydrocracking and hydrogenolysis of both small molecule alkanes and polyolefins.

#### 3.1.2 Mechanistic Considerations

Because the catalysts used in catalytic cracking and hydrocracking both contain acid sites, the proposed mechanisms exhibit many similarities.

##### 3.1.2.1 Catalytic Cracking Mechanism

Our current understanding of catalytic cracking is described in a comprehensive review.^192^ Briefly, the reaction proceeds in three stages: (1) initiation by formation of a carbenium ion; (2) skeletal rearrangement of the carbenium ion, leading to C–C bond scission that gives an alkene and a new carbenium ion, and hydride abstraction by the carbenium from another alkane; and (3) deprotonation of the carbenium ion to form an alkene.^192^

**Scheme 11:**
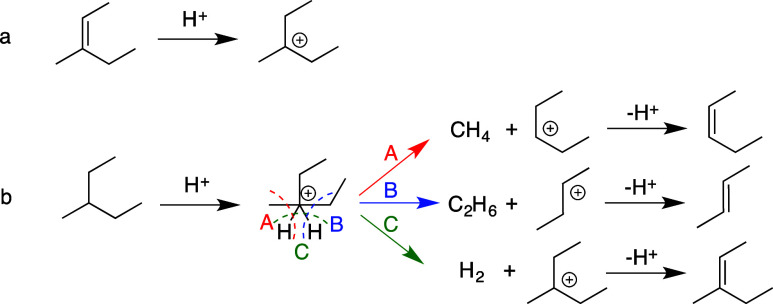
Two Mechanisms for Carbenium Ion Formation Induced by Brønsted Acid Sites: (a) Protonation of 3-Methyl-2-pentene to give a Tri-coordinate Carbenium Ion Directly; and (b) Protolytic Cracking of 3-Methylpentane and *n*-Hexane, via a Penta-coordinate Carbonium Ion^192^

Initiation can be achieved by a Brønsted acid site (BAS) that protonates an alkene to form a tri-coordinate carbenium ion (Scheme 11a). If alkenes are not present, alkane protonation by BAS must initiate the reaction, giving H_2_ and a carbenium ion. Haag and Dessau suggested that alkane cracking involves a penta-coordinate carbonium ion which evolves to a carbenium ion and either an alkane or H_2_ (Scheme 11b). Formation of carbenium ions has also been proposed to occur directly, when a Lewis acid site (LAS) abstracts a hydride from an alkanes.^192,193^

**Scheme 12:**
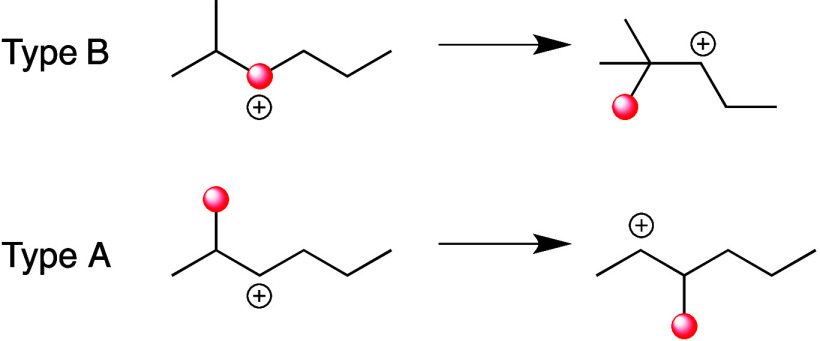
Two Types of Carbenium Ion Isomerization The red circles indicate the carbon atoms that undergo skeletal isomerization.

Carbenium ions can undergo either type A or type B isomerization, Scheme 12. The former, which is faster, involves classical alkyl and hydride shifts and results in changes in the position, but not the number, of branches. The latter causes the number of branches to change and proceeds via protonated cyclopropane intermediates.^192,194^

**Scheme 13:**
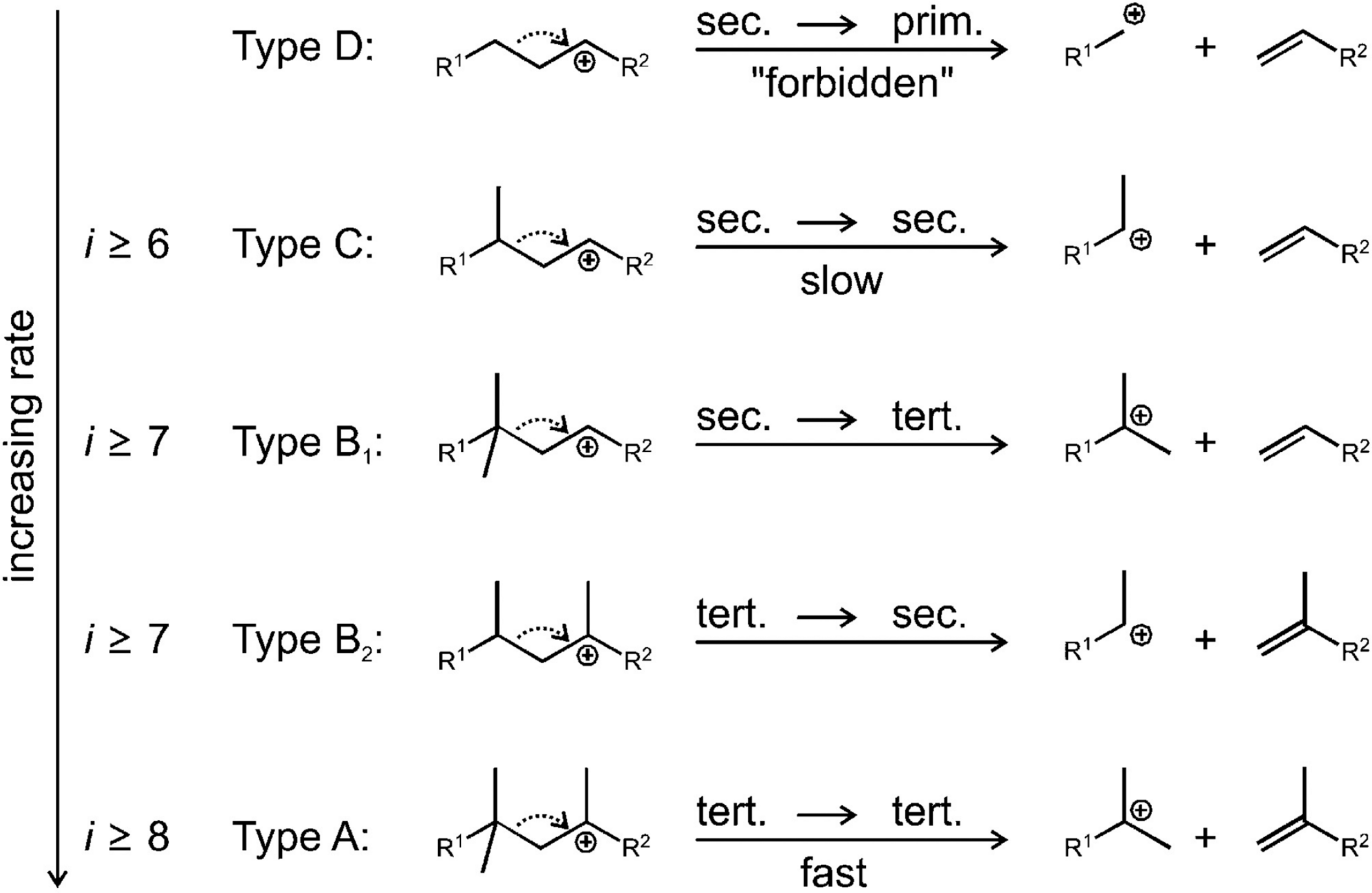
Classification of β-Scission Reactions for Carbenium Ions (the Value of *i* is the Minimum Number of Carbon Atoms Required) Reproduced with permission from ref (69). Copyright 2012, Wiley.

After possibly multiple isomerization events, the carbenium ions undergo various β-scission reactions. They are classified as Types A, B, C, or D, with reaction rates decreasing in this order due to the relative stabilities of the carbenium ions (Scheme 13). Type A β-scission requires a tertiary carbenium ion with three branches in an α,γ,γ-arrangement; heterolytic C–C bond cleavage leads to a disubstituted alkene and a new tertiary carbenium ion. Type B β-scission requires a secondary carbenium ion whose cleavage gives a tertiary carbenium ion (Type B_1_), or vice-versa (Type B_2_). Both B-types require two branches, in either the γ,γ- (for B_1_) or α,γ- (for B_2_) positions. Less common Type C and D scissions involve the reaction or formation of highly unstable primary carbenium ions. In these cases, carbenium ions may rearrange to a configuration more favorable for β-scission faster than they undergo C–C bond cleavage.^69^ While POs have more than enough carbon atoms to undergo any of these scission reactions, their branch frequency and pattern will influence the rates of β-scission under hydrocracking conditions.

Hydride abstraction from an alkane by a carbenium ion may be the dominant pathway for generating carbenium ions on new alkane chains, considering the slow rate of their formation via alkane protonation. When carbenium ions desorb from BAS, they re-form alkenes.^192^ When multiple C–C bond scissions occur on a single alkane chain, the major cracking products are alkenes. However, cyclization and aromatization, via hydrogen transfer, can also be acid-catalyzed.^191^ These mechanisms will be discussed in section 3.6.

##### 3.1.2.2 Bifunctional Hydrocracking Mechanism

**Scheme 14:**
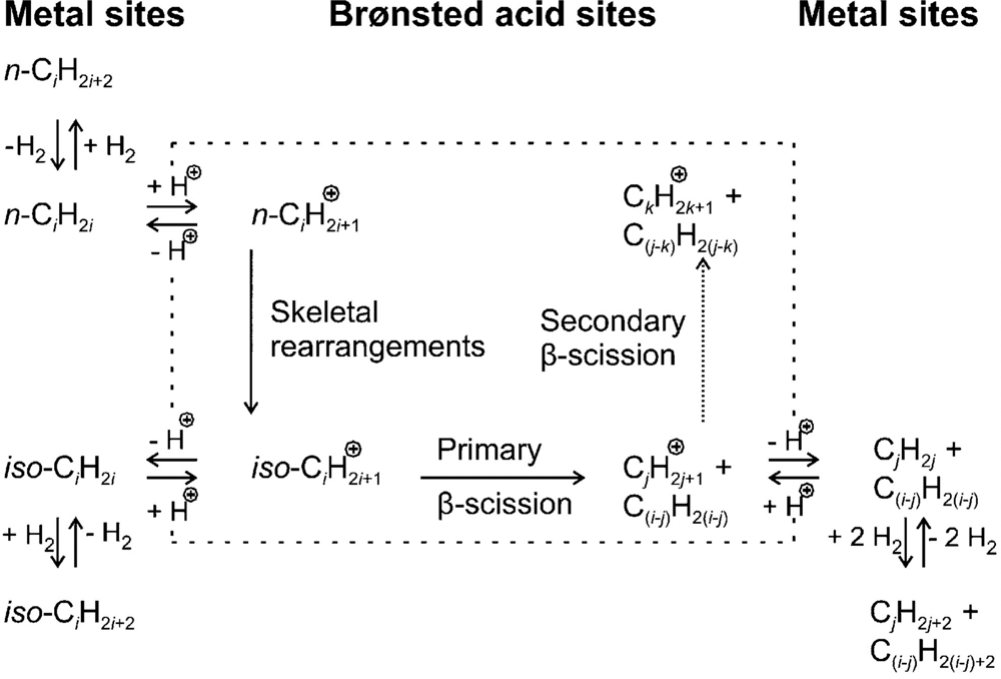
Classical Mechanism for Hydrocracking of *n*-Alkanes on a Bifunctional Catalyst Whose Metal Sites Catalyze Alkane Dehydrogenation/Hydrogenation and Whose Bro̷nsted Acid Sites Catalyze Alkene Skeletal Isomerization, Leading to C–C Bond Cleavage Reproduced with permission from ref (69). Copyright 2012, Wiley.

The classical alkane hydrocracking mechanism is summarized in Scheme 14.^69^ The acid-catalyzed steps are similar to those in catalytic cracking. Alkane hydrocracking involves the following sequence of steps: (1) alkane dehydrogenation on metal sites, leading to release of an alkene; (2) diffusion of the alkene to an adjacent Bro̷nsted acid site, where its protonation yields a highly reactive carbenium ion; (3) skeletal isomerization of the carbenium ion, leading to C–C bond scission that forms an alkene and a new carbenium ion; (4) deprotonation of the carbenium ion to generate a new alkene; and (5) diffusion of both alkenes to metal sites, where they are hydrogenated to the corresponding alkanes.

#### 3.1.3 Metal–Acid Balance (MAB)

##### 3.1.3.1 Influence on Hydrocracking Activity

**Figure 28:**
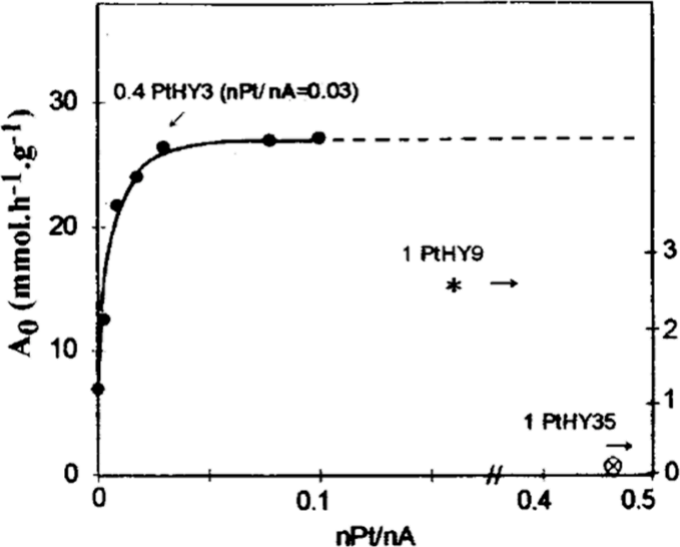
MAB dependence of the initial activity (*A*_0_, normalized by total catalyst mass) for *n*-decane hydrocracking catalyzed by Pt/HY zeolite, at 200 °C. MAB is represented by the ratio of the number of accessible Pt atoms to the number of acid sites (*n*_Pt_/*n*_A_). Data labeled PtHY3 were recorded for a constant number of acid sites. Acidity decreases in the order HY3 > HY9 > HY35. Reproduced with permission from ref (195). Copyright 1996, Elsevier.

According to the bifunctional mechanism outlined above, the overall reaction depends on the ready availability of both metal and acid sites. Bifunctional catalysts are therefore optimized for their metal-to-acid site balance (MAB), by adjusting the *n*_M_/*n*_A_ ratio. Typically, the cracking activity per acid site (turnover frequency, TOF) increases with MAB until it reaches a plateau, Figure 28. In the plateau region, the (de)hydrogenation reactions are quasi-equilibrated on the metal sites, such that the rate is controlled solely by the number of acid sites. This is called the “ideal hydrocracking region”.

##### 3.1.3.2 Influence on Hydrocracking Selectivity

**Figure 29:**
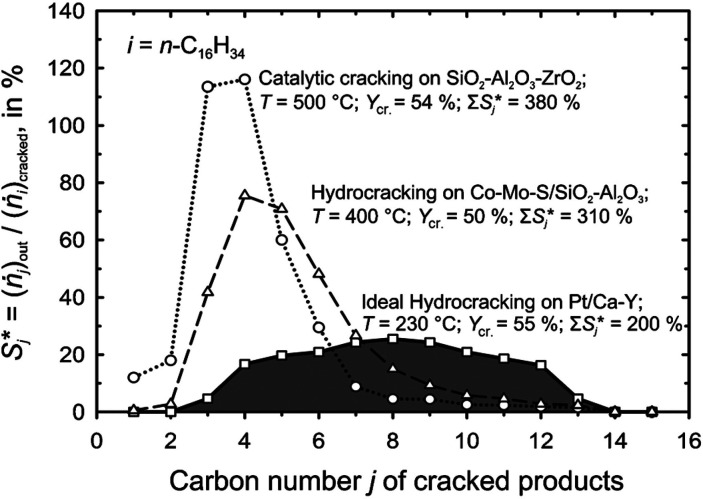
Molar distribution of hydrocarbon products from the catalytic cracking or hydrocracking of *n*-hexadecane. Typical results are shown for catalysts with no hydrogenation component (SiO_2_–Al_2_O_3_–ZrO_2_), a weak hydrogenation component (sulfided Co-Mo-S/SiO_2_-Al_2_O_3_), or a strong hydrogenation component (Pt/Ca-Y zeolite), at similar yields of cracked products (*Y*_cr._ ≈ 50 %). *S*_j_^*^ is the modified cracking selectivity, defined as moles of hydrocarbons with *j* carbon atoms per mol *n*-hexadecane converted. Reproduced with permission from ref (69). Copyright 2012, Wiley.

For longer hydrocarbons (C_*i*_H_2*i*+2_, *i* > 8), MAB also influences the regioselectivity of C–C bond cleavage. Low *n*_Pt_/*n*_A_ ratios result in mostly C_3_–C_6_ products, while high ratios give a random distribution of hydrocarbons across the entire range of carbon numbers, Figure 29.

The difference in regioselectivity is a consequence of the metal-catalyzed dehydrogenation/hydrogenation. A high MAB leads to a high alkene concentration, resulting in competitive adsorption of different alkenes on the acid sites. Consequently, C–C bond scission tends to be random for a high-MAB catalyst, and results in a bell-shaped distribution of hydrocarbon chain lengths. Conversely, a low-MAB catalyst generates more C_3_–C_6_ hydrocarbons because the low alkene concentration allows alkene-acid site interactions to persist, leading to consecutive C–C bond scission events on the same hydrocarbon chain.

#### 3.1.4 Influence of Hydrocarbon Chain Length

Since POs such as PE and PP consist almost entirely of C–C and C–H bonds, they are chemically equivalent to alkanes. In PO hydrocracking, the first-formed hydrocarbon fragments undergo further reaction, making the final product distribution a result of both intra- and inter-molecular selectivities. The intra-molecular selectivity usually corresponds to random chain scission, provided the catalyst satisfies the ideal hydrocracking criterion (as described in section 3.1.3.2). Consequently, the product distribution is determined by the inter-molecular selectivity, reflected in chain length-dependent reactivity.

Reactivity differences in the hydrocracking of hydrocarbons of different chain lengths have been explored in the range of carbon numbers from C_6_ to C_44_. Many studies have reported that the rate of alkane hydrocracking increases with carbon number from C_6_ to C_11_.^196,197^ Longer chains require lower temperatures than shorter chains to reach similar degrees of conversion in a given reaction time.^197^ Reactivity differences are also manifested in the selective hydrocracking of longer chains within an alkane mixture. Two examples involve the conversion in an equimolar mixture of *n*-C_7_H_16_ and *n*-C_10_H_22_ catalyzed by Pt/US-Y,^198^ and the conversion of an equimolar mixture of *n*-C_10_H_22_ and *n*-C_12_H_26_ catalyzed by Pd/La-Y.^199^ In both cases, the rate for the heavier alkane was not affected by the presence of the lighter alkane, while the rate for the lighter alkane was considerably lower compared to the reaction of that component alone, under the same reaction conditions.

**Figure 30:**
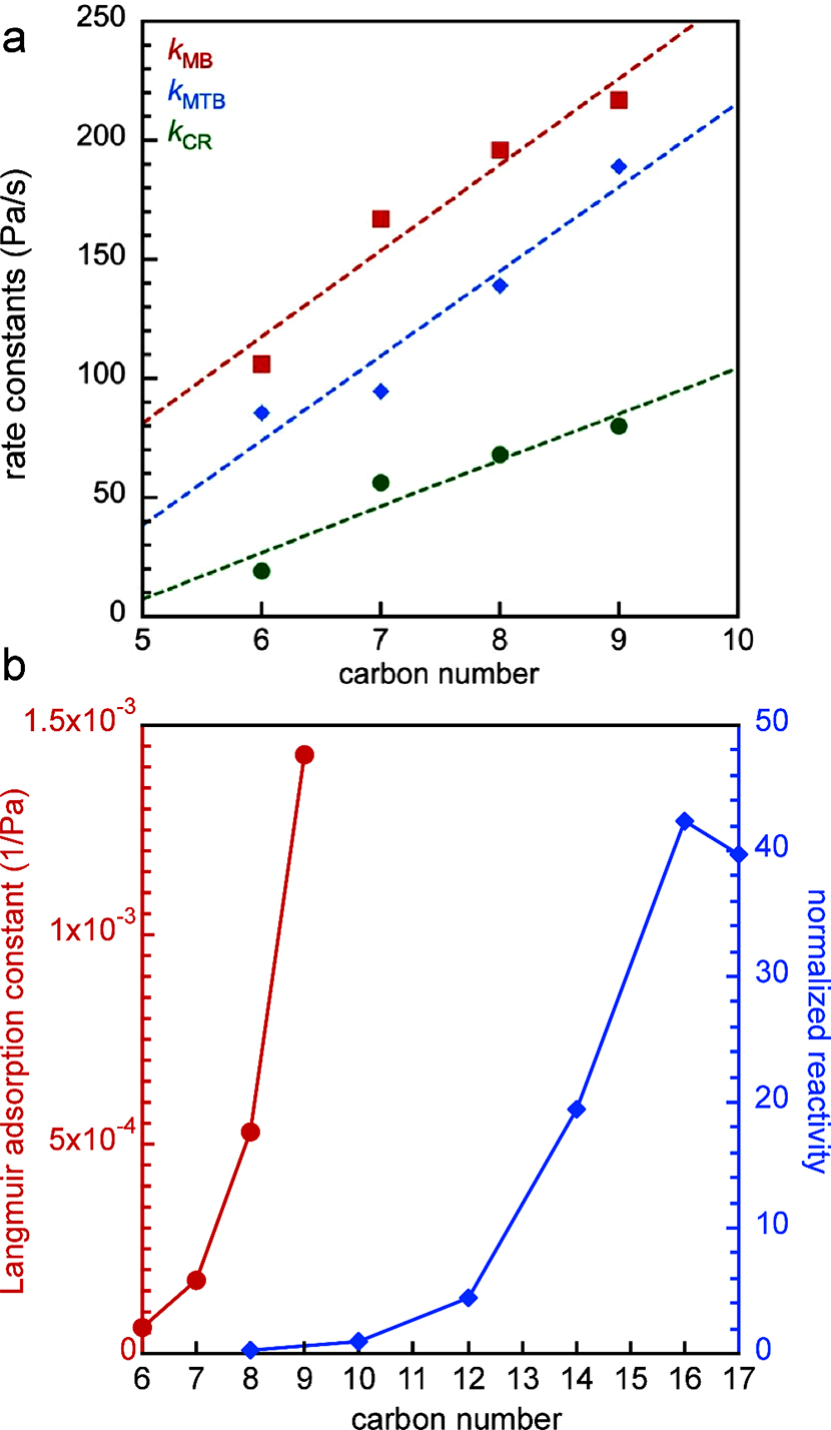
(a) Linear dependence of the rate constants for the conversion of *n*-alkanes catalyzed by Pt/H-Y at 233 °C on the hydrocarbon chain length: *k*_MB_, *k*_MTB_, and *k*_CR_ are rate constants for the conversion of linear alkanes to mono-branched alkanes, mono-branched to multi-branched alkanes, and multibranched to cracked alkanes, respectively. Adapted with permission from ref (196). Copyright 1997, American Chemical Society. (b) Comparison of *n*-alkane chain length dependence of Langmuir adsorption constants (red circles) on Pt/H-Y (adapted with permission from ref (196), copyright 1997, American Chemical Society), with activities for the catalytic cracking of *n*-alkanes (blue diamonds) over FSS-1 at 350 °C, normalized to the reactivity of 2-methylnonane (adapted with permission from ref (201), copyright 1990, Elsevier).

While the hydrocracking rate generally increases with carbon number, two different relationships have been reported. The first is exemplified by the observation that the rates of hydroisomerization and hydrocracking catalyzed by Pt/Ca-Y increased linearly with carbon number from *n*-C_6_H_14_ to *n*-C_11_H_24_.^197^ Similarly, apparent rates for hydroisomerization and hydrocracking catalyzed by Pt/HY increased linearly with carbon number for hydrocarbons from *n*-C_6_H_14_ to *n*-C_9_H_20_ (Figure 30a).^196^ The effect could be due to the increase in the number of secondary carbons.^200^

A second type of relationship was reported in the cracking of alkanes catalyzed by FSS-1 (Filtrol catalyst, containing rare earth-exchanged Y zeolite),^201^ where the reactivity doubled with each additional carbon present in the molecule, resembling the exponential increase in Langmuir adsorption constants with hydrocarbon chain length (Figure 30b).^196^ Interestingly, the catalytic cracking activity towards *n*-alkanes leveled off at C_17_, similar to the observation for a rare-earth-exchanged crystalline aluminosilicate catalyst at 480 °C.^202^

**Figure 31:**
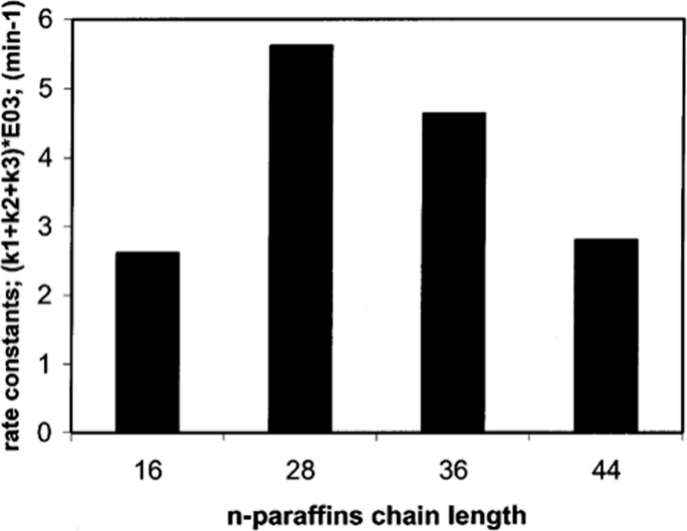
Effect of chain length on lumped rate constants for hydrocracking of *n*-alkanes, catalyzed by Pt/amorphous silica-alumina (0.3 wt% Pt) at 380 °C, where *k*_1_, *k*_2_, and *k*_3_ are pseudo-first-order rate constants for the isomerization of *n*-alkanes to mono-branched isomers, isomerization of *n*-alkanes to multi-branched isomers, and hydrocracking, respectively. Reproduced with permission from ref (203). Copyright 2004, American Chemical Society.

The effect of chain length on the hydrocracking of longer alkanes catalyzed by Pt/amorphous silica-alumina (0.3 wt% Pt) was studied.^203^ The reactivity was lower for *n*-C_16_H_34_ than for *n*-C_28_H_58_, then decreased for *n*-C_36_H_74_ and *n*-C_44_H_90_ (Figure 31). The lower reactivities of the heavier alkanes might be a consequence of individual *n*-C_36_H_74_ and *n*-C_44_H_90_ chains occupying more of the accessible catalyst surface area.

In conclusion, the intrinsic rate of *n*-alkane hydrocracking increases with carbon number for small to moderate carbon numbers, although the relationship may not be linear in all systems. Furthermore, when the carbon chains are longer, differences in their ability to contact the metal and/or the acid sites may alter observed trends. These issues are obviously highly relevant to PO hydrocracking.

### 3.2 Examples of Polyolefin Catalytic Cracking and Hydrocracking

#### 3.2.1 Reports at Elevated Temperatures

##### 3.2.1.1 Catalytic Cracking at Elevated Temperatures

The first report of catalytic cracking of a PO by a solid acid dates to the 1980s.^204^ Using silica-alumina as the catalyst at 480 °C, PP was converted predominantly to alkenes (44 wt%), *iso*-alkanes (35 wt%), and aromatics (11 wt%). Since then, many such studies have appeared. Several reviews have summarized the effect of catalyst variables (acidity, pore structure), reaction conditions, reactor type, reaction kinetics, and mechanisms.^35,187,191,205^ Nevertheless, some of these topics are mentioned below for comparison purposes. In the 1990s, the use of the mixed salt MgCl_2_/AlCl_3_ was reported in the catalytic cracking of PE at 370 °C, giving mostly gases (88 wt%) as products.^206^ Examples of the use of other salts, as well as chloroaluminate ionic liquids, have since been summarized in a review.^191^ More recently, lower temperatures have been used in the catalytic cracking of POs to target more valuable products, as discussed in more detail below.

##### 3.2.1.2 Hydrocracking at Elevated Temperatures

Hydrocracking of POs first began to attract attention in the 1990s. In contrast to pyrolysis^180^ and catalytic cracking^207^ approaches, which are typically conducted at temperatures above 400 °C and produce complex hydrocarbon mixtures including alkenes, aromatics, and alkanes, PO hydrocracking is typically performed at lower temperatures and generates saturated hydrocarbon liquids. These liquids can be used directly as transportation fuels or as fuel oil. A review described the effects of variables such as temperature, H_2_ pressure, reaction time, presence and type of catalyst, and the nature of the plastic feed on the hydrocracking of both virgin and waste plastic materials.^208^

In early studies, PO hydrocracking was generally conducted at 350–450 °C with 20–150 bar H_2_ (pressure measured at room temperature), similar to the hydrocracking of light alkanes. The hydrocracking catalysts were similar too: metal nanoparticles dispersed on an acidic support. The supports were often amorphous oxides such as silica-alumina, crystalline zeolites such as HZSM-5, or acid-modified oxides such as sulfated zirconia. The hydrogenating component was a noble metal (Pd or Pt), or a non-noble metal from group 6 (Mo or W), group 9 (Co), or group 10 (Ni). Intrinsic catalytic activity was not necessarily apparent at the high reaction temperatures, where mass transport can limit the overall reaction rate. Furthermore, the catalysts experienced significant coke build-up, leading to rapid deactivation.^51^ Therefore, more recent studies have focused on reactions conducted at lower temperatures.

#### 3.2.2 Reports Under Milder Reaction Conditions

##### 3.2.2.1 Catalytic Cracking under Milder Conditions

Recent studies have explored catalytic cracking at lower temperatures. Metal-free H-MFI zeolite (0.4 g, Si/Al = 40) was reported to crack PE (1 g, *M*_w_ = 4,000 g/mol, *Đ* = 1.9) to C_3_–C_7_ (37 wt%, mostly *n*-alkanes, *iso*-alkanes, *iso*-alkene, alkenes) in 17 h at 200 °C.^45^ More than 90 wt% of the polymer was converted, as judged by the mass of residual solid hydrocarbon. However, the mass balance was poor: only 50 wt% of the total mass was recovered.

Selectivity for desired carbon numbers and hydrocarbon product types can be controlled by catalyst design. ZSM-5 nanosheets (0.1 g, Si/Al = 21) were reported to convert PE (0.5 g, described as having an average molecular weight of 150,000 g/mol without specifying *M*_n_, *M*_w_, or *Đ*) in a quartz tube at 280 °C under flowing H_2_ into C_1_–C_7_ hydrocarbons (75 wt%).^209^ Among these products, the majority (84 wt%) were C_3_–C_6_ alkenes. The amount of coke was undetectable.

Catalytic cracking can be coupled with other reactions to make desired products selectively at even lower temperatures. In a chloroaluminate ionic liquid [C_4_Py]Cl–AlCl_3_ ([C_4_Py]Cl:AlCl_3_ molar ratio 1:2) with *tert*-butyl chloride as initiator, dissolved in CH_2_Cl_2_, a low molecular-weight PE (0.2 g, *M*_w_ = 4,000 g/mol, *Đ* = 1.9) was fully converted to liquid *iso*-alkanes (C_4_–C_30_, ∼200 wt% relative to starting PE) by its reaction with isopentane (0.8 g) at 70 °C.^210^

##### 3.2.2.2 Hydrocracking under Milder Conditions

As polymer upcycling has attracted more interest in recent years, researchers have started to investigate hydrocracking at even lower temperatures (200–300 °C), and with more advanced characterization of the catalysts and reaction products. The current state-of-the-art for an efficient bifunctional catalyst or mixture of catalysts involves converting a PO (at ca. 10× the total catalyst mass) into mostly *iso*-alkanes (C_4_–C_30_) within several hours at ca. 250 °C under ca. 20 bar H_2_.

For example, the hydrocracking of LDPE (*M*_w_ = 150,000 g mol^–1^, *Đ* not specified) was reported to be catalyzed by either Pt/USY or Pt/HBeta (10 wt% relative to LDPE), at 275–330 °C under 20 bar H_2_.^211^ C_4_–C_6_*iso*-alkanes were formed in high yields within 1 h, resulting in >95 wt% of the polymer being recovered as either gas- or liquid-phase hydrocarbons. At a similar temperature (250 °C) and H_2_ pressure (30 bar), the conversion of LDPE (2.0 g, *M*_w_ = 250,000 g mol^-1^, *Đ* not specified) was catalyzed by a physical mixture of Pt/WO_3_/ZrO_2_ (0.1 g) and HY zeolite (0.1 g) to predominantly *iso*-alkanes (85 wt%) within 2 h. The products were alkanes in the gasoline (C_5_–C_12_) and diesel (C_9_-C_22_) ranges.^212^

Highly dispersed MoS_x_-HBeta (0.1 g) was investigated as a noble metal-free catalyst to hydrocrack various POs.^213^ After 16 h at 180 °C with a low molecular weight PE wax (*M*_w_ = 3,000 g mol^–1^, *Đ* not specified), a catalyst loading of 20 wt% relative to polymer, and 25 bar H_2_, branched alkanes (C_4_–C_12_) were recovered in 96 wt% yield. At 250 °C under 20 bar H_2_, the reactions of HDPE, LLDPE, LDPE, and PP (1 g, all with *M*_w_ = 300,000 g mol^–1^, *Đ* not specified) gave branched liquid alkanes (mostly C_4_–C_16_, isomers not specified) in >85 wt% yield after 6 h.

PP hydrocracking was reported to be catalyzed by Ru, Ni, or Pt nanoparticles supported on anatase prepared by a sol–gel route (Ru, Ni, or Pt/TiO_2_-A-SG, 0.07 metal/nm^2^).^48^ At 260 °C, the Ru/TiO_2_-A-SG catalyst converted iPP (*M*_w_ = ca. 250,000 g/mol, *Đ* = 3.7) ca. 3× faster than Ru/CeO_2_, when normalized by surface Ru sites. In addition, Ru/TiO_2_-A-SG showed lower selectivity towards undesired CH_4_ (*S*_CH4_ = 3 wt%) compared to Ru/CeO_2_ (21 wt%), slightly higher selectivity towards desired liquid products (C_6–40_, *S*_liquid_ = 78 wt%) compared to Ru/CeO_2_ (72 wt%), and higher isomerization activity (62 % of C_10_ products are *iso*-alkanes), compared to Ru/CeO_2_ (40 %).

### 3.3 Catalyst Design Considerations

Due to their strong similarities in mechanisms and catalysts, catalytic cracking and hydrocracking will be considered together in the following discussion.

#### 3.3.1 Protonation of Polyolefins

Acid sites are key in both catalytic cracking and hydrocracking. Two types of sites typically co-exist in solid acids: Lewis acid sites (LAS) and Brønsted acid sites (BAS). The latter are more commonly invoked as the active sites promoting catalytic cracking of POs, and the observation of CH_4_ and C_2_H_6_ during PO catalytic cracking indicates a carbonium ion mechanism initiated by BAS (see section 3.1.2.1).^45^ The LDPE cracking activity of zeolite catalysts is linearly related to their BAS density, with no relationship to their LAS density.^214^ A similar dependence of the rate of PO catalytic cracking on BAS density was also reported for Al-modified SBA-15,^215^ SiO_2_-Al_2_O_3_,^216^ and ZSM-5.^45,209^ These findings are well-aligned with both experimental^217^ and theoretical studies^218^ of catalytic cracking involving light hydrocarbons.

Hydrocracking of PO model compounds, as well as POs themselves, is therefore expected to be catalyzed by BAS. Conversion of squalane (a methyl-branched C_30_H_62_ isomer) was reported to decrease when the Brønsted acidity of HBeta zeolite was reduced by a dealumination pretreatment.^211^ The amount of residual solid (described as coke, based on TGA analysis of the temperature required for its removal) was also positively correlated with catalyst acidity (unfortunately, coke yields were not compared at constant conversion).

Various ways of modifying Brønsted acidity have been explored in attempts to enhance PO deconstruction. The Brønsted acidity of HY (varied by changing the Si/Al ratio) was reported to correlate with LDPE hydrocracking activity, as judged by the C_1_–C_16_ yield.^212^ The importance of acid sites was further evidenced by conducting the reaction in the presence of adsorbed pyridine, which reduced the catalytic activity significantly.

A series of Pt/WO_3_/ZrO_2_ catalysts was synthesized with systematically varied Pt loadings (*x* = 0.1–1.0) wt%) and WO_3_ loadings (*y* = 5–25 wt%), to give materials denoted *x*Pt–*y*WZr.^219^ According to IR analysis of adsorbed pyridine, the number of BAS increased with WO_3_ loading from 5 to 15 wt%, then decreased as the loading increased further to 25 wt%. Raman spectra confirmed that the majority W sites shifted from monotungstates to polytungstates, and eventually to bulk WO_3_ crystallites, consistent with other reports.^220,221^ Also consistent with previous work,^220^ the intermediate-size polytungstates contributed the most to the number of BAS. Nevertheless, catalysts with the highest WO_3_ loadings produced the most low-molecular-weight alkanes (C_1_-C_35_, all classified as “extractable”), and lowered the melting temperature of the residual (i.e., non-extractable) solid. Thus, although the authors attributed differences in reactivity to changes in BAS density, the relationship is clearly complex and non-linear. For example, 0.5Pt–25WZr and 0.5Pt–15WZr gave similar yields of extractables and non-extractable solids, even though their BAS densities were very different.

IR spectra of adsorbed CO and pyridine were recorded to probe the Lewis and Brønsted acidity, respectively, of Ni/TiO_2_-A-SG (where TiO_2_-A-SG refers to anatase prepared by a sol–gel route).^48^ The catalyst contained both BAS and LAS, although LAS were significantly more abundant. Calcination at 500 °C completely suppressed activity toward PP hydrocracking. Water adsorption at room temperature was not effective in restoring the activity, however, activity was mostly (90 %) recovered by hydrothermal treatment at 130 °C. The elimination and recovery of hydrocracking activity coincided with the removal and replenishment of Brønsted-acidic OH groups, but not with changes in the LAS (which were barely affected, as evidenced by the IR of adsorbed CO). These results strongly suggest that BAS were indeed the active sites for PP hydrocracking, despite their low abundance relative to LAS.

The incorporation of Ce (5 wt%) into Pt/HY zeolite (Si/Al = 4) increased the LDPE conversion (as defined in eq 2) from 40 to 100 % after 2 h at 300 °C under 30 bar H_2_.^222^ XAFS analysis was consistent with atomically-dispersed Ce^4+^ coordinated to 6 framework oxygens in the HY supercage. The presence of Ce was deemed to enhance the strong Brønsted acidity of the zeolite and, thereby, to increase the hydrocracking activity.^223^

**Scheme 15:**
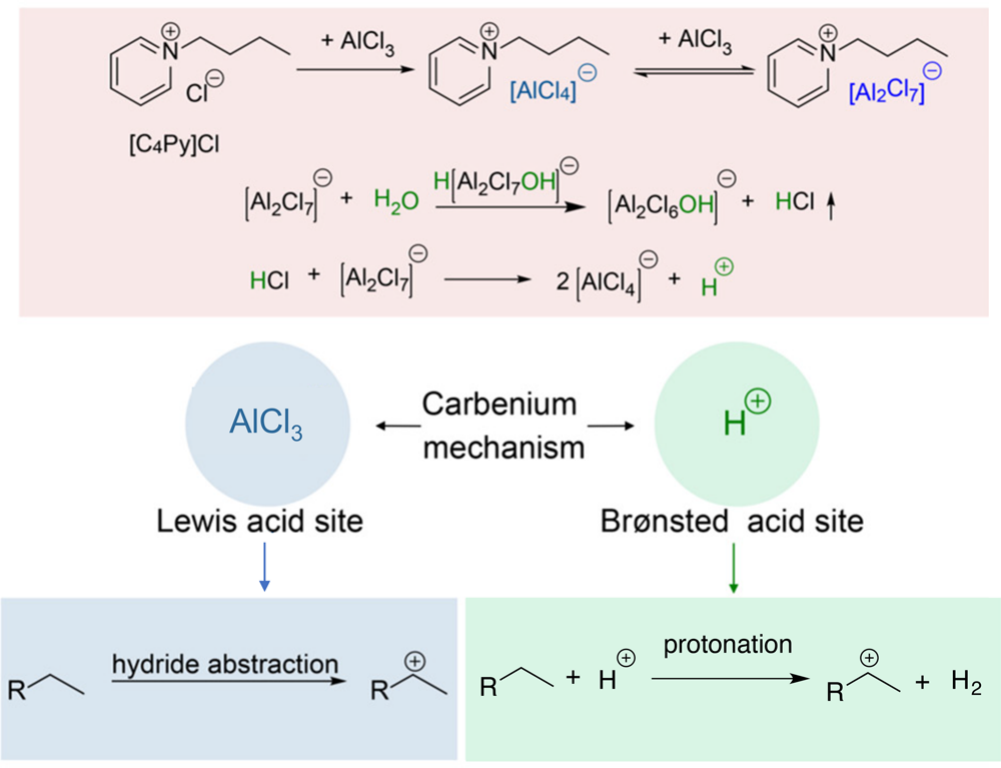
Possible Carbenium Ion Generation in [*n*C_4_Py]Cl-AlCl_3_ Adapted with permission from ref (210). Copyright 2023, AAAS.

Although BAS are generally accepted to be the sites responsible for forming carbenium ions from alkenes and alkanes, LAS are also reported to induce their formation from alkanes,^192,224^ and more readily from halogenated alkanes, as seen in reactions like Friedel–Crafts alkylation.^225^ The upcycling of LDPE or PP to liquid alkanes via tandem cracking-alkylation was reported, initiated by [C_4_Py]Cl–AlCl_3_ ([C_4_Py]Cl:AlCl_3_ molar ratio 1:2, where [C_4_Py]Cl is *n*-butylpyridinium chloride) dissolved in CH_2_Cl_2_ at low temperatures (30 to 70 °C) (for reaction details, see section 3.4.1).^210^ Lewis acidic AlCl_3_ (in equilibrium with [Al_2_Cl_7_]^−^) was assumed to initiate the reaction (Scheme 15). However, [C_4_Py]Cl–AlCl_3_ can react with moisture to form HCl, and may also generate super-acidic protons,^226^ suggesting a possible role for Brønsted acidity as well (Scheme 15).

The roles of various components were explored to identify the species responsible for initiating PO chain scission. The protic molecules H_2_O and CF_3_COOH were found to increase the conversion (defined as in eq 2) achieved in a specified time by ca. 10 %. Reaction of each molecule with one LAS generates one BAS. These experiments suggest that LAS and BAS are similarly efficient in generating carbocations from alkanes at low temperatures. However, addition of *tert*-butyl chloride significantly enhanced the conversion, presumably by reaction with AlCl_3_ to generate a *tert*-butyl cation that abstracts hydride from the PO. The reactions were described as “catalyzed” by LAS in the presence of *tert*-butyl chloride, although the LAS may be more accurately described as an initiator.

In a follow-up study, various metal chlorides and triflates (1 mmol) were explored in the tandem cracking-alkylation of LDPE (0.200 g) with *iso*-pentane (0.800 g) in CH_2_Cl_2_ (3 mL) at 70 °C.^227^ AlCl_3_ and GaCl_3_ were found to be the most active Lewis acids, achieving nearly quantitative conversion of LDPE with high selectivity to gasoline-range liquid alkanes (>70 wt%). Correlations were attempted with three Lewis acidity scales: the Gutmann acceptor number, the fluoride affinity, and the global electrophilicity index. However, in each case, the initial rates of LDPE conversion (defined as in eq 2) were uncorrelated with Lewis acidity.

The superior performances of AlCl_3_ and GaCl_3_ were attributed to fast carbenium ion formation by chloride abstraction from CH_2_Cl_2_, and more importantly, fast hydride transfer. Nevertheless, the key parameter controlling the rate of tandem cracking-alkylation rate is not clear.

Overall, BAS appear to play a more significant role than LAS in generating carbocations from alkanes and alkenes. On the other hand, LAS can potentially generate carbenium ions from other reagents, such as halogenated alkanes. LAS were proposed to enhance the acidity of nearby BAS and/or to promote alkane dehydrogenation to alkenes, thereby indirectly influencing the catalytic cracking reactivity.^205^ Acid sites also catalyze competing reactions such as alkene oligomerization,^228^ alkene cyclization,^229^ alkene aromatization,^230^ and the alkylation of aromatics by alkenes.^231^

#### 3.3.2 Polyolefin Dehydrogenation

As mentioned in section 3.1.2.2, the function of the metal catalyst component in hydrocarbon transformations is (de)hydrogenation. For example, *operando* transmission IR spectra showed that Pt/WO_3_ generates more C=C bonds during HDPE hydrocracking under 30 bar H_2_ at 250 °C, relative to WO_3_ alone.^232^ However, compared with catalytic cracking (where the catalyst has only acid sites), bifunctional catalysts typically produce mostly saturated alkanes since the metal also rehydrogenates alkenes.^208^ PO conversion is typically higher in hydrocracking compared with catalytic cracking, because making alkanes is thermodynamically more favorable relative to making alkenes.

Catalysts for low-temperature hydrocracking (typically conducted at 200 to 300 °C) often have a Pt component that catalyzes fast (de)hydrogenation^233^ and is resistant to heteroatom impurities in the feed.^234^ Combining Pt with other metal promoters, such as Sn or Ga as in the bimetallic catalysts used industrially for propane dehydrogenation,^235^ is an effective approach for improving catalyst stability.^236^ The metalloid contributes both a “geometric effect”, reducing the size of Pt ensembles, and an “electronic effect”, via electron transfer to Pt. Both effects weaken the adsorption of carbonaceous species.^236^

The ability of PtSn/SiO_2_–Al_2_O_3_ (1.0 wt% Pt, 1.8 wt% Sn) to convert HDPE was explored.^237^ The catalyst was synthesized by co-impregnation of SiO_2_–Al_2_O_3_ (Si/Al ratio unspecified) with H_2_PtCl_6_ and SnCl_2_, followed by calcination and reduction in H_2_ to give PtSn alloy nanoparticles, as evidenced by powder XRD. Compared to Pt/SiO_2_–Al_2_O_3_ (0.2 g, 1.0 wt% Pt), PtSn/SiO_2_–Al_2_O_3_ gave a higher conversion of HDPE (2.0 g), 80 vs. 64 wt% in 2 h at 270 °C under 30 bar H_2_. Conversion was even higher, 97 wt%, for PtSn–Ce/SiO_2_–Al_2_O_3_ (1.0 wt% Pt and 1.8 wt% Sn as PtSn alloy, 0.5 wt% Ce, state unspecified) prepared by co-impregnation of SiO_2_–Al_2_O_3_ with H_2_PtCl_6_, SnCl_2_ and Ce(NO_3_)_3_. The better conversion achieved with the Ce-containing catalyst could be due to its increased acidity. In addition, PtSn–Ce/SiO_2_–Al_2_O_3_ was found to produce less coke, and particularly less hard coke (i.e., carbon that requires approx. 550 °C for removal under oxidizing conditions), compared to the catalyst without Ce. In another study, the presence of CeO_2_ (3 wt%) on Pt/HY enhanced Pt dispersion and prevented Pt sintering during PE hydrocracking at 280 °C under 20 bar H_2_, and during catalyst regeneration by calcination at 400 °C. The improved stability of the ceria-promoted catalyst was attributed to strong Pt–O–Ce interactions.

The high cost of the precious metal impacted the operating cost of PO upcycling, according to a preliminary techno-economic analysis.^134^ Studies with non-precious-metal (de)hydrogenating components have been reported. Several metals (Pt, Ru, Ni, all with surface areas of 0.07 nm^–2^) dispersed on TiO_2_-A-SG (anatase prepared by a sol–gel route) were explored in the hydrocracking of PP.^48^ The activity, based on yields of liquid/gas products relative to solid residue, followed the order Pt > Ni > Ru. Ni nanoparticles initially smaller than 5 nm aggregated to sizes greater than 10 nm during the reaction. Unfortunately, the activity of the used Ni catalyst was not investigated. A much higher conversion was reported using Ni/ZSM-5 (0.2 g, 2.5 wt% Ni, 3.1 ± 0.4 nm) to convert PE (1.6 g) at 375 °C compared to Co/ZSM-5 (0.2 g, 2.5 wt% Co, 3.6 ± 0.5 nm), 99 vs. 25 % after 6 h.^70^ The effect may be due to the higher (de)hydrogenating activity of Ni relative to Co.^238^

As an alternative to metallic nanoparticles, a classic hydrodesulfurization catalyst, MoS_x_, has a high tolerance for heteroatoms (S, N, O, etc.).^239^ It was used for LDPE hydrocracking.^213^ Highly dispersed MoS_x_/HBeta was prepared by chemical vapor deposition of Mo(CO)_6_ onto HBeta, followed by sulfidation. A decrease in external and internal surface areas indicated MoS_x_ formation both inside and outside of the pores. Interaction with the zeolite led to electron transfer from Mo to the support, as evidenced by XPS and XANES. EXAFS also revealed a Mo–O path, presumably arising from the support, but no Mo–Mo path, confirming the high dispersion of the MoS_x_ phase. For comparison, a catalyst with a similar Mo loading was prepared by solution impregnation of (NH_4_)_2_MoS_4_. For this material, the Mo XPS signal was essentially identical with that of MoS_2_, and no Mo–O path was detected by EXAFS, suggesting weaker interaction with the support. In LDPE hydrocracking at 200 °C under 25 bar H_2_, MoS_x_/HBeta showed a higher non-solid yield (75 wt%) compared with MoS_x_/HBeta-IM (60 wt%). This difference was attributed to an unspecified stronger interaction between MoS_x_ and the support in MoS_x_/HBeta.

Unexpectedly, metal-free MFI zeolites showed higher efficiency than metal-loaded (Pt or Ni) MFI zeolites in converting PE,^45^ although the BAS density was similar for both types of catalysts. A different behavior of the polymer relative to traditional hydrocracking of small hydrocarbons was attributed to inefficient diffusion of C=C sites located on long polymer chains between metal and acid active sites.

In summary, nanoparticles of transition metals and metal chalcogenides both catalyze (de)hydrogenation of POs. The type of metal significantly influences the (de)hydrogenation activity, although the effects of particle size and support interactions need further elucidation. While replacing precious metals with non-precious alternatives may reduce the initial catalyst cost, future changes in cost and availability due to the energy transition may be substantial.^139^ In addition, considerations of impurity tolerance and nanoparticle stability affecting catalyst productivity may be far more significant to the overall process cost.

#### 3.3.3 Effect of Metal–Acid Balance

As discussed in the section 3.1.3, the metal–acid balance (MAB) is essential in controlling activity and selectivity of hydrocracking for small alkanes. MAB is usually varied by changing the number of metal sites while keeping the number of acid sites constant. The next section describes the impact on activity and selectivity in PO hydrocracking.

##### 3.3.3.1 Effect of MAB on Isomerization Activity and Selectivity

**Figure 32:**
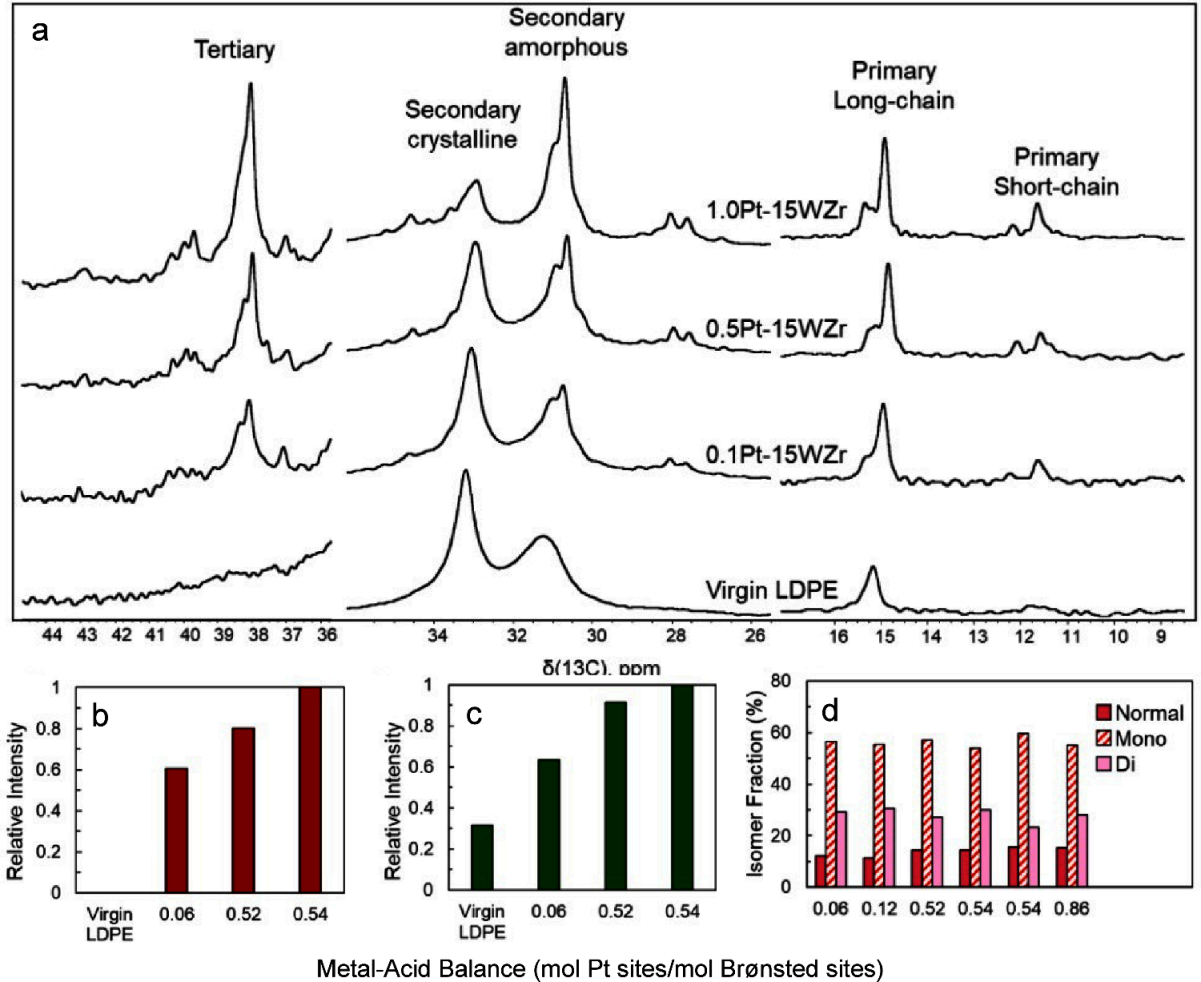
(a) ^13^C MAS NMR spectra of virgin LDPE and solid residues recovered from its depolymerization catalyzed by xPt–yWZr. Dependence on *n*_metal_/*n*_BAS_ of the signal intensities for (b) tertiary carbons; and (c) methyl groups, relative to the total number of methylenes. (d) Fractions of linear, mono-branched, and di-branched isomers in the extractable hydrocarbons. Conditions: 250 °C, 30 bar H_2_, 2 h. Reproduced with permission from ref (219). Copyright 2021, Elsevier.

Compared to small molecule hydrocarbons, characterization of branching is more challenging for products derived from POs, due to the complexity of the product distribution and the low solubility of some of the liquid hydrocarbons. The branching frequency in the solid residue (insoluble LDPE chains) remaining after hydrocracking LDPE with *x*Pt–*y*WZr catalysts was characterized by ^13^C solid-state MAS NMR (Figure 32a).^219^ The amount of branching increased with *n*_metal_/*n*_BAS_, as evidenced by the increase in the signal intensity for tertiary carbons (38 ppm) and methyl groups (12 ppm), Figure 32b,c. The fraction of methylene carbons present in a crystalline environment (33 ppm) also decreased, relative to those present in an amorphous phase (31 ppm, typical of highly branched polyethylenes). Consistent with these findings, the melting temperature and heat of fusion of the solid residue both decreased with increasing *n*_metal_/*n*_BAS_. These changes reflect isomerization/hydrocracking of long-chain *n*-alkanes.^195,240^

The selectivity for branched isomers in the extractable products was independent of *n*_metal_/*n*_BAS_ (Figure 32d). This finding contrasts with a prior report on *n*-decane hydrocracking,^195^ in which isomerization increased with *n*_metal_/*n*_BAS_. The difference was attributed to competition between light hydrocarbon products and larger alkanes (including polymer chains) for access to the acid sites. However, the *n*_metal_/*n*_BAS_ dependence in the *n*-decane study related only to the formation of decane isomers; the selectivity for branched isomers in the cracked products (in the range C_4_–C_7_) was nearly independent of *n*_metal_/*n*_BAS_. Thus, the effects of MAB on the hydrocracking of small molecule alkanes and LDPE appear to be similar.

In another study using a physical mixture of Pt/S-1 (Pt nanoparticles dispersed on the external surfaces of BAS-free silicalite) and HBeta zeolite, increasing the *n*_Pt_/*n*_BAS_ ratio from 0.02 to 0.05 (achieved by keeping *n*_BAS_ constant while changing *n*_Pt_) resulted in a higher *iso*-alkane selectivity [*i*-alkanes/(*i*-alkanes + *n*-alkanes)] in the volatile hydrocarbon products, increasing slightly from 83 to 88 (although the carbon number range was not specified).^241^ Similarly, the *iso*-alkane selectivity produced by a physical mixture of Pt@S-1 (Pt nanoparticles located in the S-1 micropores) and HBeta remained relatively stable (increasing only slightly from 67 to 75) despite an increase in the *n*_Pt_/*n*_BAS_ ratio from 0.05 to 0.17. Therefore, in both the physical mixture of HBeta + Pt/S-1, and in Pt@S-1 alone, the selectivity for *iso*-alkanes was similar despite the difference in *n*_metal_/*n*_BAS_. This observation is consistent with the finding in Figure 32d,^219^ where the lower *iso*-alkane selectivity of the physical mixture of Pt@S-1 and HBeta, compared to the physical mixture of Pt/S-1 and HBeta, was attributed to the confinement effect of Pt@S-1.

In summary, the rate of isomerization of PO chains increases with MAB (*n*_Pt_/*n*_BAS_), analogous to the behavior of small molecule hydrocarbons, and the selectivity for branched isomers in the cracked products is independent of *n*_Pt_/*n*_BAS_. However, the actual *n*_metal_/*n*_BAS_ ratio may not represent the effective value for PO hydrocracking, if some of the active sites are located in pores that polymer chains cannot access.

##### 3.3.3.2 Influence of MAB on C–C Bond Scission Reactivity and Selectivity

**Figure 33:**
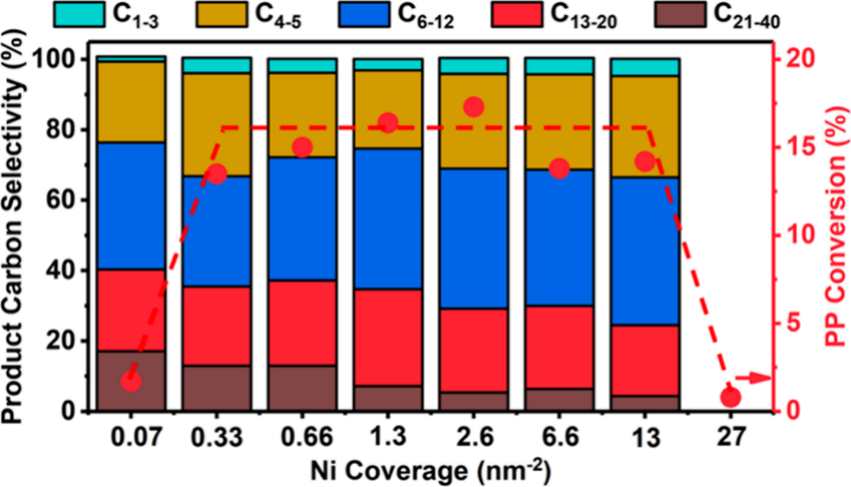
Effect of Ni coverage on PP hydrocracking catalyzed by Ni/TiO_2_-A-SG. The red dots/dashed line represent PP conversion (right ordinate), while stacked bars represent carbon selectivities. The Ni loadings (left to right) are 0.04, 0.25, 0.5, 1, 2, 5, 10, and 20 wt%. Reaction conditions: 260 °C, 30 bar H_2_, 1 g PP, 30 mg catalyst, 3 h. Reproduced with permission from ref (48). Copyright 2023, Elsevier.

Based on the discussion in section 3.1.3 for small hydrocarbons, the ratio of *n*_metal_/*n*_BAS_ is also expected to influence the rate and selectivity of C–C bond scission in PO hydrocracking. These effects were explored in a study of PP hydrocracking catalyzed by Ni/TiO_2_-A-SG.^48^ Upon varying the Ni loading from 0.07 to 0.33 nm^-2^, PP conversion increased from 2 to 15 %, then plateaued for Ni loadings from 0.33 to 13 nm^-2^, Figure 33. This observation is consistent with small alkane hydrocracking, as described in section 3.1.3.1. Further increasing the Ni loading to 27 nm^-2^ resulted in negligible PP conversion, possibly due to coverage of the BAS by Ni. This hypothesis was supported by a lack of H-bonding between surface hydroxyls and CO during chemisorption experiments. Similarly, in the hydrocracking of LDPE by Pt/HBeta, the product yield increased with Pt loading (from 0.5 to 1.0 wt%).^211^

In PP hydrocracking catalyzed by Ni/TiO_2_-A-SG, the overall selectivity to hydrocarbons in the range C_1_–C_40_ was similar but shifted slightly towards smaller carbon numbers as the Ni loading increased from 0.07 to 0.33 nm^–2^. This behavior differs from that observed in hydrocracking of longer-chain *n*-alkanes (C_>8_), where the selectivity shifted to higher carbon numbers with increasing *n*_metal_/*n*_BAS_ (as described in the section 3.1.3.2).

In contrast, heavier hydrocarbons formed when the ratio *n*_Pt_/*n*_BAS_ increased in a physical mixture of Pt/S-1 and HBeta.^241^ A similar finding was reported for *x*Pt–*y*WZr catalysts with Pt loadings *x* from 0.1 to 1.0 wt% and WO_3_ loadings y from 5 to 25 wt%.^219^ Increasing the *n*_metal_/*n*_BAS_ ratio from 0.06 to 0.86 caused the selectivity of extractable products in LDPE hydrocracking to shift from C_4_–C_6_ to larger hydrocarbons (e.g., the mass ratio C_21_/(C_4_–C_6_) increased from 0.05 to 0.7).

**Scheme 16:**
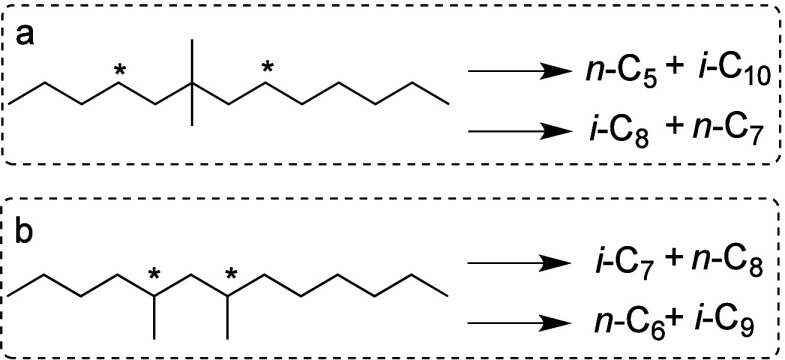
Expected Products for Various Pentadecyl Cations Undergoing of β-Scission of (a) Type B_1_; and (b) Type B_2_ the Positive Charge Is Located at Either of Carbon Atom Marked *

Formation of C_3_–C_4_ hydrocarbons as dominant products was suggested to be a fingerprint of type B β-scission (Scheme 16), since type A β-scission should result in relatively more long-chain hydrocarbons (C_9+_). However, hydrocarbon chain length distribution may not be a reliable indicator of the β-scission mechanism, since type B β-scission can also yield hydrocarbons larger than C_4_.^242^ As discussed in section 3.1.3, the key parameter that controls the yield of light (C_3_–C_4_) vs. heavier hydrocarbons is the alkene concentration, which is determined by the MAB. In addition, the structure of scission products is influenced by the rate of isomerization relative to β-scission.^195^

In conclusion, hydrocracking catalysts with high MAB (*n*_metal_/*n*_BAS_) ratios convert POs to higher-value alkanes (e.g., in the range of lubricant oils) through random scission, whereas low-MAB catalysts yield mostly lower-value C_4_–C_6_ products by end-of-chain scission. The C–C bond scission selectivity parallels that in small alkane hydrocracking. Although a higher metal content may be desirable for product selectivity, it raises the initial cost of the catalyst.

#### 3.3.4 Effect of Pore Structure and Morphology

The type of pore structure in solid acid catalysts has been reported to influence the type and molecular weight distribution of hydrocarbon products derived from catalytic cracking of POs, as well as the extent of coke formation.^191,205^ The blocking of external acid sites on MFI zeolites with bulky 2,4,6-trimethylpyridine was shown by TGA to completely shut down its reactivity towards PE at 250 °C under flowing Ar.^45^ This result suggests that PE chains crack into smaller hydrocarbon fragments on the external catalyst surface before diffusing into micropores, where further reactions occur.

To reduce the diffusion barriers that prevent POs from accessing active sites located in zeolite micropores, researchers have explored hierarchical zeolites as well as zeolites with different particle morphologies.^191,205^ After 7 h under flowing H_2_ at 280 °C, ZSM-5 with a Si/Al ratio of 21 and a *b*-axis length of 80–100 nm (denoted s-ZSM-5) was reported to convert PE (*m*_PE_/*m*_catalyst_ = 5) into light hydrocarbons (C_3_–C_7_, 60 wt%), of which the alkene fraction was 80 % (presumably also wt%, but not specified), as well as waxes and unreacted PE (C_>20_, 38 wt%), with a negligible amount of C_8_–C_20_.^209^ In contrast, “normal” ZSM-5 (denoted n-ZMS-5, *b*-axis length unspecified) with a similar Si/Al ratio produced only 17 wt% C_3_–C_7_, with wax/unreacted PE and coke comprising 40 wt% each.

**Figure 34:**
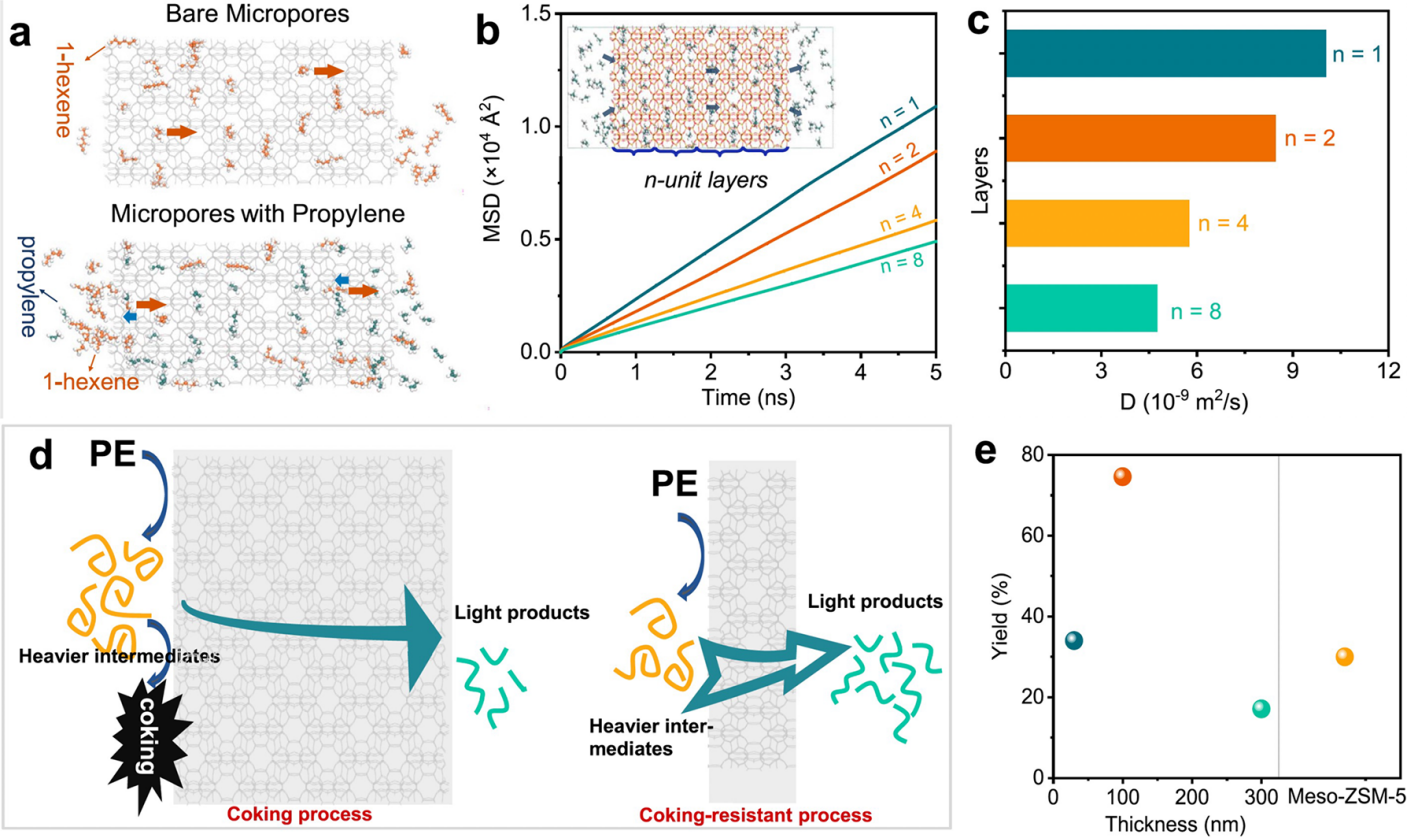
(a) Schematic illustration of the diffusion of 1-hexene through zeolite crystals with empty micropores and with micropores containing propylene. (b) Mean-square displacement (MSD) of propylene molecules diffusing across an s-ZSM-5 zeolite with 1, 2, 4, or 8 unit layers. Inset: model showing propylene diffusion in s-ZSM-5 micropores with 4 unit layers. (c) Propylene diffusion coefficients, in (d) s-ZSM-5 zeolite micropores with different unit layer thicknesses. (d) Schematic illustration of cracking on zeolite external surfaces for n-ZSM-5 (left) and within zeolite micropores for s-ZSM-5 (right). (e) C_1_–C_7_ yields from PE cracking catalyzed by ZSM-5 with different *b*-axis thicknesses, as well as in mesoporous ZSM-5. Reproduced with permission from ref (209). Copyright 2022, American Chemical Society.

To distinguish the roles of internal and external BAS, a catalyst with only external BAS was prepared by coating thin layers of aluminosilicate ZSM-5 on siliceous MFI zeolite (denoted *s*-S-1@ZSM-5).^209^ Its catalytic cracking activity towards PE was high, but the products were primarily waxes with only small amounts of C_3_–C_7_. Therefore, acid sites located in the micropores were proposed to participate in the formation of light alkenes. The diffusion of adsorbed molecules plays an important role in shape-selective catalysis, and molecular diffusion in zeolites has been modeled.^243^ In this study, diffusion coefficients in zeolite micropores for 1-hexene (simulating reaction intermediates) were determined in the presence of varying amounts of propylene (representing smaller alkene products). As expected, the values decrease as the amount of propylene increases. Furthermore, diffusion coefficients for propylene decreased as the thickness of the zeolite crystallites increased (Figure 34c). The authors inferred that a short diffusion distance is important to allow heavier intermediates to diffuse into pores and for cracked products to diffuse out, promoting catalytic activity and preventing coke accumulation. Notably, the optimal thickness of s-ZSM-5 appears to be in the intermediate range (Figure 34e), which will be an interesting direction to examine in future studies.

In “ideal” hydrocracking, C–C bond scission is random, as discussed in section 3.1.3. To achieve a narrower molecular weight distribution in PE hydrocracking, some studies have discussed the effect of pore size, achieved using different zeolite topologies, on acid site confinement. For example, the depolymerization of LDPE (*M*_w_ = 250,000 g mol^–1^, *Đ* not specified) was conducted with Pt/WO_3_/ZrO_2_ physically mixed with zeolites of different micropore sizes (HY ∼ HBEA > H-MOR > HZSM-5).^212^ The hydrocarbon products in LDPE depolymerization shifted from the gasoline and diesel ranges (i.e., heavier hydrocarbons) towards light gases (C_1_–C_4_) as the zeolite pore size decreased, suggesting that the pores induce “shape-selective” (or, more accurately, size-selective) catalysis. A similar effect has been widely studied in the (hydro)cracking of small alkanes.^244^ However, the product distribution depends not only on the selectivity of C–C bond scission, but also on the conversion (i.e., the number of C–C bond scission events). Unfortunately, selectivity comparisons of hydrocarbon product distributions were not made at similar conversions, which would have required adjusting the reaction times for catalysts with different activities.

**Figure 35:**
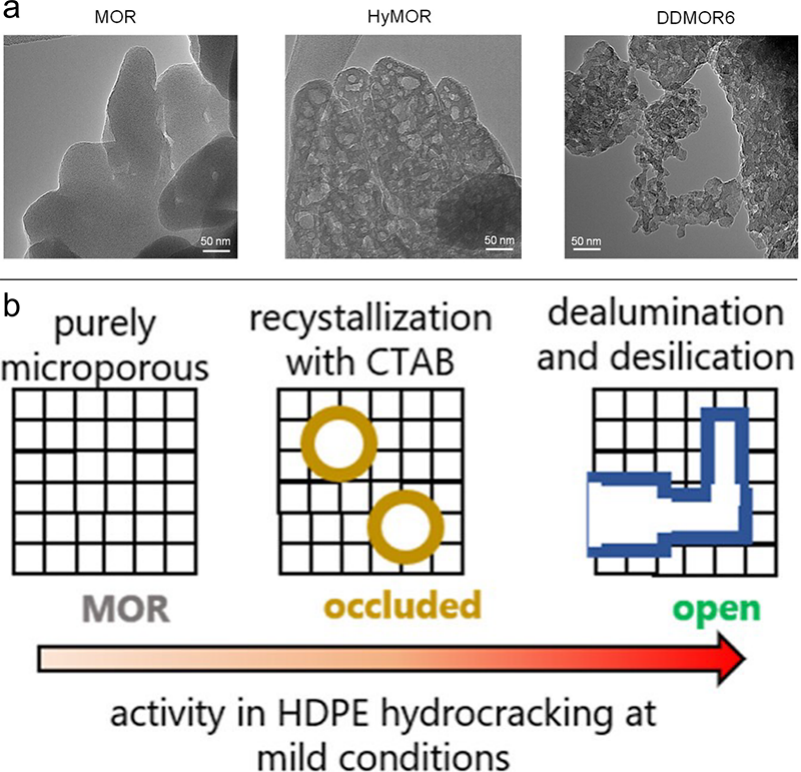
(a) TEM images; and (b) illustrations of pore geometries, for microporous mordenite (MOR), as well as hydrothermally-treated and recrystallized MOR (HyMOR), and desilicated and dealuminated MOR (DDMOR6). Reproduced with permission from ref (245). Copyright 2023, American Chemical Society.

To study the effect of pore structure, mordenite (SiO_2_/Al_2_O_3_ molar ratio of 20) was modified by hydrothermal treatment followed by recrystallization to create spherical occluded pores (HyMOR), and by desilication and dealumination to create open cylindrical mesopores (DDMOR6), Figure 35.^245^ Physical mixtures of the various zeolites with Pt/γ-Al_2_O_3_ were used in hydrocracking *n*-C_26_H_54_ and HDPE at 250 °C under 30 bar H_2_. Zeolites with either occluded or open mesopores showed similar reactivities towards *n*-C_26_H_54_. However, the catalyst with open mesopores gave an activity (defined as HDPE conversion normalized by BAS and time) 6× higher than unmodified MOR, while the catalyst with occluded mesopores showed a rate enhancement of only 2×. The difference may be a result of improved mass transfer for secondary (i.e., smaller) cracking intermediates in both zeolites, but only for the intact polymer in the zeolite with open mesopores. As polymer conversion increased with the change in catalyst from MOR to HyMOR to DDMOR6, the center of the product distribution shifted from C_5_ to C_6_ to C_7_, respectively, suggesting some size selectivity.

**Figure 36:**
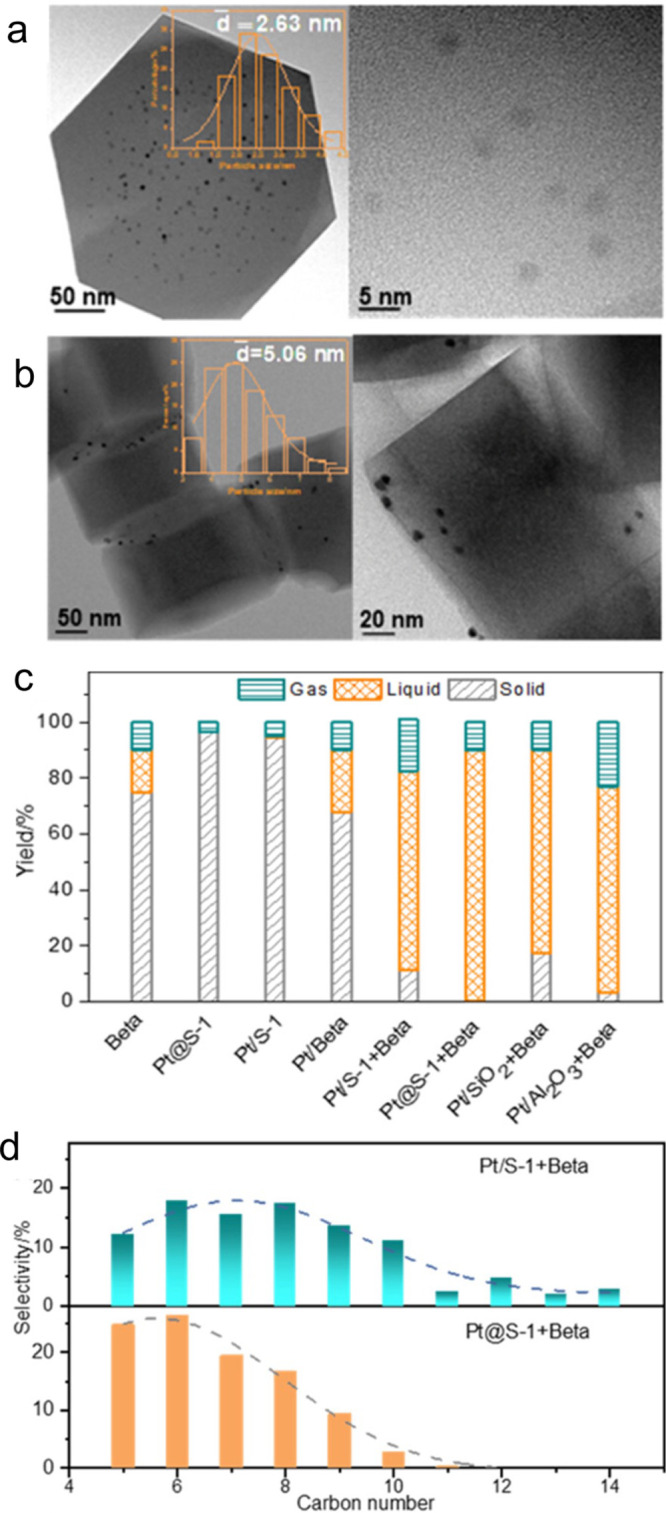
TEM images and particle size distributions for (a) Pt@S-1; and (b) Pt/S-1. Comparison of depolymerization products generated by (c) various Pt-zeolite combinations; and (d) Pt@S-1 and Pt/S-1 catalysts. The reaction involved 2.0 g LDPE and 0.2 g catalyst (where two catalysts were used, the mass was 0.1 g each, as a physical mixture), and was performed at 250 °C for 2 h under 30 bar H_2_. Reproduced with permission from ref (241). Copyright 2023, American Chemical Society.

Another approach involved confining the metal sites inside non-acidic silicalite (S-1),^241^ with the aim of synthesizing Pt nanoparticles in the micropores for comparison with Pt nanoparticles on the external surface (Pt@S-1 and Pt/S-1, respectively). The Pt nanoparticle location was inferred from TEM images, Figure 36. However, the average nanoparticle diameter in Pt@S-1 was much larger than the S-1 channel size (0.50–0.55 nm),^246^ so that many Pt nanoparticles were necessarily not confined within micropores.

Physically mixing various Pt-based catalysts (0.1 g, 0.3 wt% Pt) with acidic HBeta zeolite (0.1 g) significantly improved the activity relative to HBeta alone (Figure 36c), suggesting a bifunctional mechanism. C–C bond cleavage was suggested to occur at acid sites, producing alkenes that were rapidly hydrogenated on Pt. As discussed in section 3.3.2, the typical role of Pt is generating alkenes. This was not discussed, although monofunctional acid-catalyzed cracking of saturated hydrocarbons generally requires higher temperatures than metal-catalyzed dehydrogenation. Regardless of the mechanistic details, hydrogenation is thermodynamically favorable and shifts the depolymerization equilibrium.

A physical mixture of Pt@S-1 and HBeta (0.1 g each) gave the highest liquid yield (90 wt%, C_5_-C_10_) and the lowest yield of solid residue (<1 wt%). Surprisingly, Pt/HBeta produced much less liquid (22 wt%). The poor activity was attributed to close proximity between Pt and acid sites, which may promote carbon deposition on the Brønsted acid sites and hinder polymer cracking. Unfortunately, no information about coke formation was reported.

**Figure 37:**
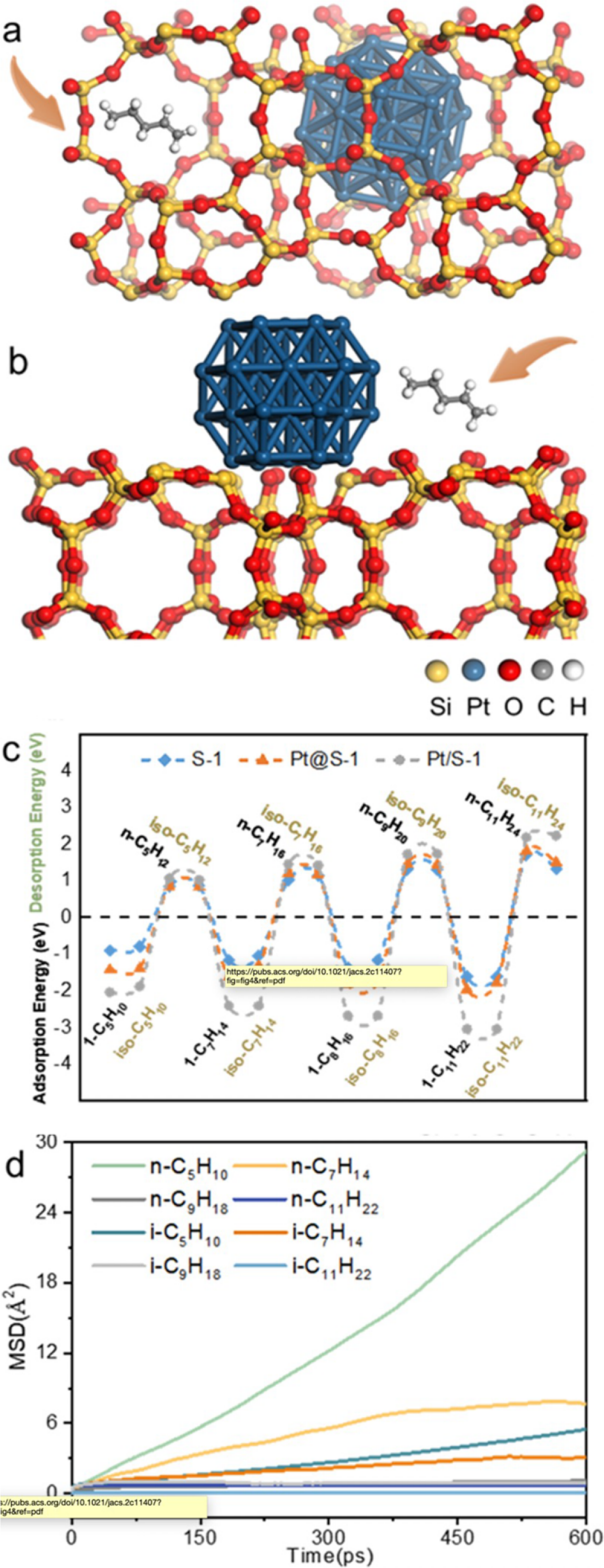
DFT calculations and molecular dynamics simulations of alkene adsorption, hydrogenation, and alkane desorption in (a) Pt@S-1; and (b) Pt/S-1 (Si in orange; O in red; Pt in cyan). (c) Energies of adsorption for alkenes and desorption for alkanes in S-1, Pt@S-1, and Pt/S-1. (d) Mean-square displacements (MSD) of alkenes diffusing in Pt@S-1 as a function of chain length and branching. Reproduced with permission from ref (241). Copyright 2023, American Chemical Society.

Compared to Pt/S-1, catalysts with Pt@S-1 gave products with a narrower carbon number distribution (Figure 36d). The origin of this “shape”-selectivity was explored using DFT. As expected based on studies of Fischer–Tropsch synthesis,^247^ adsorption energies for linear 1-alkenes and *iso*-alkenes (structure unspecified) on the externally exposed Pt NPs of Pt/S-1 increased significantly from C_5_ to C_11_ (Figure 37a–c), favoring the adsorption of heavier alkenes. Smaller alkenes diffused faster along the 10-membered-ring sinusoidal channels in Pt@S-1, as verified by molecular dynamics simulations (Figure 37d). Consequently, the acid sites in the HBeta catalyst component continued to activate and break C–H/C–C bonds until the alkenes became quite small (C_<11_). Since the adsorption energies of alkenes on Pt nanoparticles (e.g., in Pt@S-1) do not depend strongly on chain length, the small alkenes migrated onto Pt where they were hydrogenated to the corresponding alkanes.

In a complementary study, the effect of Pt nanoparticle location (either inside or outside zeolite micropores), and the effects of proximity between metal and acid sites (whether intimate or at nano/micro-scale distances) were systematically compared,^488^ using ion-exchange of Pt(NH_3_)_4_^2+^ (IE), incipient-wetness-impregnation of Pt(NH_3_)_4_^2+^ (IWI), and colloid-immobilization (CI) of Pt nanoparticles to synthesize three types of Pt/USY (0.1 wt% Pt, Si/Al = 22), denoted Pt/USY-IE, Pt/USY-IWI, and Pt/USY-CI. According to TEM, the mean diameters of the Pt nanoparticles ranged from 1.9 to 3.2 nm. Pt nanoparticles were located on the outer surface of the zeolite for Pt/USY-CI. Although the USY pore size is 0.74 nm, the nanoparticles in Pt/USY-IE and Pt/USY-IWI were deemed to be inside the pores. This conclusion was based on destructive elemental depth profile XPS analysis using Ar^+^ sputtering. It revealed a Pt signal only after the top 40 nm layer of USY was removed. The presence of Pt nanoparticles larger than the pore diameter was attributed to framework breakage. In all three catalysts, the total number and strength of acid sites were similar, as evidenced by NH_3_-TPD.

In the hydrocracking of PE (4.0 g, *M*_w_ and *Đ* unspecified), Pt/USY-CI (0.2 g) showed the highest conversion (62 wt%) after 3 h at 280 °C under 30 bar H_2_, compared to conversions of 29 and 46 wt% for Pt/USY-IE and Pt/USY-IWI, respectively. A physical mixture of Pt/γ-Al_2_O_3_ (0.02 g, Pt NPs 1.2 nm, 1.0 wt% Pt) and USY (0.2 g) gave 33 wt% conversion under the same conditions. All the product molecular weight distributions were centered at ca. C_7_, with selectivities to gasoline-range hydrocarbons (C_5_–C_12_) higher than 85 wt%.

**Scheme 17:**
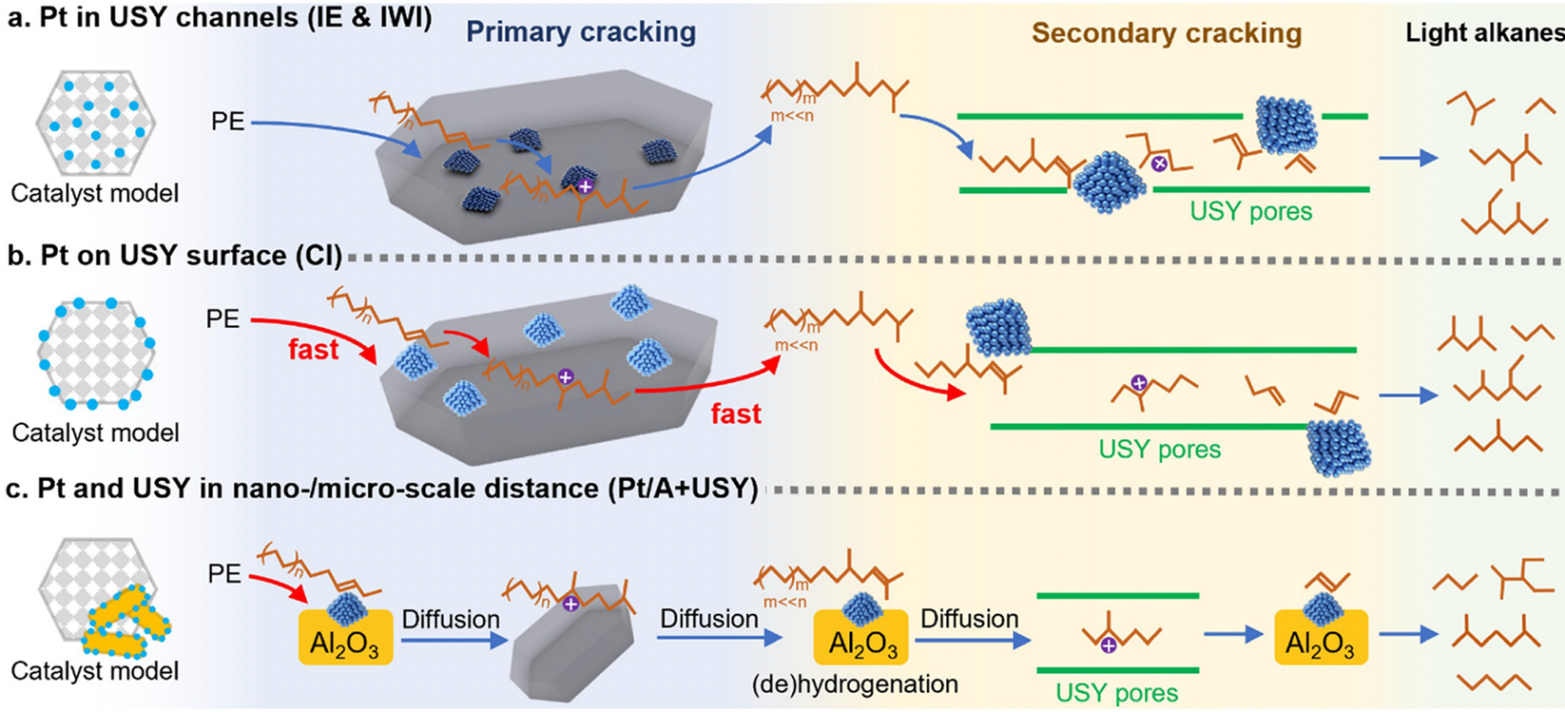
Proposed Mechanism for PE Hydrocracking Catalyzed by Various Pt/USY Materials, Based on Metal Location and Metal–Acid Proximity Reproduced with permission from ref (488). Copyright 2024, Royal Society of Chemistry.

The reactions of several *n*-alkanes of increasing size (C_6_, C_8_, C_12_, C_16_, and C_24_) were investigated to explore the accessibility of metal and acid sites. For Pt/USY-CI and the physical mixture of Pt/γ-Al_2_O_3_ and USY, conversions increased linearly with carbon number, similar to earlier observations^196,197^ (described in section 3.1.4). However, a volcano-shape pattern was observed for Pt/USY-IE and Pt/USY-IWI, with the highest conversion at ca. C_16_ for Pt/USY-IE and ca. C_24_ for Pt/USY-IWI (similar to another report,^203^ described in section 3.1.4). These results confirm that longer chains face stronger diffusion limitations when entering zeolite micropores. When the distances between Pt nanoparticles and acid sites are nm or even mm apart, the need for PE diffusion between the two types of active site slows the reaction. For the catalysts with no or little Pt on the external surfaces (i.e., Pt/USY-IE and Pt/USY-IWI), PE was inferred to crack slowly on external acid sites into smaller hydrocarbon chains, which enter the micropores and undergo further hydrocracking to light alkanes, Scheme 17. When Pt is present only on external surfaces (Pt/USY-CI), PE readily undergoes dehydrogenation on the accessible Pt nanoparticles followed by cracking on nearby acid sites, resulting in a faster depolymerization rate.

In summary, the lack of functional groups in PE and PP leads to hydrocarbon products with a broad carbon number distribution. To narrow the distribution, some studies used zeolite catalysts with their well-defined micropores to induce size selectivity. However, high molecular weight POs do not diffuse efficiently into the micropores where most of the acid active sites are located.^109^ Consequently, cracking occurs initially on external surfaces. The resulting hydrocarbon fragments then undergo further reactions within the micropores. Given the bifunctional nature of hydrocracking catalysts, the location of each type of active site within different pore structures can influence the outcome of the reaction.

Typically, the major products from zeolite-based catalysts are centered in the light gas (C_1_–C_4_) or gasoline (C_6_–C_12_) ranges. Catalysts that generate more valuable products with higher carbon numbers may be of higher interest. A better understanding of the interaction between polymers and various catalyst pore structures will aid in the design of more efficient and selective catalysts.

#### 3.3.5 Catalyst Stability and Regeneration

Techno-economic analyses suggest that low catalyst loading and facile catalyst regenerability will be important in making chemical recycling of waste polyolefins cost-effective.^134^ Similar to classical catalytic cracking and hydrocracking of naphtha, common reasons for loss of activity and selectivity in PO deconstruction catalysts include blockage of active sites by carbon deposition or accumulation of other poisons, collapse of the pore structure, and sintering or leaching of metal nanoparticles. Fortunately, the regeneration of both solid acid catalysts and metal/acid bifunctional catalysts is already widely practiced in the petroleum industry, and is readily translated to PO depolymerization catalysts.

Fast coke buildup is a significant problem in PO catalytic cracking. The amount and type of coke depends on the pore architecture. Generally, the amount of coke increases with pore size, and the coke contains larger fused aromatic ring structures.^205^ The use of ZSM-5 nanosheets with *b*-axis-oriented lengths of 80–100 nm was shown to largely suppress coke formation compared with normal ZSM-5 (*b*-axis length unspecified) because the shorter diffusion length promoted alkene removal and prevented coking.^209^ Although lower acidity resulted in lower catalytic reactivity, the decreased acidity achieved by steam treatment (at ca. 800 °C) improved the stability of ZSM-5 in the catalytic cracking of PO pyrolysis vapor (mostly C_>21_ linear hydrocarbon wax), leading to high yields of C_2_–C_4_ alkenes (>60 wt%) at approx. 600 °C.^248,249^

The ionic liquid [C_4_Py]Cl-AlCl_3_ used in tandem cracking-alkylation process of LDPE was reported to achieve full conversion in a batch process at least five times without regeneration.^210^ However, tests conducted at full conversion do not provide information about intrinsic catalyst stability. Minor products such as polymethylcyclopentadienes can deactivate the ionic liquid. These unsaturated products can be transformed into innocuous saturated hydrocarbons by reaction with H_2_ over a Pd/C catalyst, resulting in regeneration of the ionic liquid.

Hydrocracking catalysts are vulnerable to deactivation even when used at low reaction temperatures under high *P*_H2_. The recyclability of MoS_x_/HBeta was explored after its use in LDPE hydrocracking at 250 °C under 30 bar H_2_.^213^ The non-solid yield (NSY) decreased slightly after direct reuse (involving only simple washing and drying), and the product distribution shifted towards heavier components, indicating a decrease in the number of C–C bond scission events. The average Mo coordination environment remained unchanged according to EXAFS, suggesting a robust interaction between MoS_x_ and the support. A decrease in the NSY from an initial 94 wt% to 68 wt% after the second reuse was attributed to pore blockage by solid residue and coke (although the value at very high conversion does not reflect intrinsic activity). However, when the catalyst was regenerated by calcination in air at 500 °C followed by re-sulfidation, it gave an NSY of 97 wt%. Characterization after hydrocracking for 10 h at 250 °C and three regeneration cycles revealed no obvious aggregation of MoS_x_ according to high-angle annular dark-field STEM.

In other cases, regeneration changed the catalyst structure. TGA revealed the presence of coke deposits and post-reaction solid residues on a physical mixture of Pt/WO_3_/ZrO_2_ (0.1 g) and HY zeolite (0.1 g), after use in LDPE hydrocracking at 250 °C.^250^ The used catalyst mixture was regenerated by calcination at 500 °C, followed by reduction at 250 °C under H_2_. The C_5_–C_12_ selectivity remained constant, but the solid yield increased from 1.9 to 9.0 wt% after two regeneration cycles. CO chemisorption revealed that the Pt dispersion increased from 57 to 100 % upon regeneration, presumably due to PtO_2_ redistribution during calcination. X-ray diffraction analysis of the regenerated catalyst showed a decrease in the HY zeolite unit cell size, associated with a decrease in framework Al content (measured by X-ray fluorescence). Partial zeolite dealumination during regeneration resulting in a decrease in acidity may have been responsible for the small decrease in activity, although no direct acidity measurement was performed.

In summary, catalyst regeneration is important for the cost-effectiveness of PO upcycling. Some hydrocracking catalysts can be reused directly with minimal loss of activity, but in most cases, the activity decreases, presumably due to blockage of the active sites by hydrocarbon residues. Solid acid catalysts can be calcined to remove hydrocarbons, and the (de)hydrogenation sites of bifunctional catalysts can be regenerated with additional treatments (e.g., H_2_ reduction or sulfidation). The regeneration conditions may alter catalyst structure, necessitating careful control of the process. From a practical standpoint, additives present in post-consumer plastics may also deactivate catalysts (see section 3.5.5), warranting further study.

### 3.4 Kinetic and Mechanistic Studies

#### 3.4.1 Product Evolution and Reaction Mechanism

Since catalytic cracking and hydrocracking of POs involve a large number of primary and secondary reactions, product distributions are expected to vary over time. Information about the evolution of the product distribution is crucial for developing kinetic models and for understanding catalyst behavior. However, few studies report such time-resolved product distributions. Instead, most report lumped distributions, without analysis of individual carbon numbers. This approach is often necessary because of the very broad range of carbon numbers, from light gases to macromolecules. Nevertheless, even lumped product distributions can provide useful information about the reaction mechanism, as illustrated by the two examples below.

**Figure 38:**
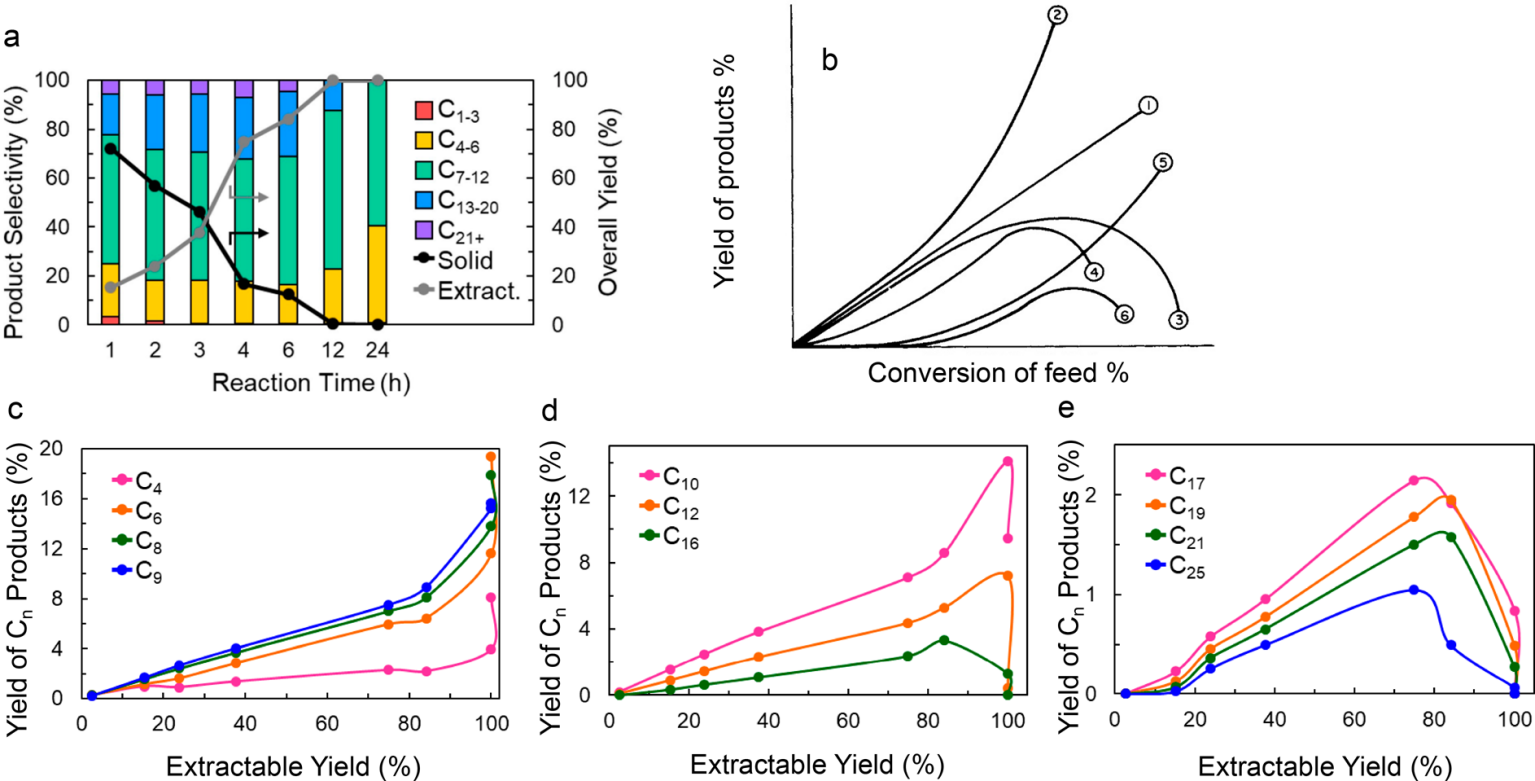
(a) Time-dependent yields of the solids and extractables recovered during LDPE hydrocracking catalyzed by 0.5Pt–15WZr. Optimum performance envelope (OPE) curves for hydrocarbon yields obtained in LDPE hydrocracking: (b) theoretical curves for various product types: 1-stable primary product; 2-stable primary plus secondary product; 3-unstable primary product; 4-unstable primary plus secondary product; 5-stable secondary product; 6-unstable secondary product. Reproduced with permission from ref (251). Copyright 2004, Wiley. (c–e) Experimental time-dependent data, obtained at time intervals from 1–24 h. Reaction conditions: LDPE (2.0 g), 0.5Pt–15WZr catalyst (200 mg), 250 °C, and 30 bar H_2_. Reproduced with permission from ref (219). Copyright 2021, Elsevier.

The progress of LDPE hydrocracking using a catalyst with a high *n*_metal_/*n*_BAS_ (0.5Pt–15WZr) was investigated by conducting time-dependent experiments.^219^ The mass selectivity for various extractable fractions remained essentially unchanged as the reaction proceeded, up to 6 h (Figure 38a). Only after the solid was fully consumed (≥12 h) did the product distribution shift to lighter alkanes (C_4_–C_6_). If polymer chains adsorb preferentially on the active sites at the expense of lighter hydrocarbons, hydrocracking of the latter should accelerate once the polymer is largely converted.

Optimum performance envelope (OPE) curves^251^ were constructed by plotting yields of C_n_ products vs. total yield of extractables, as a proxy for conversion (Figure 38c–e). The general shapes and initial slopes of the curves suggest that C_4_–C_17_ hydrocarbons are primary products of polymer cracking. Heavier hydrocarbons (C_∼17+_) are secondary products, formed via the cracking of smaller polymer chains (e.g., waxes). Obviously, intermediate-length hydrocarbons could be formed as primary, secondary, or tertiary products of depolymerization, complicating the analysis. (As noted in section 1.2, total extractable yield is not a reliable indicator of PE “conversion”.) Figure 38 shows that cracking continues even after the total yield of extractables reaches 100 %.

**Figure 39:**
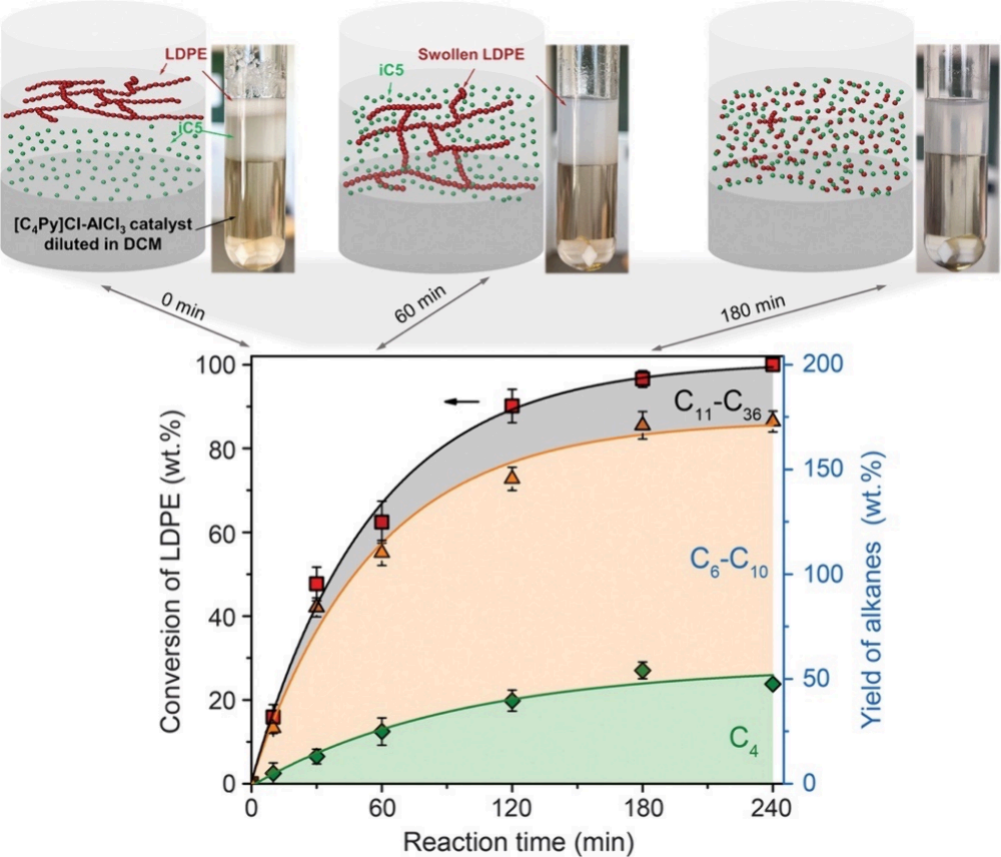
One-pot catalytic upcycling of LDPE with *i*-C_5_H_12_ into liquid alkanes in a Lewis acidic chloroaluminate ionic liquid. The graph shows time-resolved profiles for LDPE conversion and the cumulative yield of alkanes (C_4_, green diamonds; C_6_–C_10_, orange triangles; C_11_–C_36_, red squares,). Reaction conditions: LDPE, 200 mg; *i*-C_5_H_12_, 800 mg; [C_4_Py]Cl, 1 mmol; AlCl_3_ 2 mmol; ^t^BuCl, 0.05 mmol; CH_2_Cl_2_, 3 mL; 70 °C. Curves represent an “optimal” fit (details not described) to the data. Reproduced with permission from ref (210). Copyright 2023, AAAS.

The progress of the reaction between PE (*M*_w_ = 4,000 g/mol, *Đ* = 1.9, 0.2 g) and *i*-C_5_H_12_ (0.8 g) in the presence of *tert*-butyl chloride (TBC) and a Lewis acidic chloroaluminate ionic liquid ([C_4_Py]Cl-AlCl_3_, dissolved in CH_2_Cl_2_) was monitored at 70 °C.^210^ After 3 h, the PE was fully converted to alkanes, with carbon numbers from C_4_ to C_36_ (Figure 39). The yield was twice the initial PE mass (i.e., ca. 200 wt%), suggesting the incorporation of an equal mass of *i*C_5_H_12_. Since the products were predominantly C_6_–C_10_ at various degrees of conversion, the primary reactions were suggested to involve the direct combination of small hydrocarbon fragments (resulting from PE cracking) with *i*C_5_H_12_.

**Scheme 18:**
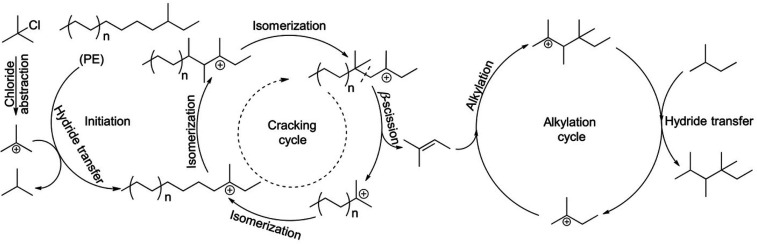
Proposed Mechanism for Tandem Polyolefin Cracking-Alkylation with *i-*C_5_H_12_ The 2-methyl-2-butene in the cracking cycle is a representative intermediate, based on the finding that 2,3,4,4-dimethylhexane is a representative product. Reproduced with permission from ref (210). Copyright 2023, AAAS.

A tandem cracking-alkylation mechanism was proposed based on a control experiment without *i*-C_5_H_12_, as well as a study performed with a model compound, *n*-C_16_H_34_. It includes the two catalytic cycles in Scheme 18. Chloride abstraction from TBC forms the *tert*-butylcarbenium ion, which abstracts a hydride (preferentially from a tertiary carbon atom) located on either *i*-C_5_H_12_ or the polymer. Carbenium ions formed within the polymer chain undergo skeletal isomerization, followed by β-scission to release small alkenes. These alkenes are alkylated by *i*-C_5_H_11_^+^, then abstract a hydride to form products in the C_6_–C_10_ range.

#### 3.4.2 Kinetic Modeling

##### 3.4.2.1 Lumped Models

Kinetic modeling can assist in reactor design via process simulation and optimization. A limited number of studies have appeared for PO catalytic cracking and hydrocracking. Due to the complexity of the product distribution, kinetic models typically lump hydrocarbons, either by molecular weight (polymer, heavy liquids, etc.) or by hydrocarbon type (alkanes, alkenes, etc.).^191,208,252^

**Scheme 19:**
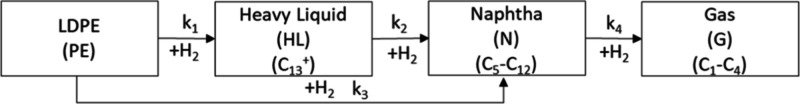
A Four-Lump Model Describing LDPE Hydrocracking to Heavy Liquids (HL), Then to Naphtha (N), and Finally to Light Gases (G) Reproduced with permission from ref (253). Copyright 2021 American Chemical Society.

A lumped model was developed for the hydrocracking of LDPE (20 g, *M*_w_ = ∼150,000 g/mol) catalyzed by bifunctional Pt/HBeta (2 g, 1 wt% Pt, Si/Al = 12.5), at 250–300 °C under 20 bar H_2_ in a batch reactor stirred at 400 rpm.^253^ Product lumping was performed by carbon number, as shown in Scheme 19.

Mass transfer effects were investigated to ensure kinetic control. Both external mass transfer and internal diffusion through catalyst pores were studied. LDPE conversion (eq 13) increased from 22 to 33 wt% as the stirring speed increased from 200 to 600 rpm, due to decreased external mass transfer resistance, eq 13:
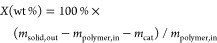
13where *m*_solid,out_, *m*_polymer,in_, and *m*_cat_ denote the masses of the post-reaction solid, the initial polymer charge, and the catalyst charge, respectively. The conversion also increased modestly, from 36 to 48 wt %, as the average particle size decreased from 550 to 175 μm due to decreased internal mass transport resistance.

**Figure 40:**
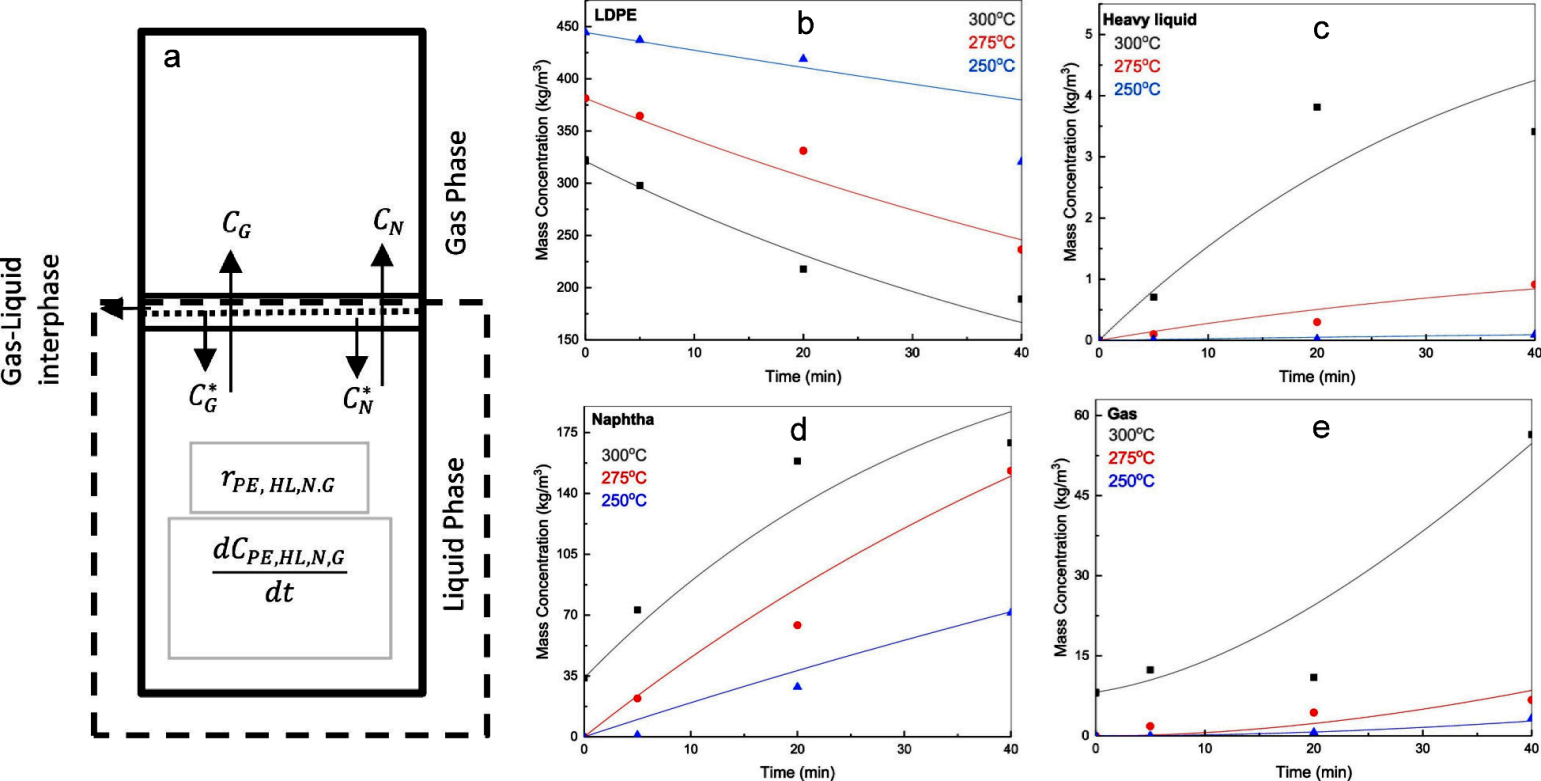
(a) Schematic of the stirred batch reactor. Evolution of mass concentrations for various hydrocarbon groups: (b) PE; (c) heavy liquids (HL); (d) naphtha (N); and (e) gases (G), as a function of time and temperature in LDPE hydrocracking catalyzed by Pt/HBeta (1 wt% Pt). Points represent experimental data, while lines are predicted by the model. Reproduced with permission from ref (253). Copyright 2021, American Chemical Society.

Subsequent experiments were conducted using an average catalyst particle size of 175 μm and a stirring speed of 600 rpm, to minimize the effects of reactant and product diffusion within the catalyst. Reactions were assumed to occur only in the condensed phase, as depicted in Figure 40a. For each reaction, a kinetic expression was formulated as a function of the hydrocarbon mass concentration in the liquid phase (*C*_*i*_) and, for volatiles, the equilibrium interphase (*C*_i_^*^), a rate constant (*k*_*j*_), a mass transfer coefficient (*k*_L_*a*_*i*_, for external mass transfer), and an effectiveness factor (*η*_*i*_, for internal diffusion). Kinetic expressions assume first-order behavior with respect to each hydrocarbon group and zeroth-order behavior with respect to H_2_ (eqs 14–17). Each experiment was modeled independently, resulting in a set of estimated rate constants and mass transfer coefficients as a function of temperature, Figure 40b–e.
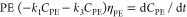
14
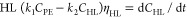
15
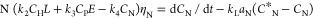
16
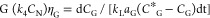
17

The lumped rate constants for the hydrocracking of different hydrocarbon groups decreased in the order: *k*_3_ (LDPE to naphtha) > *k*_4_ (naphtha to gas) > *k*_2_ (heavy liquids to naphtha) > *k*_1_ (LDPE to heavy liquids), Table 2. In principle, the nature of all of these processes is the same, i.e., C–C bond scission. The observed variation in rate constants reflects a complex dependence of the catalytic activity on hydrocarbon chain length. However, the model did not consider additional possible transformations, such as LDPE to gases, and heavy liquids to gases.

**Table 2:** Pseudo-first-order Rate Constants (s^–1^) from a Lumped Kinetic Model for LDPE Hydrocracking^a^

*T* (°C)	10^8^*k*_1_ (LDPE to heavy liquids)	10^4^*k*_2_ (heavy liquids to naphtha)	10^5^*k*_3_ (LDPE to naphtha)	10^5^*k*_4_ (naphtha to gas)
300	12	1.9	41	16
275	1.6	1.4	24	4.5
250	0.12	1.1	8.0	3.2

a Experimental conditions: LDPE (20 g, *M*_w_ = ∼150,000 g/mol) over a bifunctional catalyst Pt/HBeta (2 g, 1 wt% Pt, Si/Al = 12.5), under 20 bar H_2_ in a stirred batch reactor. Reproduced with permission from ref (253). Copyright 2021, American Chemical Society.

As expected, the rate constants increased with temperature. The estimated effectiveness factors for the lighter hydrocarbon groups (naphtha and gases) were nearly unity for all temperatures. However, the effectiveness factors were lower for the heavier hydrocarbon groups (LDPE and heavy liquid), reflecting more significant internal diffusion limitations. Overall, the lumped model indicated that the slowest step is the hydrocracking of LDPE to heavy liquids, with internal diffusion limitations being more significant for longer hydrocarbon chains.

In summary, the complexity of hydrocarbon mixtures in PO hydrocracking makes it challenging to develop detailed kinetic models. Lumped models can nevertheless lead to coarse-grained information about kinetics and diffusion.

##### 3.4.2.2 Population Balance Modeling

While lumped kinetic models are convenient, they provide minimal information about the evolving molecular weight distribution (MWD) of the hydrocarbon mixture. In contrast, population balance models are capable of predicting this distribution, based on the reaction mechanism.

**Figure 41:**
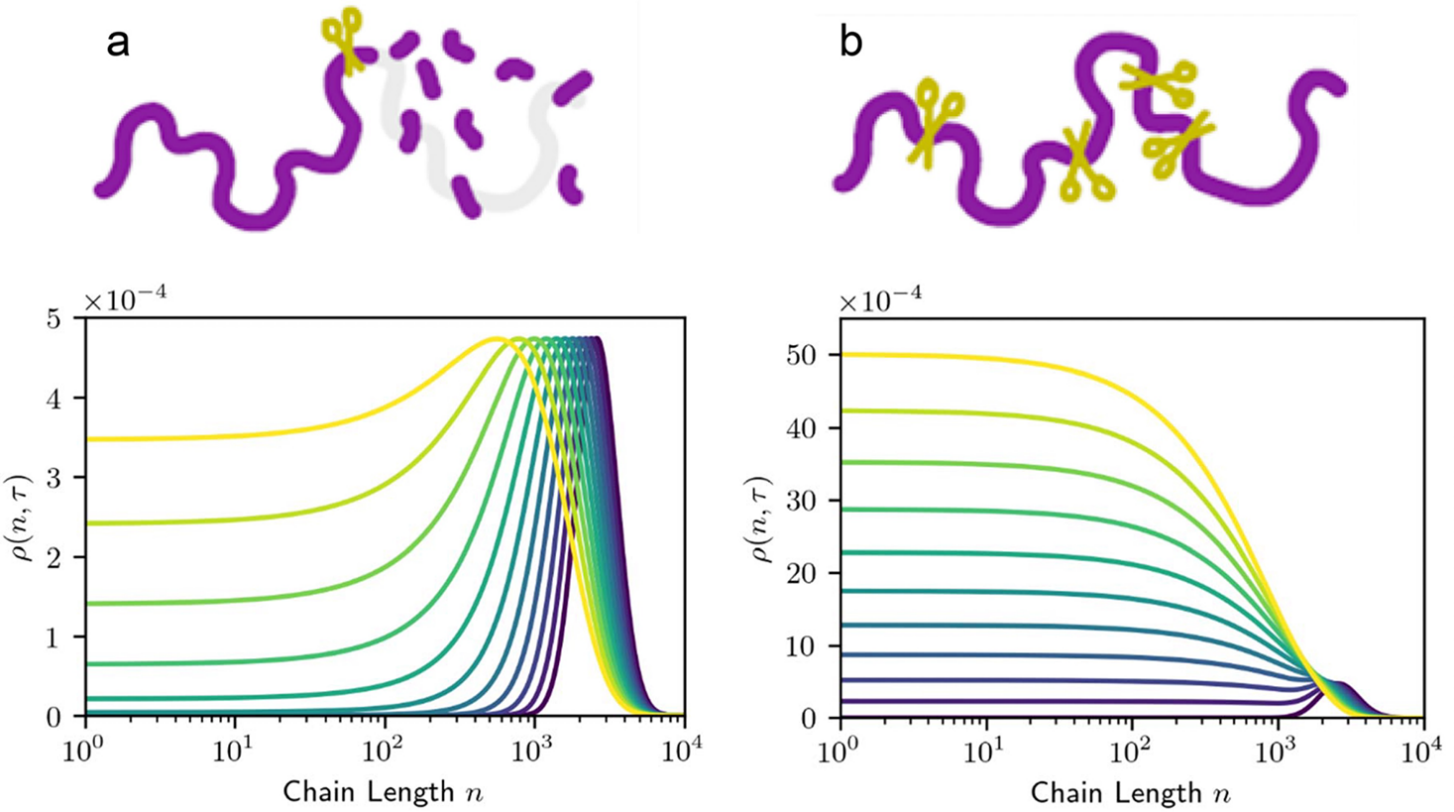
Time evolution of the molecular weight distribution (MWD) of a hydrocarbon mixture undergoing hydrocracking by (a) chain-end scission (note: the monomer concentration is not shown); and (b) random chain scission. Each line denotes the MWD at a point evenly spaced in *t*, with the initial distribution at *t* = 0 (purple). Adapted with permission from ref (254). Copyright 2021, Royal Society of Chemistry. The illustration of chain-end scission and random scission is reproduced with permission from ref (255). Copyright 2023, Royal Society of Chemistry.

As discussed in section 3.1.3, catalytic cracking involves mostly near-chain-end scission, producing C_3_–C_6_ hydrocarbon products, while ideal hydrocracking involves random scission (i.e., all C–C bonds have an equal probability of scission). MWDs for near-chain-end and random scission were modeled using population balance models.^254^ Near-chain-end scission resulted in a gradual broadening of the MWD and gradual translation to lower chain lengths (Figure 41a). On the other hand, random scission generated a rising plateau, especially among smaller hydrocarbons that were absent from the initial distribution (Figure 41b).^255^ For heterogeneous catalysts, hydrocarbon adsorption constants depend on chain length, which in turn influences the shape of the distribution (not shown).^254^ Although population balance models can predict signatures of different chain scission mechanisms via their kinetic behavior, it has thus far proven challenging to obtain experimental carbon number distributions in enough detail to compare to these simulations.

### 3.5 Impact of Reaction and Feedstock Variables

#### 3.5.1 Effect of H_2_ Pressure

Since cracking catalysts generally lack sites for readily activating H_2_,^256^ cracking is not typically conducted under a H_2_-containing atmosphere. Nevertheless, some studies have investigated the impact of H_2_. PE cracking was performed using s-ZSM-5 (*b*-axis length 80–100 nm, Si/Al atomic ratio = 21) at 280 °C. When the reaction was conducted under flowing N_2_ (i.e., H_2_ was absent), the yield of C_3_–C_7_ products was 38 wt% (with 90 % selectivity to alkenes, molar or mass basis unspecified), compared to 60 wt% (with 80 % selectivity to alkenes) under flowing H_2_ (1 vol% in N_2_).

The effect of H_2_ on PE cracking catalyzed by H-MFI-40 was also studied.^45^ The PE conversion (defined as in eq 2) was 10 wt% lower when the H_2_ pressure was 30 bar, compared to 10 bar. However, the carbon number distribution in the gas products changed little. At the higher H_2_ pressure, the ratio of alkanes to alkenes increased, suggesting that the metal-free zeolite facilitated alkene hydrogenation. Consequently, the decrease in PE conversion at higher *P*_H2_ might arise due to a lower steady-state alkene concentration, causing a decrease in the amount of carbenium ions formed. In an attempt to rule out the possibility of adventitious metal catalysis, a blank experiment was performed with *n*-hexane. No hexenes were observed after 20 h at 200 °C under 10 bar H_2_. However, the (de)hydrogenation equilibrium strongly favors hexanes under these conditions (*K*_p_ for 1-hexene hydrogenation to *n*-hexane is ca. 10^6^ at 230 °C),^258^ so this experiment does not appear to be a reliable test for metal catalysis.

**Figure 42:**
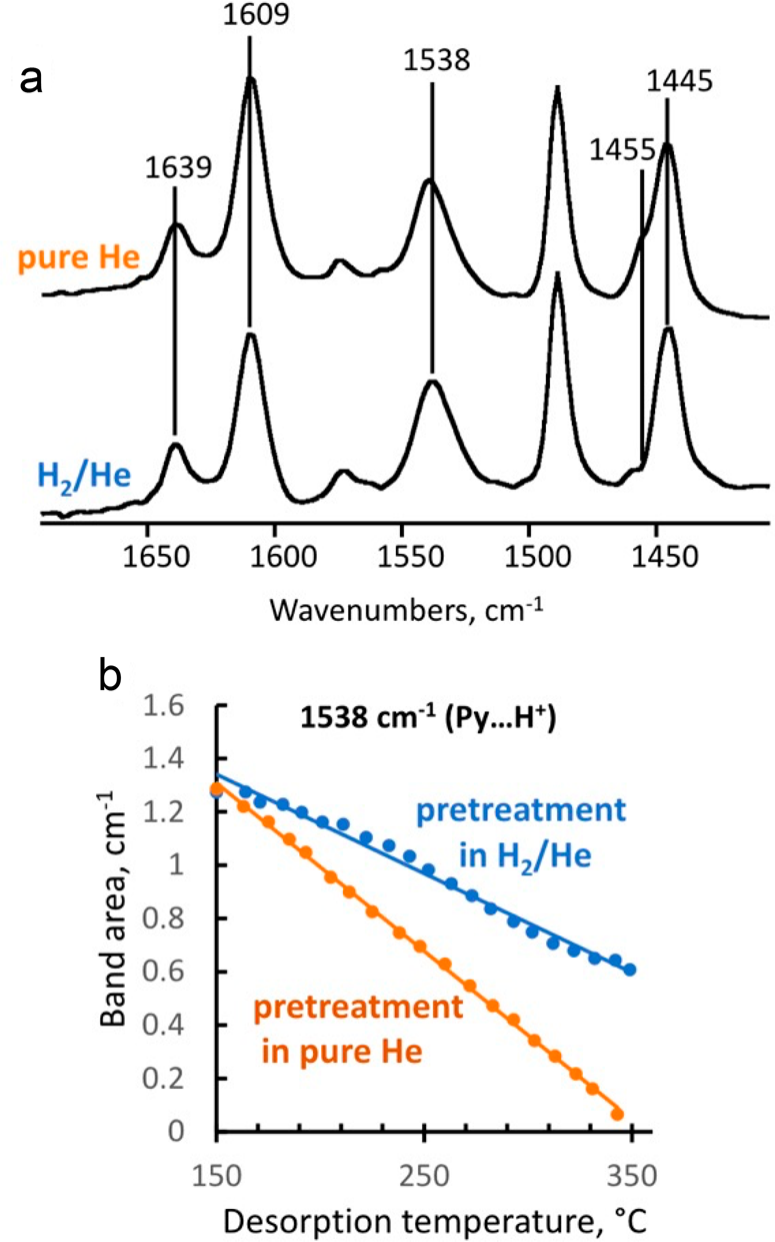
Effect of H_2_ pretreatment on Brønsted acid strength of a Pt/WO_3_/ZrO_2_ catalyst: (a) IR spectra of adsorbed pyridine on catalysts pre-treated at 250 °C in a flow of either He or H_2_ (20 % in He). Weakly-adsorbed pyridine was desorbed at 150 °C in flowing He prior to recording the spectra. (b) Decrease in the area of the band due to chemisorbed pyridinium with desorption temperature. While both catalysts have similar total numbers of Brønsted acid sites, the lower slope for the H_2_-pretreated catalyst indicates its Brønsted acid sites are stronger. Reproduced with permission from ref (212). Copyright 2020, AAAS.

For hydrocracking, typical conditions include a large excess of H_2_. Since equilibrium constants for C–C bond hydrogenolysis are large, that reaction is not equilibrium-limited. For light alkanes, the reaction order in H_2_ is usually negative, because H_2_ limits the equilibrium concentration of alkenes which are the precursors of carbenium ion intermediates.^259^ In some reports, the kinetics of PO hydrocracking show a complex dependence on *P*_H2_. For example, the maximum rate of LDPE hydrocracking catalyzed by a physical mixture of Pt/WO_3_/ZrO_2_ (0.1 g) and HY zeolite (0.1 g) at 250 °C was reported to occur in the range 20 - 50 bar, specifically, near 30 bar H_2_.^212^ Acid strength as well as the number of acid sites (as measured by pyridine adsorption) increased when the catalyst was pretreated in H_2_ at 250 °C (Figure 42). No chemical explanation was offered for the effect of H_2_ pretreatment on BAS *strength*, although reduction of WO_x_ does create BAS. Furthermore, this result reveals only that the catalyst changes when an inert atmosphere is replaced by H_2_, without addressing why the activity varied as a function of *P*_H2_.

Increasing *P*_H2_ from 10 to 30 bar similarly enhanced the rate of LDPE hydrocracking catalyzed by Pt@S-1 at 250 °C.^241^ However, an even higher pressure (40 bar) reduced the LDPE conversion, presumably by reducing the steady-state alkene concentration. The activity of MoS_x_/HBeta (inferred from the combined yields of gases and CH_2_Cl_2_-soluble hydrocarbons) was positively correlated with *P*_H2_ from 15 to 35 bar at 250 °C.^213^ The PE hydrocracking activity of Pt/SiO_2_-Al_2_O_3_ (inferred from the combined yields of gases and hexane-soluble hydrocarbons) was positively correlated with *P*_H2_ from 0 to 12 bar at 300 °C, while the activity of SiO_2_-Al_2_O_3_ was independent of *P*_H2_.^257^

In summary, *P*_H2_ influences both cracking and hydrocracking of POs. For hydrocracking, the activity tends to increase at lower *P*_H2_ values then decrease at higher *P*_H2_ values, resulting in a maximum rate at intermediate *P*_H2_ values. The decline in rate at high *P*_H2_ is consistent with traditional bifunctional hydrocracking, while the origin of the increased rate at low *P*_H2_ requires further investigation.

#### 3.5.2 Effect of Molecular Weight

The molecular weight distributions of hydrocarbons from PO (hydro)cracking are influenced by the intramolecular (terminal vs. internal C–C) selectivity as well as the intermolecular (longer vs. shorter chain scission) selectivity. Understanding the effects of molecular weight on C–C bond scission provides information about selectivity, facilitating kinetic modeling. Fundamental understanding is often more readily obtained using small molecule model compounds and/or low molecular weight POs (*M*_w_ < 10,000 g/mol), due to the greater ease of product analysis. However, commercial POs usually have much higher molecular weights. For instance, HDPE typically has *M*_w_ values from 50,000 to 250,000 g/mol, while ultra-high molecular-weight PE can have values an order of magnitude higher.^260^ Commercial PPs used in packaging typically have *M*_w_ values between 140,000 and 640,000 g mol^–1^.^109^ Therefore, it is important to understand the impact of molecular weight differences between model compounds and commercial plastics on reactivity.

The hydrocracking activity of Pt/HBeta towards LDPE (*M*_w_ = 150,000 g mol^–1^, *Đ* not specified) and a model hydrocarbon, squalane (2,6,10,15,19,23-hexamethyltetracosane, C_30_H_62_), were compared at 275–330 °C under 20 bar H_2_. Squalane was considered to be sufficiently large to simulate the behavior of PE chains.^211^ Since the zeolite pore size excludes both types of molecules, the initial reaction must occur on external or near-surface internal sites. The LDPE conversion (defined as the mass yield of non-solid hydrocarbons, relative to the initial hydrocarbon loading) was lower than that of squalane (evaluated using a traditional definition of conversion). The cause of the difference was suggested to be slower mass transfer of the polymer relative to squalane, and/or lower solubility of H_2_ in the molten PE (although neither potential explanation was fully explored). In addition, the mass of residual solid may not be a reliable descriptor of reactivity for the LDPE, since longer chains may undergo significant cleavage but still remain poorly soluble and/or non-volatile (see section 1.2.1.1). In contrast, all hydrocarbon products arising from molecular squalane can be characterized by GC.

**Figure 43:**
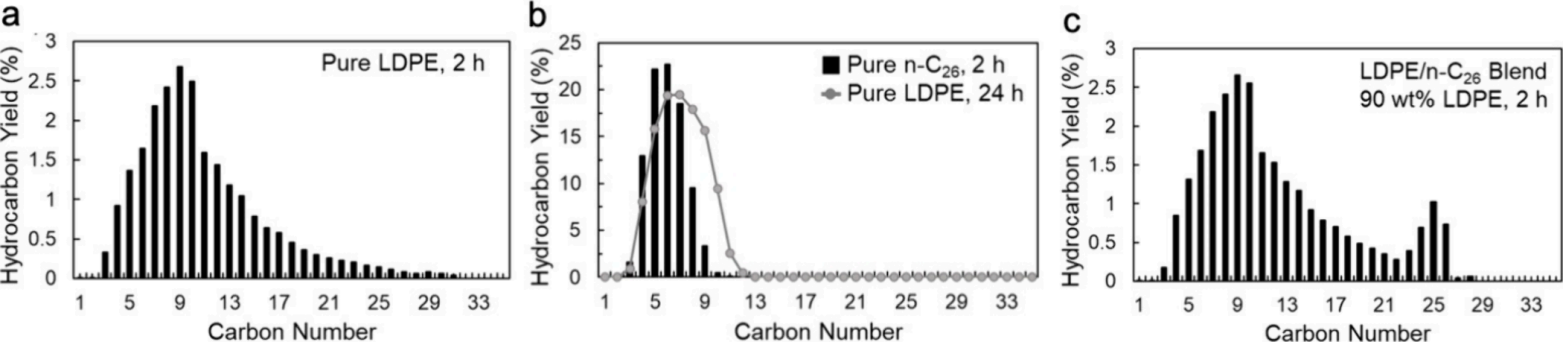
Light hydrocarbon product distributions present after 2 h hydrocracking catalyzed by 0.5Pt–15WZr at 250 °C and 30 bar H_2_. The feed was (a) LDPE alone; (b) *n*-hexacosane alone (compared to results from LDPE alone, after 24 h reaction); and (c) a mixture of LDPE (90 wt%) and *n*-hexacosane (10 wt%). Reproduced with permission from ref (219). Copyright 2021, Elsevier.

The reactivity of a mesoporous hydrocracking catalyst (0.5Pt–15WZr, consisting of 0.5 wt% Pt and 15 wt% WO_3_ supported on ZrO_2_) toward LDPE (*M*_w_ = 76,000 g/mol, *Đ* unspecified) and *n*-hexacosane (*n*-C_26_H_54_) was compared.^219^ In 2 h at 250 °C under 30 bar H_2_, the polymer was partially converted to hydrocarbons in the range C_3_–C_33_ (Figure 43a). Although heavier alkanes adsorb more strongly on a catalyst surface than light alkanes (see section 3.1.4), the expectation that LDPE should be converted faster than *n*-hexacosane was not borne out. A much longer reaction time (24 h) was required to achieve a similar yield of shorter hydrocarbons from LDPE, compared with *n*-hexacosane (2 h) (Figure 43b). However, LDPE requires many more C–C bond scission events relative to *n*-hexacosane to generate the same product distribution. For example, an LDPE chain with an average of 6000 CH_2_ groups will be fully converted C_6_H_14_ after approx. 1000 C–C bond scissions, while only 4 C–C bond scissions are required to convert C_26_H_54_ to C_6_H_14_.

The hydrocarbon product distribution was very different when LDPE was mixed with the model hydrocarbon (90:10 w/w), Figure 43c. The authors concluded that the presence of the polymer prevents smaller hydrocarbons from accessing the active sites. This inability of shorter chains to adsorb on the active sites in the presence of longer chains was attributed to the difference in their heats of adsorption. Thus, the suppression of *n*-hexacosane reactivity in the presence of LDPE suggests that the active sites were primarily occupied by the polymer, as previously proposed.^203^

Thermogravimetric experiments were conducted to explore the catalytic cracking of a series of PPs (*M*_w_ from 6,000 to 421,000 g/mol) using an FCC catalyst (a spray-dried mixture of silica-alumina, clay, and zeolite Y with FAU topology).^109^ The shorter PP chains cracked below 275 °C, while the longer chains required a higher temperature (425 °C). The difference was attributed to the higher melt viscosity of the longer chains, resulting in transport limitations.

In sum, some studies report that low molecular weight alkanes crack more readily than high molecular weight PO chains, in contrast to the behavior of small alkanes. The lower yields reported from POs may be a consequence of slower mass transfer, since viscosity increases dramatically with both molecular weight^261^ and branch frequency.^262^ However, liquid product yields are not a reliable proxy for reactivity (see section 1.2.2.1). Systematic studies for a range of PO molecular weights, along with more precise activity measurements (for example, quantifying C–C bond scission events via H_2_ uptake), will be required to deconvolute the effects of chain length from transport and adsorption effects. Furthermore, since depolymerization generates mixtures of hydrocarbon chain lengths, the behaviors of individual chain lengths may not reflect the behaviors of mixtures.

#### 3.5.3 Effect of Polyolefin Microstructure

##### 3.5.3.1 Effect of Short-Chain Branching

Reactivity in PO hydrocracking and hydrogenolysis depends on the number of branches on the polymer backbone. The hydrocracking of isotactic PP (described as having an average molecular weight *M*_avg_ = 250,000 g/mol, without indicating *M*_n_, *M*_w_, or *Đ*), catalyzed by bifunctional Pt/sulfated-zirconia under 83 bar H_2_, was compared to the hydrocracking of HDPE with the same *M*_avg_ value. The iPP was more reactive, showing full conversion to C_4_-C_9_ branched alkanes (60-70 wt%) at a temperature 50 °C lower compared to the HDPE.^263^ As expected, PP hydrocracking produced a higher ratio of branched to normal alkanes than did HDPE. Similar observations were reported using a physical mixture of catalysts. Thus, hydrocracking of iPP (*M*_w_ = 250,000 g/mol, *Đ* not specified) using a mixture of Pt/WO_3_/ZrO_2_ (0.1 g) and HY zeolite (0.1 g) was reported to give a diesel-like product in 43 wt% yield, higher than the yields from either LDPE (16 wt%, *M*_w_ = 250,000 g/mol, *Đ* not specified) or HDPE (19 wt%, *M*_w_ and *Đ* not specified) under similar conditions.^212^ The higher yield was attributed to the more abundant tertiary carbons in PP. Using a catalyst mixture of Pt@S-1 and Beta zeolite, a higher gas yield was observed from PP (26 wt%; tacticity, *M*_w_ and *Đ* not specified) compared to HDPE (15 wt%, *M*_w_ and *Đ* not specified) after 2 h at 250 °C, also suggesting that PP is more reactive.^241^

The rate of β-scission in hydrocracking increases with the number of branch points (see section 3.1.2), while C–C bond cleavage by hydrogenolysis in HR_2_C–CH_*x*_R_3–*x*_ (*x* = 0, 1, 2, or 3) is slower than in H_2_RC–CH_2_R or H_2_RC–CH_3_, due to steric hindrance.^84,96^ In reactions of various hydrocarbons catalyzed by Ni or Ru supported on TiO_2_-A-SG at 260 °C, the ratio (C_2_ + C_3_)/C_4_ in the gas products decreased as the branch frequency increased from *n*-C_16_H_34_ (no tertiary C), to LDPE (*M*_w_ = ca. 250,000 g/mol, *Đ* = 3.1, 2 mol% tertiary C), squalane (C_30_H_62_, 20 mol% tertiary C), and PP (isotactic, *M*_w_ = ca. 250,000 g/mol, *Đ* = 3.7, 33 mol% tertiary C), suggesting that the contribution of hydrocracking increases relative to hydrogenolysis as the fraction of tertiary C increases.^48^ A similar conclusion was reached based on liquid yields from PE (*M*_w_ = 4,000 g/mol, *Đ* unspecified), which correlated with Ru dispersion (but not acidity) for various catalysts (Ru/FAU, Ru/BEA, Ru/C, Ru/SIRAL30, Ru/SIRAL40HPV, and Ru/Si-BEA). In contrast, the liquid yields from PP (*M*_w_ = 12,000 g/mol, *Đ* and tacticity unspecified) were better correlated with the number of acid sites for the same series of Ru catalysts at ca. 200 °C under 30 bar H_2_.^123^

When depolymerization was catalyzed by microporous MoS_x_-HBeta at 250 °C under 20–30 bar H_2_, lower non-solid yields (NSY, defined as the sum of gas and liquid products) were recovered from PP (*M*_w_ = 300,000 g/mol, *Đ* and tacticity unspecified) and LDPE (*M*_w_ = 300,000 g/mol, *Đ* unspecified), compared to HDPE (*M*_w_ = 300,000 g/mol, *Đ* unspecified) and LLDPE (*M*_w_ = 300,000 g/mol, *Đ* unspecified).^213^ The highly branched structures of PP and LDPE were presumed to hinder the rates at which their polymer chains enter the zeolite channels,^264^ even though the tertiary carbon atoms should facilitate C–C bond scission.

In conclusion, reactivity in PO hydrocracking typically follows the order HDPE < LDPE < PP for non-microporous catalysts, and the selectivity for *iso*-alkane products increases in the same order. However, higher branching impedes interactions with acid sites in zeolite micropores, and direct hydrogenolysis may be faster for hydrocarbons with fewer branches.

##### 3.5.3.2 Effect of Aromatic Side-Chains

**Scheme 20:**
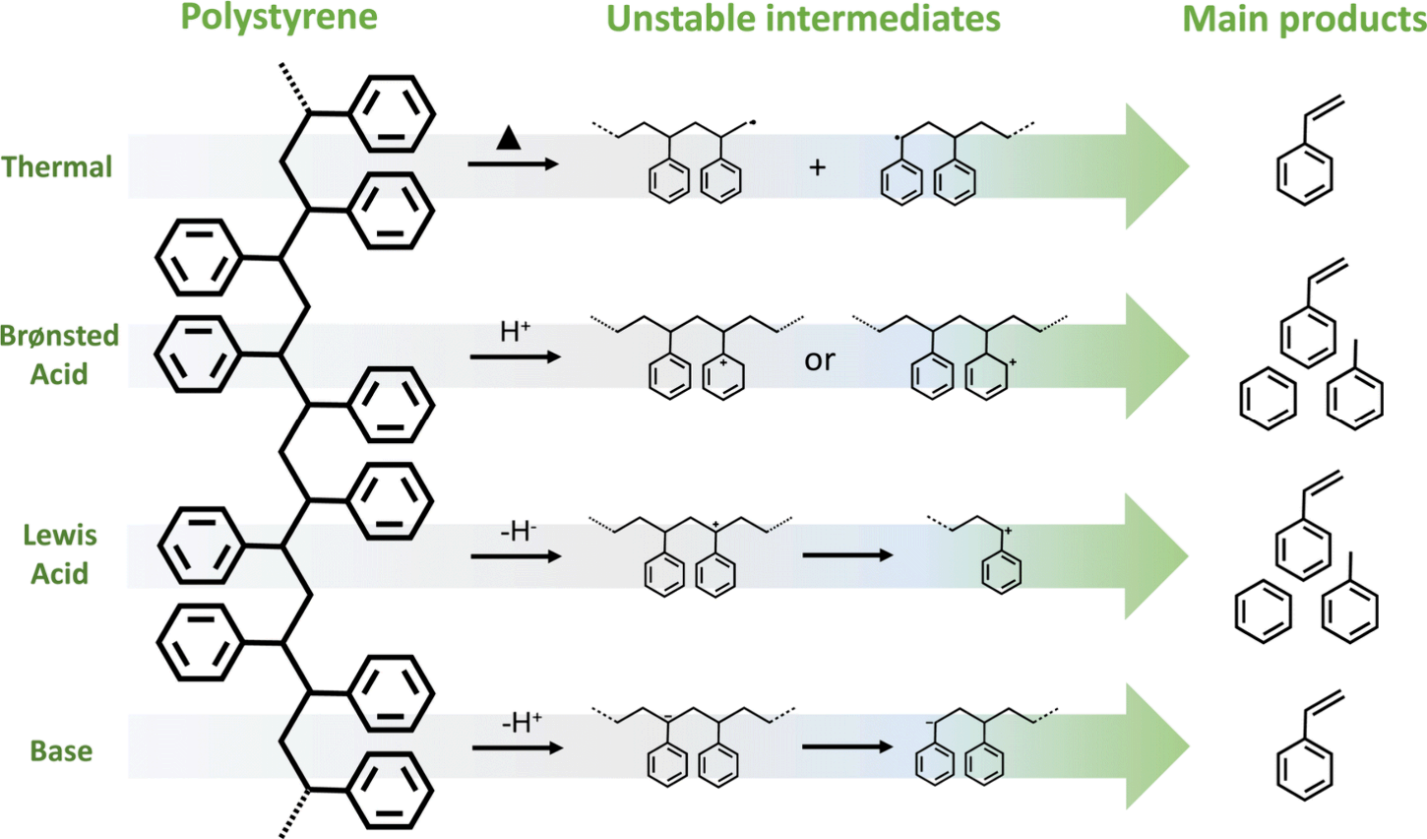
Comparison of Products Obtained from PS via Different Chemical Degradation Mechanisms Reproduced with permission from ref (265). Copyright 2023, Royal Society of Chemistry.

PS is one of the least recycled plastics, at 1 %.^265^ Unlike PE and PP, which generate a large number of products when pyrolyzed, PS pyrolysis can yield styrene monomer in moderate yields, typically 55–85 wt% at temperatures at or above 400 °C, Scheme 20.^265^ Non-catalytic depolymerization follows a radical mechanism.

Solid acid catalysts, including zeolites, silica-aluminas, aluminas, and their modified counterparts, have been used to convert PS at slightly lower temperatures, near 350 °C.^265^ BAS are proposed to protonate aromatic rings, forming arenium ions that can undergo hydride shifts, isomerization, C–C bond scission, and further cyclization of their alkyl substituents. The products are mostly monoaromatics (such as styrene, α-methylstyrene, benzene, toluene, ethylbenzene, cumene, and indane), and naphthalenes, as well as styrene dimer and its derivatives.^266^ Pore size and acidity influence the yield and selectivity.^265,267^ For example, the benzene yield in the depolymerization of PS (*M*_w_ = 35,000 g/mol, *Đ* unspecified) catalyzed by HY zeolite at 400 °C increased from 0 to 60 wt% with increasing zeolite acidity (achieved by varying the Si/Al ratio).^268^ Reminiscent of the catalytic cracking mechanisms for aliphatic POs, a few reports propose that Lewis acidic catalysts such as AlCl_3_ and Al_2_O_3_ generate carbenium ions by hydride abstraction from PS. However, the products resemble those from BAS-catalyzed reactions,^268^ and trace water can induce BAS formation in such catalysts, complicating the assignment of active sites.

PS can also be depolymerized using basic catalysts, including oxides of alkali metals (such as K_2_O), alkaline earths (such as MgO and BaO), and transition metals (such as ZnO and Co_3_O_4_) near 350 °C.^265^ The proposed reaction mechanism begins with H^+^ abstraction from a tertiary carbon. The resulting carbanion undergoes β-scission, producing mostly styrene monomer. To increase the active surface area, basic metal oxides can be dispersed on a high surface area silica support, as in K_2_O/MCM-41 and BaO-SBA-15.

PS hydrocracking using a bifunctional catalyst under H_2_ leads to the production of *n*-alkanes, *iso*-alkanes, alkenes, cycloalkanes, and aromatics.^269−271^ Although the depolymerization temperature can be further reduced to 300 °C, the added cost of H_2_ and the lower product selectivity has thus far limited the usefulness of PS hydrocracking. Nevertheless, PS depolymerization is of interest in PE and PP hydrocracking, since a PS impurity may impact catalyst robustness (discussed further in section 3.5.4).

##### 3.5.3.3 Effect of Chlorine

**Figure 44:**
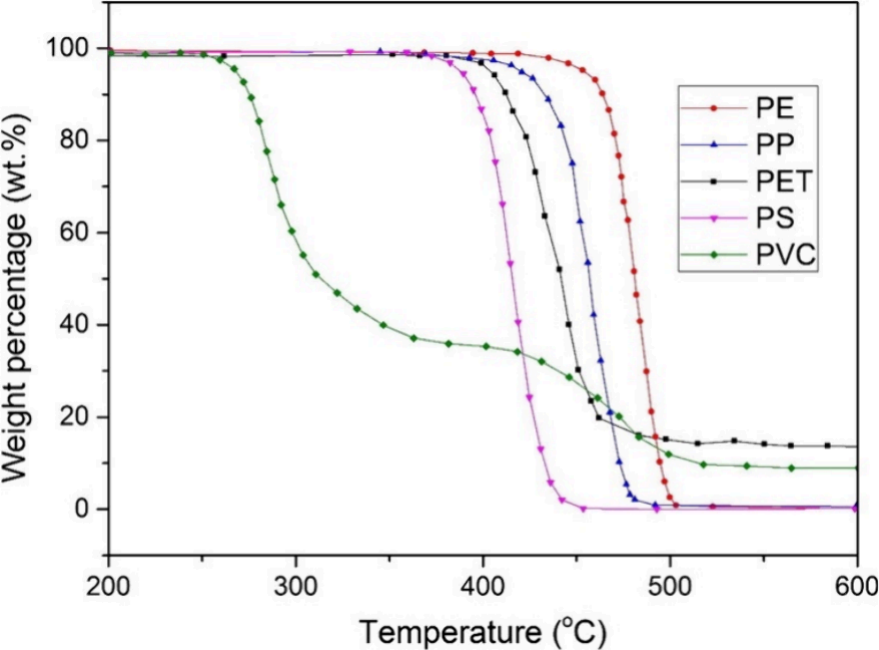
Thermogravimetric analysis of major POs as well as PET, recorded at 10 K min^-1^ under N_2_. Reproduced with permission from ref (25). Copyright 2016, Elsevier.

The thermal decomposition of PVC under N_2_ occurs at much a lower onset temperature (280 °C) than for PS, PP, or PE (all ≥400 °C), Figure 44.^25^ The lower thermal stability of PVC is due to its atacticity and the weak tertiary C–Cl bond, which readily undergoes homolytic cleavage. HCl can catalyze further dehydrochlorination (elimination of HCl, accompanied by C=C bond formation).^272^ PVC pyrolysis is characterized by two distinct mass loss events, while other POs typically show just one.^25^ The first stage, which accounts for 65 % of the weight loss, is dehydrochlorination which liberates HCl, accompanied by much smaller amounts of benzene, toluene, and other chlorinated and unchlorinated hydrocarbons. The second stage corresponds to cracking and decomposition of the dehydrochlorinated polymer, creating an appreciable amount of nonvolatile carbon residue.

Although this thermal dehydrochlorination is not particularly energy-intensive, the HCl product can cause reactor corrosion. In addition, chlorohydrocarbon contamination of the hydrocarbon products raises environmental concerns.^25^ To suppress these effects, basic metal oxides such as ZnO and MgO have been used as sorbents for HCl.^273^

PVC hydrocracking has not been extensively studied, but many studies have considered PVC because it is a likely feedstock impurity accompanying other waste POs, thereby justifying investigations of its effect on catalyst performance (discussed further in section 3.5.4). If the upcycling reaction involves a metal catalyst (e.g., for hydrogenolysis or hydrocracking), the catalyst may be poisoned by Cl coordination to metal sites.^106^ To suppress this effect, Cl can be mostly (≥ 90 %) removed by thermal hydrodechlorination, or by the nucleophilic substitution of chloride by hydroxide. For example, treatment of PVC with water or an aqueous alkaline solution at temperatures from 220 to 300 °C generated an aqueous solution containing chloride and solid hydrochar (a mixture of polycarbonyl, polyalcohol, and polyene structures).^274^

Chlorine can also be removed by catalytic dehydrochlorination. When NaOH or KOH was dissolved in ethylene glycol instead of water, HCl elimination was favored over hydroxyl substitution at elevated temperatures (60–180 °C), converting PVC into a polyene with a structure similar to polyacetylene.^275−277^ HCl elimination was even faster with organic bases such as tributylamine, NaNH_2_, and *t*-BuOK, allowing the reaction to proceed even at room temperature.^278−280^ The bases are soluble in polar organic solvents that also dissolve the polymer. Ionic liquids have also been used to induce HCl elimination from PVC.^281^ The resulting dehydrochlorinated residues were partially depolymerized by catalytic hydrogenolysis and/or hydrocracking.^179,282^ Polyenes can also undergo catalytic ethenolysis, producing 1,5-hexadiene and 1,6-heptadiene^281^ (discussed in section 4.3.5).

**Scheme 21:**
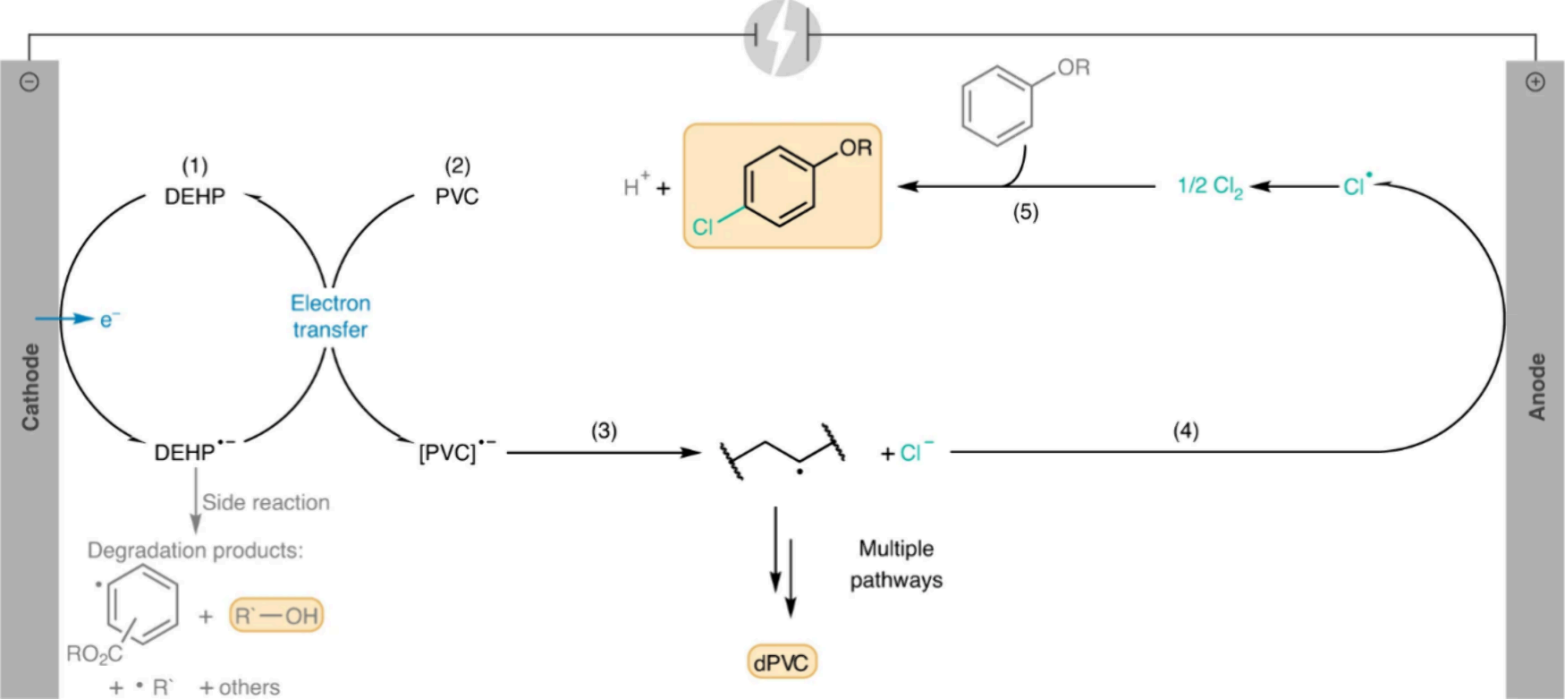
Proposed Mechanism for Electrochemical Dechlorination of PVC, Coupled to Chloroarene Synthesis Reproduced with permission from ref (283). Copyright 2023, Springer Nature.

Partial PVC dechlorination was accomplished electrochemically.^283^ To avoid the high overpotential for direct dechlorination, di(2-ethylhexyl)phthalate, a common PVC plasticizer, was used as a redox mediator. The chloride removed from PVC was oxidized to perform arene chlorination (Scheme 21). Using PVC as a chloride source for chloroarenes was deemed to have the potential to decrease greenhouse gas emissions by 71 %, compared to using HCl as a source of chloride for electrosynthesis. However, there was no comparison to the footprint of non-electrosynthetic arene chlorinations. The process was demonstrated with post-consumer PVC waste, although the dechlorination efficiency was low, with the Cl content decreasing only slightly from 57 to 44 wt% after 16 h electrolysis.

**Scheme 22:**
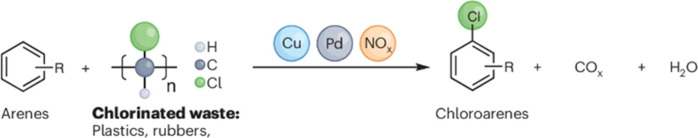
Tandem Catalytic Dechlorination of Chlorine-Containing Polymers, Accompanied by Formation of Chloroarenes Reproduced from ref (284). Copyright 2024, Springer Nature.

A thermal tandem catalysis process was reported to remove chlorine from PVC via Cu/NO_x_-catalyzed oxidation. PVC was mineralized to CO_x_ and H_2_O in the process, while the HCl product was used to chlorinate various arenes in the presence of a Pd catalyst (Scheme 22).^284^ After 15 h, no residual polymer was detected by GPC or ^1^H NMR. Other types of chlorine-containing polymers, including neoprene, polyepichlorohydrin (PECH), poly(epichlorohydrin-*co*-ethylene oxide) (PECHEO), and poly(epichlorohydrin-*co*-CO_2_) (PECHC), were all degraded successfully. The process was also used to synthesize the anticancer drug vismodegib. (Note, however, that t is doubtful that drugs made from waste are unlikely to pass necessary health and safety inspections.)

**Scheme 23:**
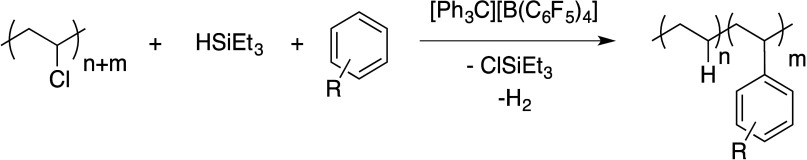
Tandem Hydrodechlorination/Friedel–Crafts Alkylation of PVC,Initiated by [Ph_3_C][B(C_6_F_5_)_4_]^286^

**Scheme 24:**
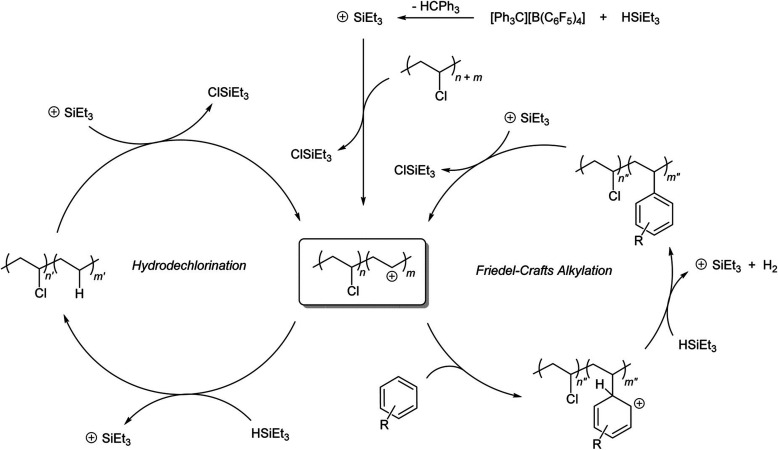
Proposed Catalytic Cycle for Tandem Hydrodechlorination/Friedel–Crafts Alkylation of PVC Initiated by [Ph_3_C][B(C_6_F_5_)_4_], Using Et_3_SiH as a Stoichiometric Reductant and an Aromatic to Trap the Carbocation; Adapted from Ref (286). Copyright 2023, Royal Society of Chemistry. Reproduced with permission from ref (286). Copyright 2023, Royal Society of Chemistry.

An alternative approach is the modification and functionalization of PVC by nucleophilic substitution of Cl with isothiocyanate, thiocyanate, or thiols, to make new polymer materials.^285^ PVC (0.136 g, 2.2 mmol monomer, *M*_n_ = 67,600 g/mol, *Đ* = 1.76) was fully dechlorinated at 100 °C by reaction with a stoichiometric amount of Et_3_SiH (2.4 mmol) in the presence of excess benzene (24 mmol). The tandem catalytic hydrodechlorination/Friedel–Crafts alkylation is shown in Scheme 23.^286^ The reaction is initiated by the trityl cation (Ph_3_C^+^, 0.011 mmol), which abstracts hydride from the silane to form a silylium ion (Et_3_Si^+^) that in turn removes chloride from the polymer. The resulting macromolecular carbocation can undergo either hydride abstraction from the silane, or a Friedel–Crafts reaction with benzene, which reacts with the silane to liberate H_2_. Both reactions regenerate the silylium ion (Scheme 24). The product is poly(ethylene-*co*-styrene) containing 20 mol% styrene. In addition to benzene, this strategy was demonstrated with toluene and various xylenes, and can also be performed in aromatic-free systems to generate a PE-like product.^485^

#### 3.5.4 Polyolefin Mixtures

To mimic the complexity of real plastic waste streams, simulated mixtures were created by combining different types of virgin plastics. Two types of PO mixtures were considered. The first was a mixture of PE (0.91–0.97 g cm^–3^), PP (0.85–0.94 g cm^–3^), and PS (0.96–1.05 g cm^–3^).^287^ These POs have similar enough densities that they are not fully separated by conventional sink-float separation in water.^288^ Although POs can be effectively separated from denser PVC (1.38 g cm^–3^), PET (1.34–1.39 g cm^–3^), poly(lactic acid) (PLA, 1.21–1.43 g cm^–3^), and polycarbonate (PC, 1.20 g cm^–3^) in this way, the presence of fillers, air bubbles, and additives in the plastics can reduce separation efficiency.^288^ Therefore, PE, PP, and/or PS contaminated with non-POs was the second type of mixture. Additives and fillers present in mixtures of post-consumer plastics are discussed in section 3.5.5.

The depolymerization of virgin PP and HDPE granules, catalyzed by a combination of Pt/WO_3_/ZrO_2_ and HY zeolite, was compared with the behaviors of formulated HDPE bottles, HDPE bags, and composite “tapes” consisting of 45 vol% PP, 45 vol% PE and 10 vol% PS.^212^ At 250 °C under 30 bar H_2_, each plastic gave mostly gasoline- and diesel-range hydrocarbons (60 to 85 wt %). The catalysts and process were therefore deemed capable of dealing with mixed PO streams. Similarly, POs such as LDPE, HDPE, PP, PS, were compared individually as well as in a mixture (whose composition reflected the relative global production amounts of each polyolefin in 2015).^213^ All were converted to liquid products in high yields (>80 wt%) by MoS_x_-HBeta at 250 °C under 20–30 bar H_2_ (Table 3). While the products obtained from PE and PP were mainly *iso*-alkanes (mostly C_4_–C_16_), PS was converted to a mixture of alkylbenzenes and alkanes (carbon numbers and structures not specified).

**Table 3:** Products from the Hydrocracking of Various Plastics and Plastic Mixtures, Catalyzed by MoS_x_-HBeta^a^

plastic	time (h)	non-solid yield (%)	product composition
LDPE, HDPE, or PP (each *M*_w_ = 300,000 g/mol)	6	85–96	*iso*-alkanes, C_4_–C_16_
PS (*M*_w_ = 120,000 g/mol)	16	87	alkylbenzenes and alkanes
mixed POs^b^	8	81	C_1_–C_4_ (10%), C_5_–C_7_ (38%), C_8_–C_12_ (23%), C_13_–C_16_ (6%), C_17+_ (1%), aromatics (3%)
PVC (*M*_w_ = 120,000 g/mol)	8	66	HCl, aromatics, and alkanes
PU (*M*_w_ = 120,000 g/mol)	16	71	THF and aliphatic acids
PLA (*M*_w_ = 120,000 g/mol)	16	89	oxygen-containing products
mixed plastics^c^	12	71	not reported

a Reaction conditions: 1 g plastic, *P*_H2_ = 25 bar, 250 °C, 10 wt% catalyst (except where noted).

b LDPE (*M*_w_ = 300,000 g/mol, *Đ* not specified, 31 wt%), HDPE (*M*_w_ = 300,000 g/mol, *Đ* not specified, 25 wt%), PP (*M*_w_ = 300,000 g/mol, *Đ* and tacticity unspecified, 32 wt%), and PS (*M*_w_ = 300,000 g/mol, *Đ* not specified, 12 wt%).

c LDPE (*M*_w_ = 300,000 g/mol, *Đ* not specified, 24 wt%), HDPE (*M*_w_ = 300,000 g/mol, *Đ* not specified, 20 wt%), PP (*M*_w_ = 300,000 g/mol, *Đ* and tacticity unspecified, 26 wt%), PS (*M*_w_ = 300,000 g/mol, *Đ* not specified, 10 wt%), PVC (*M*_w_ = 120,000 g/mol, *Đ* not specified, 5 wt%), PU (*M*_w_ = 150,000 g/mol, *Đ* not specified, 5 wt%), PLA (*M*_w_ = 60,000 g/mol, *Đ* not specified, 5 wt%), PC (*M*_w_ = 45,000 g/mol, *Đ* not specified, 5 wt%), 15 wt% catalyst, relative to plastic.

Reproduced with permission from ref (213). Copyright 2023, AAAS.

MoS_x_-HBeta appears to tolerate many heteroatoms, including Cl, O, and N. PVC and other heteroatom-containing polymers like polyurethanes (PUs), PC, and PLA, and a mixture of POs with these polymers, were also successfully converted to smaller molecules (>70 wt%) at 250 °C under 25 bar H_2_ by MoS_x_-HBeta, Table 3.^213^ However, the products were incompletely characterized.

A small amount of PVC (1 %) mixed with PP was reported to deactivate the hydrocracking catalyst Ni/TiO_2_-A-SG (TiO_2_-A-SG refers to anatase prepared by a sol–gel route),^48^ as well as various Ru-based hydrogenolysis catalysts.^48,106,179^ HZSM-5 with a controlled *b*-axis length of 80–100 nm was used to catalyze the cracking of a supermarket shopping bag (composition unspecified), a plastic film (composition unspecified), and a mixture of PE (96 wt%, described as having an average molecular weight 150,000 g/mol without indicating *M*_n_, *M*_w_, or *Đ*) with PVC (4 wt%, molecular weight and *Đ* unspecified). Under flowing H_2_, C_1_–C_7_ hydrocarbons were 60-90 wt% of the product mass, with 60-80 % of this yield (presumably wt%, but not specified) being alkenes.^209^ These products are similar to those obtained from PE alone, indicating little effect of additives or PVC on catalyst deactivation. Unfortunately, the fate of Cl was not discussed. Metal-free HZSM-5 may be more tolerant of Cl impurities than metal-based catalysts.

In conclusion, processes designed for handling mixed plastics will need to tolerate impurities in order to minimize separation costs. However, avoiding feedstock separations will increase variability in product compositions. More studies of the reactions of mixed plastics and post-consumer plastics, as well as more informative product analyses, will be required to understand these challenges.

#### 3.5.5 Additives and Impurities

Additives are widely used in plastics to improve their performance but are feared to influence chemical upcycling of POs.^15,17^ Globally, 10,000 individual chemical substances were produced as additives in 2015.^1^ Their total mass, 25 MMT, is equivalent to world PS production. Typical additives typically belong to one or more of eight categories: fillers (e.g., clays, glass, carbon black, up to ∼50 wt%), plasticizers (e.g., di-*n*-octyl phthalate, *tris*(2-chloroethyl)phosphate, 10–70 wt%), flame retardants (e.g., poly(bromodiphenyl ethers), 10–20 wt%), colorants (e.g., inorganic pigments, including oxides, sulfides, and metals; organic dyes, 1–4 wt%), UV stabilizers (e.g., hindered amines or benzophenones), thermal stabilizers (e.g., dialkyl maleates, 0.1–8 wt%), processing aids (waxes, 0.5–2 wt%), and others (e.g., anti-statics, biocides, <2 wt%).^289^

**Scheme 25:**
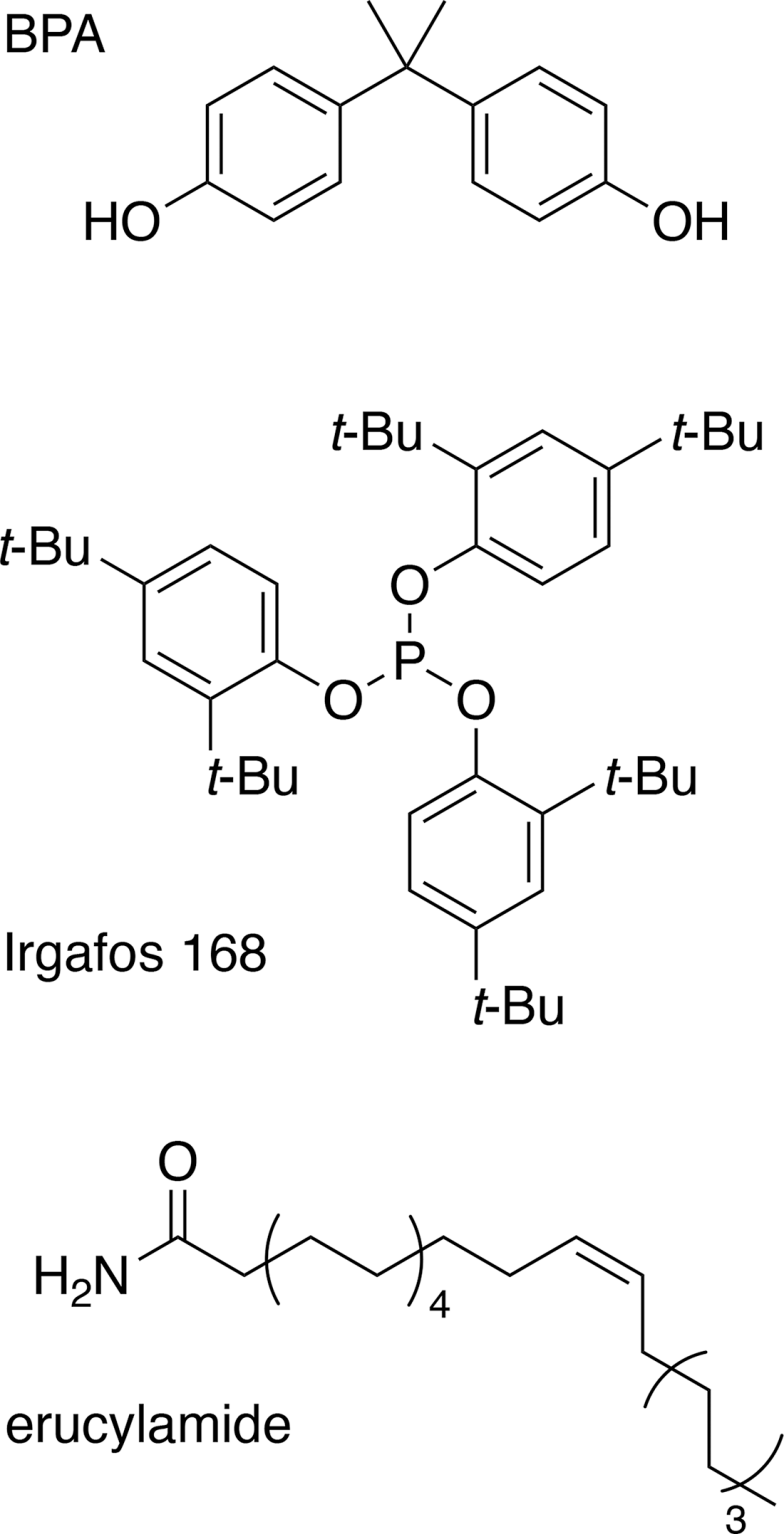
Chemical Structures of Three Common Polymer Additives: Antioxidant BPA, Antioxidant Irgafos 168, and Slip Agent Erucylamide

The effects of three common additives (Scheme 25) on hydrocracking catalyzed by MoS_x_-HBeta was examined.^213^ At loadings of 0.5 to 1 wt%, their effects on catalytic activity were limited, suggesting that the catalyst tolerates certain functional groups, including phenols, amides, and phosphates. However, excess amounts may poison the catalyst. At 5 wt% loading, erucylamide caused the non-solid yield to decrease by 61 %, presumably due to competitive binding of amine groups to the acid sites, and thereby changing the balance between cracking and hydrogenation.

**Figure 45:**
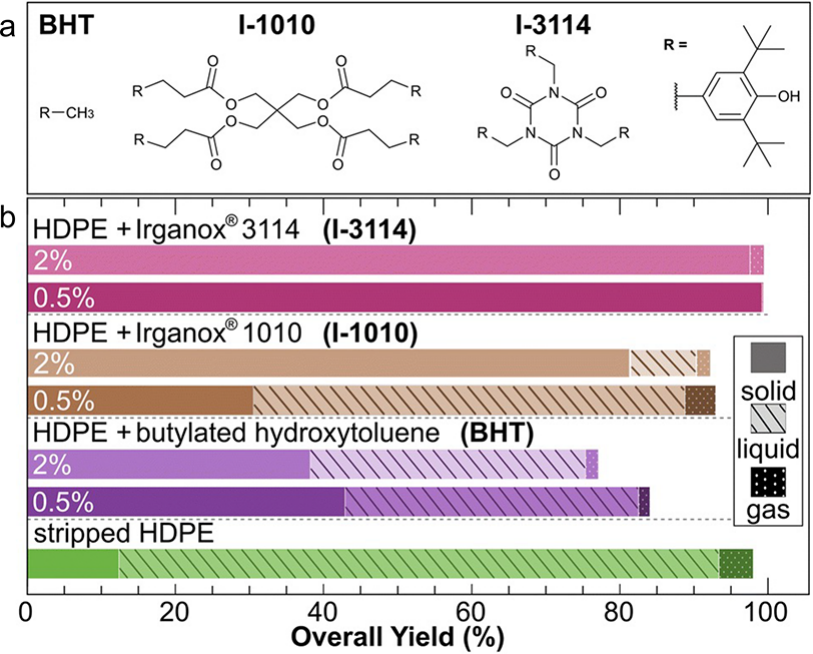
(a) Chemical structures of three common antioxidants; and (b) overall yields of solid, liquid, and gas products for all antioxidant-containing HDPE samples (at 0.5 and 2 wt% loadings of antioxidant) and for HDPE depleted of additives. Reaction conditions: 250 °C, 30 bar H_2_, 2 g HDPE, 50 mg Pt/WO_3_/ZrO_2_, 2 h. Reproduced with permission from ref (290). Copyright 2022, Royal Society of Chemistry.

To investigate the effect of additives on hydrocracking catalyst performance, additives were first removed from a commercial HDPE (*M*_w_ = ca. 90 kg mol^–1^) by Soxhlet extraction.^290^ Pt/WO_3_/ZrO_2_ (1 wt% Pt, 25 wt% WO_3_) converted the stripped HDPE with a much higher liquid yield (80 wt%) compared to the as-received HDPE (20 wt%). Hindered-phenolic antioxidants and fatty-amide slip agents were proposed to cause the lower hydrocracking activity. The effects of three phenolic antioxidants (BHT, I-1010, and I-3114, Figure 45) on hydrocracking were investigated further. Both antioxidant concentration and chemical structure appear to affect hydrocracking outcomes. For the BHT additive, varying the loading in the range 0.5–2 wt% had little effect on catalyst activity, suggesting that the catalyst surface was already saturated. The ester groups of Irganox 1010 were deemed likely responsible for a more significant decrease in activity (less liquid, more solid, Figure 45) with increased loading. Finally, no conversion was observed in the presence of Irganox 3114, presumably because the cyanurate poisons the active sites completely. Further systematic studies will be required to elucidate the roles of various functional groups and structures of antioxidants.

**Figure 46:**
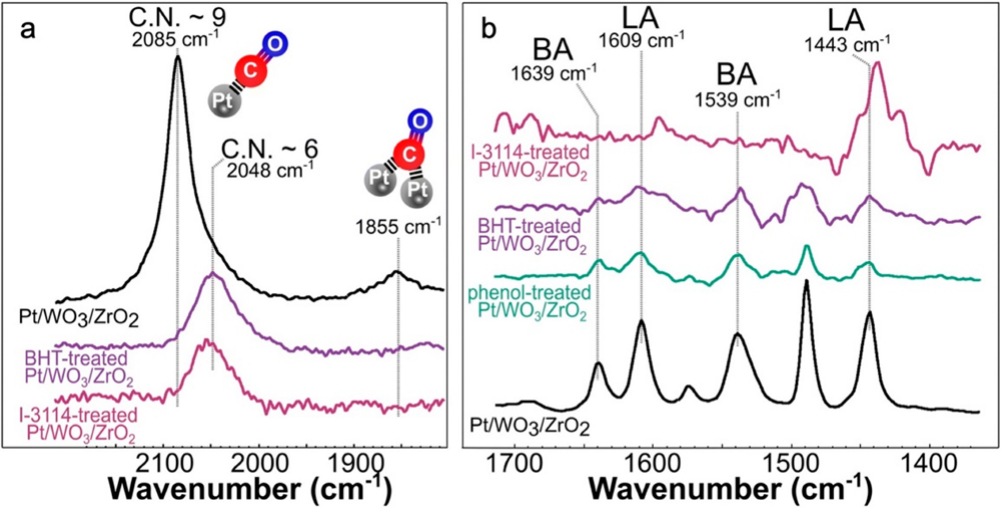
DRIFTS characterization of Pt/WO_3_/ZrO_2_ catalysts with and without adsorbed antioxidants: (a) CO adsorption at 35 °C shows a reduction in Pt coordination number; and (b) pyridine chemisorption on acid sites at 150 °C, showing changes in the relative concentrations of Lewis acid (LA) and Brønsted acid (BA) sites. Reproduced with permission from ref (290). Copyright 2022, Royal Society of Chemistry.

Activity was linked to antioxidant-mediated interactions with the bifunctional catalyst using DRIFTS. Specifically, phenolic groups adsorbed on the facets of Pt nanoparticles, leaving only undercoordinated edges and corner sites of the nanoparticles accessible to CO (Figure 46a). The ester groups of I-1010 may transesterify OH sites on WO_x_, reducing the number of Brønsted acid sites, while I-3114 reacts completely with the acid sites (Figure 46b). Consequently, antioxidants can change the effective metal-to-acid site ratio (MAB) through adsorption, depending on their functional group profile (e.g., phenols, acids, esters). However, since adsorption measurements were conducted at room temperature in the absence of polymer, the conditions differed significantly from the depolymerization conditions.

In addition to additives incorporated during polymer formulation, impurities can also contaminate plastics during the use and disposal phases. The catalytic performance of s-ZSM-5 (ZSM-5 zeolite with a Si/Al ratio of 21 and a *b*-axis length of 80–100 nm) was tested witha waste plastic stream rich in films, consisting of PE (93 wt%, *M*_n_, *M*_w_, or *Đ* unspecified) and various impurities (7 wt%, including Al powder, PET, and glass).^209^ At 280 °C under flowing H_2_ (3.3 %, balanced with Ar and N_2_), the products were mostly C_1-7_ (64 wt%), of which 83% were alkenes, similar to the results from a model PE (described as having an average molecular weight 150,000 g/mol without indicating *M*_n_, *M*_w_, or *Đ*).

**Figure 47:**
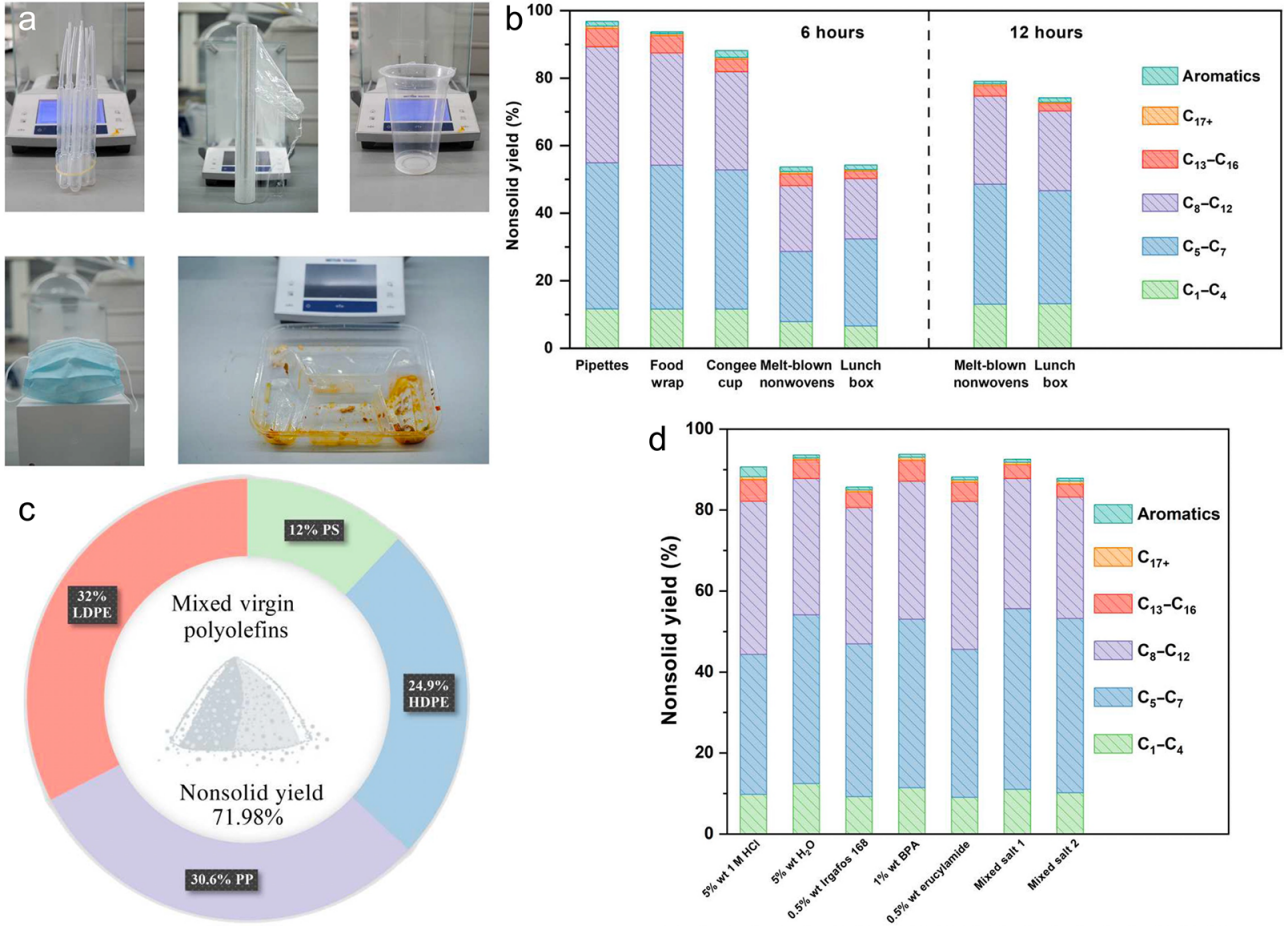
Hydrocracking of post-consumer plastic waste. (a) Photographs of the post-consumer plastics used in (b). The disposable cup and tray were not cleaned prior to reaction. (b) Non-solid yields from catalytic hydrocracking of plastic mixture (1.0 g) with MoS_x_-HBeta (0.100 g) at 250 °C under 25 bar H_2_, after 6 and 12 h. (c) Hydrocracking of a mixture of virgin polyolefins (1.0 g) with MoS_x_-HBeta (0.100 g) at 250 °C under 25 barH_2_, after 6 h. (d) Non-solid yields from catalytic hydrocracking of LDPE (1 g, *M*_w_ = 300,000 g/mol, *Đ* unspecified) in the presence of MoS_x_-HBeta (0.100 g) and various impurities (mixed salt 1 is 50 mg each of CaCO_3_, FeCl_3_, MgSO_4_, and KBr; mixed salt 2 is 50 mg each of Na_2_CO_3_, PbSO_4_, ZnCl_2_, and CuBr_2_), at 250 °C under 25 bar H_2_ for 6 h. All values were calculated on a carbon basis. Reproduced with permission from ref (213). Copyright 2023, AAAS.

The influence of moisture, mineral acid, and various inorganic salts on the hydrocracking of LDPE (*M*_w_ = 300,000 g/mol, *Đ* unspecified) catalyzed by MoS_x_-HBeta was examined at 250 °C under 20-30 bar H_2_.^213^ There was no noticeable decline in catalytic activity in the presence of H_2_O or a 1 M HCl solution (5 wt% each), Figure 47. This resistance could be a consequence of the protection offered by hydrophobic LDPE and its depolymerization products. Various inorganic salts were also merely spectators. However, an NaOH solution or HF, both of which are expected to destroy the zeolite structure, had highly detrimental effects. A cup and tray containing food residue gave non-solid yields of 88 and 54 wt%, respectively (Figure 47a, b). The non-solid yield of the tray increased further, to 96 wt%, after 12 h. (Since the food content was not reported, reproducing these experiments would be challenging.)

In summary, phenolic groups adsorb on metal sites, while esters, amides, and cyanurates occupy and/or react with acid sites. In some cases, it may be necessary to pretreat plastic waste to remove additives containing these functional groups in order to protect the catalyst. Alternatively, future research could focus on designing additives that are less detrimental to catalysts, and on developing depolymerization strategies that tolerate a wider range of real-world plastic additives and impurities.

### 3.6 Hydrogen Redistribution

Plastic waste is generally highly dispersed, and its collection, sorting and transportation to a processing site incur significant costs. A preliminary techno-economic analysis showed that producing gasoline from POs through hydrocracking is not likely to be profitable at a small plant scale (e.g., 5 kton/year).^291^ Aromatics may be more attractive depolymerization targets due to their higher intrinsic value, relative to fuels.^292^

#### 3.6.1 Mechanism of Aromatics Formation

##### 3.6.1.1 Catalytic Reforming

**Scheme 26:**
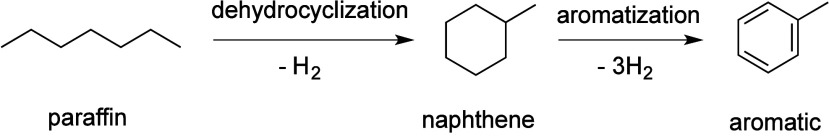
Two Main Reactions in Naphtha Catalytic Reforming

Monocyclic aromatic hydrocarbons such as benzene, toluene, and xylenes (the mixture is known as BTX) are important chemical building blocks. In 2020, the global demand for BTX was ca. 108 MMT, and it is projected to increase at a compound annual growth rate of 5 % in coming years.^293^ The conventional route for producing BTX involves catalytic reforming of naphtha (which consists of mostly C_6_–C_10_ alkanes), typically at ca. 500 °C. The major reactions include alkane dehydrocyclization to cycloalkanes, and further dehydrogenation of the cycloalkanes to aromatics. Both reactions generate H_2_ as a byproduct, Scheme 26. Since the use of POs to make molecular aromatics shares much in common with naphtha reforming, we will briefly review the relevant catalytic reactions involving smaller hydrocarbons.

**Scheme 27:**
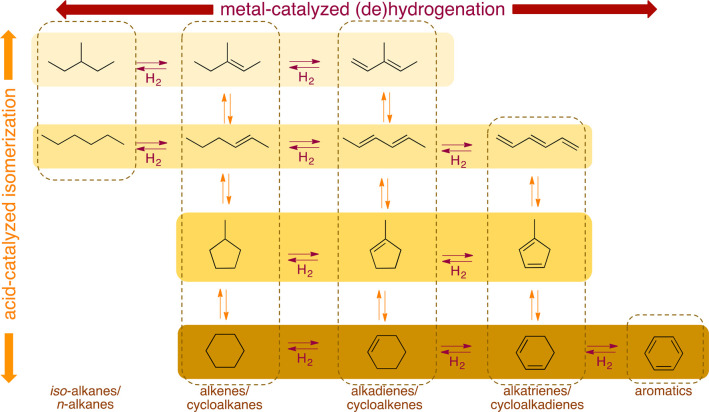
Proposed Mechanism for Bifunctional Catalytic Reforming, Involving Alkane (De)Hydrogenation, Isomerization, Dehydrocyclization and Aromatization Horizontal reactions represent H_2_ transfers; vertical reactions are skeletal rearrangements. Redrawn with permission from ref (230). Copyright 2010, Wiley.

A traditional catalyst for naphtha reforming is bifunctional, consisting of Pt nanoparticles supported on an acidic alumina.^294^ Similar to the hydrocracking mechanism, the metal sites are proposed to catalyze (de)hydrogenation, while the acid sites are proposed to catalyze skeletal transformations, including isomerization, the cyclization of alkenes to cycloalkanes, and C–C bond scission (Scheme 27). Cyclization is the key reaction forming aromatic rings. Initially, the cyclization of an alkene or alkadiene produces either a five- or six-membered ring.^295^ Although only the latter is dehydrogenated to give an aromatic directly, ring expansion of five-membered rings is rapid under reforming conditions.^296−301^

Skeletal isomerization of *n*-alkanes is desired in catalytic reforming because *iso*-alkanes have higher octane numbers, which are important for gasoline performance. However, C–C bond scission is undesirable, because it generates light gases with much lower value. In general, catalytic rates decrease in the following order: (de)hydrogenation > isomerization > dehydrocyclization ∼ C–C bond scission.

Monofunctional catalysts are also used commercially in catalytic reforming. For example, Pt/KL (where KL is K^+^-exchanged zeolite L) is used in the Aromax process to convert *n*-hexane to benzene at ca. 500 °C.^302^ The non-acidic nature and specific pore geometry of KL contributes to the selectivity, as well as conferring resistance to coke formation and Pt sintering.^303−306^

**Scheme 28:**
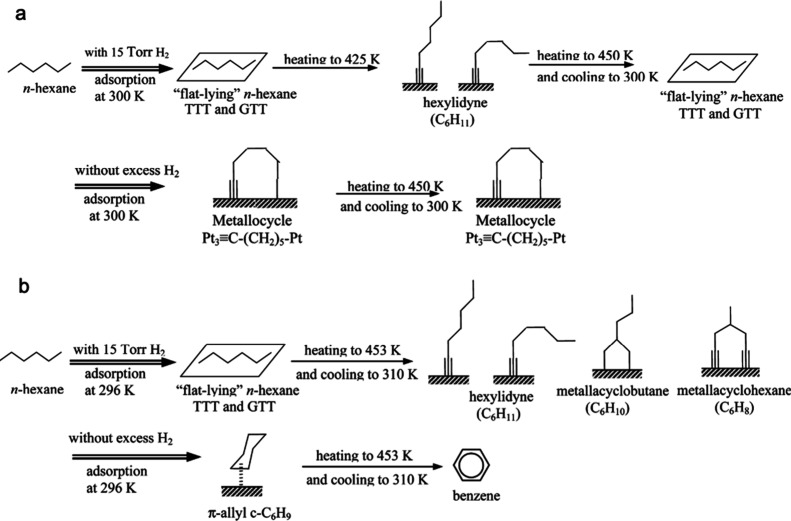
Hydrocarbon Species Observed on (a) Pt(100); and (b) Pt (111), After Exposure to 1.5 Torr *n*-Hexane in the Presence or Absence of H_2_ Reproduced with permission from ref (308). Copyright 2007 American Chemical Society.

Metal sites on single-crystal surfaces catalyze isomerization and cyclization of *n*-alkanes, in the absence of acidity.^307,308^ Adsorption geometries and surface reactions of *n*-hexane on Pt(100) and Pt(111) surfaces were investigated using sum-frequency generation vibrational spectroscopy. Initially, *n*-hexane adsorbs in a “flat-lying” conformation, Scheme 28. Increasing the surface temperature causes dehydrogenation of this intermediate to adsorbed hexylidyne. In the absence of low pressure H_2_, several other adsorbates coexist: a metallacyclic Pt_3_≡C–(CH_2_)_5_–Pt on Pt(100) and a π-allylic form of cyclo-C_6_H_9_ on Pt(111). The latter is significant, because it leads directly to benzene.

##### 3.6.1.2 Polyolefin Conversion to Molecular Alkylbenzenes

Because various hydrocarbon types, including alkenes, isolated and conjugated alkadienes, are present during PO depolymerization, there are two possible mechanisms for aromatic formation. In the first, a benzene ring is formed by cyclization of an alkene or alkadiene, similar to the mechanism for small hydrocarbons shown in Scheme 27. The second is a Diels–Alder reaction of an alkene with an alkadiene (both derived from PE cracking), giving a benzene ring by cycloaddition.^309^

**Scheme 29:**
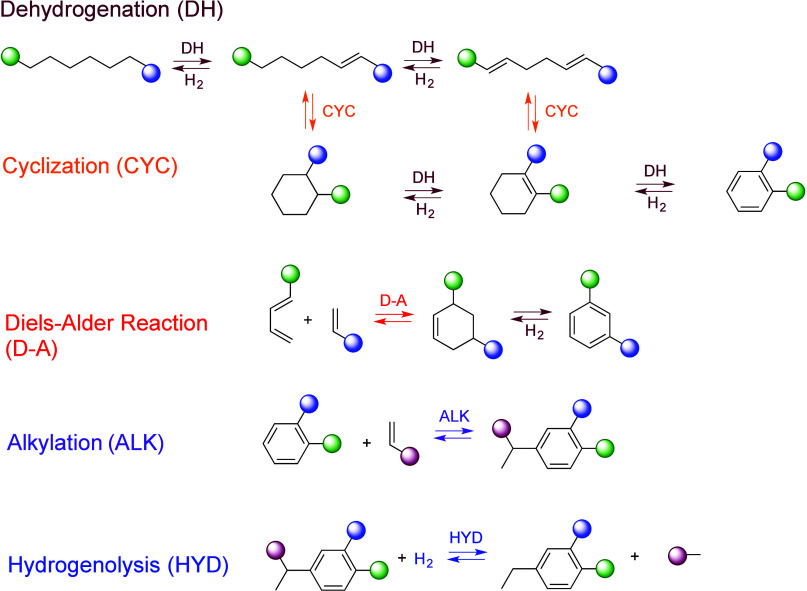
Some Proposed Reaction Pathways in PE Conversion to Alkylaromatics The colored circles represent alkyl chains of varying lengths.

In principle, aromatic ring formation can occur on either long or short alkane chains, depending on thecatalyst and reaction conditions. Other possible reactions include the alkylation of aromatic rings by alkenes present in the reaction mixture, and the shortening of alkyl substituents on aromatic rings by C–C bond scission. All reactions occurring in parallel generate alkylaromatics with a range of molecular weights, Scheme 29.

#### 3.6.2 Monofunctional Catalysts

Unlike naphtha catalytic reforming, which employs monofunctional metal catalysts or metal–acid bifunctional catalysts, the most commonly reported catalysts for producing aromatics from alkenes are solid acids, particularly zeolites. Zeolites generate small aromatics (C_6_–C_8_, BTX) by catalytic cracking of PE and PP under an inert atmosphere at temperatures between 400 and 500 °C.^191^ Their activity and aromatic selectivity are attributed to their strong acidity and shape-selective micropores, respectively.^191^

Catalyst acidity is important in maximizing the aromatic yield, presumably via a mechanism similar to that described in Scheme 27, but without the metal function. For example, decreasing the acidity of HZSM-5 (1.5 g), either by increasing the SiO_2_/Al_2_O_3_ ratio (from 23 to 80) or by NaOH pretreatment, led to a reduction in aromatic selectivity in the liquid products obtained from catalytic cracking of LDPE (10.0 g, molecular weight unspecified) at ca. 450 °C under N_2_.^310^

A mesoporous silica-alumina (0.200 g) converted a low molecular weight PE (0.130 g, *M*_w_ = 3.52 × 10^3^ g/mol, *Đ* = 1.9) to alkylaromatics (carbon numbers unspecified) at 280 °C under Ar.^311^ The silica content (from 1.5 to 30 wt%) was varied while maintaining a similar surface area (ca. 200 m^2^/g) and mesopore diameter (ca. 10 nm). Both the rate of chain scission and the formation of aromatics were correlated with the number of BAS. The fraction of polyaromatics, measured at similar aromatic contents (ratios of aromatic protons to total protons, *H*_arom_/*H*_total_), also increased with the number of BAS. Compared to Pt/Al_2_O_3_, silica-alumina produced a similar amount of CHCl_3_-soluble hydrocarbons, a similar *H*_arom_/*H*_total_ in these products, and a similar molecular weight distribution for the total hydrocarbons (as evidenced by GPC-RI), although the molecular weight distribution of the aromatic products was not necessarily the same as for other types of hydrocarbons. Nevertheless, this study indicated that a strongly acidic catalyst without a metal can produce significant amounts of aromatics, similar to a weakly acidic catalyst with a strong dehydrogenation function.

The zeolite microstructure also plays a role in determining the carbon number distribution in the aromatic products. For example, HZSM-5 with its 10-membered ring channels (pore diameter ca. 0.5 nm) produced primarily C_7_–C_8_ aromatics from catalytic cracking of LDPE (viscosity-average molecular weight, *M*_v_ = 298,000 g/mol, *Đ* = 4.6) at 480 °C under N_2_, while C_10_–C_11_ aromatics were the main products from HY, whose 12-membered ring channels have a larger diameter of 0.7 nm.^312^

Activated carbon was reported to convert PE to aromatics in a fixed bed flow reactor at 526 °C. Molten PE (viscosity-average molecular weight *M*_v_ =1.3 × 10^4^ g/mol, *Đ* unspecified) was forced through a capillary under N_2_ pressure. In addition to alkanes (C_1_–C_10_, 56 wt%) and alkenes (C_2_–C_8_, 6 wt%), a significant amount of BTX aromatics (37 wt%) was formed. Under the same conditions, the aromatic yield was only 10 wt% when silica-alumina (13.5 wt% Al_2_O_3_) was the catalyst.^313^ The proposed mechanism over activated carbon was proposed to involve radicals rather than carbocations, due to the low yield of branched alkanes.

Treating carbon derived from wood chips with H_3_PO_4_ enhanced the formation of aromatics in a dual-stage fixed-bed reactor. In the first stage, PE (10 g, molecular weight unspecified) was simply pyrolyzed at 550 °C under N_2_ to alkanes and alkenes (carbon number unspecified). In the second stage, these compounds were exposed to the catalyst (4 g) at 600 °C. The aromatic (C_7_–C_10_) yield of the products increased from 0 to 24 wt% when the P-to-wood chip (carbon source) mass fraction increased from 0 to 40 %.^314^

#### 3.6.3 Metal-Assisted Hydrogen Redistribution

##### 3.6.3.1 Making Methylated Aromatics

Transformation of POs to mixtures of small-molecule alkanes and aromatics requires hydrogen redistribution. Acid sites alone are typically not efficient in catalyzing (de)hydrogenation, therefore metal sites are usually present. For example, Pt/Al_2_O_3_ is used in naphtha catalytic reforming to BTX, while Ga/ZSM-5 is the Cyclar catalyst that converts LPG (liquefied petroleum gas, a mixture of propane and butanes) to BTX.^315,316^

Several studies have investigated the use of supported metal catalysts for the conversion of PE to small molecule aromatics in fixed-bed tubular reactors under a continuous flow of inert gas (e.g., He or N_2_) at ca. 500 °C. One study forced molten PE through a capillary by a flow of inert gas. Metal addition, e.g., Pt, Fe, and Mo, to supports like activated carbon, alumina, and silica-alumina resulted in higher activity compared to the corresponding support alone in the conversion of PE (*M*_v_ = 1.3 × 10^4^ g/mol, *Đ* unspecified) to aromatics at 526 °C.^317^ H-gallosilicate gave higher selectivity to aromatics (BTX, 60 wt%) from LDPE (molecular weight not specified), compared to just 34 wt% with Al-containing HZSM-5 at 525 °C (*W*/*F* = 0.12 h, where *W* is the catalyst mass, and *F* is the mass flow rate of molten LDPE through the capillary).^318^ Ion-exchange of HZSM-11 with Zn^2+^ gave a catalyst that produced aromatics (C_6_–C_9_) in 53 wt% yield at 500 °C, compared to 27 wt% with the original HZSM-5.^319^ Pt/ZSM-5 (1.4 wt% Pt, Si/Al = 38) was reported to reduce the onset temperature for forming BTX aromatics from 310 to 270 °C, compared with the zeolite alone, in the reaction of PE (*M*_w_ = 4,000 g/mol, *Đ* = 2.4). In that study, PE and catalyst (*m*_PE_/*m*_catalyst_ = 1:6) were heated together in a stainless steel cup in a micropyrolyzer under flowing He.^320^

**Figure 48:**
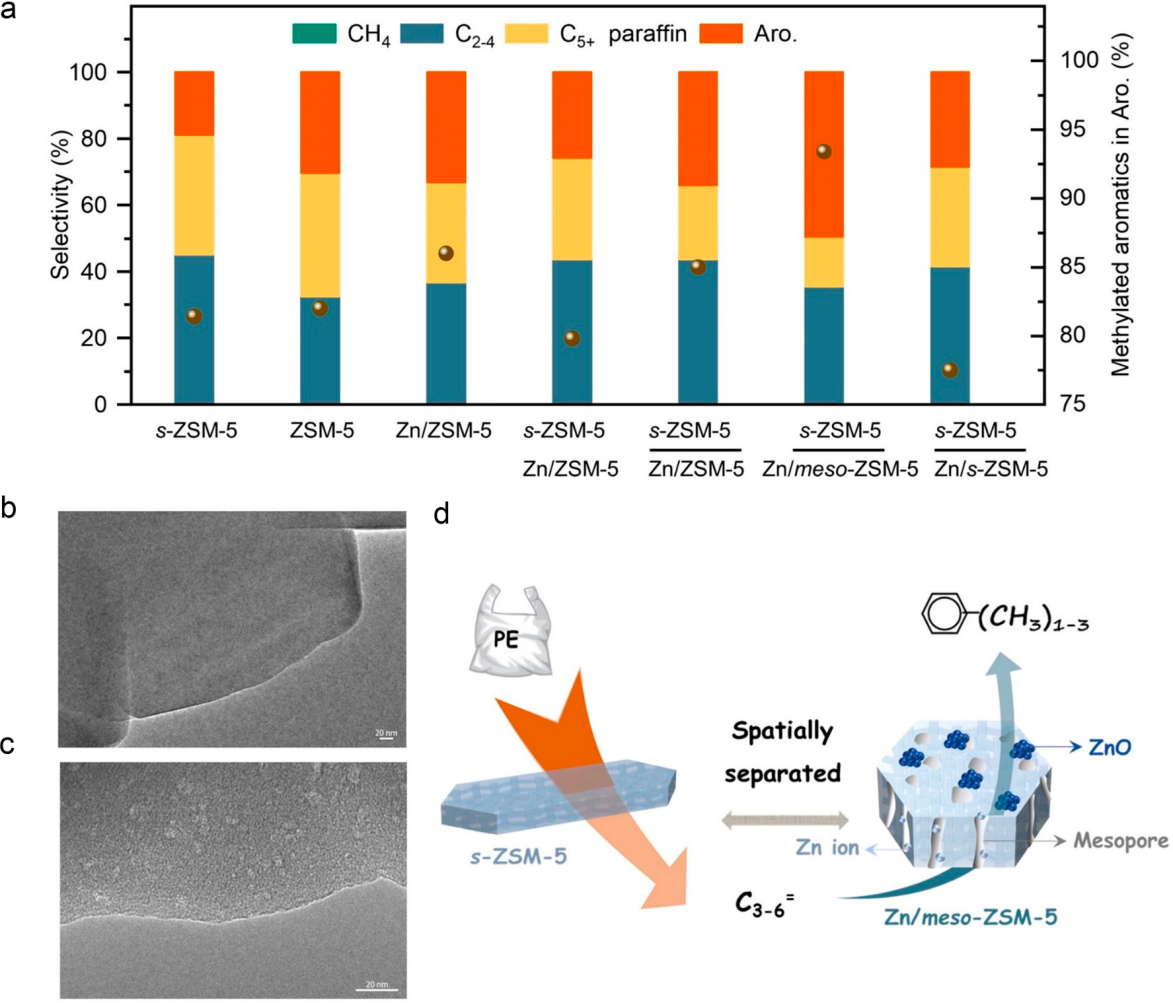
Top: (a) Product selectivity in PE aromatization after 4 h at 400 °C with various ZSM-5-based catalysts, configured in either single or dual beds. For the single-bed systems (first four bars), a mixture of PE (500 mg) and s-ZSM-5 (100 mg), ZSM-5 (100 mg), Zn/ZSM-5 (400 mg), or a mixture of s-ZSM-5 (100 mg) and Zn/ZSM-5 (400 mg), were combined in one bed. For the dual-bed systems (last three bars), a mixture of PE (500 mg) with s-ZSM-5 (100 mg) was placed in the first bed, while the Zn-containing aromatization catalyst (400 mg) was placed in the second bed. H_2_ (3.3 %) balanced with Ar/N_2_ flowed through both beds at 3 mL min^-1^. A small amount of C_5+_ alkene products is included with the alkane products. Bottom: TEM images of (b) ZSM-5, and (c) meso-ZSM-5. (d) Schematic diagram of hydrocarbon transport through the dual catalyst beds. Reproduced with permission from ref (42). Copyright 2023, Royal Society of Chemistry.

Metals are not necessary if the reaction temperature is high enough. Use of a ZSM-5 catalyst with a nanosheet morphology (s-ZSM-5, having a *b*-axis thickness of ca. 100 nm) to depolymerize PE (*M*_n_ = 28,000 g/mol, *Đ* unspecified), under a flow of 3% H_2_ (balanced with Ar/N_2_) suppressed coking by facilitating rapid diffusion of alkene products at 280 °C.^209^ Increasing the temperature to 400 °C led to the formation of aromatics. The products included C_2_–C_4_ alkanes and alkenes (45 wt%, 91% selectivity to alkenes, molar or mass selectivity unspecified), C_5+_ alkanes and alkenes (35 wt%), and various methylated aromatics (20 wt%, C_7_–C_10_), Figure 48.^42^ A commercial ZSM-5 (Si/Al =12) gave a higher aromatic yield (30 wt%), likely due to the longer residence time of alkenes in the catalyst pores. Adding ZnO_x_ (ZnO_x_/ZSM-5, 3.0 wt% Zn) resulted in an even higher aromatic yield and selectivity for methylated aromatics. This outcome is expected, since similar catalysts are known to be effective in the aromatization of alkanes and alkenes.^321^

Industrial catalytic reforming of naphtha commonly uses multiple reactors, such that an individual catalyst bed can be isolated from the main process flow, regenerated, and put back into service without shutting down the process.^322^ This configuration also allows each reactor to be loaded with a different catalyst, if desired. A cascade conversion of PE to variously methylated benzenes was developed using a dual-bed reactor.^42^ The first bed, containing s-ZSM-5 (0.100 g), converted PE (0.500 g, *M*_n_ = 28,000 g/mol, *Đ* unspecified) to low molecular weight alkenes, while the second bed, Zn/meso-ZSM-5 (0.400 g, 3.0 wt% Zn on meso-ZSM-5), transformed the alkenes to aromatics. Both beds were held at 400 °C under a flow of 3% H_2_ balanced with Ar/N_2_. After 4 h, the process gave aromatics in 50 wt% yield. These aromatics consisted mostly of toluene, xylenes, and mesitylene (94 %, relative to all aromatic products), Figure 48.

Physically mixing s-ZSM-5 with Zn/ZSM-5 gave worse aromatic yields, compared to Zn/ZSM-5 alone. In contrast, spatial separation of s-ZSM-5 and Zn/ZSM-5 in two catalyst beds was more effective. In the catalyst configuration optimal for aromatics production, s-ZSM-5 was located in the first catalyst bed and Zn/meso-ZSM-5 in the second. The mesoporous zeolite produced less coke, as evidenced by TGA and Raman analysis, presumably due to faster mass transport. Furthermore, the coke required a lower temperature for oxidative removal post-reaction compared to the coke formed in microporous Zn/ZSM-5. However, Zn/s-ZSM-5 showed a lower yield and selectivity for aromatics, presumably due to insufficient retention of alkenes in the zeolite pores. Therefore, Zn/meso-ZSM-5 strikes a balance between minimizing coke formation and maximizing aromatization.

Various post-consumer PO products, including supermarket shopping bags, deep-freeze food pouches, and PE reagent bottles, were converted to volatile hydrocarbons (89–92 wt%) including methylated aromatics (42–47 wt%, relative to the initial charge of PE) using a dual bed system pairing s-ZSM-5 and Zn/ZSM-5 catalysts. The process tolerated PVC (10 wt%) or water (amount unspecified), without compromising catalytic performance. Under similar conditions and at comparable reaction times, PP was converted to volatile hydrocarbons in similar yield (90 wt%) with a lower aromatics fraction (35 wt%, relative to the initial PP charge). Control experiments using s-ZSM-5 as the catalyst gave heavier products from PP, relative to PE. The lower activity of s-ZSM-5 in converting PP to aromatics (35 wt% yield) compared to PE to aromatics (50 wt% yield) was attributed to the greater bulkiness of highly branched intermediates from the PP reaction, compared to those from the PE reaction. The PP intermediates were presumed to have less access to the zeolite micropores.

##### 3.6.3.2 Making Long-Chain Alkylaromatics

**Scheme 30:**
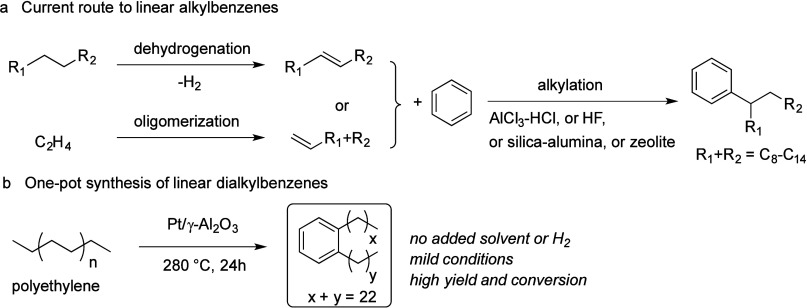
Comparison of Routes to Linear Alkylbenzenes: (a) Current Route, i.e., “Assembly” Synthesis;^326^ and (b) One-Pot Tandem Process from PE, i.e., “Deconstruction” Synthesis^49^

Alkylbenzenes with alkyl chains considerably longer than methyl (surfactant-range linear alkylbenzenes, or LABs, with carbon numbers C_16_–C_22_) are the direct precursors of linear alkylbenzene sulfonates, which are produced commercially as biodegradable surfactants.^323^ Global LAB production was 1.4 million tons in 2018, with demand projected to increase at an compound annual growth rate of 4.7 % through 2027.^324^ The principal synthesis route involves the alkylation of petroleum-derived benzene with a petroleum-derived alkene (derived from either alkane dehydrogenation or ethylene oligomerization), in the presence of an acid catalyst (either homogeneous, as in AlCl_3_-HCl or HF, or solid, as in silica-alumina or a zeolite), Scheme 30a.^325−328^ These syntheses are “assembly”-type.

A “deconstruction” approach was devised to synthesize long-chain dialkylaromatics by combining partial depolymerization of PE with H_2_ redistribution in a tandem hydrogenolysis/aromatization process.^49^ Using Pt/γ-Al_2_O_3_ (0.200 g, 1.5 wt% Pt) as the catalyst, the reaction of PE (0.120 g, *M*_w_ = 3.5 × 10^3^ g/mol, *Đ* = 1.9) was conducted under Ar (i.e., in the absence of external H_2_) and in the absence of added solvent for 24 h at 280 °C. The liquid products contained dialkylbenzenes, dialkyltetralins, and dialkylnapthalenes in ca. 40 wt% yield relative to the initial mass of PE, and with an overall carbon number distribution centered at C_∼__30_ (*Đ* = 1.1), Scheme 30b. Other hydrocarbon products included long-chain *n*-alkanes (also C_∼__30_, ca. 40 wt%), light hydrocarbon gases and volatile hydrocarbons (C_<10_, 10 wt%), and insoluble hydrocarbons (10 wt%). An LDPE plastic bag and an HDPE water-bottle cap were also converted to alkylaromatics using the same process, although with slightly lower yields.

The conversion of PE catalyzed by Pt/γ-Al_2_O_3_ was slow, and the alkyl side-chains were longer than desired for drop-in use in anionic surfactants (which typically have total carbon numbers C_16_–C_22_). In a subsequent study, the catalytic activity and selectivity for surfactant-range alkylaromatics was improved with a catalyst modification inspired by the bifunctional (metal/acid) catalysts used in naphtha catalytic reforming (see section 3.6.1.1). At 280 °C under Ar, the more acidic catalysts Pt/Cl-Al_2_O_3_ (1.5 wt% Pt, 1.4 wt% Cl) and Pt/F-Al_2_O_3_ (1.6 wt% Pt, 0.8 wt% F) proved much more active than less acidic Pt/γ-Al_2_O_3_ in the tandem hydrogenolysis/aromatization of PE (*M*_w_ = 3.5 × 10^3^ g/mol, *Đ* = 1.9).^50^ A 5-fold enhancement in the rate of C–C bond scission and a doubling of the molar yield of alkylaromatics were achieved for the most Brønsted acidic catalyst (Pt/F-Al_2_O_3_) relative to the least Brønsted acidic catalyst (Pt/γ-Al_2_O_3_). The highest acidity catalyst also generated alkylaromatic products with a lower average carbon number, ca. C_20_, making them more similar to conventional LABs. A physical mixture of Pt/γ-Al_2_O_3_ and F-Al_2_O_3_ showed activity and selectivity similar to Pt/F-Al_2_O_3_ with the same active site content, indicating that nanoscale proximity between Pt sites and BAS is not necessary to convert PE to alkylaromatics.

Metals can also be loaded onto acidic zeolites. The depolymerization of HDPE (5.00 g, *M*_w_ = 2.8 × 10^5^ g/mol, *Đ* = 3.8) catalyzed by Ru/HZSM-5 (0.50 g, Si/Al = 300) was conducted at 280 °C in an inert atmosphere.^329^ After 24 h, PE conversion reached 70 wt%. The products included alkanes (34 mol%) and alkenes (6 mol%) ranging from C_3_ to C_6_, but the major products were cyclic hydrocarbons from C_7_ to C_15_ (60 mol%). The latter consisted of cyclopentanes (15 mol%), cyclopentenes (1 mol%), and alkylbenzenes (44 mol%). Distillation between 69 °C (the boiling point of *n*-C_6_H_14_) and 91 °C (the boiling point of 1,3-dimethylcyclopentene) removed most of the acyclic alkanes, producing a liquid containing 90 mol% cyclic hydrocarbons.

**Figure 49:**
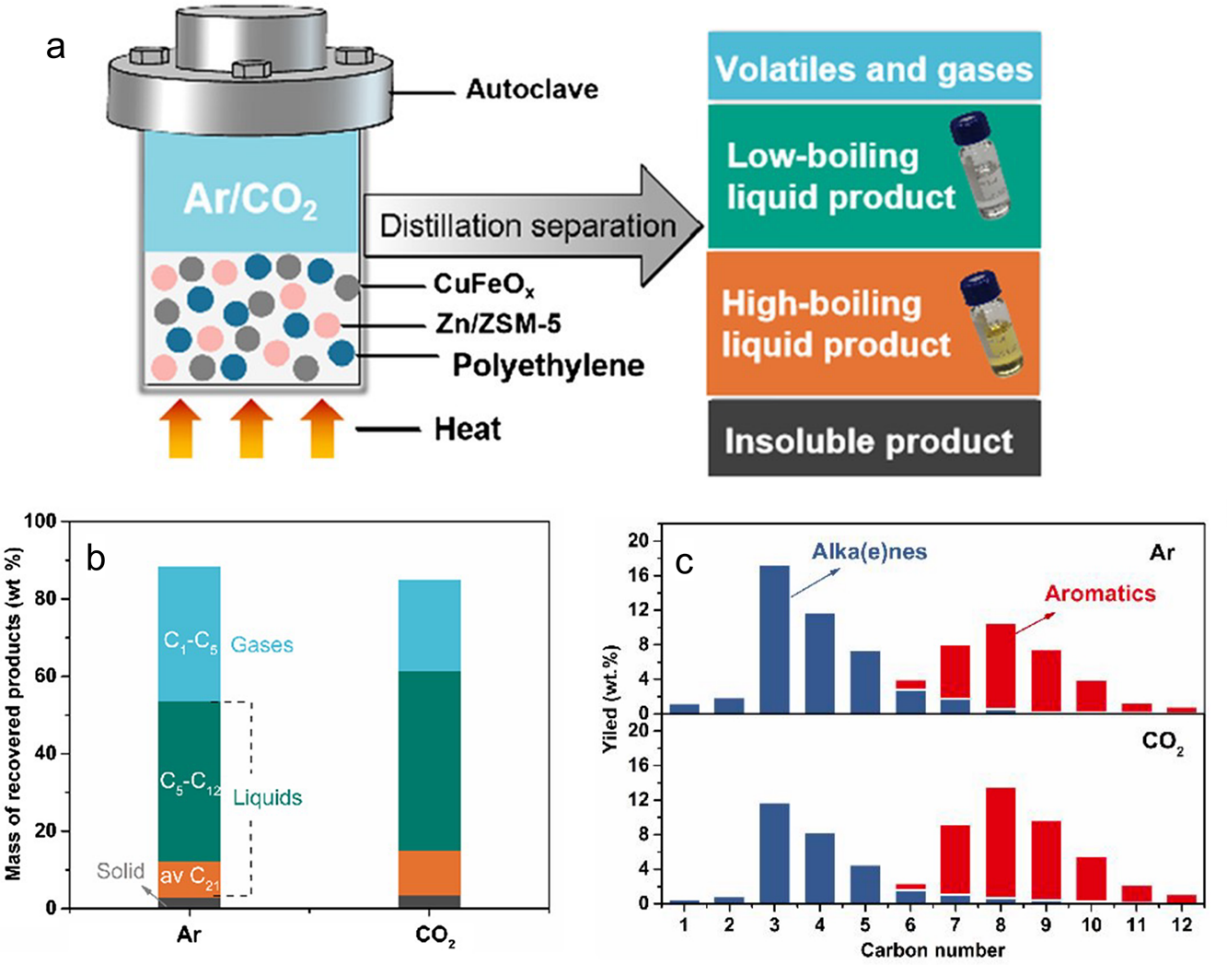
Strategy for converting PE to aromatics in the presence of CO_2_: (a) Schematic of the reactor and product fractions; (b) recovered product distributions; and (c) distribution of hydrocarbons in the volatile and low-boiling liquid products. Reaction conditions: physical mixture of Zn/ZSM-5 5 (0.2 g, Si/Al = 42.5, 3 wt% Zn, Zn particle size and size distribution unspecified), Cu-Fe_3_O_4_ (0.2 g, 10 wt% Cu, Cu particle size and size distribution unspecified), and PE (2.0 g, *M*_w_ = 3.5 × 10^3^ g/mol, *Đ* unspecified) at 360 °C under 20 bar Ar or CO_2_, 1.0 h. Reproduced with permission from ref (330). Copyright 2023, Chinese Chemical Society.

Instead of conducting the reaction in an inert atmosphere, PE depolymerization was explored under 20 bar CO_2_ (a weak oxidant), Figure 49.^330^ The catalyst was a combination of Zn/ZSM-5 and Cu/Fe_3_O_4_. After 1 h at 360 °C, PE was converted to a mixture of gases and volatiles (C_1_–C_5_, 24 wt%, ca. 90 wt% alkanes), low-boiling liquids (C_6_–C_12_, 46 wt%, ca. 85 wt% aromatics), high-boiling liquids (soluble in CH_2_Cl_2_, 12 wt%, nearly 100 wt% aromatics), and insoluble solids (3 wt%). Compared to the depolymerization products obtained under Ar, the molecular weight distribution under CO_2_ was shifted slightly to heavier products. In addition, CO was detected as a byproduct. The total aromatics yield under CO_2_ was slightly higher (56 wt%) than under Ar (43 wt%). These results suggest that CO_2_ consumes H_2_ formed as a byproduct of dehydrocyclization and aromatization via the reverse water-gas shift reaction. Lowering the amount of internally generated H_2_ reduces the rate of C–C bond scission, while simultaneously promoting the formation of aromatics.

The promoting effect of CO_2_ (10 bar) on aromatic formation from LDPE (1.0 g, *M*_w_ = 290,000 g/mol, *Đ* unspecified) was also studied at 300 °C using a bifunctional Pt/MnO_x_-ZSM-5 catalyst (0.4 g).^331^ Control experiments showed that HZSM-5 alone catalyzed the cracking and aromatization of LDPE, producing H_2_ as a byproduct. At the same time, Pt/MnO_x_ catalyzed CO_2_ reduction by H_2_, driving the product distribution towards increased aromatic content. Isotopic labeling experiments involving ^13^CO_2_ revealed that 0.2 g CO_2_ was consumed per gram PE. The authors concluded that most of this CO_2_ (90 %) was incorporated into the aromatic products (benzene and toluene) via an unspecified mechanism, with the remaining 10 % forming CO. Overall, the products included 0.63 g aromatics (C_6_–C_11_, with BTX accounting for 60 wt%), with the remainder being alkanes.

The process was also explored using other POs, including a higher molecular weight LDPE (*M*_w_ = 4.8 × 10^5^ g/mol, *Đ* unspecified), HDPE (*M*_w_ = 2.5 × 10^5^ g/mol, *Đ* unspecified), PP (*M*_w_ = 4.7 × 10^4^ g/mol, *Đ* and tacticity not specified), and post-consumer plastics like kitchen PE film (*M*_w_ = 3.5 × 10^5^ g/mol, *Đ* unspecified) and PP bottles (*M*_w_ = 6.8 × 10^6^ g/mol, *Đ* unspecified). Aromatic yields ranged from 56 to 61 wt%. Catalyst recycling was investigated by calcining used Pt/MnO_x_-ZSM-5 in air at 600 °C, followed by reduction in H_2_ (10 vol% in Ar) at 320 °C. No loss of activity was detected after four such regeneration cycles. A combination of ZSM-5 and CuZnZrO_x_ was also shown to catalyze PE depolymerization to aromatics under 5 bar CO_2_.^486^

###### 3.6.3.2.1 Characterization of Alkylaromatics

The complexity of the product slate (molecular weight, hydrocarbon type, and number of isomers) from PO aromatization makes characterization of the alkylaromatic component challenging. Simple 1D gas chromatography often fails to resolve individual species, especially for products with intermediate carbon numbers (C_10-30_). Characterization of long-chain alkylaromatics often requires a combination of techniques, including NMR spectroscopy, gas chromatography, mass spectrometry, and size exclusion chromatography (also called gel permeation chromatography, GPC).

**Figure 50:**
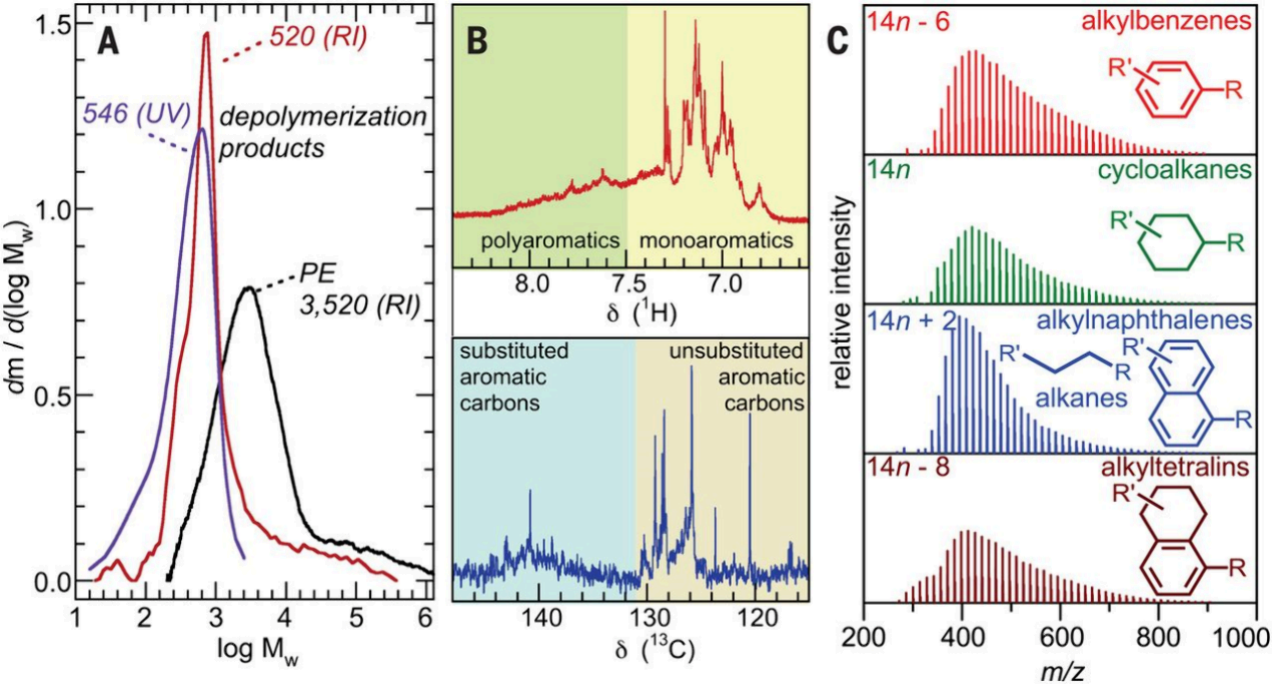
Analysis of the liquid hydrocarbons recovered from the solvent-free depolymerization of PE (*M*_w_ = 3.5 × 10^3^ g/mol, *Đ* = 1.9) catalyzed by Pt/γ-Al_2_O_3_ (0.200 g, 1.5 wt% Pt), after 24 h at 280 °C in an unstirred mini-autoclave reactor: (a) GPC analysis, conducted using both refractive index (RI) and UV detectors; (b) ^1^H and ^13^C NMR spectra, recorded in 1,1,2,2-tetrachloroethane-d_2_; and (c) FD-MS analysis. Reproduced with permission from ref (49). Copyright 2020, AAAS.

The carbon number distribution of the alkylaromatic components specifically can be elucidated by GPC by using UV detection to distinguish aromatic from non-aromatic hydrocarbons, as well as by FD-MS (which uses a soft ionization technique for generating molecular ions from hydrocarbons). FD-MS signals can be isolated for different mass series, e.g., 14*n*-6 (representing alkylbenzenes) and 14*n*-8 (representing alkyltetralins), Figure 50c.^49^ In ^1^H NMR, aromatic protons resonate mostly in the range 6.5–8.5 ppm (*H*_arom_), while aliphatic protons on carbons directly attached to an aromatic ring (H_α_) resonate mostly in the range 2.0–3.5 ppm. Using the relative signal intensities, as well as the ^13^C NMR-derived ratio of substituted aromatic carbons (131–146 ppm) to unsubstituted (i.e., proton-bearing) aromatic carbons (118–130 ppm), the average number of alkyl substituents on each aromatic ring can be deduced. A detailed discussion of product analysis for such mixtures has been published.^44^

###### 3.6.3.2.2 Chain Length of Alkyl Substituents

**Figure 51:**
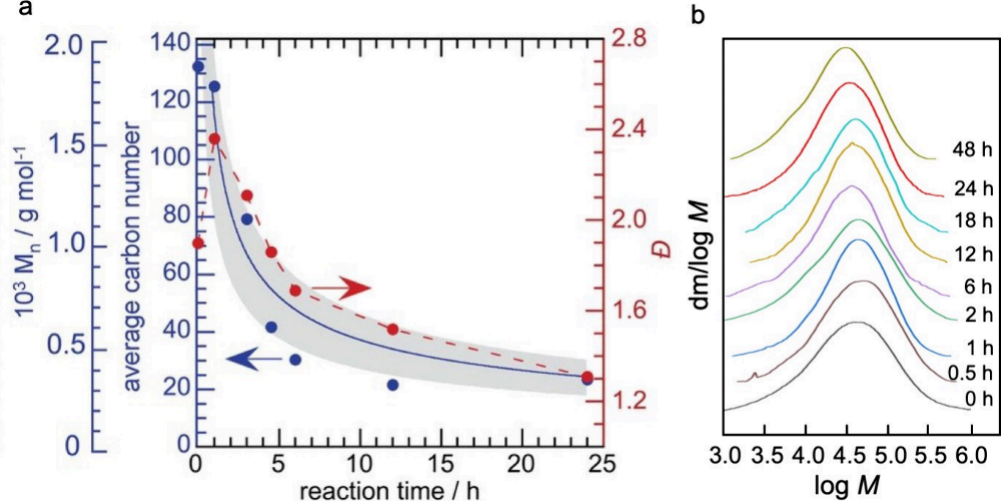
(a) Time course of solvent-free depolymerization of PE (*M*_w_ = 3.52 × 10^3^ g/mol, *Đ* = 1.9) catalyzed by Pt/γ-Al_2_O_3_ (0.200 g, 1.5 wt% Pt) in an unstirred mini-autoclave reactor at 280 °C. Average molecular weight (*M*_n_, blue) and dispersity (*Đ*, red) are shown for all non-gas hydrocarbons. The red dashed line is present only to guide the eye. The shaded region indicates 95 % confidence bands for the model fit. Reproduced with permission from ref (49). Copyright 2020, AAAS. (b) Time-dependent GPC analysis of solid residue recovered from depolymerization of HDPE (5 g, *M*_w_ = 2.8 × 10^5^ g/mol, *Đ* = 3.8) catalyzed by Ru/HZSM-5 (0.5 g, Si/Al = 300) at 280 °C. Reproduced with permission from ref (329). Copyright 2023, Springer Nature.

The time course of PE depolymerization at 280 °C catalyzed by Pt/γ-Al_2_O_3_ under Ar showed a gradual shift in the products away from CHCl_3_-insoluble hydrocarbons to CHCl_3_-soluble liquids and gases over several hours.^49^ This shift indicates that C–C bond scission was random.^54^ The value of *M*_n_ in the liquid fraction decreased rapidly, with the average carbon number declining from C_130_ to C_40_ in the first 6 h, eventually stabilizing at C_25_ after ca. 12 h, Figure 51a. A slowing ofthe rate of change in the average carbon number is expected, even if the rate of scission remains constant (see section 1.2.1.1). However, aromatics (especially polyaromatics) may adsorb preferentially onto the active sites, slowing the reaction as it proceeds.^332^

In the depolymerization of HDPE (5.00 g, *M*_w_ = 2.8 × 10^5^ g/mol, *Đ* = 3.8) to C_3_–C_15_ hydrocarbons (including aromatics) catalyzed by Ru/HZSM-5 (0.50 g, Si/Al =300) at 280 °C, the molecular weight distribution of the solid residue was similar to that of the original polymer, according to GPC (Figure 51b). The authors inferred that once a polymer chain adsorbs onto the catalyst, it remains adsorbed until the entire polymer chain is converted to low molecular weight hydrocarbons. Hence, polymer chains with intermediate carbon numbers did not appear in the liquid phase.

The apparent difference in mechanisms for Pt/γ-Al_2_O_3_ and Ru/HZSM-5 may arise due to the stronger dehydrogenating ability of Pt relative to Ru, combined with the weaker acidity of γ-Al_2_O_3_ relative to the zeolite. The high metal-to-acid ratio (MAB) of Pt/γ-Al_2_O_3_ created the conditions for ideal hydrocracking (see section 3.1.3), in which adsorption and desorption of alkene intermediates occurred readily. These conditions favor random scission. In contrast, Ru/HZSM-5 operated in the low metal-to-acid regime where low alkene concentration resulted in persistent alkene-acid interactions, promoting consecutive C–C bond scission on the same hydrocarbon chain. In addition, the zeolite micropores may have played a role (see section 3.6.3.2.4).

###### 3.6.3.2.3 Thermodynamics of Alkylaromatic Formation

**Scheme 31:**
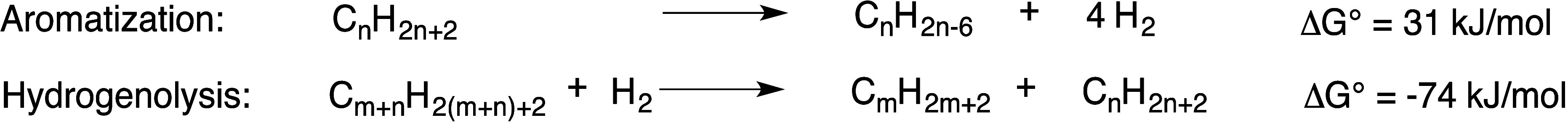
Gibbs’ Energies for Aromatization and C–C Bond Hydrogenolysis at 280 °C, Estimated Using Benson Group Contributions^49^

PE conversion to long-chain alkylaromatics involves tandem hydrogenolysis and aromatization. The latter is highly endothermic, whereas the former is exothermic (Scheme 31). Their coupling can make the overall process thermodynamically favorable at 280 °C, via the use of some of the H_2_ generated by aromatization in the hydrogenolysis of C–C bonds.^49^ Since PE acts as both a source and a sink for H_2_, the aromatic yield was higher starting from the polymer compared to the tandem reaction of a low-molecular-weight alkane, with its limited ability to undergo hydrogenolysis.

**Figure 52:**
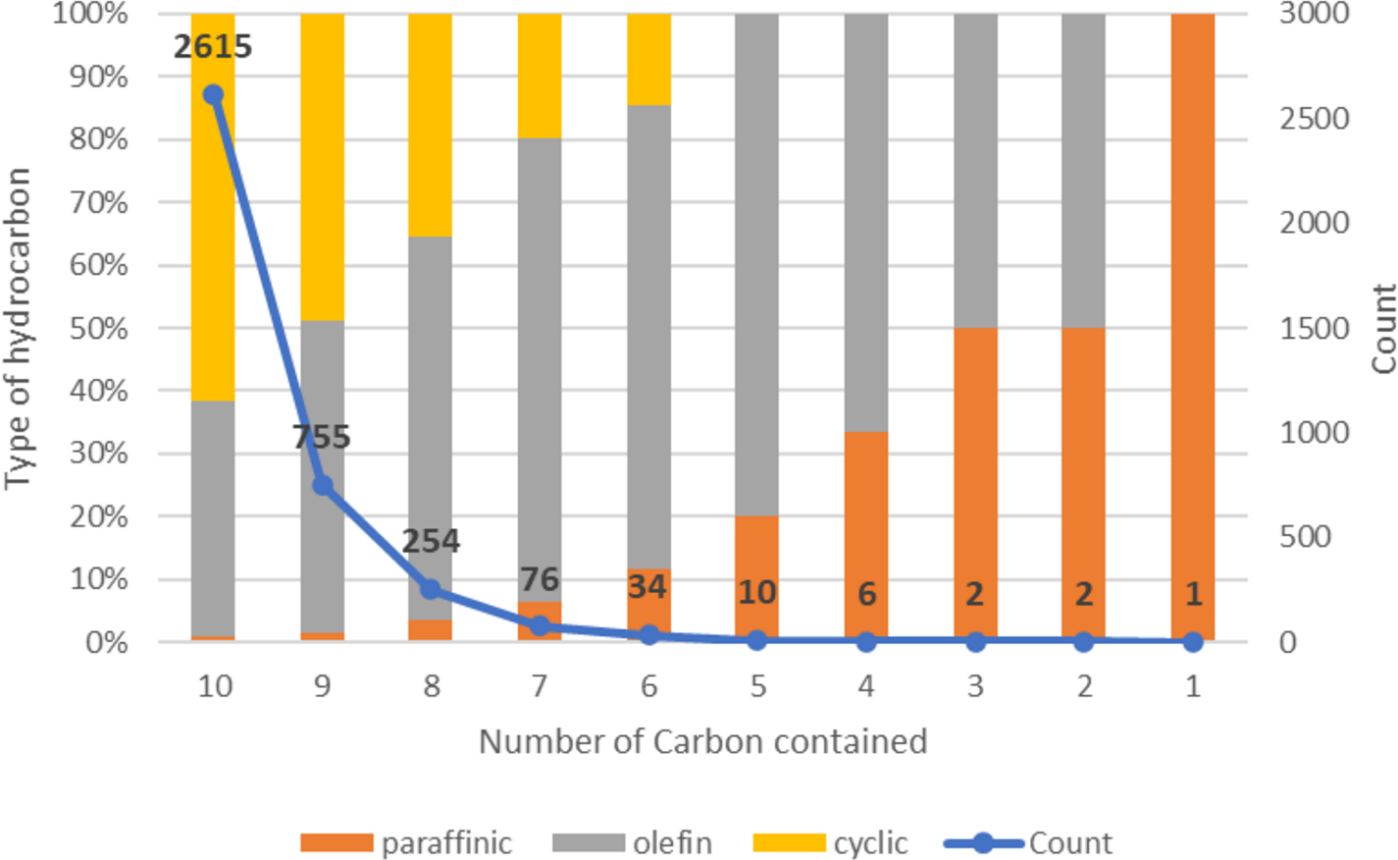
Statistical analysis of the reaction network for *n*-decane aromatization coupled with hydrogenolysis. The abscissa represents the number of carbon atoms in each product molecule, while the blue line is the number of possible molecules with carbon number *C*_*n*_ for *n* = 1 to 10 (right-hand ordinate). Species in the reaction network are classified as alkanes (orange), acyclic alkenes (including alkadienes, gray), and cyclic hydrocarbons (including cycloalkanes, cycloalkenes, cycloalkadienes, and aromatics, yellow). Reproduced with permission from ref (333). Copyright 2023, American Chemical Society.

Predicting thermodynamic equilibria in such a complex reaction network is challenging. The number of possible structures, particularly substituted cyclic hydrocarbons, rises rapidly as the carbon number increases (Figure 52). As a result, the equilibrium yield of aromatics would be significantly underestimated if the vast array of possible aromatic structures were not considered. An informatics-based reaction network generation for the conversion of a simple model compounds, *n*-C_10_H_22_, to aromatics was integrated with machine learning-based thermodynamic calculations to identify 24,000 plausible reactions giving 3,759 species.^333^ This study showcases the potential value of machine learning in dealing with complex systems.

Adding CO_2_ (20 bar) during PE depolymerization catalyzed by a physical mixture of Zn/ZSM-5 (Si/Al = 42.5, 3 wt% Zn) and Cu–Fe_3_O_4_ (10 wt% Cu) at 360 °C decreased the yield of alkanes and improved the aromatics yield. This outcome was suggested to be a result of H_2_ consumption via the reverse water-gas shift reaction.^330,331^

###### 3.6.3.2.4 Effect of Catalyst Design on the Aromatization Mechanism

To confirm the bifunctional reaction mechanism and improve the rate of PE conversion to aromatics, a series of catalysts were created by loading Pt onto supports with different acidities, including non-acidic SiO_2_, mildly acidic γ-Al_2_O_3_, more acidic SiO_2_–Al_2_O_3_, and highly acidic HZSM-5.^50^ For each catalyst (0.200 g), the reaction of triacontane (*n*-C_30_H_62_, 0.120 g) was explored at 280 °C under Ar. Use of a small molecule alkane as a model for PE facilitated quantitative product analysis. The rate of C–C bond scission increased with catalyst acidity. However, the more acidic catalysts led to the formation of low-value gases as well as insoluble hydrocarbons. Thus, catalyst acidities intermediate between γ-Al_2_O_3_ and SiO_2_–Al_2_O_3_ were targeted for further study.

Mild surface halogenation of γ-Al_2_O_3_ resulted in the replacement of surface OH groups by halogens (Cl or F), with little effect on surface area or porosity. The fraction of remaining hydroxyls that were strong BAS increased, due to the inductive effect of the halide ligands. The number of strong LAS also increased. Mixing Pt/γ-Al_2_O_3_ with Cl-Al_2_O_3_ or F-Al_2_O_3_ caused the rate of C–C bond scission in *n*-C_30_H_62_ to increase significantly.

**Figure 53:**
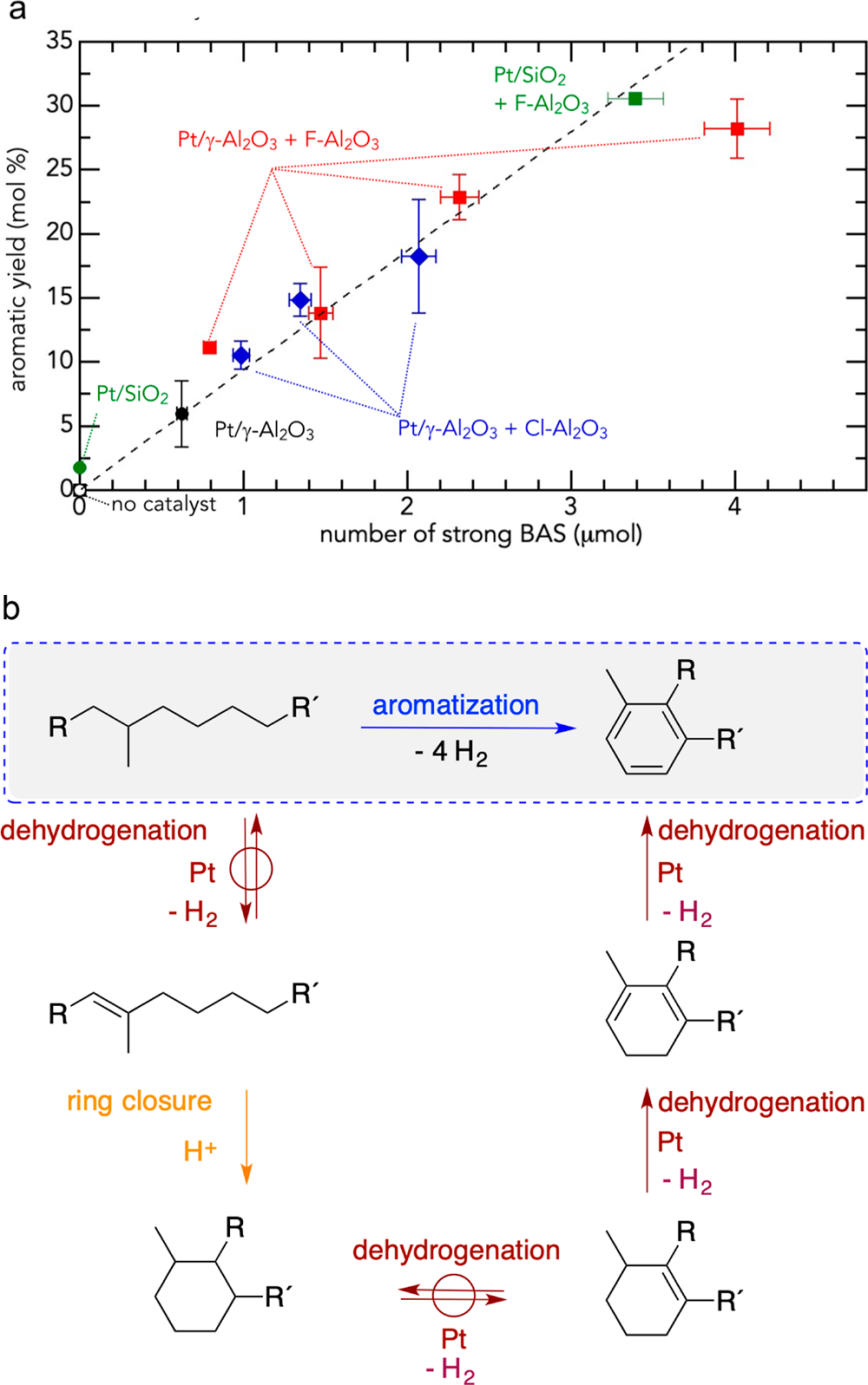
(a) The number of strong BAS correlates with the yield of aromatics from triacontane. Reaction conditions: *n*-C_30_H_62_ (0.120 g), Pt catalyst (0.100 g, 1.5 wt% Pt supported on either SiO_2_ or γ-Al_2_O_3_) alone or physically mixed with various amounts of either F-Al_2_O_3_ (1.3 wt% F) or Cl-Al_2_O_3_ (1.8 wt% Cl), 12 h at 280 °C. Abscissa error bars represent the standard deviation in the measurement of the sum of the number of strong BAS contributed by each catalyst support. Ordinate error bars represent the standard deviation of duplicate experiments. (b) Proposed bifunctional mechanism for aromatization of PE fragments. Reproduced with permission from ref (50). Copyright 2023, Elsevier.

To probe the roles of BAS and LAS, the Pt catalyst was mixed with varying amounts of each acid catalyst. In all cases, the metal-to-acid balance (*n*_Pt_/*n*_BAS_) (MAB) was high enough to ensure that Pt-catalyzed (de)hydrogenations remained quasi-equilibrated. The rate of C–C bond cleavage, the yield of *iso*-alkanes, and the yield of aromatics, all correlated linearly with the number of strong BAS.^50^ Correlations with the total number of BAS (including weak BAS), or with the number of LAS (strong or total) were much weaker. As an example, the correlation of the aromatics yield with the number of strong BAS is shown in Figure 53a. Note that addition of F-Al_2_O_3_ to inactive, non-acidic Pt/SiO_2_ resulted in a highly effective bifunctional tandem catalyst. The proposed bifunctional mechanism for aromatization is illustrated in Figure 53b. Pt sites are primarily responsible for alkane hydrogenation/dehydrogenation, while strong BAS protonate alkenes to form carbenium ions, which cyclize in the rate-determining step. Cycloalkanes are then dehydrogenated on Pt sites to form aromatics.

**Figure 54:**
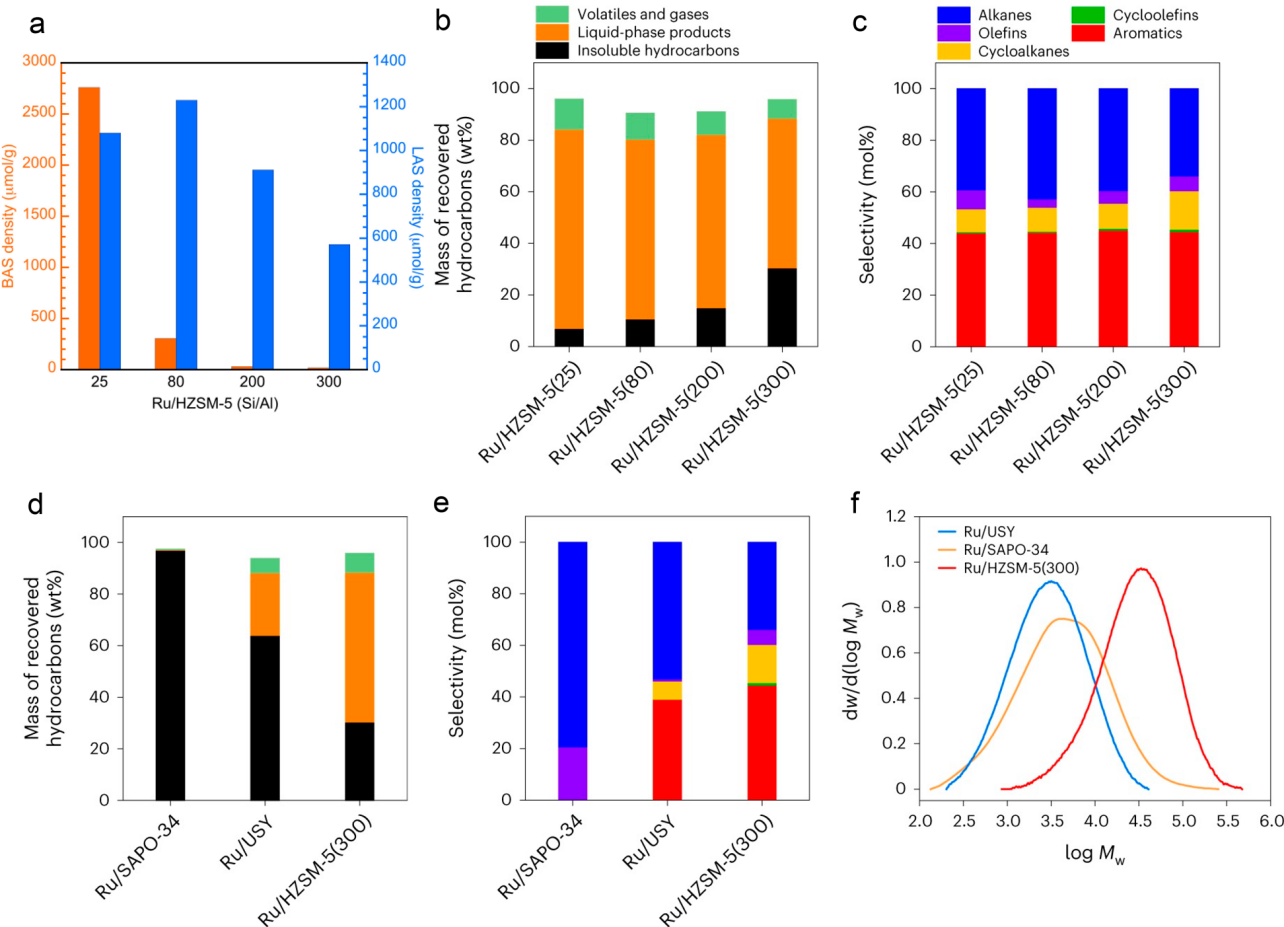
(a) Acid site characterization of Ru/HZSM-5 with various Si/Al ratios. The effect of various Ru-based catalysts on HDPE upcycling at 280 °C for 24 h on (b and d) the yields of volatiles/gases, liquid-phase products and insoluble hydrocarbons; (c and e) the selectivity for volatiles/gases and liquid-phase products; and (f) GPC analysis of the solid residues. Adapted with permission from ref (329). Copyright 2023, Springer Nature.

To explore the role of support acidity in HDPE depolymerization, Ru/HZSM-5 catalysts with different Si/Al ratios (25, 80, 200, 300, 0.50 g) were prepared.^329^ The total acidity of the catalysts, measured by NH_3_-TPD, ranged from 3800 to 600 mmol g^–1^. Based on a combination of NH_3_-TPD and IR of adsorbed pyridine adsorption, the BAS density decreased as Si/Al increased, while the LAS density initially increased slightly then decreased (Figure 54a). At 280 °C, the conversion of HDPE (5.00 g, *M*_w_ = 2.8 × 10^5^ g/mol, *Đ* = 3.8), as well as the gas and liquid hydrocarbon yields, decreased as Si/Al increased (Figure 54b), correlating with increases in both BAS and total acidity. A control experiment using non-acidic Ru/SiO_2_ showed even lower activity (21 mg_HDPE_ g_cat_^–1^ h^–1^) than Ru/HZSM-5 with Si/Al = 300 (290 mg_HDPE_ g_cat_^–1^ h^–1^). The selectivity for cyclic hydrocarbons (cycloalkanes, cycloalkenes, and aromatics) increased slightly as Si/Al increased, Figure 54c.

**Scheme 32:**
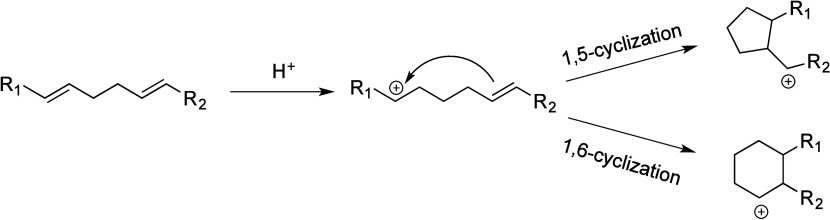
Proposed Mechanism for the 1,5- and 1,6-Cyclization of Alkadienes during HDPE Upcycling Catalyzed by Ru/HZSM-5^329^

In PE depolymerization catalyzed by Ru/SiO_2_, only methane, linear alkanes, and alkenes were observed, indicating that while the non-acidic catalyst promotes hydrogenolysis and dehydrogenation, it is incapable of inducing alkene cyclization. The authors proposed that cyclization occurs by protonation of an alkadiene intermediate to give a carbenium ion that reacts with a nearby C=C bond to form a five- or six-membered ring, Scheme 32. For catalysts with abundant BAS, carbenium ions form readily, but the number of nearby C=C bonds is likely to be insufficient to allow for efficient cyclization. In such cases, the carbenium ions may undergo β-scission instead. Alternatively, cyclization has been proposed to occur directly from an alkene intermediate, as proposed in the mechanism of naphtha catalytic reforming (Scheme 27).^334^ DFT studies have shown that 1-hexene cyclization via a 2-hexylcarbenium ion has a lower activation energy than 1,5-hexadiene cyclization via attack of the double bond on the carbenium ion.^335,336^

The effect of pore size on HDPE depolymerization to aromatics was studied, comparing Ru/USY, Ru/SAPO-34, and Ru/HZSM-5 (Si/Al = 300). HDPE conversion was higher for Ru/HZSM-5 than for Ru/USY, and Ru/SAPO-34 showed little reactivity (Figure 54d). The larger pore diameter of USY, compared to HZSM-5, was suggested to weaken interactions between polymer chains and pore walls, causing long-chain alkanes to desorb and exit the pores instead of continuing to crack. This hypothesis was further supported by the observed shift in molecular weight distribution (Figure 54f). The inability of Ru/SAPO-34 to make aromatics (Figure 54e) was attributed to the small pore size of SAPO-34, which does not allow for cyclization. However, the catalysts have different acidities and activities, and the selectivity comparisons were made at very different HDPE conversions, weakening conclusions about pore-size effects.

The role of the metal component in aromatic formation has also been studied. In PE depolymerization at 280 °C for 24 h, Pt/γ-Al_2_O_3_ gave significantly enhanced PE conversion (from 40 to 95 wt%) and increased aromatic yield (from negligible to 40 wt%), compared with γ-Al_2_O_3_ alone.^49^ The role of Ru nanoparticles in HDPE conversion was similarly assessed by comparing Ru/HZSM-5 (Si/Al = 300) with the pristine support.^329^ At 280 °C, the conversion after 24 h had increased from 40 wt% without Ru to 70 wt% with Ru, while the liquid product yield increased from 23 to 58 wt%. Almost identical molecular weight distributions were obtained for both Ru/HZSM-5 and HZSM-5, indicating that the acid component controlled the location of C–C bond scission. However, Ru nanoparticles enhanced the total yield of cyclic products from 42 to 60 mol%, mostly due to the increased yield of aromatics (from 24 to 44 mol%). The role of the metal in promoting dehydrogenation was further supported by the higher conversion of cyclohexane to aromatics catalyzed by Ru/HZSM-5, compared with HZSM-5.

The presence of a second metal can be beneficial in naphtha catalytic reforming,^294^ and the concept has been extended to PO depolymerization to aromatics. Fe_2_O_3_-modified Pt/Al_2_O_3_ showed improved selectivity in the production of light oil (C_7_–C_19_) from PE at 280 °C and also promoted aromatic formation in the oil fraction.^43^ The Fe_2_O_3_ component was proposed to catalyze Diels–Alder reactions between alkene and alkadienes, although no experimental evidence was provided in support of this hypothesis.

### 3.7 Conclusions and Perspectives

**Scheme 33:**
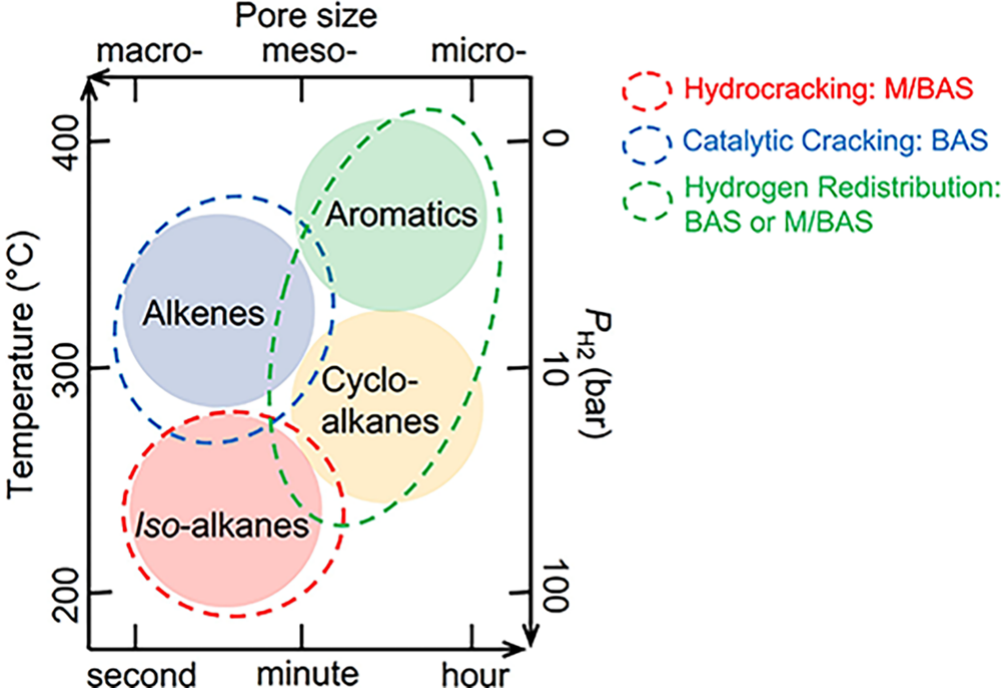
Semiquantitative Depiction of Reaction Conditions and Catalyst Architectures That Determine Selectivity in Polyolefin Cracking to Different Hydrocarbon Types (Filled Circles) and Reaction Regimes (Dashed Circles), for Monofunctional Acid Catalysts and Bifunctional Metal–Acid Catalysts

Catalytic cracking, hydrocracking, and hydrogen redistribution convert POs into smaller alkenes, cycloalkanes, *iso*-alkanes, and/or aromatics, which could serve as chemical building blocks, fuels, lubricants, and surfactants. Selectivities for different types of hydrocarbon products depend on reaction conditions (e.g., *P*_H2_, temperature, residence time), as well as catalyst properties (Brønsted acidity, metal function, pore structure), as shown in Scheme 33.

At reaction temperatures typical of selective PO depolymerization, BAS are the major active sites that generate carbenium ions. These key intermediates undergo isomerization, cyclization, and C–C bond scission. Metal sites activate C–H bonds, increasing the rate of alkane dehydrogenation to alkenes, which are readily protonated by BAS to form carbenium ions. The conversion of cycloalkanes to aromatics is also accelerated by metal-catalyzed dehydrogenation. In addition, metals activate H_2_ readily, and when *P*_H2_ is high, lead to the formation of saturated *iso*-alkanes.

Low molecular weight alkanes appear to crack more readily than PO chains, possibly due to differences in mass transfer rates. In PO hydrocracking, reactivity typically increases with the branching frequency (i.e., HDPE < LDPE < PP); *iso*-alkane selectivity increases similarly. The complexities in modeling PO hydrocracking stem from the diverse hydrocarbon mixtures that are generated. Lumped models offer a practical way to analyze basic kinetic and mass transport data. Studies of the influence of *P*_H2_ on PO hydrocracking reveal that optimal rates require intermediate *P*_H2_ values, suggesting the need for further investigation into the effects of *P*_H2_ on the reaction mechanism.

Making hydrocarbon products with a narrow molecular weight distribution is an important challenge in PO upcycling. For unfunctionalized hydrocarbons, hydrocracking selectivity is controlled by MAB (*n*_metal_/*n*_BAS_). A high MAB favors random PO scission and generates higher-value alkanes (e.g., in the lubricant range), whereas a low MAB yields mostly lower-value light gases via chain-end scission. Catalysts with higher metal contents ensure that reactions on the acid sites are rate-determining, although the catalyst cost increases. Another way to control the product carbon number distribution is to use well-defined pore sizes to induce size selectivity. However, high molecular weight POs do not diffuse efficiently into catalyst micropores. Consequently, cracking occurs first on external catalyst surfaces, and the resulting hydrocarbon fragments undergo further reactions in the micropores. Typically, the major products are centered in the light gas (C_1_–C_4_) or gasoline (C_6_–C_12_) ranges. Catalysts that generate more valuable products selectively and with intermediate carbon numbers would be of interest.

PO conversion to aromatics has attracted significant attention due to the higher value of the products. Traditionally, the focus has been on PO conversion to light aromatics (BTX). However, more valuable aromatics (for example, linear alkylbenzenes) can be synthesized directly by PO deconstruction, as an alternative to their construction from BTX. The one-pot deconstruction approach may be beneficial in terms of efficiency, although the complexity of the reaction network and the large number of possible products make characterization and separation, as well as prediction of the thermodynamics, very challenging. Overall, converting POs to aromatics offers significant potential for advancing the circular economy but initial findings will require more research and development to reach scale.

Acidity is essential in catalytic PO transformations to aromatics. While acid sites promote aromatization, the extensive skeletal isomerization induced by these sites leads to methyl branches directly on the aromatic rings and/or in the alkyl substituents, potentially altering product properties. For example, branching could affect the ease of sulfonation, surfactant performance, and biodegradability.^337,338^ In addition, acid sites catalyze C–C bond cleavage, influencing the average length of alkyl substituents. Separate control of cyclization and C–C bond scission processes would help in directing product distributions. Zeolite pore sizes affect the yield and type of aromatics, by controlling diffusion of intermediate alkenes as well as confinement effects. Some monofunctional metal catalysts known to convert *n*-C_6_H_14_ selectively to benzene are less effective in PO conversion, especially at lower temperatures (ca. 300 °C).^50,329^ These catalysts could be interesting for further study, perhaps at higher temperatures.

Hydrocracking is capable of converting individual POs under similar reaction conditions. However, processing mixed plastics poses challenges in maintaining reasonably constant product compositions. In addition, plastic additives or contaminants can poison metal and/or acid active sites, requiring either pretreatment of plastic waste or the development of less harmful additives and more tolerant depolymerization strategies. Catalyst regenerability is important for assessing the potential cost-effectiveness of PO upcycling. Although some hydrocracking catalysts can be reused directly with minimal loss of activity, the activity decreases in most cases, presumably due to blockage of the active sites by hydrocarbon residues. Post-reaction catalyst regeneration is feasible with established industrial practices. Solid acid catalysts can be calcined to remove residues, while the (de)hydrogenation sites of bifunctional catalysts may require additional treatments (e.g., H_2_ reduction or sulfidation). The entire process must be carefully controlled, since the regeneration conditions may be detrimental to the catalyst structure. Coke buildup on the catalyst may be suppressed through continuous removal of aromatic products and/or active control of reactor *P*_H2_ levels.

## 4 Metathesis Upcycling of Polyolefins

### 4.1 Introduction to Alkane Cross-Metathesis

#### 4.1.1 General Principles

Alkane metathesis was first explored in the late 1960s and early 1970s by Chevron researchers, who named their new reaction “molecular redistribution”.^339^ The term “alkane metathesis” was coined in a seminal 1997 paper.^340^ Analogous to the well-known *alkene* metathesis reaction, *alkane* metathesis transforms alkanes of intermediate chain lengths into higher and lower homologs, or combines short and long chains to create intermediate-length alkanes. The chain disproportionation process is near-thermoneutral overall. Since PE is a macromolecular alkane, alkane cross-metathesis of PE with a light alkane converts polymer chains into intermediate length alkanes. Unlike PO hydrogenolysis, which can generate similar alkanes as products (see section 2), PO cross-metathesis does not require added H_2_ (although it does require a large quantity of light alkane, usually a low-cost hydrocarbon).

**Scheme 34:**
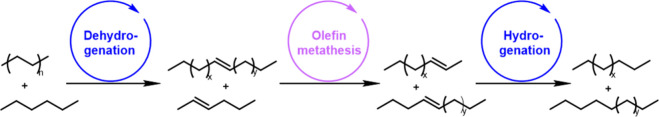
Overall Process of Tandem Alkane Metathesis to Transform PE into Intermediate Chain-Length Alkanes

Scheme 34 shows the overall process. First, alkenes are formed in a metal-catalyzed dehydrogenation step. Pairs of alkenes undergo cross-alkene metathesis to give new alkenes that are hydrogenated by the metal catalyst. When two discrete catalysts are used, the overall reaction is considered to be a tandem catalytic process.^341^ Since there is no need for high alkene concentrations or significant H_2_ production in the endothermic dehydrogenation step, the overall reaction can proceed at relatively mild temperatures.

#### 4.1.2 Catalyst Requirements

A tandem catalytic strategy for alkane metathesis requires two active sites: one for alkane dehydrogenation/alkene hydrogenation and a second for alkene metathesis. Thus far, alkane cross-metathesis has been investigated in PO upcycling using two homogeneous catalysts or two heterogeneous catalysts, as well as mixed systems. In one example of a fully homogeneous tandem catalyst system, an Ir pincer catalyst for dehydrogenation/hydrogenation was combined with a Schrock-type alkene metathesis catalyst.^342^ In a hybrid (homogeneous/heterogeneous) example, an Ir pincer catalyst was used in conjunction with a supported metathesis catalyst, either Re_2_O_7_/γ-Al_2_O_3_^343^ or CH_3_ReO_3_/Cl-Al_2_O_3_.^344^ Fully heterogeneous catalyst systems include PtSn/γ-Al_2_O_3_ combined with Re_2_O_7_/γ-Al_2_O_3_,^345^ and Pt/γ-Al_2_O_3_ combined with WO_3_/SiO_2_.^346^

For small molecule hydrocarbons alone (i.e., no PO component), alkane cross-metathesis has also been reported using “single-site” catalysts (typically, an organometallic complex of Zr, Ta, or W) activated by attachment to a solid support (e.g., SiO_2_, γ-Al_2_O_3_, or SiO_2_-Al_2_O_3_), followed by its reaction with H_2_ to generate supported metal hydrides. In such cases, one catalyst performs both functions.^347^ It is often presumed that a single metal site performs all the required reactions (although it is possible that a family of similar sites with different reactivities exists). There are as-yet no reports of PE upcycling by alkane cross-metathesis using these single-site catalysts, although there is one relevant report on the self-metathesis of PE (see section 4.3.6).

The alkane cross-metathesis strategy for PO upcycling has only been reported for PEs, although the branch frequency varies (i.e., HDPE, LDPE, and LLDPE). The strategy does not appear to be suitable for PP, due to the difficulty in achieving chain cleavage of tri-substituted alkenes via alkene metathesis.^348^

#### 4.1.3 Operating Conditions

In tandem alkane metathesis, the first step is alkane dehydrogenation. When conducted independently, this strongly equilibrium-limited reaction requires high temperatures (generally, near 500 °C) to achieve significant conversion.^349−351^ Such temperatures are not necessary in the tandem reaction, which is close to thermoneutral due to the coupling of endothermic alkane dehydrogenation with exothermic alkene hydrogenation. Furthermore, high temperatures are often undesirable operationally, since alkene metathesis catalysts generally have lower thermal stabilities than dehydrogenation catalysts. Consequently, one of challenges in the tandem catalysis approach is finding a single operating condition suitable for both catalysts, whose thermal sensitivities and optimal operating temperatures are quite different. Generally, the overall rate of alkane metathesis is limited by the rate of alkane dehydrogenation, while overall catalyst productivity is limited by the rate of deactivation of the alkene metathesis catalyst.

Although equilibrium constants for alkane dehydrogenation to alkenes become more favorable as the hydrocarbon chain length increases,^352−355^ the differences are not large enough to achieve extensive dehydrogenation of long-chain alkanes under typical reaction conditions. In alkane metathesis involving PE, working with the molten polymer is challenging because it is extremely viscous at temperatures slightly above typical melting points (120-140 °C). In addition, medium-to-high molecular weight PEs have low solubilities in hydrocarbon solvents. Consequently, the temperatures required to make the tandem reaction feasible are typically 150–300 °C. As the reaction temperature increases, side-reactions such as the dehydrocyclization of alkanes to cycloalkanes can occur. Cycloalkanes can be further dehydrogenated to aromatics, which lead to catalyst deactivation by coking.

### 4.2 Catalytic Alkane Metathesis of Model Hydrocarbons

We begin by describing kinetic and mechanistic studies of alkane cross-metathesis using model hydrocarbons, prior to applying the ideas to PE upcycling.

#### 4.2.1 Heterogeneous Metathesis Catalysts

In the earliest report of a molecular disproportionation reaction, the distinct roles of the dual active sites in enabling the redistribution of alkane chain lengths was studied by feeding *n*-butane at ca. 400 °C to a continuous, fixed-bed reactor containing one or both of two discrete catalysts: Pt-Li/γ-Al_2_O_3_ (a dehydrogenation/hydrogenation catalyst) and WO_3_/SiO_2_ (an alkene metathesis catalyst).^339^ Four catalyst configurations were explored: Pt catalyst alone, W catalyst alone, and two stacked catalyst beds consisting of Pt followed by W, or Pt on either side of W, Table 4. For catalyst beds with more than one catalyst, each component was separated by an inert layer.

**Table 4:**
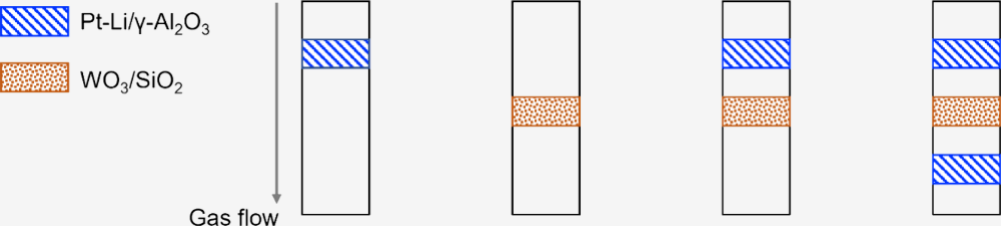
Product Distributions (Relative to 100 Mol *n*-Butane Feed) in Molecular Redistribution of *n*-Butane as a Function of Catalyst Configuration in a Continuous Flow, Fixed-Bed Reactor^a^

product component	Pt alone	WO_3_ alone	Pt/WO_3_	Pt/WO_3_/Pt
CH_4_	0.23	0	0.08	0.07
C_2_H_6_	0.16	0	0.05	0.17
C_2_H_4_	0	0	0.14	0
C_3_H_8_	0.19	0	0.09	1.17
C_3_H_6_	0.002	0	0.10	0.01
C_4_H_8_	4.10	0	1.95	4.10
C_5_H_12_	-	0	0	0.43
C_5_H_10_	-	0	0.38	0
C_6_^+^	-	0	0.29	0.31
total	4.68	0	3.08	6.26

a Reaction conditions: *n*-butane (5 mL/h, 4.4 bar), 399 °C, Pt-Li/γ-Al_2_O_3_ layer (0.5 wt% Pt, 0.5 wt% Li, 22 vol% relative to 5 mL total catalyst volume), WO_3_/SiO_2_ layer (2 wt% W, 78 vol% relative to 5 mL total catalyst volume). Adapted with permission from ref (339). Copyright 1973, Elsevier.

With the Pt catalyst alone, the main reaction was *n*-butane dehydrogenation to butenes, although a small amount of hydrogenolysis to lighter alkanes also occurred. Since alkane dehydrogenation is strongly endothermic, the yield of butenes was equilibrium-limited. The W catalyst alone generated no products. However, when the W catalyst was placed after the Pt catalyst, some of *n*-butene underwent disproportionation to lighter (C_3_) and heavier (C_5_) alkenes. When a second Pt catalyst bed was installed after the W catalyst, the products were the corresponding alkanes formed by hydrogenation of the alkene disproportionation products. These layered bed experiments established that both Pt and W components are necessary for *n*-butane self-metathesis, and that alkene intermediates are involved.

Installing a guard bed improved catalyst stability, therefore poisoning tests were carried out. Water was identified as a trace impurity in the feed, responsible for reversible deactivation of Pt catalyst. The activity was restored by heating at ca. 480 °C and purging thoroughly with dry H_2_. Another type of impurity, ammonia, also led to reversible deactivation. Details regarding which specific catalysts were affected by ammonia were not provided, however, activity was restored by purging with N_2_ at 400 °C for ca. 20 h. O_2_ and H_2_S poisoned the catalysts irreversibly. For example, injection of 138 μmol H_2_S resulted in complete and rapid loss of activity. Once again, deactivation by O_2_ and H_2_S was not explored in detail. Although alkenes and H_2_ are not catalyst poisons, an excess of either had a negative impact on catalytic productivity. The alkene deactivated the Pt catalyst but not the WO_3_ catalyst, by suppressing alkane dehydrogenation. With a high concentration of H_2_ (32 mol%) in the feed, the amount of *n*-butane hydrogenolysis increased, resulting in more methane production.

**Figure 55:**
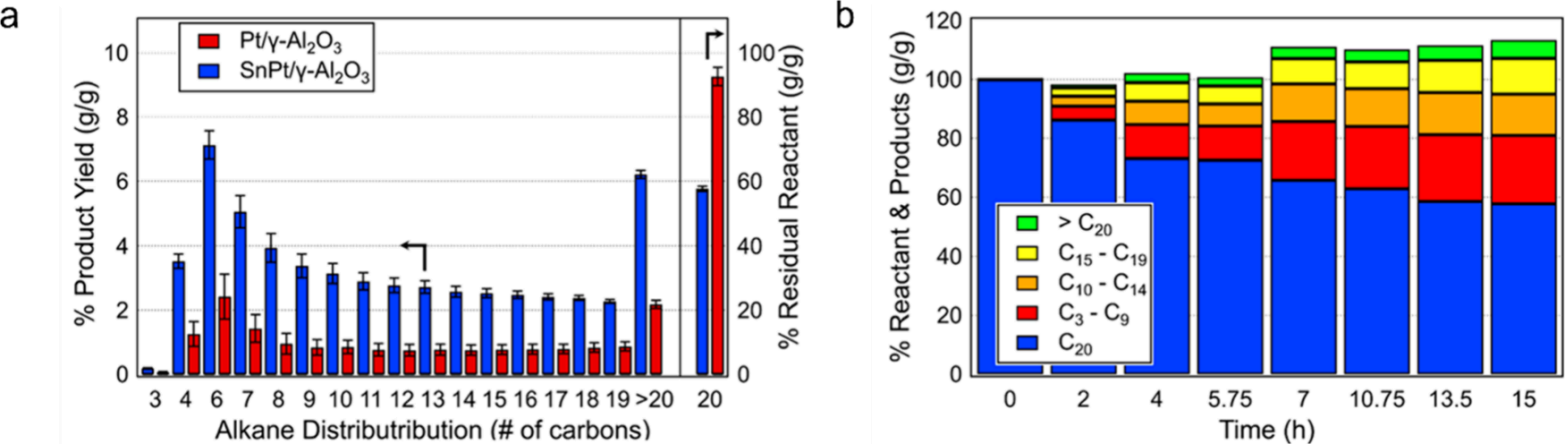
Tandem alkane cross-metathesis of *n*-eicosane (5 wt%, 1 g) with *n*-pentane (ca. 19 g), catalyzed by Re_2_O_7_/γ-Al_2_O_3_ (8 wt% Re, 500 mg) and either PtSn/γ-Al_2_O_3_ (0.8 wt% Pt, 1.7 wt% Sn, 500 mg) or Pt/γ-Al_2_O_3_ (5 wt% Pt, 500 mg) in a batch reactor at 200 °C: (a) distribution of linear alkane products formed after 15 h; and (b) time-course of the product distribution in the tandem reaction catalyzed by PtSn/γ-Al_2_O_3_ and Re_2_O_7_/γ-Al_2_O_3_. Reproduced with permission from ref (345). Copyright 2021, American Chemical Society.

Cross-metathesis of *n*-eicosane (*n*-C_20_H_42_, a surrogate for PE) with excess *n*-pentane was studied in a batch reactor with a physical mixture of catalysts: PtSn/γ-Al_2_O_3_ (0.8 wt% Pt, 1.7 wt% Sn) for dehydrogenation/hydrogenation, and Re_2_O_7_/γ-Al_2_O_3_ (8 wt% Re) for alkene metathesis, in a 1:1 mass ratio (active phase + support).^345^ The results are shown in Figure 55a. After 15 h at 200 °C, the conversion of *n*-eicosane was 41 % and the products were a distribution of linear alkanes from C_3_ to C_35_, with a maximum at C_5_. The conversion of *n*-eicosane was much lower (ca. 6 %) when the dehydrogenation catalyst was Pt/γ-Al_2_O_3_ with fewer active sites (CO monolayer uptake 1640 μmol/g_Pt_, compared to 4960 μmol/g_Pt_ for the PtSn catalyst), although the product distributions were similar. The claimed existence of a Sn promotion effect^350^ on the Pt catalyst is doubtful, since another Pt/γ-Al_2_O_3_ catalyst with 0.8 wt% Pt loading and a CO monolayer uptake of 2660 μmol/g_Pt_ converted 39 % of *n*-eicosane, comparable to PtSn/γ-Al_2_O_3_. Nevertheless, these comparisons suggest that the low temperature activity of the dehydrogenation catalyst is critical in alkane metathesis.

Catalytic activity as a function of time is shown in Figure 55b. Since the total mass of linear alkane products was greater than the mass of *n*-eicosane consumed, cross-metathesis between *n*-eicosane and *n*-pentane was deemed to have occurred. After 13.5 h, the product distribution stopped changing, possibly due to catalyst deactivation (although the cause of deactivation was not investigated). The dehydrogenation catalyst may have deactivated due to carbon deposition, since the post-reaction catalyst mixture was significantly darker in color than the fresh catalysts, and showed a significant reduction (49 %) in active surface area as measured by CO chemisorption. Another possibility is deactivation of the metathesis active sites, due to poisoning by trace impurities and/or by intrinsic deactivation.^356,357^

Catalyst pretreatment had a significant effect on performance. Calcining a mixture of the two catalysts (O_2_, 500 °C, 1 h) resulted in more than twice the conversion of *n*-eicosane, compared to calcining each catalyst separately. Analysis of the calcined mixture by scanning transmission electron microscopy with energy dispersive X-ray spectroscopy showed that both Pt and Re were mobile, with a portion being transferred to the other catalyst during calcination. Therefore, catalysts with both Re and Pt deposited on the same support were explored. Surprisingly, both Re_2_O_7_ deposited on PtSn/γ-Al_2_O_3_ and PtSn deposited on Re_2_O_7_/γ-Al_2_O_3_ gave lower *n*-eicosane conversions (11 and 8 %, respectively) after 15 h at 200 °C. The lower activities were suggested to be due to the formation of inactive alloys (e.g., PtRe and ReSn), resulting in fewer active sites for alkene metathesis. Adding extra Re_2_O_7_/γ-Al_2_O_3_ to either of the codeposited catalysts gave the highest conversion of *n*-eicosane. These observations suggest that migration of Re_2_O_7_ to PtSn/γ-Al_2_O_3_ increased the number of alkene metathesis active sites.

Since Re is expensive and Re_2_O_7_ is volatile, its loss during catalyst regeneration by high temperature calcination is a practical concern. By comparison, WO_x_/SiO_2_ is inexpensive and does not volatilize at high temperatures. It was used in combination with Pt/γ-Al_2_O_3_ for alkane cross-metathesis.^346^ The tandem reaction was conducted in batch using a model compound, *n*-hexadecane (1.58 g) dissolved in *n*-heptane (6.84 g, mol C_7_/mol C_16_ = 9.8), at 300 °C with WO_x_/SiO_2_ (2 wt% W, 0.8 g) and Pt/γ-Al_2_O_3_ (0.5 g, 1 wt% Pt) as catalysts. Zeolite 4A (1.0 g) was added as a sorbent to remove oxygenates formed during activation of the metathesis catalyst, which can poison the catalyst.^358^ After 2 h, the *n*-hexadecane and *n*-heptane conversions were 87 and 28 mol%, respectively. The liquid products (165 mol%, calculated on a *n*-hexadecane basis) consisted of a broad distribution of *n*-alkanes (C_3_–C_16+_). Control experiments included reactions: (1) without catalyst, (2) with only WO_x_/SiO_2_, (3) with WO_x_/SiO_2_ and zeolite 4A. In each case, conversions of both alkane reactants were below 5 mol%. A control experiment with only Pt/γ-Al_2_O_3_ gave 7 mol% conversion of *n*-C_7_H_16_ and 36 mol% conversion of *n*-C_16_H_34_, due to Pt-catalyzed cracking. Both values are low compared to the tandem reaction conducted with both WO_x_/SiO_2_ and Pt/γ-Al_2_O_3_.

#### 4.2.2 Homogeneous Metathesis Catalysts

**Scheme 35:**
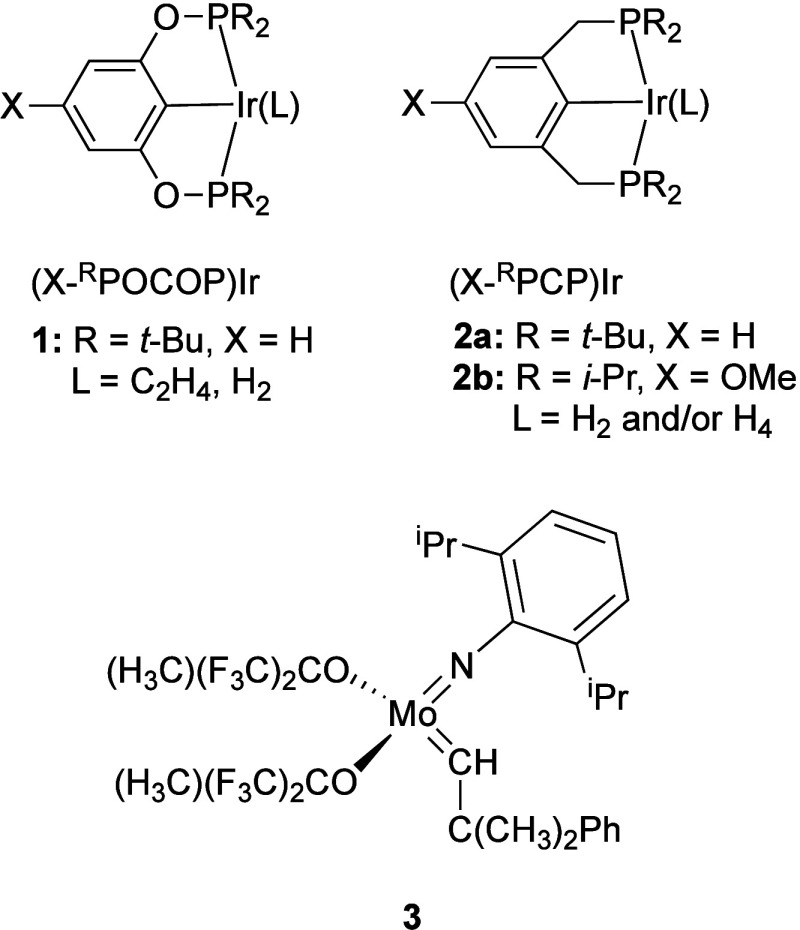
Structures of Homogeneous Dehydrogenation Catalysts (**1** and **2**) Compatible with a Schrock Catalyst (**3**) for Tandem Alkane Metathesis^342^

Alkane metathesis can also be conducted using homogeneous catalysts, under even milder reaction conditions. Self-metathesis of *n*-hexane was achieved at 125 °C using a dual catalyst system consisting of an Ir pincer complex (**1** or **2**) for dehydrogenation/hydrogenation and Schrock catalyst **3** for alkene metathesis (Scheme 35).^342^ The Schrock catalyst was used instead of an easier-to-handle Grubbs’ catalyst because the latter proved incompatible with the Ir catalyst. In a typical experiment, **1**-C_2_H_4_ (12.8 mg, 0.021 mmol), Schrock catalyst **3** (26 mg, 0.034 mmol), and *n*-hexane (2 mL, 15.3 mmol) were heated to 125 °C. After 6 h, the products were exclusively linear alkanes (0.75 M) from C_2_ to C_15_, concentrated in the ranges C_2_–C_5_ and C_7_–C_10_ (no branched or cyclic alkanes were detected). The large number of products suggested that alkene isomerization occurred simultaneous with metathesis (Ir pincer complexes are known to catalyze alkene isomerization).^359^ When the reaction time was extended to 24 h, the product yield doubled, but the distribution did not change significantly. Heating for even longer times (e.g., 2–4 d) resulted in little change in product yield. However, addition of a second aliquot of catalyst **3** caused the yield to increase significantly, suggesting that deactivation of **3** limits the overall productivity of the tandem catalyst system.

Three control experiments were performed with *n*-hexane at 125 °C to establish the necessity of both catalysts for alkane metathesis. No alkane metathesis products were observed after 24 h using (1) only dehydrogenation catalyst **1-C_2_H_4_**; (2) a combination of dehydrogenation catalyst **2a-H_2_** and *tert*-butyl-ethylene (TBE); or (3) only metathesis catalyst **3**.

Cross-metathesis of *n*-hexane (4.4 M) with *n*-eicosane (1.1 M) was carried out using **1-C_2_H_4_** (7 mM) and **3** (11 mM) at 125 °C. After 1 d, the total product concentration was 1.56 M, in a distribution consisting of *n*-alkanes from C_2_ to C_38_ (most being C_2_ to C_20_, 1.35 M). However, the catalyst system was not stable under the reaction conditions, and the total alkane product concentration had increased to only 1.97 M after 6 d.

Low thermal stability of the metathesis catalyst is an important factor limiting conversion. Replacing **3** with a heterogeneous catalyst, Re_2_O_7_/γ-Al_2_O_3_ (8 wt% Re) resulted in better productivity. In a typical experiment, *n*-decane (2.5 mL, 13 mmol) and TBE (10 μL, 0.08 mmol, added as a sacrificial H_2_ acceptor) were heated at 175 °C with **2b-H_4_** (13 mg, 0.023 mmol) and Re_2_O_7_/γ-Al_2_O_3_ (540 mg). The products ranged from C_2_ to C_28_ alkanes. The total product concentration was 1.6 M after 3 h, increasing to 4.4 M after 9 d. At that time, C_9_, C_10_, and C_11_ alkanes were present in comparable concentrations.

#### 4.2.3 Metathesis Catalyzed by Supported “Single-Site” Metal Complexes

Supported hydrides of Zr, Ta, and W can be prepared by protonolysis of appropriate organometallic compounds by hydroxyl-terminated oxide surfaces, followed by hydrogenolysis of the remaining alkyl ligands. The resulting metal hydrides^351,360,361^ are highly coordinatively unsaturated, electron-deficient catalysts that are reactive toward the C–H and C–C bonds of alkanes. In contrast to tandem catalytic systems that combine different catalysts for (transfer) dehydrogenation/hydrogenation and alkene metathesis, these supported metal hydrides are single-component catalysts for alkane metathesis.^347^

**Figure 56:**
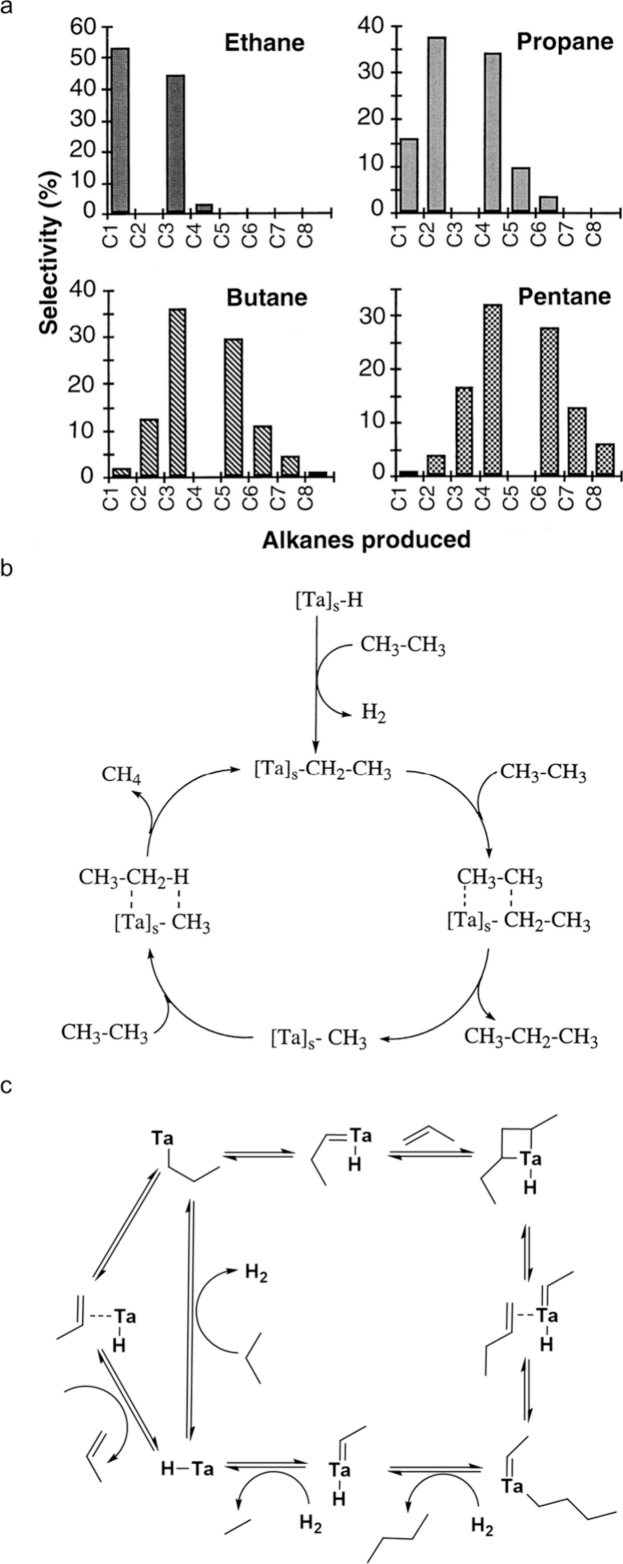
(a) Hydrocarbons produced during the self-metatheses of ethane, propane, *n*-butane, and *n*-pentane (all at 3% conversion), catalyzed by silica-supported [Ta]-H at 150 °C (ethane, propane, or *n*-butane/Ta ∼ 800, P = 1 bar; or *n*-pentane/Ta ∼ 400, P = 0.5 bar), and (b) proposed mechanism for ethane self-metathesis. Reproduced with permission from ref (340). Copyright 1997, AAAS. (c) Proposed mechanism for propane metathesis catalyzed by silica-supported [Ta-H]. Adapted with permission from ref (364). Copyright 2005, American Chemical Society.

In the first claim of single-site metathesis of acyclic alkanes, a supported Ta or W hydride catalyzed the metathesis of light alkanes such as ethane, propane, *n*-butane or *n*-pentane, at temperatures from 25 to 200 °C.^340^ The products were mainly C_*n*+1_ and C_*n*–1_ alkanes, with traces of other alkanes (Figure 56a). For example, ethane metathesis at 150 °C and 1 bar (C_2_H_6_/Ta ∼ 800) catalyzed by silica-supported [Ta]-H yielded comparable amounts of propane and methane, as well as trace amounts of *n*-butane and isobutane. An isotope-labeling experiment with ^13^C-monolabeled ethane yielded unlabeled, mono-labeled, di-labeled, and tri-labeled propane (5:44:43:8), indicating cleavage of the ^13^C–^12^C bond of ethane and redistribution of the C atoms. The formation of traces of *n*-butane and isobutane suggested secondary proane metathesis.

In the metathesis of higher alkanes, the product distribution is broader since there are more C–H and C–C bond types to activate, as well as more secondary reactions. Branched alkanes such as isopentane underwent metathesis with a metal hydride catalyst. However, highly substituted alkanes such as neopentane resulted only in the stoichiometric formation of a surface [Ta]-neopentyl species. The lack of further reaction in the absence of H_2_ was attributed to steric constraints in the transition state for σ-bond metathesis.^340^ Productivity was lowest for isobutane self-metathesis (TON = 17/Ta), compared to 46, 47, and 66 for the self-metatheses of ethane, propane, and *n*-butane, respectively.

The original mechanism proposed for ethane metathesis catalyzed by [Ta]-H started with cleavage of a C–H bond of ethane by σ-bond metathesis, producing H_2_ and an [Ta]-ethyl site (Figure 56b).^362,363^*In situ* IR spectroscopy confirmed the disappearance of the hydride, presumably due to its conversion to [Ta]-ethyl. The next step was σ-bond metathesis between [Ta]-ethyl and ethane. This pathway was deemed plausible due to the highly electrophilic character of the [Ta]-alkyl sites, although there was no experimental or computational evidence to support it at that time. The resulting [Ta]-methyl was proposed to react with ethane in a second σ-bond metathesis step to liberate methane and regenerate [Ta]-ethyl. Note that [Ta]-H was assumed to be the catalyst precursor whose activation of an alkane formed [Ta]-alkyl active sites.

The mechanism was subsequently revised based on a kinetic study in a continuous reactor, which showed that H_2_ and alkenes (from C_2_ to C_5_) were the primary products of propane metathesis.^364^ In the updated mechanism (Figure 56c), the initiation step is the same C–H bond activation of an alkane to form the corresponding [Ta]-alkyl complex and H_2_. However, the [Ta]-alkyl undergoes either α-H elimination to give a [Ta]-alkylidene hydride, or β-H transfer to generate an alkene. The alkene can undergo cycloaddition to the alkylidene, and the resulting tantalacyclobutane can extrude a new alkene to generate a new [Ta]-alkylidene. The catalytic cycle is completed by migration of a hydride onto the new [Ta]-alkylidene, resulting in a [Ta]-alkyl that liberates alkane by hydrogenolysis and also regenerates the [Ta]-H site, which is now an on-cycle intermediate. The revised mechanism is in better agreement with the observed experimental results and DFT calculations.^365^

**Figure 57:**
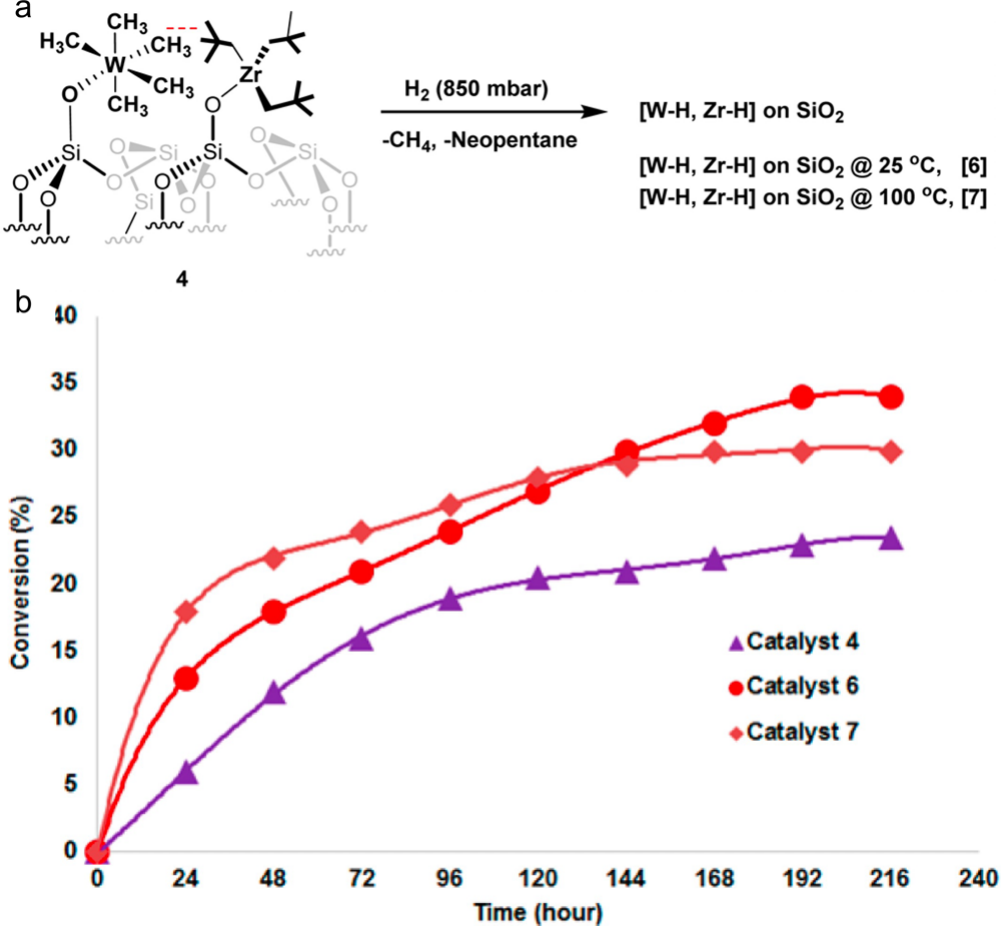
(a) Preparation of a series of bimetallic supported W-Zr hydride catalysts; and (b) kinetic profiles for the self-metathesis of *n*-decane, catalyzed by the bimetallic catalysts. Adapted with permission from ref (366). Copyright 2016, American Chemical Society.

The low overall productivity of the monometallic catalysts was addressed by creating a bimetallic, bifunctional silica-supported catalyst.^366^ Silica-supported WMe_6_ and ZrNp_4_ are independently-known materials.^367,368^ To synthesize the bimetallic precatalyst [≡SiOWMe_5_≡SiOZrNp_3_] (**4**), WMe_6_ was first anchored onto a partially dehydroxylated silica, then ZrNp_4_ was grafted onto this material. A 2D ^1^H-^1^H NMR correlation was observed between methyl groups bonded to W (2.0 ppm) and methyl groups of the neopentyl ligands bonded to Zr (0.9 ppm), confirming that at least some of the metal complexes were grafted near each other (Figure 57a). The catalyst was activated with H_2_ at either 25 °C (**5**) or 100 °C (**6**). According to IR and ^1^H NMR, the methyl groups were fully converted to hydrides at 25 °C, while the neopentyl groups were only partly converted; the neopentyl ligands were, however, fully converted at 100 °C. The process also generated surface [Si]-H_x_ sites by transfer of hydride ligands from metal to Si (accompanied by transfer of O coordination from Si to the metal).

C–H bond activation was suggested to take place on [Zr] sites, with alkene metathesis taking place on [W] sites (thus, no longer qualifying as a single-site catalyst). To compare alkane metathesis activities, *n*-decane (5 mmol) was heated to 150 °C with each catalyst (*n*-decane/W = 4000). The resulting kinetic profiles are shown in Figure 57b. The initial rates were slightly higher for catalysts **5** and **6** than for **4**, presumably because the barriers for initial C–C and C–H bond activation by hydrides **5** and **6** are lower than for the precursor metal alkyls.^369,370^ The fastest initial rate, seen for **5**, is consistent with its lower amount of SiH_x_ sites, implying that more hydride ligands were present on the transition metal when the catalyst was activated at the lower temperature. The bimetallic W/Zr system was more active for *n*-decane metathesis than either monometallic catalyst (Table 5). After 9 d at 150 °C, *n*-decane was 34 % converted by **5**, corresponding to a TON of 1436. A physical mixture of the silica-supported W and Zr hydrides gave a TON of 898. Since this value is lower than for any of the bimetallic catalysts, it indicates synergy between the two co-located metals.

**Table 5:** Comparison of Productivities in *n*-Decane Self-Metathesis, for Monometallic and Bimetallic Silica-Supported Catalysts^a^

catalyst	*n*-decane/W	*n*-decane/Zr	TON^b^
[WH@25°C]	1981	-	650
[ZrH@100 °C]	-	1234	32^c^
[≡SiOWMe_5_≡SiOZrNp_3_] (**4**)	4182	1234	1005
[WH,ZrH@25 °C] (**5**)	4182	1234	1436
[WH,ZrH@100 °C] (**6**)	4182	1234	1250

a All reactions were conducted at 150 °C.

b Turnover number is expressed as mol *n*-decane converted per mol W, except where noted.

c Turnover number is expressed as mol *n*-decane converted per mol Zr.

Adapted with permission from ref (366). Copyright 2016, American Chemical Society.

Compared with the metathesis of lighter alkanes, a broader product distribution was observed in the self-metathesis of *n*-decane. The width of the distribution may be a consequence of alkene isomerization catalyzed by W and/or Zr hydrides. On a molar basis, lower alkanes were formed in preference to higher alkanes, using bimetallic catalyst **5**. The same finding was reported in the self-metathesis of *n*-decane catalyzed by an Ir pincer complex in combination with Re_2_O_7_/γ-Al_2_O_3_.^371^ However, the average carbon number should not change from its starting value in the alkane mixture. One possibility is that the yield of heavy alkanes was underestimated, because these products are harder to detect by GC. Since no mass balance was provided, it is not possible to know if a part of the product distribution was missing.

**Figure 58:**
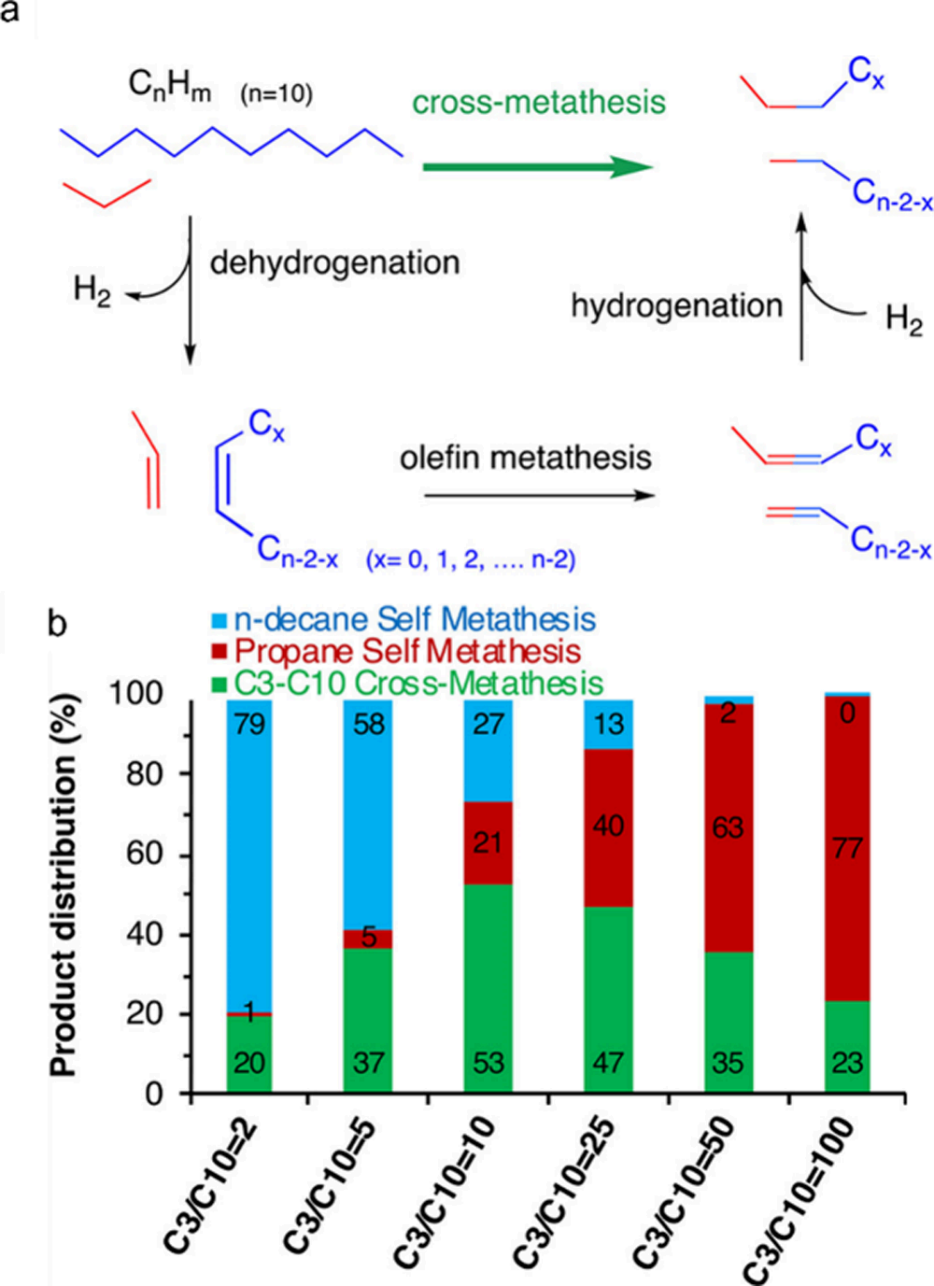
(a) Overall scheme for cross-metathesis between propane and *n*-decane, and (b) influence of the C_3_/C_10_ molar ratio on the product distribution. Reaction conditions: propane (0.5 mL/min), fully deuterated *n*-decane (in Ar carrier gas, 10 mL/min), molar ratio C_3_/C_10_ = 2-100, [(≡SiO)W(CH_3_)_2_(H)_3_] catalyst (150 mg, 1.5 wt% W), 150 °C. Adapted with permission from ref (372). Copyright 2019, American Chemical Society.

Cross-metathesis between a light alkane (propane) and a heavy alkane (*n*-decane) was catalyzed by a silica-supported tungsten hydride (Figure 58a).^372^ A study of the self-metathesis of each alkane led to two insights: (1) *n*-decane self-metathesis yields a very broad product distribution, consisting of alkanes in the range C_2_–C_19_, in contrast with propane, which forms ethane and *n*-butane selectively, and (2) *n*-decane self-metathesis is considerably faster (8×) than propane self-metathesis. Cross-metathesis of propane/*n*-decane mixtures (with C_3_/C_10_ molar ratios of 2-100) gave broad product distributions. Isotopically-labeled alkanes were used to prove that cross-metathesis was occurring. In a typical experiment, ^13^C-labeled propane and non-labeled *n*-decane were combined in a molar ratio C_3_/C_10_ = 2.25 in a batch reactor with [≡SiOW(CH_3_)_2_H_3_] and allowed to react at 150 °C for 5 d. All hydrocarbon products in the range C_4_–C_19_ were ^13^C-enriched, according to mass spectrometry analysis.

To establish how much of the hydrocarbon products arose from cross-metathesis, experiments with mixtures of fully labeled *n*-decane and unlabeled propane were conducted. Three control experiments were carried out in a batch reactor, to rule out the possibility of H/D exchange resulting in partially deuterated hydrocarbons which would complicate the analysis of cross-metathesis products: (1) self-metathesis of unlabeled *n*-decane was conducted in the presence of D_2_, giving alkane products whose mass spectra were identical with those of the unlabeled alkanes; (2) self-metathesis of fully deuterated *n*-decane was conducted in the presence of H_2_, and only fully deuterated alkane products were observed; and (3) in the cross-metathesis of fully deuterated propane and unlabeled *n*-decane, the molecular weights of the long-chain alkane products increased up to 8 units (m/z), suggesting the incorporation of deuterated propane into the products.

Experiments with mixtures of fully deuterated *n*-decane and unlabeled propane were conducted with the tungsten hydride catalyst at 150 °C in a fixed-bed reactor. Based on the control results, cross-metathesis products should be partially deuterated, while self-metathesis products from *n-*decane should be fully deuterated and propane self-metathesis products should be undeuterated. In the flow reactor, propane was introduced first, to promote the formation of metathesis active sites, then *n*-decane was introduced after 2 h. Figure 58b shows the product distribution as a function of the C_3_/C_10_ molar ratio. The fraction of cross-metathesis products increased initially, reaching a maximum of ca. 50 % at C_3_/C_10_ = 10. Further increases in the C_3_/C_10_ ratio led to a decrease in the fraction of cross-metathesis products.

**Scheme 36:**
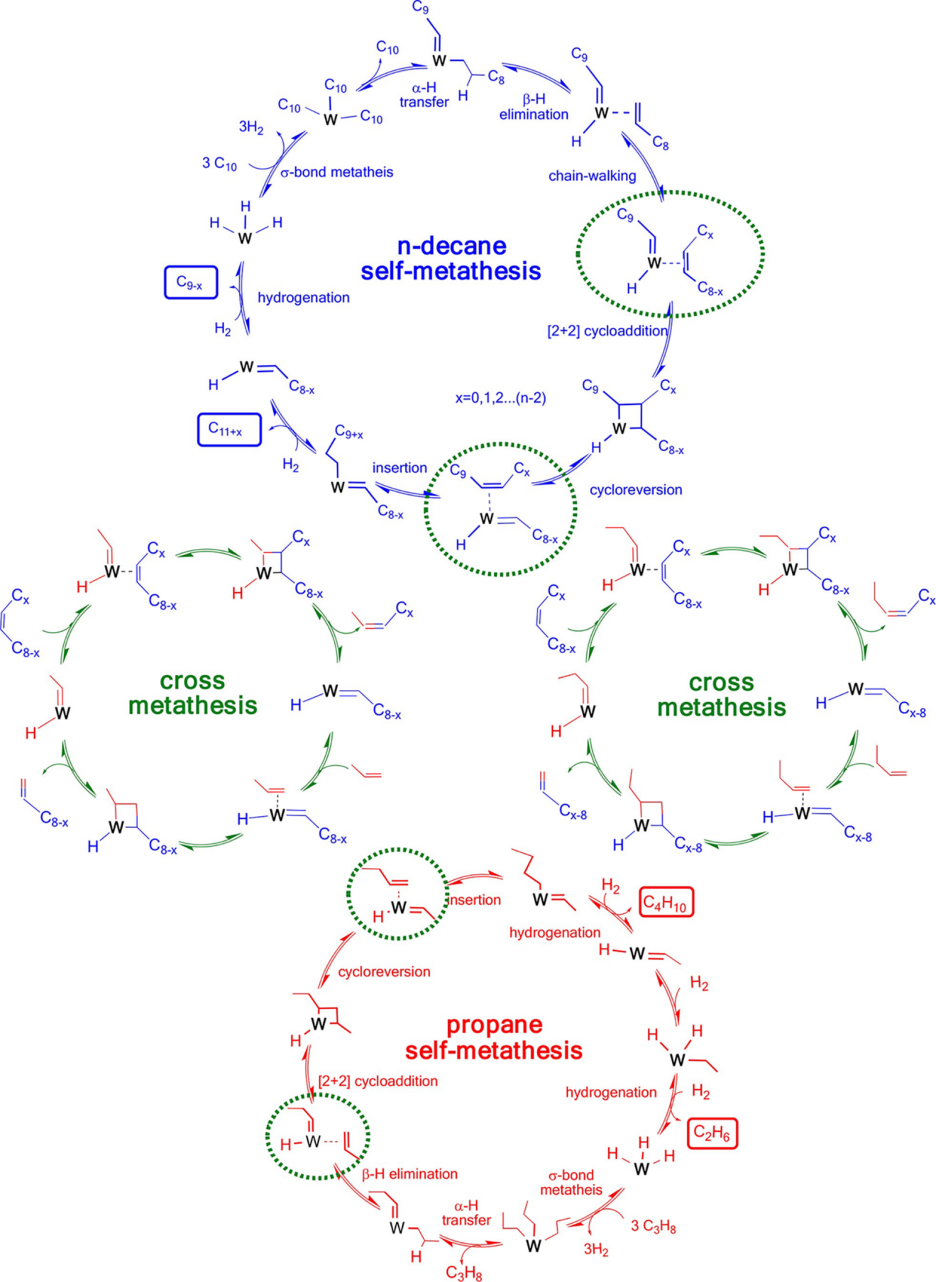
Proposed Mechanism for Cross-Metathesis Between *n*-Decane and Propane, Catalyzed by Silica-Supported “Single-Site” W Including competing self-metathesis reactions. Reproduced with permission from ref (372). Copyright 2019, American Chemical Society.

A general mechanism for single-site alkane cross-metathesis is proposed in Scheme 36,^372^ in which C–H bonds are activated by σ-bond metathesis.^373^ This reaction is followed by α-H elimination, producing a metal-alkylidene, or by β-H elimination, liberating an alkene and H_2_ as primary products. Alkenes react with either of the two hydrido-metal alkylidenes to form hydrido-metallacyclobutanes, which can undergo cycloreversion to give new alkenes and hydrido-metal alkylidenes. Cross-metathesis competes with self-metathesis. The mechanism also includes isomerization, via chain walking and double bond migration, which broaden the product distribution. Finally, an alkene inserts into the metal hydride bond, and/or the hydride migrates onto an alkylidene ligand.

#### 4.2.4 Promoting Alkane Dehydrogenation with a H_2_ Acceptor

Acceptorless alkane dehydrogenation to alkene is highly endothermic, requiring elevated temperatures to achieve significant conversion unless coupled with a similarly exothermic reaction. Hydrogenation of TBE provides such an exothermic reaction, turning dehydrogenation into transfer dehydrogenation. The use of TBE as a sacrificial H_2_ acceptor was first reported in 1979.^374^ Refluxing [IrH_2_(acetone)_2_(PPh_3_)_2_]^+^ in 1,2-dichloroethane containing cyclopentane and TBE for 18 h produced an cyclopentadienyl complex. In tandem alkane metathesis, the use of a sacrificial alkene as H_2_ acceptor can assist in performing alkane dehydrogenation at lower temperatures, thereby making the overall process more compatible with temperature-sensitive alkene metathesis catalysts.

The nature of the H_2_ acceptor affects both catalytic activity and selectivity. Various H_2_ acceptors were explored in the transfer dehydrogenation of *n*-octane catalyzed by (^tBu^P^H^PP)IrH_3_ (2.5 mM).^375^ At 80 °C with TBE (0.2 M), various octenes (21 mM) were produced in 40 min. When the temperature was increased to 100 °C, the octenes yield was 40 mM after just 10 min. The use of 1-hexene (0.2 M) as the H_2_ acceptor was more effective: at 100 °C, 60 mM octenes were formed in 6 min. The rate of *n*-octane transfer dehydrogenation slowed as 1-hexene isomerized to 2-hexene, since internal alkenes are less effective than terminal alkenes as H_2_ acceptors.^376^ Compared to 1-hexene, 4,4-dimethyl-1-pentene was a better H_2_ acceptor, since it is too bulky to isomerize readily to an internal alkene. At 100 °C, *n*-octane transfer dehydrogenation to 4,4-dimethyl-1-pentene (0.2 M) was complete in 10 min. At 80 and 50 °C, the same reaction was complete in 30 and 300 min, respectively. Computational studies showed that the overall barrier for transfer dehydrogenation of *n*-alkanes is 25 kJ/mol lower for 4,4-dimethyl-1-pentene, compared to TBE.

4-Methyl-1-pentene was also screened as a potential H_2_ acceptor, but the isopropyl subgroup was not sufficiently bulky to prevent 1,2-double-bond isomerization, and its performance was poor. Two vinyl ethers were also investigated: *tert*-butyl vinyl ether and *n*-butyl vinyl ether. Both resulted in lower activity than 4,4-dimethyl-1-pentene, presumably due to their ability to bind strongly to the catalyst.^377^ Based on these findings, efficient H_2_ acceptors are characterized by: (1) weak bonding to metal centers, (2) low barriers to hydrogenation, and (3) slow C=C bond isomerization. Ethylene and propylene are light, inexpensive, and sterically unhindered alkenes that can also be used as H_2_ acceptors. They do not isomerize, are readily separated from liquid products, and can be regenerated in a separate dehydrogenation step.^353,378^ For example, ethylene (4 atm) or propylene (2 atm) were used as H_2_ acceptors in the transfer dehydrogenation of *n*-pentane catalyzed by [(^*t*Bu3Me^PCP)Ir(C_2_H_4_)], forming 1-pentene at 240 °C.^379^

#### 4.2.5 Effect of Alkane Chain Length

**Figure 59:**
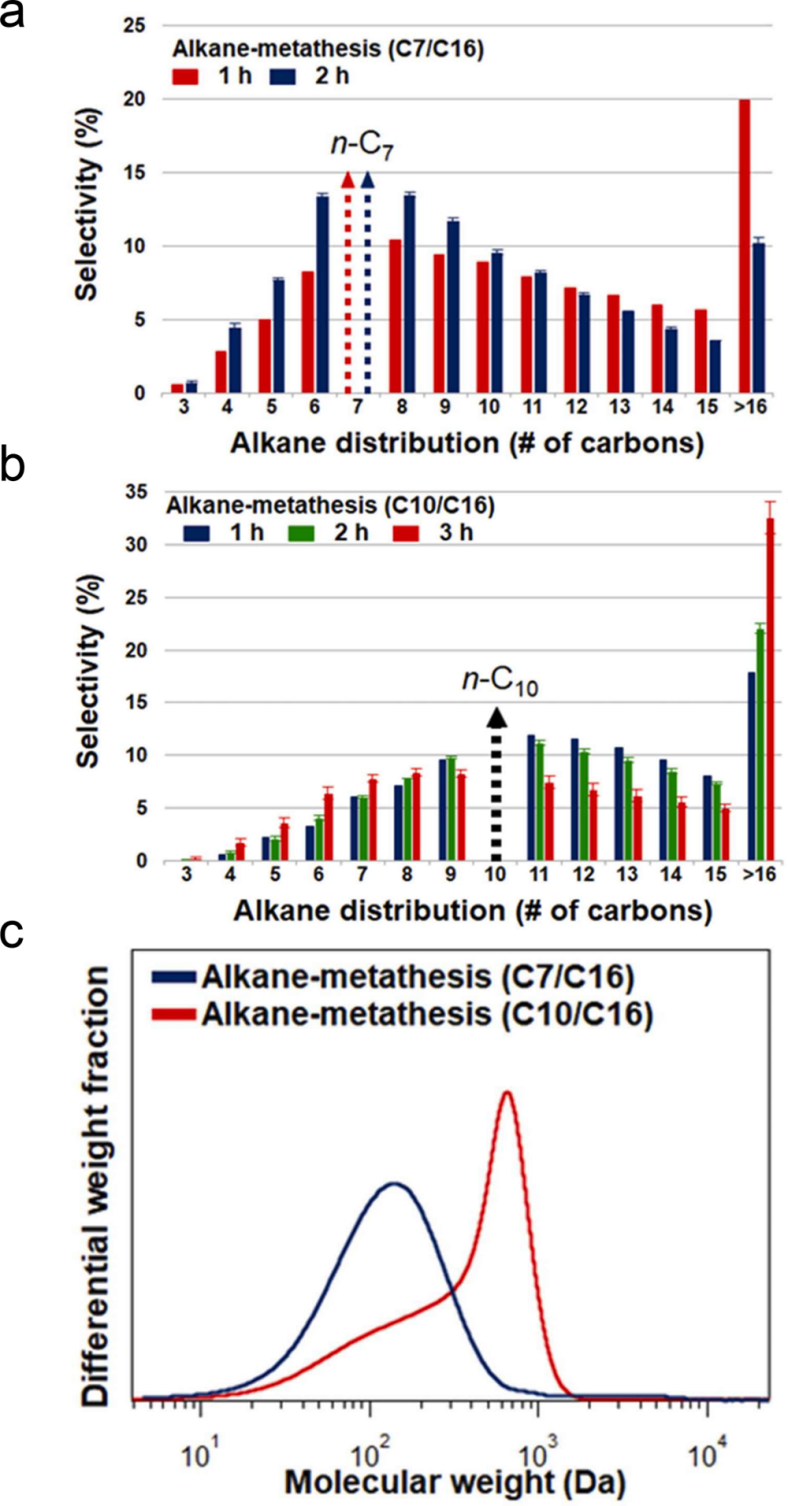
Selectivity for liquid alkane products as a function of time, in alkane cross-metathesis between *n*-C_16_H_34_ and either (a) *n*-C_7_H_16_; or (b) *n*-C_10_H_22_. (c) Differential molecular weight distributions (as measured by high temperature GPC) of the post-reaction solids from C_7_/C_16_ cross-metathesis after 2 h; and from C_10_/C_16_ cross-metathesis after 3 h. Adapted with permission from ref (346). Copyright 2022, Elsevier.

Short-chain alkanes are sources of the light alkenes in cross-metathesis with longer alkenes, and also as solvents for POs. Solvents improve heat/mass transport but also serve to solubilize POs. For example, higher activity for HDPE hydrogenolysis was observed at 220 °C in *n*-hexane compared to *n*-pentane.^125^ HDPE was proposed to be poorly solvated by supercritical *n*-pentane at the reaction temperature. The effect of hydrocarbon chain length was studied in the cross-metathesis of *n*-C_16_H_34_ (considered to be a model for PE) with either *n*-C_10_H_22_ or *n*-C_7_H_16_.^346^ Use of *n*-decane caused the liquid products to shift towards higher molecular weight alkanes (C_>16_) as the reaction proceeded, while *n*-heptane caused the selectivity for higher molecular weight alkanes to decrease with time (Figure 59a,b). Characterization of the post-reaction solids by high temperature GPC showed a bimodal molecular weight distribution was generated in the reaction with *n*-decane: one mode was centered at ca. 100 g/mol, with another at ca. 700 g/mol (waxes), Figure 59c. (The former represents carbon numbers less than 8, which are liquids not removed prior to GPC characterization.) In the reaction with *n*-heptane, the product molecular weight was centered at 100 g/mol (liquid products), with only a small amount of wax with *M*_w_ < 700 g/mol. The wax yield was higher starting from *n*-decane than from *n*-heptane. The increased solubility of longer chains in *n*-decane may enhance alkane metathesis between *n*-decane and waxy intermediates, favoring products with molecular weights of ca. 700 g/mol under the reaction conditions. In contrast, cross-metathesis with *n*-heptane failed to generate much wax, due to the insolubility of longer chains in *n*-heptane.

In its cross-metathesis with *n*-hexadecane, *n*-decane reacted faster than *n*-heptane, as judged by the ratio of the number of reacted short-chain alkanes to reacted C_16_, *r*_short_/*r*_C16_. The different reactivity in *n*-decane vs. *n*-heptane was ascribed not only to the distinct phase behaviors of the short alkane chains under the reaction conditions but also to the influence of their carbon chain lengths on adsorption and on the intrinsic rates of alkane dehydrogenation and/or alkene metathesis.^380^ During cross metathesis with *n*-hexadecane, more *n*-decane was incorporated into longer chains in the final product, in comparison to *n*-heptane. However, no isotopic labeling experiments or control reactions were conducted to establish how much product was derived from cross-metathesis between *n*-hexadecane and *n*-decane, vs. *n*-decane self-metathesis.

#### 4.2.6 Tandem Ethenolysis/Isomerization of Model Alkenes

**Scheme 37:**
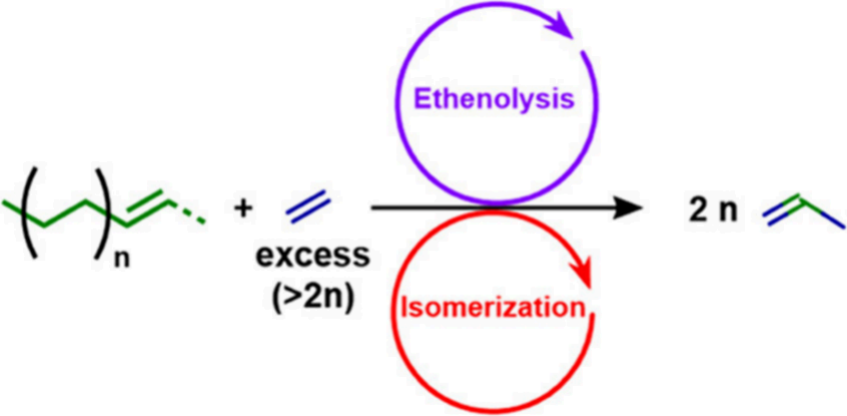
Tandem Ethenolysis/Isomerization of an Unsaturated Alkene to Propylene Reproduced with permission from ref (344). Copyright 2022, American Chemical Society.

Alkene cross-metathesis with ethylene (i.e., ethenolysis) of linear mono-alkenes can be coupled with alkene isomerization of terminal to internal alkenes. Repeated, tandem ethenolysis-isomerization of long-chain alkenes can be highly selective to propylene (Scheme 37). The principle was first described in a paper that predicted propylene formation rates and the evolution of polymer molecular weight as a function ofr alkene metathesis and isomerization rates.^381^ For a constant rate of alkene isomerization, the propylene formation rate should increase with the metathesis rate, since the latter is a function of the ethylene partial pressure. However, the increase will not be linear, because only chains with internal double bonds react to generate propylene and other light alkenes; ethenolysis of terminal alkenes is degenerate. The rate of propylene production and the reaction order in ethylene were analyzed using a microkinetic model in which ethenolysis was fast compared to isomerization.^382^ The overall rate can be first-, zeroth- or even negative order in ethylene concentration. Ethylene inhibition arose due to unproductive formation of the unsubstituted metallacyclobutane, competing with formation of substituted metallacyclobutanes that lead to propylene.

**Figure 60:**
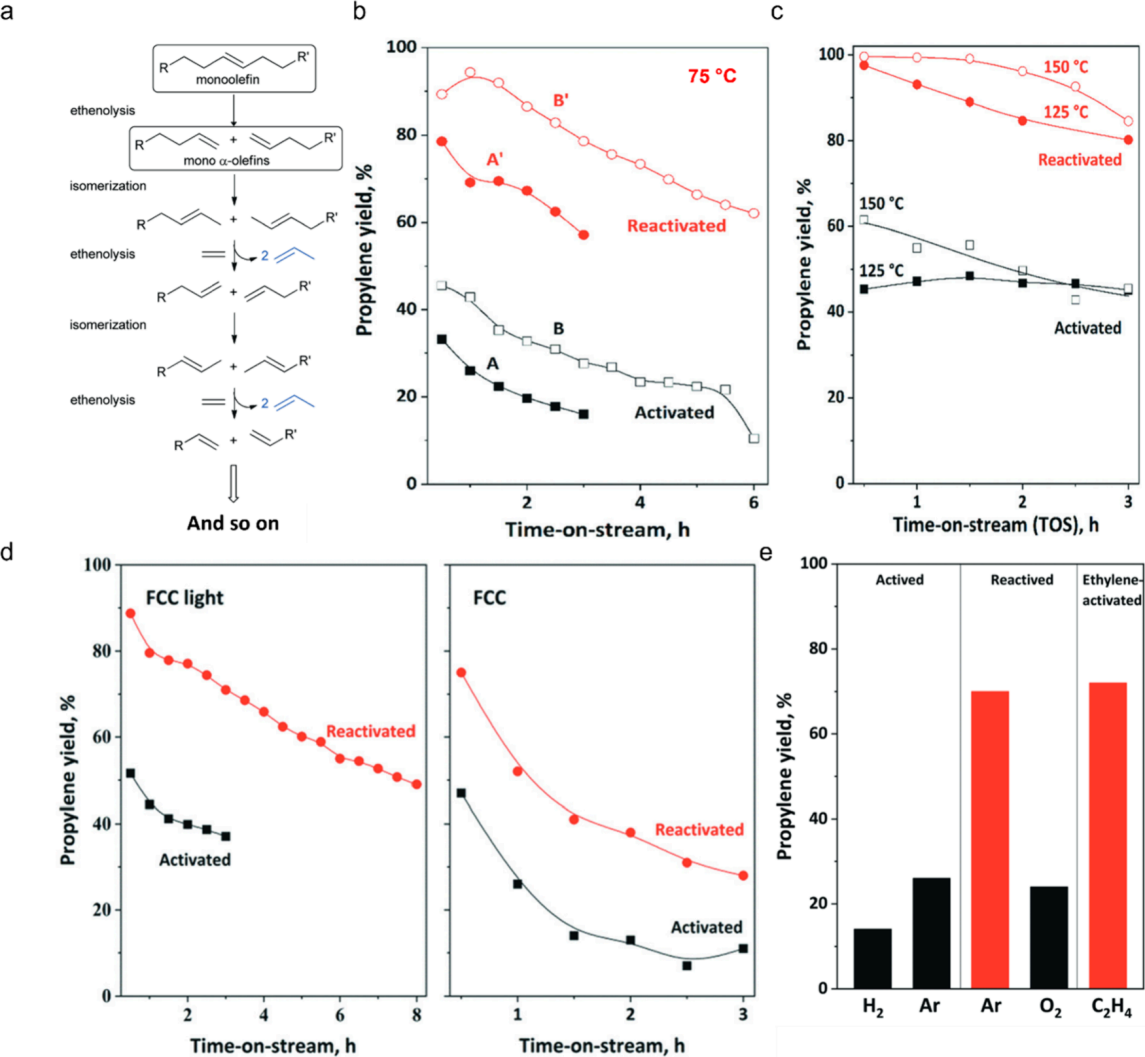
(a) Scheme for C_6+_ alkene conversion to propylene via tandem ethenolysis-isomerization; and (b–c) propylene yields from tandem ethenolysis-isomerization of 1-hexene, catalyzed by a physical mixture of activated (and reactivated) HBEA and MoO_3_/γ-Al_2_O_3_ (12 wt% Mo) at various temperatures (A and A′ correspond to a space time of 6 g_cat_ g_hexene_^–1^ h; B and B′ to a space time of 12 g_cat_ g_hexene_^–1^ h). (d) Evolution of propylene yield in tandem ethenolysis-isomerization of FCC light and crude FCC, catalyzed by a physical mixture of activated (and reactivated) HBEA and MoO_3_/γ-Al_2_O_3_ (12 wt% Mo), as a function of time on-stream at 75 °C under 3 bar ethylene, with a space-time of 6 g_cat_ g_alkene_^–1^ h. (e) Propylene yields in tandem ethenolysis-isomerization of 1-hexene using activated and reactivated 12MoO_3_/Al_2_O_3_-HBEA catalyst mixtures, as a function of catalyst pretreatment. Reaction conditions: 3 bar ethylene, ethylene/1-hexene molar ratio of 10, 75 °C, 1 h time on-stream, space time 6 g_cat_ g_hexene_^–1^ h. Adapted with permission from ref (383). Copyright 2021, Royal Society of Chemistry.

The approach was first demonstrated experimentally in a continuous flow reactor for the conversion of 1-hexene, as well as a mixture of heavier alkenes (C_>5_) derived from the fluid catalytic cracking (FCC) of naphtha (Figure 60a).^383^ Several heterogeneous catalysts were explored: MoO_3_/HBEA (6 or 13 wt% Mo, Si/Al = 12), MoO_3_/γ-Al_2_O_3_ (12 wt% MoO_3_), and a physical mixture of HBEA and MoO_3_/γ-Al_2_O_3_. Mo contributed the metathesis activity, while HBEA catalyzed 1-hexene isomerization. Activity and stability declined upon impregnation of the zeolite with MoO_3_, since the metal oxide partially consumed the Brønsted acid sites which are the active sites for alkene isomerization. Consequently, subsequent studies were performed with a physical mixture of catalysts.

The activity and stability of the physical catalyst mixture were studied at 75–150 °C, for an ethylene:1-hexene molar ratio of 10. At 75 °C, the propylene yield was ca. 33 % after 0.5 h but decreased to 18 % over 3 h, Figure 60b,c. When the used catalyst mixture was regenerated in Ar at 550 °C, the propylene yield increased to 80 % but then declined to 57 % over the next 3 h (Figure 60b). Rapid deactivation was attributed to alkene oligomerization, which creates carbonaceous deposits that block access to the active sites located inside the HBEA pores. In addition, formation of an inactive crystalline phase of MoO_3_ under the reaction conditions decreased the number of metathesis active sites. At a higher reaction temperature (125 °C), the propylene yield was higher and the catalyst activity did not change much as a function of time on-stream (Figure 60c). Again, the reactivated catalyst appeared to be more active, but activity and stability cannot be judged reliably since the reaction was conducted at near-full conversion.^384^

Propylene production from the alkene mixture produced by FCC cracking of naphtha was also demonstrated. Materials described as crude FCC and FCC light had alkene contents of ca. 22 and 37 wt%, respectively (mainly C_5_–C_7_), with a large fraction (ca. 50 %) being internal alkenes. Tandem ethenolysis-isomerization was carried out with the 12MoO_3_/Al_2_O_3_-HBEA catalyst mixture at 75 °C under 3 bar ethylene. Due to its higher alkene content and purity, the propylene yield from FCC light was higher. The reactivated catalyst was again more active and showed a longer catalyst lifetime than the freshly activated catalyst, Figure 60d**.**

Improved activity of the catalyst reactivated in Ar at 550 °C was attributed to partial reduction of Mo(VI) to Mo(V), observed by XPS. No improvement in activity was found when the catalyst was reactivated in O_2_ instead. Pre-treatment with ethylene at 75 °C for 1 h followed by Ar for 0.5 h, then heating in Ar at 550 °C for 2 h, also resulted in a significant improvement in activity (Figure 60e). These findings suggest that residual alkenes in the catalyst bed reduced Mo to an optimal oxidation state during the high temperature treatment. Similar observations were made for silica-supported MoO_3_ and WO_3_ metathesis catalysts pre-treated with propylene at high temperatures.^385^ The propylene yield was low (ca. 15 %) after pretreatment in H_2_ (5 % in Ar), suggesting it is possible to over-reduce the metathesis catalyst. However, the low fraction of active sites has made it challenging to identify the precise Mo sites which perform alkene metathesis.

**Scheme 38:**
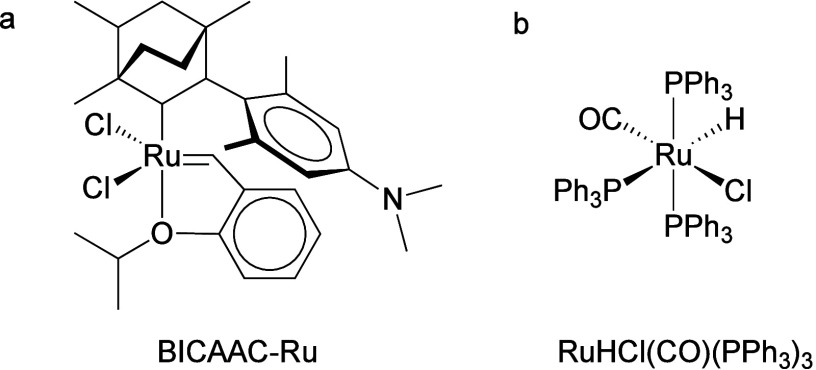
Structures of Homogeneous Catalysts Used in Tandem Isomerization-Metathesis of 1-Octadecene: (a) Bicyclic (Alkyl)(amino)Ru Carbene Catalyst (for Alkene Metathesis), and (b) Ru Hydride Catalyst (for Alkene Isomerization) Adapted with permission from ref (386). Copyright 2022, Wiley-VCH GmbH.

A high yield of propylene from linear 1-alkenes typical of PE pyrolysis oils was achieved by tandem ethenolysis-isomerization at lower temperatures, using a combination of homogeneous catalysts (Scheme 38). In the presence of a thermally stable bicyclic (alkyl)(amino)Ru carbene complex (BICAAC-Ru) for alkene metathesis and RuHCl(CO)(PPh_3_)_3_ for alkene isomerization, 1-octadecene was converted to propylene by reaction with excess ethylene at 75 °C.^386^ In a typical experiment, the metathesis catalyst (0.098 mg in 100 μL toluene, 0.001 mol%,), isomerization catalyst (3 mg, 0.02 mol%), 1-octadecene (4 g, 15.8 mmol), and toluene (3 mL) were combined under Ar. The reactor was charged with 10 bar ethylene (99.9 %), then heated to 75 °C for 24 h. A typical result was a TON of 26,000 mol_propylene_ mol_cat_^–1^ (normalized by the amount of metathesis catalyst).

A higher propylene yield from 1-octadecene was obtained by increasing the concentration of the isomerization catalyst from 20 to 2000 ppm. The TON increased from 7,600 to 36,000 mol_propylene_ mol_cat_^–1^, suggesting rate-limiting alkene isomerization. The ethylene purity also strongly influenced catalytic activity. Changing its purity from 99.9 to 99.995 % caused the propylene yield to double (from 26,000 to 51,000 mol_propylene_ mol_cat_^–1^). With ultra-high purity ethylene (99.995 %), and at a metathesis catalyst loading of only 1 ppm, the turnover numbers were 30,000 mol_propylene_ mol_cat_^–1^ after 24 h and 50,000 mol_propylene_ mol_cat_^–1^ after 96 h. The ethylene impurity that deactivates one or both homogeneous catalysts was not identified.

### 4.3 Metathesis Involving Polyolefins

Chemically, POs are simply ultra-long alkanes. Consequently, cross-metathesis of a PO with a short alkane converts the PO to alkanes with shorter chain lengths. Depending on the polymerization process used to make them, POs may be fully saturated (e.g., polymers made by Ziegler–Natta,^387^ metallocene, and post-metallocene catalysts,^388,389^ where chain termination is achieved with H_2_), or very lightly unsaturated (e.g., polymers made by Phillips catalysts,^390^ which self-terminate without H_2_). In the former case, the unsaturation needed for alkane metathesis can be introduced by acceptorless dehydrogenation, transfer dehydrogenation, or bromination/dehydrobromination. Examples of each are described below.

#### 4.3.1 Transfer Dehydrogenation of Saturated Polyolefins

**Scheme 39:**
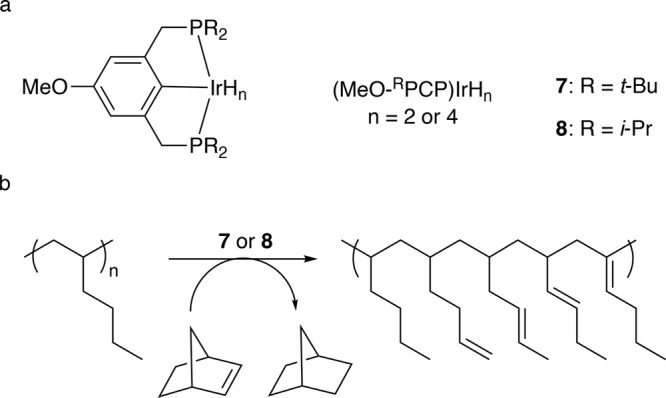
(a) Structures of Two PCP-Pincer Ir Catalysts; and (b) Transfer Dehydrogenation of Poly(1-Hexene), Accompanied by C=C Bond Isomerization Adapted with permission from ref (391). Copyright 2005, Royal Society of Chemistry.

Transfer dehydrogenation introduces C=C bonds along saturated PO chains, priming them for further reaction. The dehydrogenation of aliphatic POs to partially unsaturated hydrocarbon polymers was first reported in 2005,^391^ using *p*-methoxy-substituted PCP-pincer iridium complexes as catalysts, Scheme 39a. In a typical experiment, a *p*-xylene solution of catalyst **7** (5 mM), norbornene (NBE, 0.21 M, used as a sacrificial hydrogen-acceptor), and poly(1-hexene) (PH, 1.1 M in monomer repeat units, *M*_n_ = 6,900 g/mol, *Đ* = 1.5) were sealed in a tube and immersed in an oil bath at 150 °C. After 20 min, terminal vinyl groups (10 mM) and internal C=C bonds (30 mM) were observed by ^1^H NMR, as multiplets at 5.1/5.8 ppm, and 5.45 ppm, respectively. After 260 min, the concentration of double bonds in PH was 0.13 M, of which most (0.12 M) were internal; far fewer (0.007 M) were terminal (as expected, based on their relative thermodynamic stabilities). The consumption of NBE, 0.13 M, was equivalent to the extent of dehydrogenation. Under identical reaction conditions, PCP-pincer catalyst **8** gave a higher yield of C=C bonds in PH than **7**.

After 20 min at 150 °C, the concentrations of terminal and internal double bonds formed by **8** were 39 and 56 mM, respectively. After 50 min, all NBE was consumed and the total concentration of PH double bonds was ca. 0.2 M. Since isomerization is fast relative to dehydrogenation, no terminal vinyl groups were detected in the ^1^H NMR spectrum, Scheme 39b. Size-exclusion chromatography revealed that the PH molecular weight and distribution before and after transfer dehydrogenation were essentially unchanged: (*M*_n_ 6,540 vs. 6,680 g/mol, *M*_w_ 10,250 vs. 10,680 g/mol), confirming that chain scission was negligible.

The same method was used to dehydrogenate a saturated PE. A solution of catalyst **8** (15 mM), NBE (0.21 M) and a low molecular weight PE (3.2 M monomer units, *M*_n_ = 606 g/mol, *M*_w_ = 3,000 g/mol) was heated to 150 °C. After 230 min, the degree of dehydrogenation was 4.4 % (corresponding to 0.14 M double bonds), while no significant chain scission was observed.

**Scheme 40:**
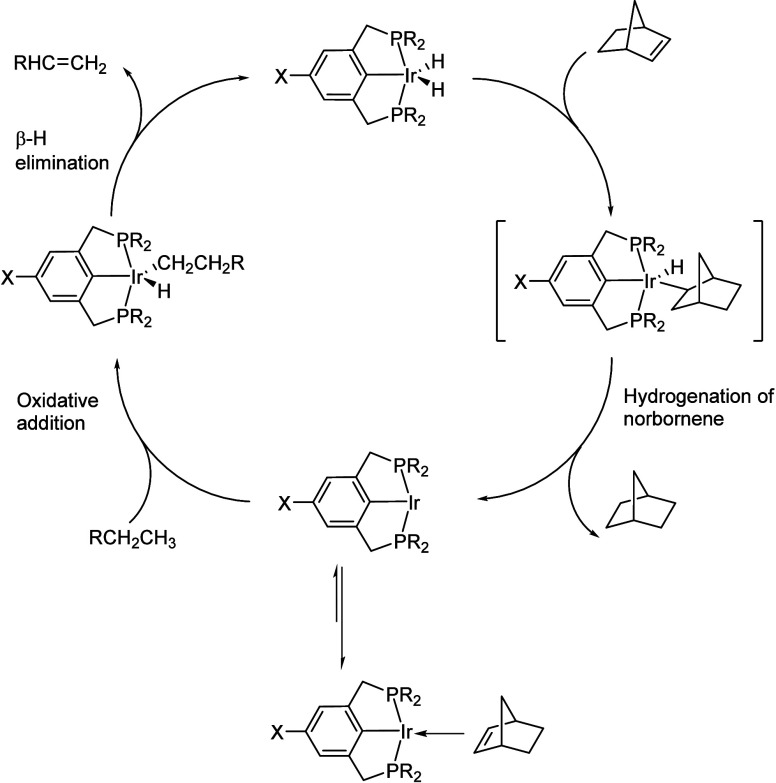
Mechanism of Transfer Dehydrogenation between Poly(1-hexene) and Norbornene, a Sacrificial Hydrogen Acceptor, Catalyzed by a Pincer-PCP Ir Complex Reproduced with permission from ref (392). Copyright 2006 Elsevier.

A kinetic study of PH dehydrogenation was undertaken using the same two Ir pincer catalysts.^392^ The proposed mechanism is shown in Scheme 40. It involves: (1) NBE hydrogenation with formation of coordinatively-unsaturated (*p*-MeO-^R^PCP)Ir (not observed experimentally, presumably due to its low concentration); (2) dynamic equilibrium between (*p*-MeO-^R^PCP)Ir without and with coordinated NBE; and (3) dehydrogenation of an ethyl group of PH. Dehydrogenation was assumed to occur exclusively on the side-chains, since their concentration is much higher than that of the terminal monomer units in PH (the average number of monomer units per polymer chain was ca. 80). The kinetic model also accounted for C=C bond isomerization, and irreversible deactivation of the catalyst. The results are shown in Table 6. Compared to catalyst **7**, hydrogenation of NBE was ca. 5× faster for catalyst **8**, and its deactivation was neglected. The rates of PH dehydrogenation and double bond isomerization were both ca. 2× faster for catalyst **8**.

**Table 6:** Estimated Rate Constants^a^ for the Transfer Dehydrogenation of Poly(1-hexene) Catalyzed by PCP-Pincer Ir Catalysts **7** and **8**^b^

reaction	rate constant	catalyst **7**	catalyst **8**
hydrogenation of NBE	*k*_h_ (M^–1^ min^–1^)	3.8	18.6
dehydrogenation of PH	*k*_dh_/*K*_1_ (min^–1^)	0.15	0.3
isomerization of C=C bond	*k*_iso_ (min^–1^)	0.06	0.15
catalyst deactivation	*k*_deact_/*K*_1_ (min^–1^)	0.01	–

a The ratio of binding constants to Ir for NBE vs. dehydrogenated polymer, *K*_2_/*K*_1_, was assumed to be 5 for both catalysts.

b Reaction conditions: PH (1–2 M monomer units) dissolved in *p*-xylene, Ir catalyst (5 mM), NBE (0.2 M), 150 °C.

Adapted with permission from ref (392). Copyright 2006, Elsevier.

#### 4.3.2 Cross-Metathesis of Saturated Polyethylene with a Light Alkane

**Figure 61:**
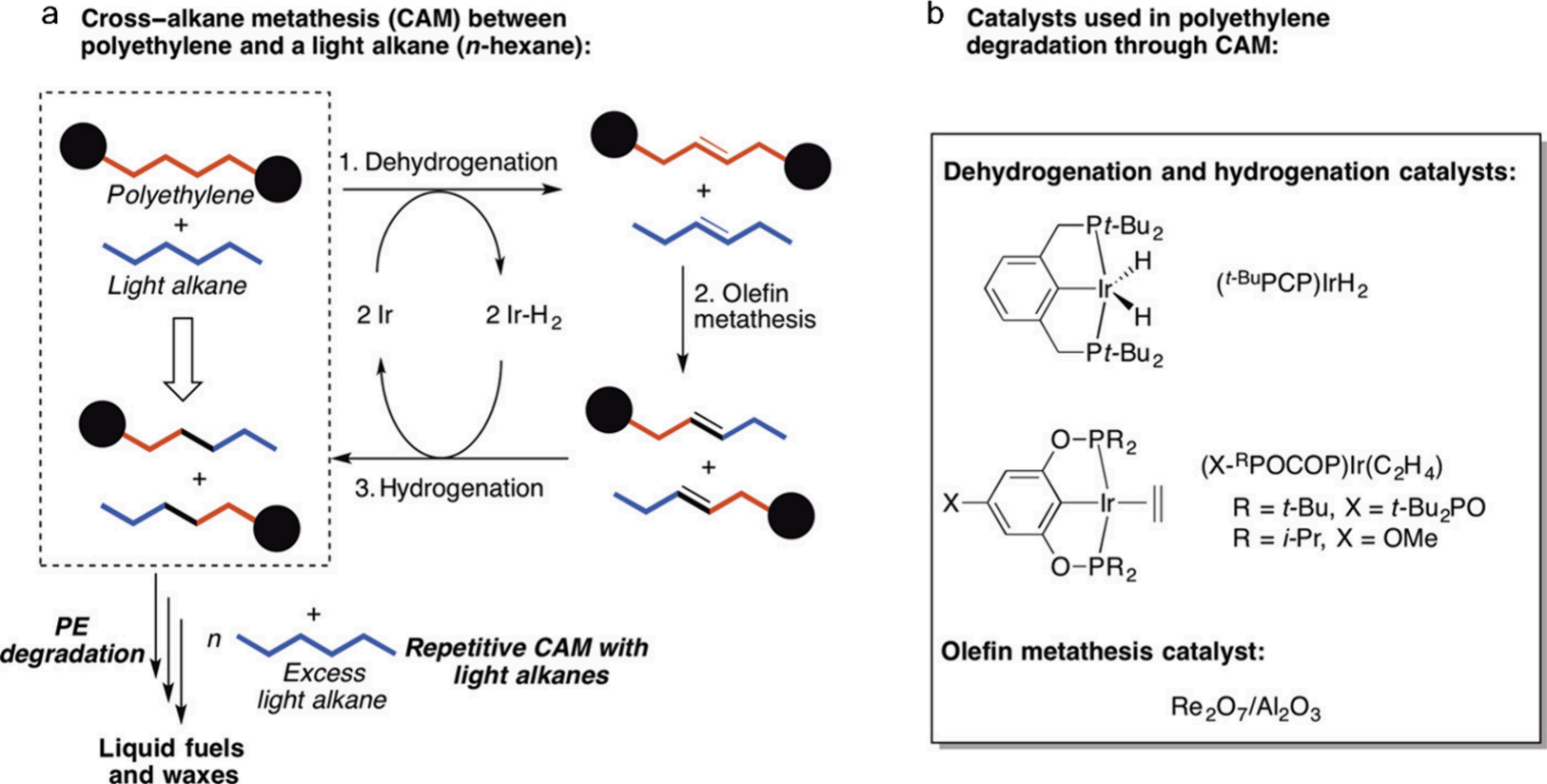
(a) Mechanism of cross-metathesis between PE and a light alkane; and (b) structures of the homogeneous alkane dehydrogenation/alkene hydrogenation catalysts, and formula for the heterogeneous alkene metathesis catalyst. Reproduced with permission from ref (343). Copyright 2016, the authors.

Cross-metathesis with a saturated PO requires a catalyst to dehydrogenate both the polymer and a light alkane, forming two types of unsaturated chains, Figure 61a. Next, the alkenes undergo cross-metathesis, resulting in shortening of the PE chain. Finally, hydrogenation of the newly formed alkenes affords the saturated alkane products. In order to minimize self-metathesis of unsaturated PE chains, the process is generally conducted with a large excess of the light alkane (although this strategy makes undesired metathesis between light alkanes more likely). The initial cross-metathesis products can react further with light alkenes to form secondary cross-metathesis products with even shorter chain lengths. After multiple cross-metathesis reactions, PE can be converted to liquid hydrocarbons.

The tandem catalytic cross-alkane metathesis between PE and *n*-hexane was conducted using a dual catalyst system, Figure 61b.^343^ In a typical batch experiment, PE (*M*_w_ = 3,350 g/mol, *Đ* = 1.6, 120 mg) was dissolved in *n*-hexane (3 mL) at 150 °C under Ar. An Ir pincer catalyst (20 μmol) performed transfer dehydrogenation to TBE (40 μmol), while alkene metathesis was catalyzed by Re_2_O_7_/γ-Al_2_O_3_ (546 mg, 5 wt% Re or 57 μmol Re_2_O_7_). PE was fully converted after 3 d. The products were mostly liquid hydrocarbons in the diesel range, with carbon numbers from 7 to 40 (1.07 g), as well as a smaller amount of heavier wax (53 mg). The liquid yield was 9× higher than the initial mass of PE, thus ca. 90 % of the carbon in the liquid products originated in the *n*-hexane.

Petroleum ether (distillation fraction from 35 to 60 °C) was suggested to be more practical as a reagent in alkane metathesis due to its lower cost and wider availability, compared to *n*-hexane or *n*-octane. The degradation of an HDPE bottle (0.3 g, *M*_w_ = 109,000 g/mol, *Đ* = 6.8) was performed in petroleum ether at 175 °C, yielding 1.8 g alkanes as major products (carbon numbers from C_7_ to C_38_), as well as 2 mg PE wax. Here too, the mass of liquid products was much larger than the initial mass of HDPE, so the products mostly contained carbon derived from the light hydrocarbon solvent. The low recycling efficiency is one of the many challenges for the process economics. The rate of PE cross-metathesis is very low, and may be inhibited by the self-metathesis of light alkanes that are necessarily used in large excess. Nevertheless, the reaction was successful with various commercial PE grades, including HDPE, LDPE, LLDPE, and post-consumer PE waste (Table 7). Both the Ir pincer catalyst and the Re metathesis catalyst tolerated the presence of antioxidants 1010 and 168 (polyphenol- and phosphite-based additives, respectively), as well as zinc stearate.

**Table 7:** Products from the Cross-Metathesis of Various Grades of PE with *n*-Octane^a^

PE			oil yield (wt%)^c^	mass of soluble products (mg)^b^						solid products	
type	mass (mg)	10^4^*M*_w_ [*Đ*]		C_3–7_	C_9–13_	C_14–19_	C_20–25_	C_>25_	total	mass (mg)	*M*_w_ [*Đ*]
HDPE-2 pellet^d^	120	12.4 [3.9]	72	312	289	81	23	31	740	34	780 [1.3]
HDPE-3 pellet^d^	117	36.5 [13.0]	62	262	264	79	22	29	660	44	780 [1.2]
LDPE film^d^	120	9.2 [17.9]	86	281	252	54	15	22	620	17	4320 [4.0]
LLDPE pellet^d^^,^^e^	120	7.4 [3.0]	77	271	236	71	22	31	630	26	790 [1.3]
used PE bottle	122	10.9 [6.8]	67	314	283	77	21	29	720	40	860 [1.3]
used PE film	122	21.6 [12.7]	72	305	290	80	21	30	730	34	810 [1.2]

a Reaction conditions: *n*-octane (4.0 mL, except where noted), (*t*-Bu^2^PO-^*t*-Bu^POCOP)Ir(C_2_H_4_) (4.2 μmol), Re_2_O_7_/γ-Al_2_O_3_ (546 mg, 57 μmol Re_2_O_7_), 175 °C, 4 d.

b Products were dissolved in *n*-octane at room temperature.

c Yield of oil products when PE conversions were 100 %.

d Polymer contains antioxidant 1010, antioxidant 168, and zinc stearate, 1000 ppm each.

e *n*-Octane, 2.5 mL.

Adapted with permission from ref (343). Copyright 2016, the authors.

The selectivity of the transfer dehydrogenation catalyst was suggested to play an important role in the rate at which the PE chains decreased in length. The rate should be greatest when the C=C bond initially forms in the middle of the PE chain. The (POCOP)Ir complex, with its *bis*(phosphinite) ligand, is therefore more efficient for PE degradation than the (PCP)Ir catalyst, because the latter is more selective for terminal alkane dehydrogenation. Double bond migration via alkene isomerization is also catalyzed by the Ir complexes.

**Figure 62:**
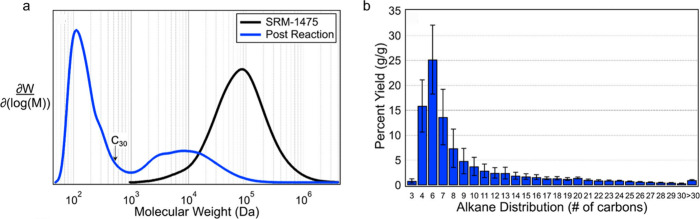
Product characterization from tandem dehydrogenation/alkene cross-metathesis of PE (130 mg, *M*_w_ = 5,200 g/mol, *Đ* = 3.0) with *n*-pentane (30 mL), catalyzed by Re_2_O_7_/γ-Al_2_O_3_ (500 mg) and PtSn/γ-Al_2_O_3_ (500 mg) at 200 °C: (a) molecular weight distributions for PE and post-reaction solids; and (b) analysis of light alkane products. Reproduced with permission from ref (345). Copyright 2021, American Chemical Society.

A fully heterogeneous catalyst system for PE upcycling via alkane cross-metathesis was developed. It consisted of a physical mixture in a 1:1 mass ratio (active phase + support) of PtSn/γ-Al_2_O_3_ (0.8 wt% Pt , 1.7 wt% Sn) and Re_2_O_7_/γ-Al_2_O_3_ (8 wt% Re).^345^ Alkane cross-metathesis of PE (*M*_w_ = 54,000 g/mol, *Đ* = 3.0) was conducted in *n*-pentane, and the extent of depolymerization was assessed by high-temperature GPC. After 15 h at 200 °C, the weight-averaged molecular weight of the residual PE had decreased by 73 % (not including contributions below 500 g/mol), Figure 62a. The light alkane product distribution is shown in Figure 62b. The total alkane yield in the liquid phase, including lighter hydrocarbons not detected by GPC, was 99 % (calculated based on an initial charge of 130 mg PE). However, the carbon balance was 126 % (also based on PE). A control reaction without PE (i.e., only *n*-pentane) gave no alkanes longer than C_13_, suggesting that longer hydrocarbons were derived from the polymer. However, an isotope-labeling study would be required to conclusively distinguish products of *n*-pentane self-metathesis from products of cross-metathesis between *n*-pentane and PE.

**Figure 63:**
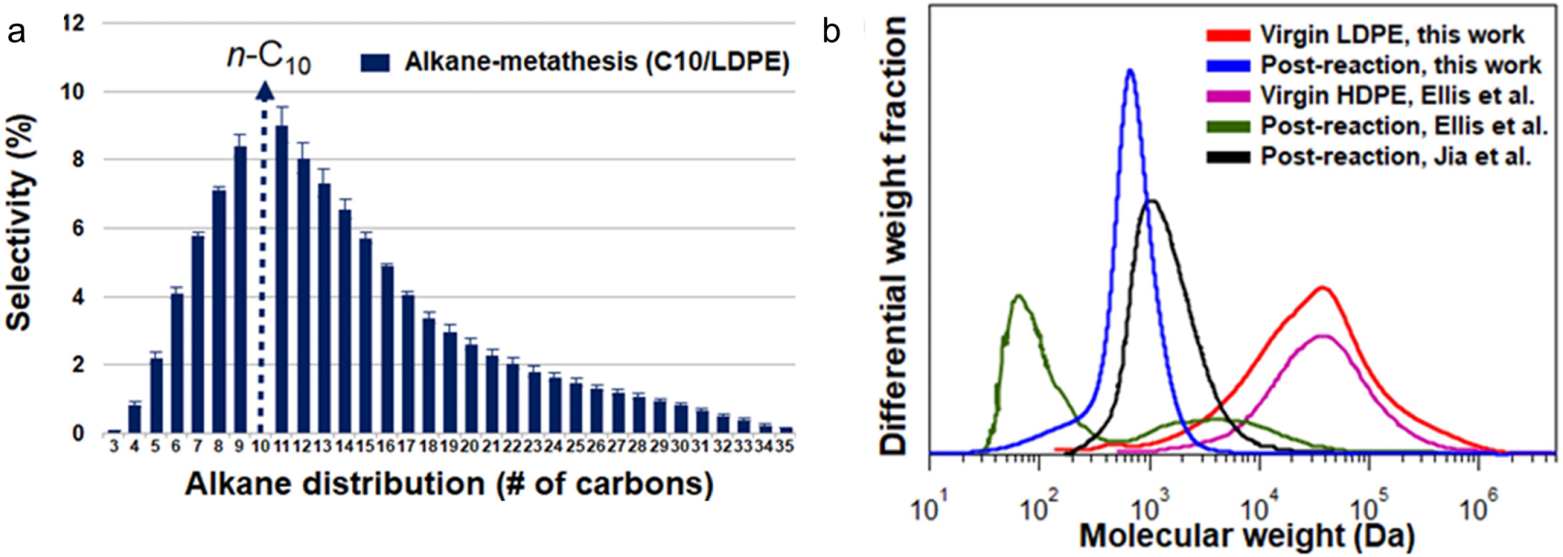
(a) Selectivity in the alkane cross-metathesis of LDPE with *n*-decane. Reaction conditions: LDPE (*M*_w_ = 75,000 g/mol, *M*_n_ = 9,100 g/mol, 1.55 g), *n*-C_10_H_22_/LDPE = 6.3 (g/g), Pt/γ-Al_2_O_3_ (1 wt% Pt, 500 mg), WO_x_/SiO_2_ (2 wt% W, 800 mg), zeolite 4A (1.0 g), 30 bar N_2_, 300 °C, 3 h. (b) Comparison of differential molecular weight distributions for the solid products, measured by high temperature GPC.^343,345^ Reproduced with permission from ref (346). Copyright 2022, Elsevier.

A physical mixture of Pt/γ-Al_2_O_3_ and WO_x_/SiO_2_ catalyzed the cross-alkane metathesis of LDPE with *n*-decane.^346^ Compared with the Re-based heterogeneous metathesis catalyst, the higher thermal stability of the W-based catalyst^393^ allowed higher temperature operation, increasing the dehydrogenation rate and yield in a shorter time. Heating LDPE with *n*-decane and the catalysts in a batch reactor for 3 h at 300 °C resulted in full PE conversion. The liquid product distribution shown in Figure 63a. The carbon balance was ca. 95 %, based on the total amounts of PE and *n*-decane. The conversion of *n*-decane (60 %) and the liquid product yield (350 %, based on LDPE) are low compared to a model reaction between *n*-decane and *n*-hexadecane (82 % conversion of *n*-decane and 448 % yield of liquid products based on *n*-hexadecane), although the selectivities are similar. The lower conversion of *n*-decane in the former case was attributed to reduced *n*-decane adsorption on the catalyst surface, due to the blocking of active sites by the molten polymer.

High temperature GPC of the solid showed a bimodal molecular weight distribution, Figure 63b, with one mode corresponding to wax centered at 700 g/mol (major component), and a second mode corresponding to intermediate chain-length alkanes at ca. 200 g/mol. The *M*_w_ and *M*_n_ values for the wax were 840 and 680 g/mol, respectively.

#### 4.3.3 Catalyst Regeneration

For dual catalyst systems used in tandem alkane metathesis, post-reaction catalyst separation and regeneration are problematic. Combinations of homogeneous and heterogeneous catalysts (e.g., an Ir pincer complex and Re_2_O_7_/γ-Al_2_O_3_)^343^ are particularly difficult to regenerate due to differences in their thermal stability and regeneration procedures. After one PE upcycling reaction, the recovered solid catalyst consisted of the Ir catalyst adsorbed on γ-Al_2_O_3_, as well as Re_2_O_7_/γ-Al_2_O_3_. The solid was washed with toluene, but it was unclear whether the Ir catalyst was removed in this step. Based on literature procedures,^356,357^ Re_2_O_7_/γ-Al_2_O_3_ should be regenerated by calcination in O_2_ at 550 °C. The washed solid was reactivated at high temperature (details of the regeneration procedure were not provided), and its alkene metathesis activity was evaluated with 1-octene (200 μL) dissolved in toluene (1 mL) at 175 °C. After 12 h, the 1-octene conversion was 97 %. Although this test demonstrated that regenerated Re_2_O_7_/γ-Al_2_O_3_ was active, the activity was not compared to that of fresh Re_2_O_7_/γ-Al_2_O_3_.

The dehydrogenation activity of the washed solid (presumably still containing some adsorbed Ir) was tested in the transfer dehydrogenation between cyclooctane and TBE. After 8 h at 200 °C, the washed catalyst gave 420 turnovers, compared to 940 turnovers for the fresh Ir catalyst adsorbed on γ-Al_2_O_3_. Thus, although the Ir component of the dual catalyst system can be recycled, it showed reduced activity. Separation of the two catalyst components to accommodate their distinct regeneration requirements was not attempted.

#### 4.3.4 Ethenolysis of Unsaturated Polyolefins

Light alkenes are desirable as depolymerization products because they can be repolymerized to give new materials that are indistinguishable from virgin polymer, with properties tailored specifically for the desired application.^17^ However, direct depolymerization to monomer is energetically very demanding for PE and PP, whose heats of polymerization are large (−105 kJ/monomer for PE, −96 kJ/monomer for PP).^394^ Although polymerization should be reversed above the ceiling temperature (*T*_c_, ca. 400 and 300 °C for PE and PP, respectively),^394^ catalytic pyrolysis of HDPE at 500 °C catalyzed by HY zeolite gave ethylene in only 0.62 wt% yield; the other main products were waxes.^223^ Cold plasma pyrolysis in the presence of HZSM-5 or sulfated zirconia produced just 13 wt% ethylene at 500 °C.^395^

An alternative to direct depolymerization is cross-metathesis of PE with ethylene (i.e., ethenolysis) combined with alkene isomerization to produce light alkene monomers at modest reaction temperatures (≤200 °C).^381^ Two groups independently reported the tandem catalytic conversion of PE to propylene by partial dehydrogenation of PE and repeated tandem ethenolysis/isomerization of the unsaturated polymer chains.^344,396^ Both homogeneous pincer Ir complexes and supported Pt nanoparticles were used to catalyze dehydrogenation, due to their high activities at moderate reaction temperatures (<200°C). The alkene yield can be enhanced by transfer dehydrogenation to a sacrificial H_2_ acceptor such as TBE, or to ethylene itself.

**Figure 64:**
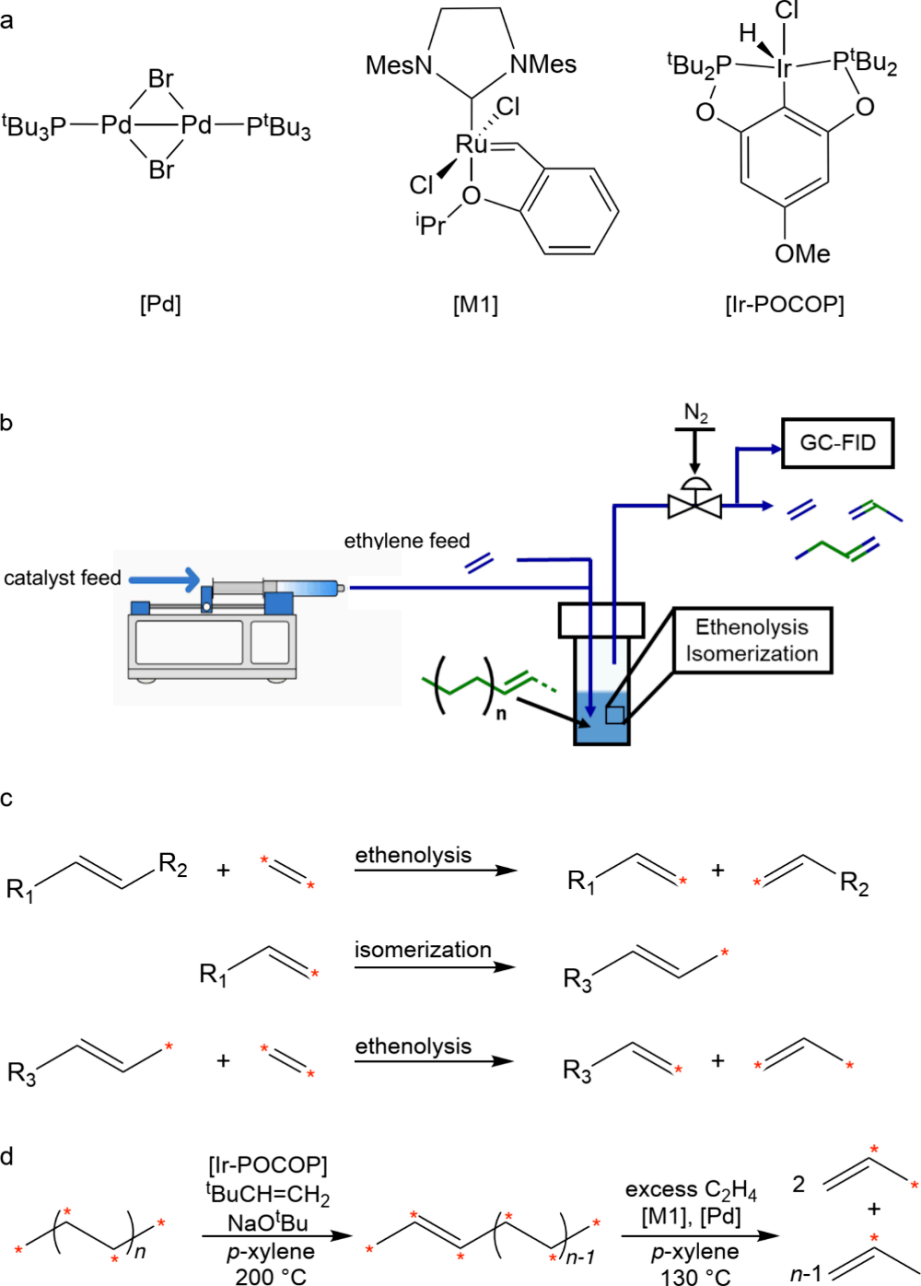
(a) Catalysts used in PE conversion to propylene by dehydrogenation and tandem ethenolysis/isomerization. Reproduced with permission from ref (396). Copyright 2022, AAAS. (b) Setup of the flow reactor for semi-continuous tandem ethenolysis/isomerization at atmospheric pressure. Reproduced with permission from ref (344). Copyright 2022, American Chemical Society. (c) Proposed pathway for forming doubly-^13^C-labeled propylene via tandem ethenolysis/isomerization of a hydrocarbon chain using doubly-^13^C-labeled ethylene. Reproduced with permission from ref (344). Copyright 2022, American Chemical Society. (d) Isotopic labeling of propylene made by dehydrogenation/ethenolysis/isomerization of labeled PE (99.9 % ^13^C) with unlabeled ethylene. Adapted with permission from ref (396). Copyright 2022, AAAS.

In one approach, PE dehydrogenation and ethenolysis/isomerization of the resulting unsaturated polymer were performed in separate batch reactors.^396^ In a typical experiment, unsaturated PE was obtained by dehydrogenation of HDPE (*M*_n_ = 26,100 g/mol, *Đ* = 3.3, 100 mg, 3.56 mmol monomer) using a homogeneous catalyst, Ir-(^tBu^POCOP)-HCl (9.8 mg, 15 μmol, 0.0042 equiv. relative to PE monomer units) in the presence of NaOtBu (1.6 mg, 16 μmol, 1.1 equiv. relative to Ir), *p*-xylene (3 mL) and TBE (0.02-0.40 equiv. relative to PE monomer units). After 12 h at 200 °C, the hot mixture was poured into acetone (100 mL) to precipitate the unsaturated polymer. With 0.4 equiv. TBE, the alkene yield (ratio of C=C bonds to total monomer units) was 3.5 %, with internal alkenes being strongly favored (97 %). The heterogeneous dehydrogenation catalysts Pt/Sn-γ-Al_2_O_3_ and Pt/Zn-SiO_2_ were less effective, even at 350 °C, yielding only 0.6 % C=C bonds. Batch ethenolysis/isomerization of the dehydrogenated polymer (dehydro-HDPE, 50 mg, *M*_n_ = 18,200 g/mol, 1.9 % alkene relative to monomer units) was conducted in *p*-xylene (1 mL) in a Parr reactor using metathesis catalyst **M1** (3.6 mol% relative to monomer units) and isomerization catalyst [Ir-POCOP] (2.2 mol%) (Figure 64a).^396^ The reactor was charged with ethylene (25 bar) and methane (25 bar, as an internal standard). After 16 h at 130 °C, the propylene yield was 87 %.

No direct information about catalyst deactivation can be obtained by operating in batch mode. However, different combinations of ethenolysis and isomerization catalysts gave propylene yields from 10 to 95 %, suggesting that catalyst stability and/or mutual compatibility effects are significant. A higher degree of polymer unsaturation was detrimental to the propylene yield because of the higher probability of forming dienes, which poison the metathesis catalyst. Compared with HDPE, LDPE gave a lower propylene yield for the same degree of unsaturation. Presumably, trisubstituted alkenes (which are thermodynamically favored in unsaturated LDPE) undergo very slow ethenolysis due to steric hindrance.

Continuous PE ethenolysis/isomerization was performed at atmospheric pressure in a continuous stirred tank reactor (CSTR) with a variety of homogeneous and heterogeneous catalysts and an ethylene feed (10 mL/min), Figure 64b.^344^ The PE and solid catalyst (where used) were loaded into the reactor at the start of the reaction. Homogeneous catalysts were fed continuously to the reactor by syringe pump to minimize deactivation. Volatile products and unreacted ethylene were continuously removed as gases, while non-volatile components remained in the reactor. The low pressure minimized ethylene self-metathesis and equilibrium limitations on the yield of propylene. With this setup, the kinetics of the tandem reaction and catalyst deactivation were readily monitored.

A bifunctional heterogeneous catalyst, CH_3_ReO_3_/Cl-Al_2_O_3_, proved most effective. The Re component performed alkene metathesis,^397^ while the acidic support was active for alkene isomerization.^398^ In a typical experiment, terminally-unsaturated PE (250 mg, *M*_n_ = 3,000 g/mol, *Đ* unspecified) was combined with CH_3_ReO_3_/Cl-Al_2_O_3_ (150 mg, 2.5 wt% Re, 4 wt% Cl) and heated to 100 °C in the presence of ethylene. After 5 h, PE was 50 % converted to light alkenes, with a propylene selectivity of 95 %. The catalyst deactivated gradually, and was inactive after 7 h.

According to the mechanism of tandem ethenolysis/isomerization, only the internal carbon of propylene originates from the polymer. Consequently, reaction with ^13^C_2_H_4_ should produce propylene with two ^13^C labels, Figure 64c. The origin of each carbon atom in propylene was investigated in the reaction of 1-octadecene with excess ^13^C_2_H_4_ (9 mol/mol *n*-C_18_H_36_) catalyzed by CH_3_ReO_3_/Cl-Al_2_O_3_ (10 mg, 2.5 wt% Re, 4 wt% Cl).^344^ After 3 h at 100 °C, the propylene analyzed by GC-MS was mostly (70 %) isotopomers with two ^13^C atoms (i.e., derived from ^13^C_2_H_4_). *In situ*^13^C MAS NMR spectroscopy confirmed that the labeled positions correspond to the terminal olefinic carbon (116–118 ppm) and the methyl carbon (19–21 ppm); the internal olefinic carbon (135 ppm) was mostly unlabeled (and therefore originated from 1-octadecene). These results confirm that propylene was the product of PE ethenolysis, rather than ethylene dimerization followed by cross-metathesis of butenes with ethylene.

A complementary isotope-labeling experiment was reported using ^13^C-labeled PE.^396^ Propylene arising from ethenolysis with unlabeled ethylene should contain a single ^13^C, Figure 64d. Lightly dehydrogenated and fully ^13^C-labeled PE (15.0 mg, 0.5 mmol, 1.2 % alkene) underwent ethenolysis catalyzed by **M1** (12.0 mg, 0.019 mmol) and isomerization catalyzed by Ir-POCOP (9.0 mg, 0.012 mmol) in a batch reactor with excess unlabelled ethylene (25 bar). After 16 h at 130 °C, the majority of the propylene (>70 %) was isotopically enriched with one ^13^C carbon.

#### 4.3.5 Metathesis Following Dehydrohalogenation

**Scheme 41:**
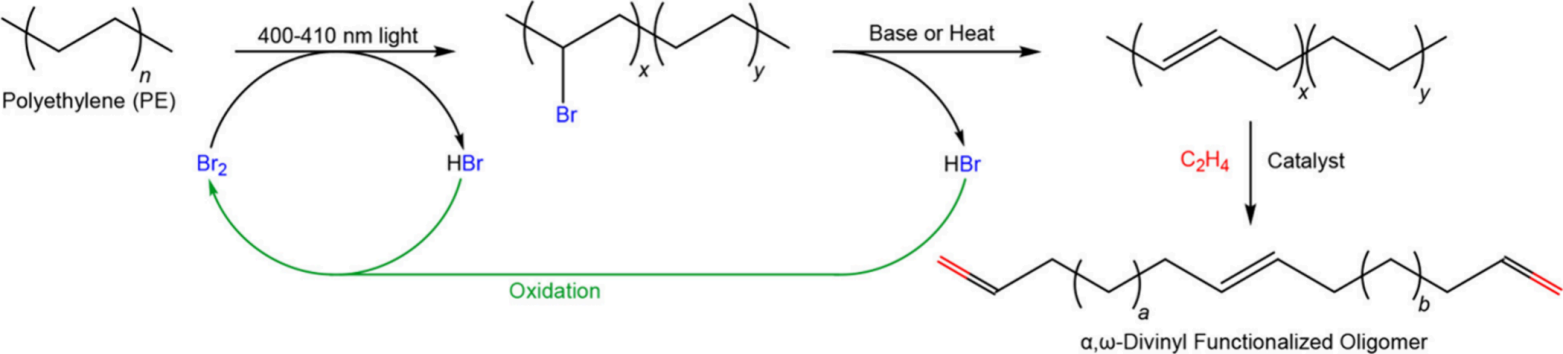
Strategy for Free Radical Bromination of PE, Followed by Dehydrobromination and Ethenolysis Reproduced with permission from ref (399). Copyright 2021, American Chemical Society.

An alternative strategy to generate unsaturated POs for ethenolysis involves free radical bromination, followed by dehydrobromination.^399^ The overall strategy is shown in Scheme 41. In a typical experiment, PE (5 g, 179 mmol monomer units) was dissolved in 200 mL benzene at 60 °C, Br_2_ was added, and the solution was irradiated with 400 nm light while stirring at reflux (80 °C) until no more HBr was released. Br was recovered in high yield (up to 91 %) as solid AgBr by bubbling through a 0.1 M AgNO_3_ solution (potentially useful for Br_2_ regeneration).

Brominated PE (BPE) showed new signals in the ^1^H NMR spectrum at 3.6–5.2 and 1.8 ppm, assigned to protons located α and β to the brominated carbons, respectively, while the ^13^C NMR spectrum contained characteristic signals at 59 and 39 ppm representing these α- and β-carbons, respectively. As expected, the C and H contents as measured by elemental analysis decreased due to the incorporation of Br, Table 8. The Br content of the polymer was controlled simply by varying the amount of added Br_2_. As the molar ratio of Br_2_ to ethylene monomer increased from 1:10 to 1:5, the Br content in the corresponding BPEs increased from 21 to 32 wt%. Under the mild bromination conditions, the structural integrity of the polymer backbone was largely preserved, however, PE chains with different initial molecular weights of *M*_n_ = 1,500 g/mol (*Đ* = 2.2) and *M*_n_ = 6,600 g/mol (*Đ* = 3.8) incorporated different amounts of bromine per chain.

**Table 8:** Bromination Efficiency in the Conversion of PE to Brominated Polyethylene (BPE)

PE			BPE						
entry^a^	10^3^ M_n_ (g/mol)	*Đ*	entry	Br_2_/C_2_H_4_^b^	10^3^ M_n_ (g/mol)	*Đ*	(C + H)^c^ (wt%)	efficiency^d^ (%)	isolated yield (%)
PE-1	1.5	2.2	BPE-1	1:5	4.4	2.1	68	88	83
			BPE-2	1:10	3.8	2.0	79	94	85
PE-2	6.6	3.8	BPE-3	1:5	15	4.2	72	77	86

a PE-1 was used to make BPE-1 and BPE-2; PE-2 was used to make BPE-3.

b Ratio of mol Br_2_ added per mol ethylene monomer.

c Determined by elemental analysis.

d Calculated as [(100 – [C + H, wt%])/expected Br, wt%] × 100 %. Expected Br content is based on the quantity of Br_2_ added.

Reproduced with permission from ref (399). Copyright 2021, American Chemical Society.

Base-catalyzed dehydrobromination of BPE gave unsaturated vinylene PE (VPE). Its Br content was not reported, but dehydrobromination was deemed complete by the disappearance of ^1^H signals at 3.6–5.2 ppm and ^13^C signals at 59 ppm. The appearance of ^1^H NMR peaks at 5.39 and 5.35 ppm, and ^13^C signals at 131 and 130 ppm, confirmed the presence of internal alkenes. Quantitative analysis indicated that the molar ratio C/vinylene groups in the VPEs was higher than the C/Br atomic ratio in the corresponding BPEs, although the two values were expected to be similar because each grafted Br should produce one C=C bond. The lower-than-expected vinylene content was attributed to cross-linking. More cross-linking occurred in a VPE derived from a BPE with a higher Br content.

Finally, ethenolysis with a commercial Ru metathesis catalyst (Grubbs Catalyst M202) at 100 °C shortened the VPEs into α,ω-divinyl-functionalized oligomers, Table 9. ^1^H and ^13^C NMR analysis confirmed the formation of vinyl groups via a pair of ^1^H NMR signals at 5.8 and 5.0 ppm, and a pair of ^13^C NMR signals at 140 and 114 ppm. The vinylene ^13^C NMR signals at 130 and 131 ppm were significantly attenuated. According to GPC, the divinyl products had lower molecular weights than the VPEs, confirming that ethylene cleaved the C=C bonds in the unsaturated polymer. The average number of carbon atoms per chain decreased from 108 in the initial PE to 48 and 61 for the products divinyl-1 and divinyl-2, respectively, generated from different BPEs. These carbon number values suggest one ethenolysis event, on average, per VPE chain.

**Table 9:** Characterization of α,ω-Divinyl-Functionalized Oligomers Obtained from Ethenolysis of Unsaturated Vinylene PE (VPE)^a^

entry	*M*_n_ (g/mol)	*Đ*	vinyl^b^ (%)	vinylene (%)	recovered^c^ C=C (%)	isolated yield^d^ (%)
divinyl-1	671	2.0	24	41	65	97
divinyl-2	854	2.1	46	12	58	92

a Reaction conditions: 50 mg polymer, 2.5 mol% catalyst, 2.7 bar C_2_H_4_, 1 h.

b Values are calculated relative to the quantity of vinylene groups initially present in the VPE. The quantification of vinylenes and vinyl groups was achieved by ^1^H NMR using an internal standard.

c Values were calculated as vinyl (% yield) + vinylene (% remaining).

d Calculated as recovered mass/[(100 % – % remaining vinylene in reaction mixture) × moles of starting vinylene × C_2_H_4_ molar mass (28 g/mol) + mass of starting VPE].

Adapted with permission from ref (399). Copyright 2021, American Chemical Society.

A preliminary technoeconomic assessment of this approach to chemical upcycling of PE to α,ω-divinyl-functionalized oligomers was performed. High recovery of both Br_2_ and solvent (ca. 99 %) and sale of the coproducts (KBr and *t*-BuOH) were revealed to be crucial factors in the economics of the proposed process. At the same time, recovering Br_2_ and solvent increased costs. The *t*-BuOK cost could be eliminated by using a thermal process to replace base-catalyzed dehydrobromination.

**Scheme 42:**
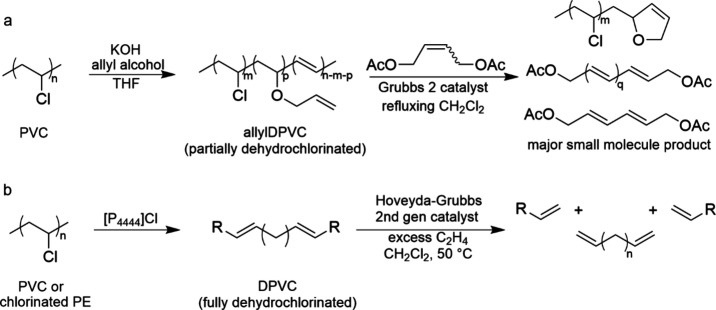
Two Strategies for Depolymerization of Chlorinated Polyolefins: (a) Partial Dehydrochlorination via Base-Catalyzed Elimination of HCl, Followed by Alkene Cross-Metathesis with *Z*-1,4-Diacetoxy-2-butene; and (b) Complete Dehydrochlorination in an Ionic Liquid, Followed by Ethenolysis (a) Adapted with permission from ref (400). Copyright 2023, Wiley-VCH GmbH. (b) Adapted with permission from ref (281). Copyright 2014, Wiley.

Unsaturated PE was the product of PVC dehydrohalogenation. The PVC molecular weigh, as measured by GPC was *M*_w_ = 65,000 g/mol, significantly higher than the supplier-provided value (*M*_w_ = 43,000 g/mol), due to the difference in behavior between PVC and PS reference standards. Molecular weights given below are all referenced to PS, introducing a systematic error (see section 1.3.2). Dehydrohalogenation was achieved in the presence of a base (Scheme 42).^400^ In a typical procedure, PVC (0.97 g) was dissolved in THF (20 mL) containing allyl alcohol (10 mL, see below), then variable amounts of KOH (as crushed pellets) were added with stirring at room temperature for 48 h. The amount of base determined the extent of HCl elimination. With excess KOH, the product was a fully dechlorinated polymer (i.e., polyacetylene), which was unreactive due to its insolubility in all solvents. However, when the amount of base was less than stoichiometric, a partially dehydrochlorinated polymer was formed.

Subsequent alkene cross-metathesis conducted in the presence of *Z*-1,4-diacetoxy-2-butene resulted in oligomers and other small molecules. The partially dehydrochlorinated polymer, which was soluble in THF, underwent alkene metathesis readily. After 16 h in the presence of the Grubbs-2 catalyst in refluxing dichloromethane (40 °C), the products were mostly PVC oligomers containing polyenes with a variety of conjugation lengths. A control experiment conducted in the absence of *Z*-1,4-diacetoxy-2-butene gave a molecular weight reduction similar to that observed in its presence. Therefore, ring-closing metathesis (RCM) was suggested to be the primary path for decreasing the molecular weight.

The addition of allyl alcohol during dehydrochlorination gave allyl ether side-chains grafted onto the polymer, facilitating intramolecular RCM. This reaction cleaved the chains, improving the access of the metathesis catalyst to internal double bonds and facilitating reduction in the polymer molecular weight. Dehydrochlorinated PVC without pendant allyl ether groups exhibited a more modest decrease in molecular weight (to *M*_w_ = 15,000 g/mol) upon cross-metathesis, while a substantial reduction in molecular weight (to *M*_w_ = 3,000 g/mol) was observed for dehydrochlorinated PVC with pendant allyl ether groups.

PVC dehydrochlorination was also promoted by various ionic liquids.^281^ In a typical reaction, the ionic liquid [P_4444_][Cl] (0.5 g) and unstabilized PVC powder (0.025 g, *M*_w_ = 48,000 g/mol, *M*_n_ and *Đ* unspecified, average particle size 0.004 ± 0.002 mm^3^) were combined under Ar and heated. After the reaction, water was added to precipitate the polymer and the mixture was titrated with hydroxide ion to evaluate the amount of HCl formed by dehydrochlorination. At 180 °C, the extent of dehydrochlorination was 88 % after 5 min, rising to 98 % after 60 min. The reaction is autocatalytic, since new C=C bonds formed by dehydrochlorination weaken adjacent C–Cl bonds, facilitating further dehydrochlorination. When PVC (4 mm^3^ fragments) was stabilized by Ca(stearate)_2_ (1500 ppt), dehydrochlorination was slower, resulting in only 59 % chlorine removal after 30 min. Near-full (96 %) dehydrochlorination of the stabilized PVC required 8 h. However, finely-ground stabilized PVC (0.1 mm^3^ fragments) underwent faster dehydrochlorination (74 % in 30 min). The authors surmised that particle size is more important than the presence of the stabilizer.

The dehydrochlorination activity of an ionic liquid depends on the Lewis basicity of its anion. Basic anions such as acetate and diethyl phosphate showed higher activities, lowering the dehydrochlorination onset temperature to below 150 °C. Anions with strong hydrogen-bond-accepting abilities also promoted dehydrochlorination. The reactivity of ionic liquids with identical cations (1-butyl-3-methylimidazolium) and variable anions followed the order: CH_3_COO > (C_2_H_5_O)_2_PO_2_ > Cl > Br > CH_3_C_6_H_4_SO_3_ > CF_3_COO ≈ CH_3_SO_3_ ≈ alkylOSO_3_ > N(CN)_2_ > N(CF_3_SO_2_)_3_ ≈ CF_3_SO_3_ ≈ HSO_4_. The cation identity also plays a role: increasing the size of the cation decreased the onset temperature, presumably by better solvating the PVC chains and thereby increasing their accessibility. Removal of Cl, as well as C=C bond formation, were confirmed by solid-state ^13^C NMR and IR spectroscopies. The dehydrochlorinated polymer was washed, dried, then subjected to ethenolysis, as shown in Scheme 42. When a PE chlorinated post-polymerization (0.012 g) was dehydrochlorinated then subjected to ethenolysis in dichloromethane (0.5 mL) with a Hoveyda-Grubbs 2nd-generation catalyst (5 mg) in a stainless steel autoclave under 4 bar ethylene at 50 °C for 24 h, the most abundant products were 1,5-hexadiene and 1,6-heptadiene. The combined yield of α,ω-dienes and isomerization products was 34 %.

#### 4.3.6 Polyethylene Self-Metathesis

Alkane metathesis of small molecules is catalyzed by [Ta-H]^+^ sites supported on sulfated alumina (SAO).^156^ The cationic [Ta] sites are more active in both hydrogenolysis and alkane metathesis than the corresponding neutral [Ta-H] sites present on silica. The self-metathesis of *n*-C_14_H_30_ (220 μL, 100 equiv.) was demonstrated for both [Ta-H]^+^/SAO and Ta-H/SiO_2_ (0.0085 mmol) at 150 °C. After 10 h, the cationic sites gave 3% tetradecane conversion, while the neutral catalyst gave only 0.6 % conversion. Even after 90 h, the conversion was still only ca. 10 % for the more active catalyst, which was fully deactivated after 96 h.

**Figure 65:**
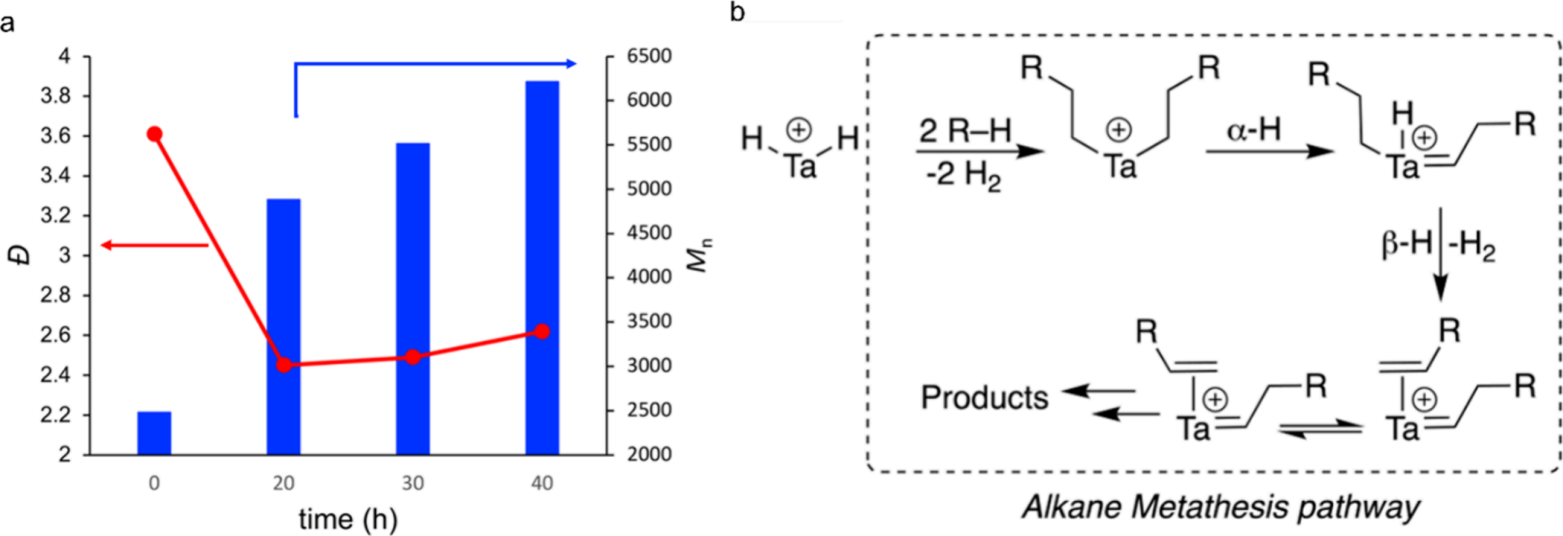
(a) Evolution of polymer molecular weight (blue) and dispersity (red) during PE self-metathesis catalyzed by [Ta-H]^+^ supported on sulfated alumina; and (b) proposed single-site mechanism. Adapted with permission from ref (156). Copyright 2023, American Chemical Society.

This strategy was extended to self-metathesis of a low molecular weight PE (340 mg, *M*_n_ = 2,500 g/mol, *Đ* = 3.6), using the cationic catalyst (17 μmol Ta). After 40 h at 150 °C, a higher molecular weight polymer (*M*_n_ = 6,200 g/mol, *Đ* = 2.3) as well as low molecular weight alkanes (8 mg, 2.3 wt% yield) were formed. All major products were linear alkanes. The dispersity (*Đ*) of the polymer initially decreased to 2.5, then remained constant as the reaction progressed, while the *M*_n_ value of the high molecular weight fraction increased monotonically, Figure 65a. According to GC-FID, the low molecular weight products had carbon numbers from 12 to 28; however, heavier alkanes may not have been soluble enough for recovery and/or volatile enough for detection.

The proposed single-site mechanism is shown in Figure 65b. Cationic [Ta-H]^+^ sites react with PE to form [TaR_2_]^+^ sites that undergo successive α-H and β-H eliminations to form [Ta(=CHR)(alkene)]^+^ intermediates. Higher and lower molecular weight alkenes are formed by alkene metathesis. The low yield of light alkane products and the long reaction times suggest that PO self-metathesis is not an efficient upcycling strategy.

#### 4.3.7 Polymer-to-Polymer Transformations

**Scheme 43:**
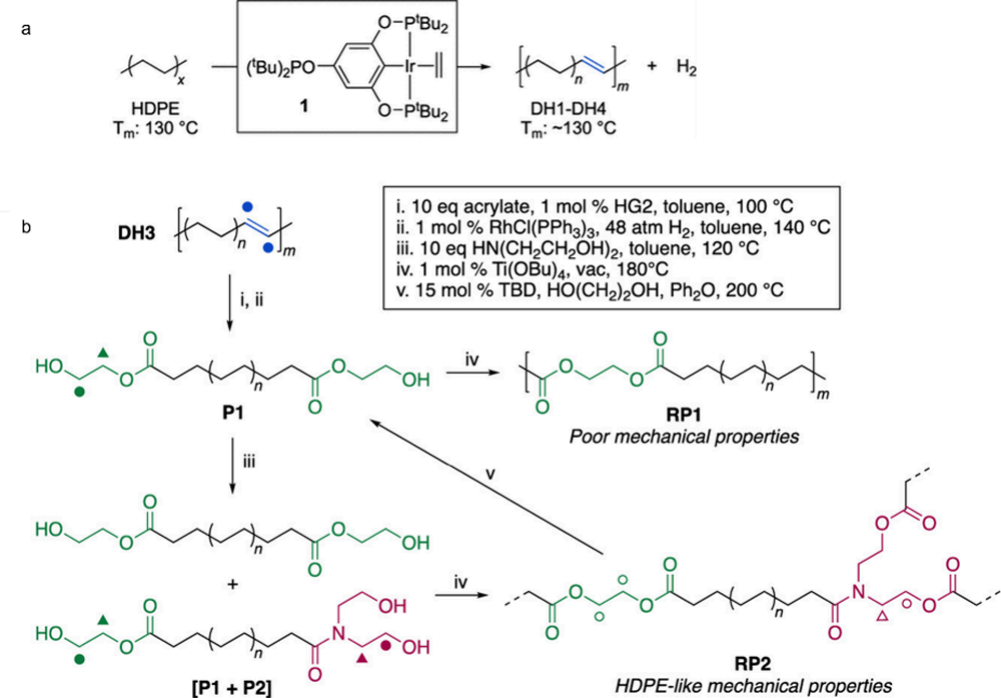
Strategy for Converting Post-Consumer Polyethylene to a Chemically Recyclable PE-Like Polymer: (a) Ir-Catalyzed Dehydrogenation; and (b) Reaction Conditions and Key Intermediates DH1–DH4: products from dehydrogenation of HDPE at various times (24–96 h); P1: telechelic macromonomer; RP1: repolymerization product; [P1 + P2]: macromonomers; RP2: products from repolymerization of [P1 + P2]. Adapted with permission from ref (401). Copyright 2022, American Chemical Society.

Instead of converting polymers into much smaller molecules, they can be used in polymer-to-polymer transformations. This approach converted a post-consumer HDPE into a material with mechanical properties comparable to PE but with enough hydrolysable ester linkages to facilitate its future depolymerization. The process consisted of five steps, each with a different catalyst and reaction conditions, as shown in Scheme 43.^401^ In order, the steps are: (1) dehydrogenation to give a slightly unsaturated polymer, (2) cross-metathesis to make telechelic macromonomers, (3) hydrogenation, (4) aminolysis, and finally, (5) repolymerization through transesterification.

Initial conversion of saturated HDPE to unsaturated HDPE installed the C=C bonds t be cleaved by alkene metathesis. The reaction was conducted at 200 °C under vacuum, catalyzed by a POCOP-Ir complex without a H_2_ acceptor, and driven by the removal of H_2_. The reaction required 96 h at 200 °C to generate a dehydrogenated HDPE with 0.8 mol% internal alkenes, as determined by ^1^H NMR. The molecular weight of the polymer did not change significantly during this step, indicating that C–C bond cleavage did not occur. Dehydrogenation of HDPE was tested and also worked well in the presence of iPP (10 wt%, *M*_w_ = 250,000 g/mol, *M*_n_ = 67,000 g/mol). iPP is an expected contaminant of post-consumer HDPE.

The partially dehydrogenated polymer (700 mg, 0.4 mmol) was subsequently subjected to cross-metathesis with excess 2-hydroxyethylacrylate (0.46 mL, 4 mmol) in the presence of Hoveyda-Grubbs’ catalyst HG_2_ (1 mol%, relative to mol monomer in the dehydrogenated polymer) for 6 h at 100 °C, producing telechelic macromonomers (P1) with an *M*_n_ 4× lower than the starting material (e.g., 4,300 vs. 18,000 g/mol). ^1^H NMR confirmed the disappearance of signals for internal alkenes (5.4-5.5 ppm) and the appearance of signals (3.88, 4.32, 5.91, and 7.05 ppm) for 2-hydroxylethylacrylate chain ends. Hydrogenation of the macromonomers at 140 °C under 48 bar H_2_ with Wilkinson’s catalyst, ClRh(PPh_3_)_3_, removed the remaining unsaturation, preventing cross-linking. The esters were partially converted to amides (8 mol%, relative to total esters) by reaction with a small amount of diethanolamine at 120 °C. The mixture of macromonomers ([P1 + P2]) was repolymerized in the presence of Ti(O^*n*^Bu)_4_ at 180 °C for 6 h under vacuum. The new polymer (RP2) had *M*_n_ = 18,200 g/mol and *M*_w_ = 80,300 g/mol, and showed some mechanical properties comparable to those of the starting HDPE.

The potential of RP2 depolymerization to P1 in closed-loop reprocessing was demonstrated using the organic base triazabicyclodecene as an organocatalyst in the presence of excess ethylene glycol and diphenyl ether at 200 °C, Scheme 43b (step v). This strategy is reminiscent of the design of PE-like materials with sparse ester or carbonate linkages to enhance their recyclability.^402,403^ In polymer-to-polymer transformations, the economic cost and environmental impact of multi-step processes may make using PE waste less attractive compared to *de novo* synthesis.

### 4.4 Conclusions and Perspectives

Waste POs (specifically, PE and PVC) can be upgraded to intermediate chain-length alkanes with potential value as fuels and chemical feedstocks by PO cross-metathesis with a short-chain alkane. Although the reactions require only mild to moderate temperatures, they tend to be very slow and to consume large amounts of the light alkane. Despite the mild conditions, catalyst deactivation is significant. Selectivity for cross-metathesis over self-metathesis in the presence of large amounts of the light alkane is challenging to achieve, although thermodynamics favor dehydrogenating the heavier alkane over the light alkane. Alkane self-metathesis of PE must also be suppressed because it results in polymer chains with even higher molecular weights. Therefore, achieving a balance between the reactivity of PE and that of the light alkane is crucial for efficient depolymerization.

Lighter alkanes can also act as solvents, softening or even dissolving PE and thereby reducing the resistance to heat and mass transfer, promoting cross-metathesis. Flow reactors with a light alkane recycle stream may allow for continuous removal of reaction products, allowing for lower pressure operation and reducing the self-metathesis of light alkanes.

In tandem catalytic processes such as the selective ethenolysis/isomerization of PE to propylene, a primary challenge lies in identifying catalyst combinations that are mutually compatible, and compatible with the required operating conditions. For example, many metathesis catalysts exhibit limited stability at the temperatures required to achieve sufficient alkane dehydrogenation (200–300 °C). More active dehydrogenation catalysts and/or more thermally stable metathesis catalysts will be helpful. The relative rates of alkene metathesis and isomerization influence the rate of C–C bond scission and product selectivity. For example, the rate of propylene formation by PE ethenolysis is influenced by the relative rates of ethenolysis and olefin isomerization.^381,382^ Multistep processes allow for catalyst compartmentalization and individual tailoring of reaction conditions but add considerably (and, probably, prohibitively) to the process cost, while sacrificing the benefit of coupling endothermic and exothermic steps in a single-pot process.

An additional challenge is the recovery and regeneration of catalyst mixtures, particularly those based on expensive metals and/or elaborate catalyst architectures (ligands as well as pore structures). Most organometallic-based catalysts are not readily regenerated. Moreover, many of the supported transition metal hydrides and homogeneous/heterogeneous alkene metathesis catalysts are extremely sensitive to polar impurities (moisture, oxygen, H_2_S, etc.). Purification is routine in the chemical industry for small molecule reactants, but can be complex and costly in the case of waste plastics. At this time, it remains unclear how many of these catalysts would perform with realistic post-consumer plastic waste.

## 5 Catalytic Oxidation of Polyolefins

### 5.1 Introduction to Oxidation of Polyolefins

Oxidation, including both thermal and photodriven processes, is the most common degradation pathway for POs in the natural environment, where complete oxidative degradation usually takes many years.^404^ The ultimate products are CO_2_ and H_2_O (mineralization), which is not considered to be upcycling. For this reason, papers focusing on complete oxidation are not included in this review.

**Scheme 44:**
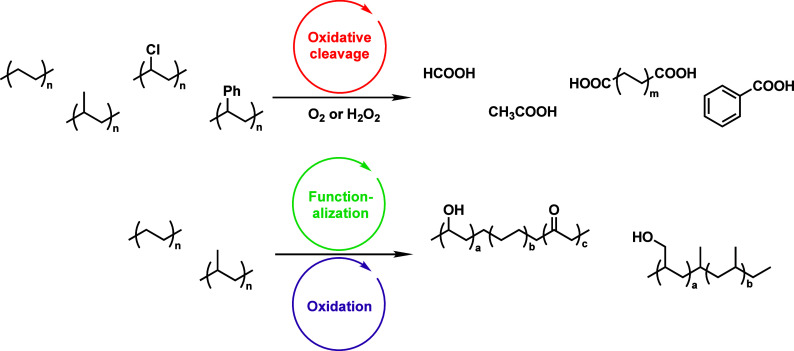
Two Strategies for Oxidative Upcycling of Polyolefins: Oxidative Cleavage to Small Molecule Oxygenates, and Polyolefin Functionalization without Chain Cleavage

Oxidative upcycling of POs can occur with or without chain cleavage (Scheme 44), depending on the mechanism. In oxidative cleavage, the PO reacts with an oxidant such as O_2_ or H_2_O_2_. Alkyl hydroperoxides are metastable intermediates, commonly observed and/or invoked in such processes. Oxidative C–C bond cleavage lowers the polymer molecular weight, producing shorter chains and eventually small molecule oxygenates. In contrast, C–H bond functionalization can occur without C–C bond cleavage. A variety of functional groups installed in this step can be converted to oxygen-containing functional groups in a subsequent step. Although the intended purpose is not usually recycling or upcycling of post-consumer plastic, and there are few examples involving POs to-date, the potential of this strategy for valorizing waste plastic led us to include a few such studies here.

#### 5.1.1 Mechanisms of Oxidative Cleavage

The oxidants most commonly used in the direct oxidation of POs are O_2_ and H_2_O_2_. When O_2_ (or air) is employed, the process is called autoxidation. In Fenton-type processes, H_2_O_2_ is the oxidant. In both processes, several mechanistic steps involve radicals. Nevertheless, there are some important differences between them, as discussed below.

##### 5.1.1.1 Mechanism of Thermal Catalytic Autoxidation

**Scheme 45:**
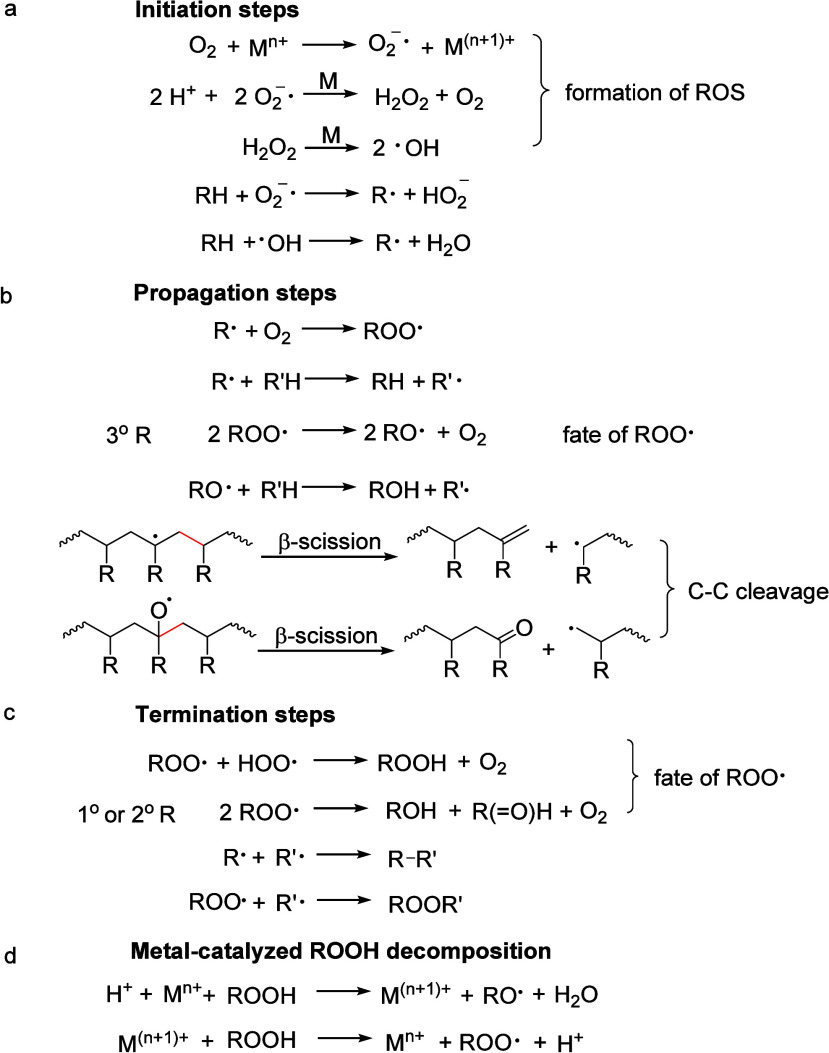
Proposed Radical Chain Mechanism for Thermal Catalytic Oxidation of Polyolefins: (a) Initiation, Starting with the Formation of Reactive Oxygen Species (ROS); (b) Propagation, Including β-Scission Resulting in Polymer Chain Cleavage; (c) Termination, Showing Several Possible Fates of the Alkyperoxy Radical ROO^•^; and (d) Peroxide Decomposition

Radical chain mechanisms for catalytic autoxidation of POs have been described in many reviews,^405,406^ so only a brief summary will be presented here. The general mechanism is depicted in Scheme 45. Note that the terms chain mechanism, chain reaction, and chain transfer (all referring to reaction steps) are completely distinct from the use of the term polymer chain (referring to the physical structure).

Initiation of autoxidation involves creating an alkyl radical (R^•^) on a polymer chain by hydrogen atom transfer (in synthetic chemistry, this is often abbreviated HAT) to a reactive oxygen species (ROS), such as the superoxide radical anion (O_2_^–•^) or the hydroxyl radical (HO^•^). Both radicals can be formed from O_2_ in the presence of a redox-active metal.^407−409^ In the autoxidation propagation steps, R^•^ is trapped by O_2_ to form an alkylperoxy radical (ROO^•^) located on the polymer chain.

The next step is usually described as H atom abstraction by ROO^•^ from another C–H bond in the polymer chain, to give an alkyl hydroperoxide (ROOH) and a new R^•^ site. However, this reaction is thermodynamically disfavored,^410^ unless an allylic or benzylic C–H bond is involved. ROO^•^ can also react with another peroxy radical (ROO^•^ or HOO^•^) to give alcohols, aldehydes, alkoxy radicals (RO^•^) or ROOH. These oxygenates are often further oxidized to carboxylic acids, esters, and even CO_2_. The newly-formed radical RO^•^ can abstract H atoms from other C–H bonds, propagating the oxidation of the polymer chain in a chain reaction. Alternatively, RO^•^ can undergo β-scission to form alkenes, aldehydes or ketones and a new alkyl radical. This scission also results in polymer chain cleavage. Metal-catalyzed decomposition of ROOH and ROOR′ can reinitiate the chain reaction.

##### 5.1.1.2 Mechanism of Photocatalytic Autoxidation

Many of the steps in the *photocatalytic* autoxidation of POs are the same as those in Scheme 45, but a few are unique to the photocatalytic process. PS is unique among the major POs in having an intrinsic UV chromophore (the phenyl ring), making it particularly well-suited to photocatalytic oxidation. Defect-free POs such as PE and PP have no intrinsic UV chromophores, but a light-absorbing catalyst can still accelerate the formation of ROS. In addition, ROOH sites on the polymer chain undergo spontaneous UV-induced O–O bond scission to give RO^•^ and HO^•^, even in the absence of a catalyst.^405^

**Scheme 46:**
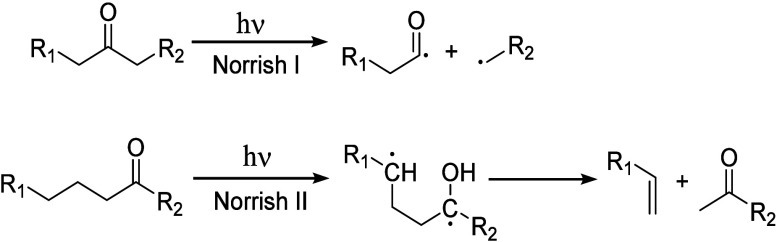
Norrish-Type Photochemical Reactions, Shown in the Context of Polyolefin Photooxidation Where a Prior Reaction Has Installed a Ketone or Aldehyde on the Polymer Backbone

Norrish reactions are also unique to photooxidation. They occur when UV absorption by ketones (present in PE and PP as defects, or as products of initial polymer oxidation) triggers C–C bond cleavage (Scheme 46).^411^

##### 5.1.1.3 Fenton Mechanism

**Scheme 47:**
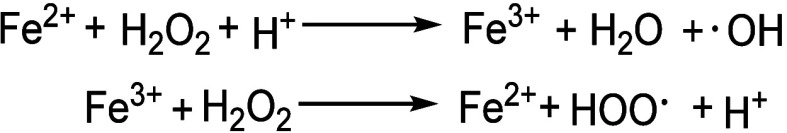
Fe-Catalyzed Formation of HO^•^ and HOO^•^ Radicals via the Fenton Reaction^412^

The Fenton reaction, discovered in 1894, has been widely investigated for wastewater treatment, due to its effectiveness in mineralizing organic pollutants.^412^ The oxidation of soluble Fe^2+^ by H_2_O_2_ generates HO^•^ and Fe^3+^, Scheme 47. Fe^3+^ is reduced by H_2_O_2_, regenerating Fe^2+^ and making HOO^•^. Since the second step is generally slower than the first, Fe^3+^ is usually the most abundant state of the catalyst during the reaction. The Fenton process has been applied in PE upcycling to carboxylic acids (see section 5.4). The roles of the HO^•^ and HOO^•^ radicals are presumed to be the same as in Scheme 45.

#### 5.1.2 Mechanism of Polyolefin Oxidation without Chain Cleavage

Oxidative C–H bond functionalization in POs without chain cleavage can be achieved in a two-step process.^413^ Just as for small molecule hydrocarbons, the C–H bond in a PO can be replaced by a C–B or C–Al bond, mediated by a transition metal catalyst. The resulting functionalized PO reacts with O_2_ or H_2_O_2_ to produce an OH-functionalized polymer. Alternatively, organic oxidants such as *meta*-chloroperoxybenzoic acid (^m^CPBA) can oxidize a metal catalyst which then oxidizes the PO, converting C–H bonds to C–O and/or C=O bonds. Specific examples of C–H functionalization in POs are discussed in section 5.5.

### 5.2 Thermal Catalytic Oxidation of Polyolefins

This section, organized by PO type, describes methods for oxidizing various types of POs and gives examples of their outcomes. In early research, much of the interest was in identifying functional groups generated during polymer oxidation, rather than the extent of conversion or the value of the products. Papers seldom reported quantitative analysis. Nevertheless, insights from these investigations remain relevant to contemporary research goals related to polymer upcycling. In many cases, the methods are suitable for PO mixtures, eliminating the need for sorting and separation in the recycling of plastic waste.

#### 5.2.1 Catalytic Oxidation of Polyethylene

The first catalytic oxidation of a PO was reported in 1960,^414^ only a few years after PE started to be widely used commercially. Inspired by observations on the oxidation of alkanes,^415^ and motivated by the deterioration of PE packaging materials caused by contact with metal-containing pigments including Fe, Cr, Mn and Co compounds,^416^ the metal-catalyzed oxidation of PE was explored under high pressure O_2_. For example, PE (25 g) was suspended in an aqueous solution of the catalyst (KMnO_4_ or Co(naphthenate)_2_, ca. 2 wt% metal relative to PE), for 8 h under 70 bar O_2_ at 70 °C. The modest reaction temperature suppressed undesired polymer crosslinking.

Since the products proved difficult to isolate, IR spectroscopy was used to identify functional groups. The recovered solid was a partly oxidized PE containing carbonyls, hydroperoxides and C=C bonds. The aqueous phase contained polyols with 6 to 8 carbons, in 5 wt% yield. Other organic compounds, including acids, esters, and alcohols, were extracted from the aqueous solution using diethyl ether. A wax with carbonyl groups and a softening point of 90 °C, soluble in methanol, was recovered in 3 wt% yield. Despite the low yields, the reaction was proposed as an interesting way to make valuable chemicals from PE waste, showing considerable foresight more than 60 years ago. However, the high pressure created a safety hazard which limited further use of the method.

**Scheme 48:**
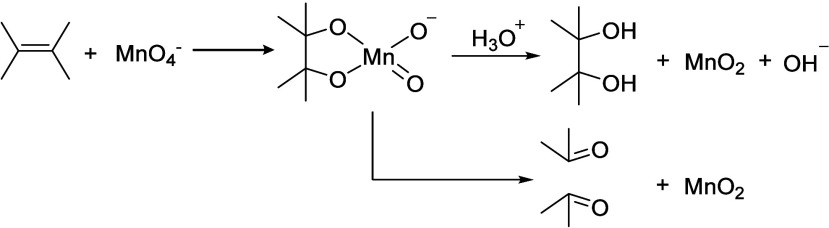
Two Possible Outcomes of the Stoichiometric Oxidation of an Alkene by Permanganate

Possible mechanisms were discussed at length.^414^ In the absence of a catalyst, the oxidation products included a large number of alkyl hydroperoxides, but the amount decreased significantly in the presence of a metal. KMnO_4_ and Co(naphthenate)_2_ were the most effective “catalysts” for PE autooxidation, as judged by the abundance of carbonyl groups in the products. The main role of the metal was presumed to be catalyzing the decomposition of alkyl hydroperoxides, to generate alkoxy and hydroxyl radicals that initiated new chain reactions (Scheme 45d). The formation of polyols led to the idea that MnO_4_^–^ reacts with C=C bonds (formed during PE autoxidation) to give Mn(V) diolates, which hydrolyze to give diols (Scheme 48). MnO_4_^–^ is also known to cleave alkenes to form ketones and/or aldehydes. In both kinds of reaction, MnO_4_^-^ is usually a stoichiometric oxidant rather than a catalyst. However, given the low metal concentration, such stoichiometric reactions should not be major contributors to the overall reaction.

The 1970s saw the beginning of the use of PE as an insulating material for Cu-based conductors, mainly Cu wiring. To understand the slow degradation of PE insulation, researchers investigated the effect of metallic Cu^417−419^ and Cu(stearate)_2_^419,420^ on PE oxidation in air or O_2_. The catalytically active oxidation state was believed to be Cu^2+^, regardless of the initial form of the catalyst. Metal ions were proposed to decompose peroxides via a radical pathway,^420^ similar to the route shown in Scheme 45d. These early studies focused on identifying functional groups (C=O and C=C bonds) present in the products using IR. They did not report yields or molecular weights for the hydrocarbon products.

**Figure 66:**
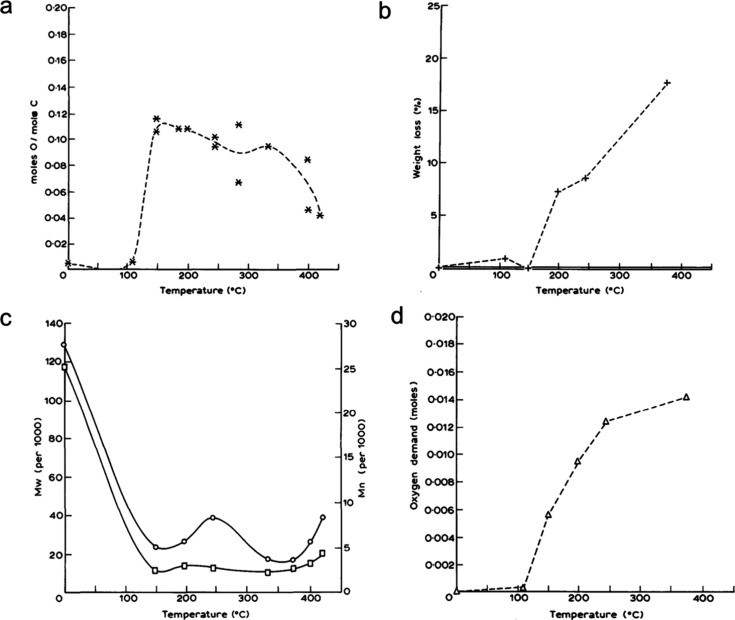
Quantitative analysis of LLDPE after Co(acac)_2_-catalyzed oxidation for 3 h at various temperatures: (a) oxygen content of the solid residue; (b) weight loss relative to the starting amount of polymer; (c) number- and weight-averaged molecular weights of the solid residue; and (d) “oxygen demand”. Adapted with permission from ref (421). Copyright 1993, Elsevier.

The oxidation of linear low-density polyethylene (LLDPE, *M*_n_ = 35,000 g/mol, *M*_w_ = 128,700 g/mol) catalyzed by Co(acac)_2_ was investigated quantitatively.^421^ PE was pressed into a thin film and placed in an acetone solution of Co(acac)_2_ (4 wt%) with dicumyl peroxide (0.4 wt%) as initiator. After evaporation of the acetone, the mixture was heated in air in a bomb reactor for 3 h. Although the mechanism was barely investigated, this study reported the first quantitative analysis of PO oxidation, including gas products, as a function of temperature. The oxygen content and weight loss of the solid residue were quantified, and the molecular weight was measured. After 3 h, the oxygen content was highest and the weight loss was lowest at 150 °C (Figure 66a,b). In addition, the molecular weight was a minimum for the solid residue, *M*_n_ = 2,500 g/mol and *M*_w_ = 24,000 g/mol (Figure 66c). At lower reaction temperatures, the extent of oxidation was less, and little chain scission occurred. At higher reaction temperatures, the formation of volatile small molecules (such as H_2_O, CO, and CO_2_) was extensive. However, the average chain length hardly changed, presumably because cross-linking caused the molecular weight of some remaining chains to increase. The “oxygen demand” was calculated as the sum of the final oxygen contents in both the gas and solid phase products. It increased considerably with temperature (Figure 66d), consistent with the above analysis.

**Figure 67:**
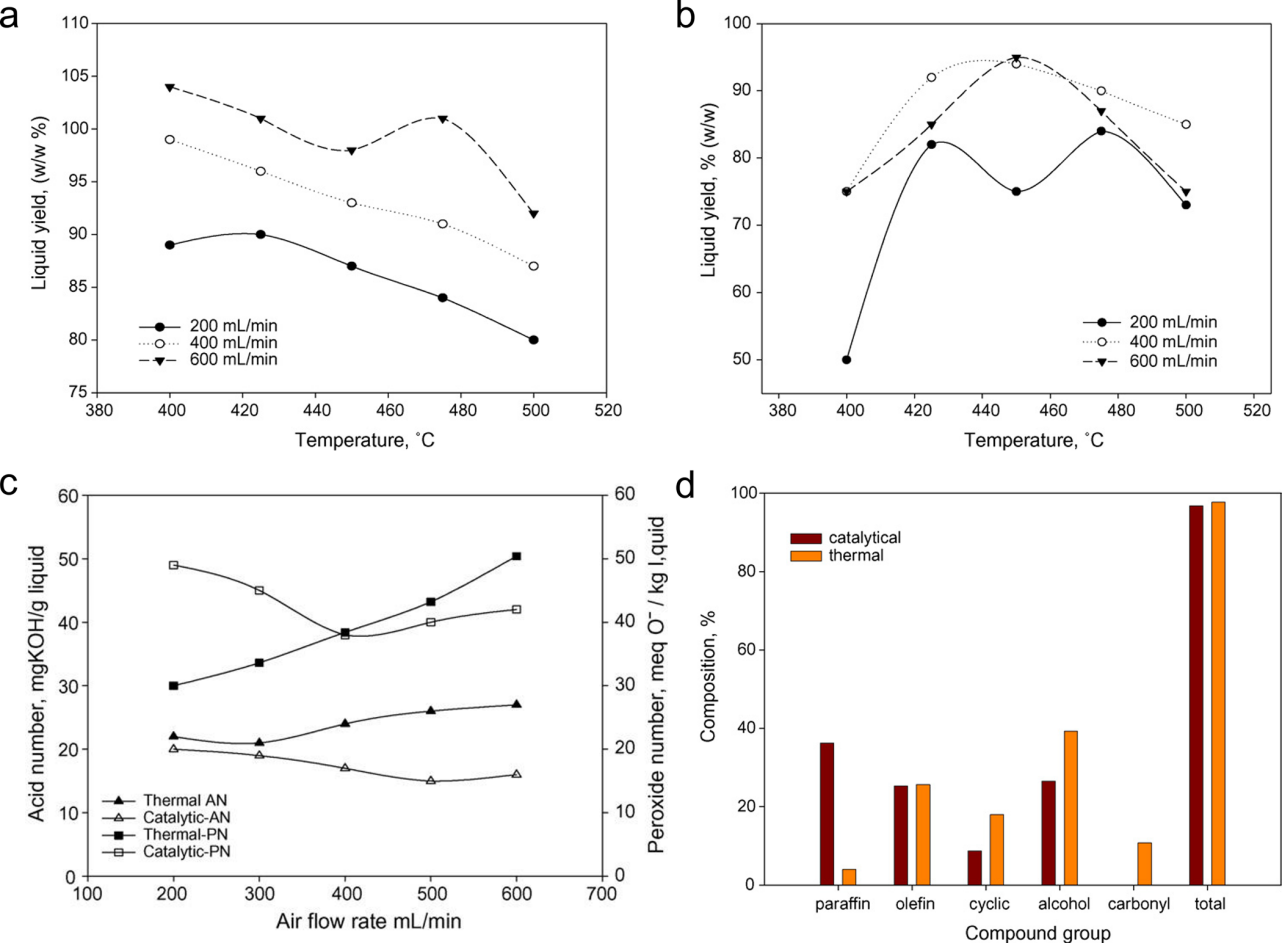
Liquid yields from the oxidation of LDPE at various temperatures and air flow rates: (a) without a catalyst; (b) in the presence of a MoO_3_/SiO_2_ catalyst; (c) AN and PN of liquid products formed at 400 °C with and without the catalyst, as a function of air flow rate; and (d) functional group distribution with and without the catalyst at 400 °C, in flowing air (600 mL/min). Adapted with permission from ref (422). Copyright 2006, Elsevier.

Thermal oxidation of LDPE was compared with and without a catalyst, MoO_3_/SiO_2_ (5 wt% Mo),^422^ by heating the polymer in flowing air for 20 min at 400 °C. Without the catalyst, the yield of liquid products was high (≥90%), but decreased due to the formation of gases (including CO and CO_2_) when the temperature increased up to 500 °C (Figure 67**a**). The liquid yield sometimes exceeded 100 %, due to the incorporation of oxygen. The degree of oxidation was judged by measuring the acid number (AN) (i.e., the mass of KOH, in mg, required to neutralize 1 g products), and the peroxide number (PN) (i.e., mol peroxide oxygen per kg products, determined by reaction with iodide, where 1 mequiv. = 0.5 mmol peroxide because each peroxide contains two peroxo oxygens). The AN and PN values of the products increased with the air flow rate (Figure 67c).

The products of uncatalyzed oxidation were predominantly alcohols and carbonyl compounds (Figure 67d). Catalytic oxidation gave similar liquid yields, approx. 80–95 % at 420–480 °C (Figure 67b), but yielded primarily alkanes and alkenes instead (Figure 67d). In addition, the AN and PN values decreased as the air flow rate increased. The authors suggested that alcohols and carbonyl compounds are more likely to be oxidized further to CO or CO_2_ in the presence of the catalyst, so that only alkanes and alkenes remained in the liquid phase. This explanation also accounted for the lower liquid yields in the catalytic process relative to the non-catalytic process. Although only the liquid products were analyzed and the mechanism was speculative, this study revealed key distinctions between catalytic and non-catalytic oxidation.

**Figure 68:**
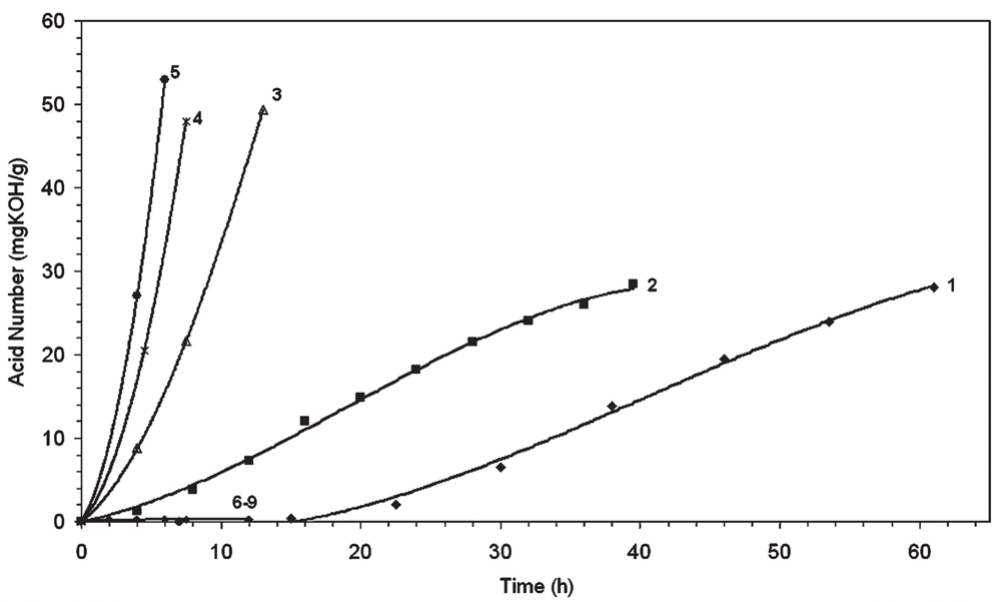
Effect of metal acetylacetonate catalysts on PE oxidation, as judged by the acid numbers (AN) of the hydrocarbon products: (1) without catalyst or dicumyl peroxide (DCP) initiator; (2) without catalyst but with DCP; (3–9) with DCP and a transition metal acetylacetonate, containing (3) Fe(III), (4) Mn(II) and (5) Co(II), (6) V(III), (7) Cu(II), (8) Ni(II), or (9) Al(III). Reproduced with permission from ref (423). Copyright 2010 Wiley.

The ability of various transition metal acetylacetonates to catalyze the aerobic oxidation of HDPE was investigated.^423^ Powdered PE was mixed with the catalyst (0.15 wt%) and dicumyl peroxide (initiator, 1 wt%) in acetone and heated at ca. 110 °C for 7.5 h in flowing air (15 L/h). The oxidized PE was characterized only by functional group analysis; no information was provided on its molecular weight or yield. The organic products were dark-colored due to the presence of trace metal residues. Based on the product AN, catalysts containing Co(II), Mn(II), and Fe(III) significantly accelerated the reaction (Figure 68). IR spectroscopy confirmed the presence of various types of carbonyl groups, including ketones, carboxylic acids, and esters.

The aerobic oxidation of HDPE (*M*_n_ = 38,800 g/mol, *M*_w_ = 522,200 g/mol) was also investigated in water, using the same radical initiator and metal catalysts.^424^ An autoclave was charged with PE powder, a metal acetylacetonate (0.5 wt%), dicumyl peroxide (1 wt%) and water, and the mixture was heated at 120 °C under 8 bar O_2_ for 5 h. Surfactants such as Rokanol L10 (2 wt%) were added to aid the dispersion of PE in water. After the reaction, the molecular weight decreased by about 40× (*M*_n_ = 1,100 g/mol, *M*_w_ = 11,500 g/mol). The oxidized PE was colorless, unlike the dark-colored products of the solid-state reactions,^423^ because the catalysts remained dissolved in the aqueous phase. Reusing the water containing the dissolved catalyst on fresh PE led to products with much lower ANs. No yields were reported in this study.

PE oxidation catalyzed by Ru/TiO_2_ (1.6 wt% Ru) was conducted at 160 °C under 15 bar air for 24 h.^425^ Five kinds of PE were tested (Table 10). The yields of oil products exceeded 80 wt%, most of the remainder being solid residue. Only traces of volatiles were formed. According to NMR and IR analyses, the major compounds in the oils were dicarboxylic acids, with traces of esters. The recycled catalyst (recovered by washing with DCM and EtOH, filtering and drying) was even more effective than the fresh catalyst: the molecular weight of the oil decreased with each successive reuse. The effect was attributed to increasing hydrophobicity of the catalyst, as judged by its water contact angle. The accumulation of organic residue on the catalyst was suggested to enhance contact between the catalyst and molten PE.

**Table 10:** Results of the Oxidation of Various PEs Catalyzed by Ru/TiO_2_ (1.6 wt% Ru), After 24 h in Air

form	polymer		liquid products		
	*M*_w_ (g/mol)	*M*_n_ (g/mol)	yield (wt%)	*M*_w_ (g/mol)	*M*_n_ (g/mol)
PE pellets	2260	738	85	1290	279
LDPE powder	64,600	19,500	87	1650	150
HDPE powder	107,000	28,800	96	609	467
Pipettes	117,000	25,200	81	1060	109
Ziploc bag	126,000	29,300	83	1410	216

#### 5.2.2 Catalytic Oxidation of Polypropylene

Since the requirements for catalytic oxidation of PP are similar to those for PE, it is not surprising that Co and Mn complexes are also commonly reported as catalysts in PP oxidation. Importantly, the PP tacticity (i.e., isotactic, syndiotactic, atactic) has an influence on the rate of oxidation, although many upcycling papers fail to specify the type or degree of polymer stereoregularity.

Various organic solutions containing Co(II) (10^–3^ to 10^–1^ mol/L) were used to catalyze the oxidation of iPP at 80 °C for 5 h while stirring vigorously in flowing O_2_ (100 mL/min).^426^ Oxidation was much faster in acidic solvents such as propionic and acetic acids, compared to non-acidic solvents such as chlorobenzene and acetophenone. The effect was ascribed to the longer lifetime of the active form of the catalyst in the acidic solvents, although the nature of this active form was not specified. After 2 h, the polymer molecular weight (probably *M*_w_, although not specified as such) had decreased dramatically from 235,000 to 10,500 g/mol. CO_2_ and a small amount of CO were observed. The ratio of the CO_2_ formation rate to the O_2_ uptake rate increased gradually until it reached about 1:5 after 200 min. IR spectra of the insoluble iPP residue indicated the presence of carbonyl groups, signifying partial oxidation. Other products were acid-soluble compounds with molecular weights ranging from 250 to 730 g/mol, and C/O ratios of 3.0–5.4.

Although aPP is more soluble in acetic acid, its oxidation was much slower than that of iPP. The authors proposed that because iPP has tertiary hydrogens on the same side of the polymer backbone, they experience less steric hindrance during autoxidation chain propagation. Addition of NaBr as a cocatalyst (equimolar with Co(OAc)_2_) accelerated the rate of oxidation of aPP. The enhancement was attributed to formation of Br^•^, which abstracts H^•^ from a C–H bond in a chain-propagating step.^427^

An aerogel of MnO_x_ was reported to catalyze the oxidation of PP (tacticity not specified) to acetic acid in O_2_-enriched supercritical (sc) CO_2_ at 135 °C.^428^ Without sc-CO_2_, some of the products were solid (i.e., partially oxidized PP). When sc-CO_2_ was present, all polymer was converted to liquids, mostly acetic acid accompanied by smaller amounts of formic acid, propionic acid, and acetone. Sc-CO_2_ was proposed to accelerate the reaction by plasticizing the PP granules, thereby enhancing their contact with the solid catalyst. The mass of products was larger than the initial mass of PP due to oxygen incorporation. Notably, some CO_2_ was lost in the process (determined by comparing the volume of CO_2_ present before and after reaction). Two explanations were proposed: either CO_2_ remained dissolved in the liquid products, or it participated in carboxylation reactions. The latter seems more plausible due to the low solubility of CO_2_ in acetic acid at room temperature and atmospheric pressure.

#### 5.2.3 Catalytic Oxidation of Other Polyolefins and Polyolefin Mixtures

Various types of POs, including LDPE, HDPE, PP and PS (tacticities not specified), were oxidized to carboxylic acids at 170 °C under an atmosphere consisting of NO (2.75 bar), O_2_ (6.9 bar), and N_2_ (31.7 bar).^429,430^ After 16 h, the PE oxidation products were mostly small dicarboxylic acids, including succinic, glutaric, adipic, and pimelic acids. The main product of PP oxidation was acetic acid, while PS gave mostly benzoic acid and nitro-substituted benzoic acid. Although the mechanism was not investigated, the role of NO_x_ was likely catalytic.

**Scheme 49:**
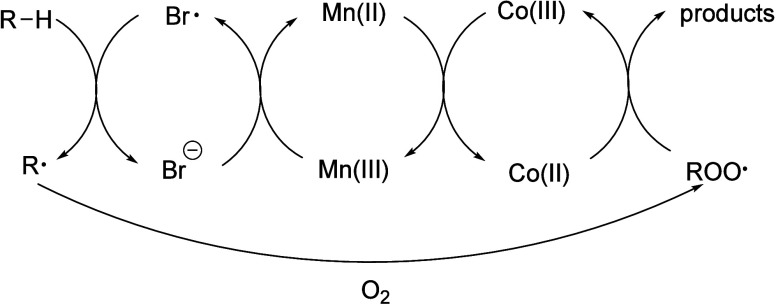
Simplified Mechanism for the Amoco Mid-Century Process for Hydrocarbon Oxidation^431^

The Amoco Mid-Century process is an important industrial process for hydrocarbon oxidation, especially the oxidation of *p*-xylene to terephthalic acid.^431^ The most common catalyst system is a mixture of Co(acetate)_2_, Mn(acetate)_2_, and NaBr, all dissolved in acetic acid. Br^–^ is the precursor of Br^•^, which initiates hydrocarbon oxidation by abstracting H^•^. The resulting alkyl radicals are trapped by O_2_ to form alkylperoxy radicals. Co(II) reacts with these radicals to form the products and, in so doing, is oxidized to Co(III). Mn(II) reduces Co(III) to Co(II) and the resulting Mn(III) reoxidizes Br^–^ to Br^•^ to complete the catalytic cycle (Scheme 49). A mixture of ammonium metavanadate and NaBr in water can also catalyze hydrocarbon oxidation.

This process was used to convert several polymers, including PE, PP, and PS (tacticities not specified), poly(4-vinylpyridine), poly(2-vinylpyridine-*co*-styrene), poly(butylene)terephthalate (PBT), PET, polyethylene(naphthanate) (PEN), poly(bisphenol A)carbonate (PC), and PVC.^432^ In less than 4 h under 70 bar air, PP or PE were fully converted to small molecule oxygenates. The oxidation of PP (*M*_w_ = 12,000 g/mol, *M*_n_ = 5,000 g/mol) catalyzed by the Co/Mn/Br combination at 180 °C gave acetic acid as the main product, in 53 mol% yield (relative to monomer subunits), as well as acetone in 5.6 mol% yield. Other products included methyl acetate, bromoacetic acid, and succinic acid. The V/Br combination gave slightly higher yields of acetic acid. PE proved to be more resistant to oxidation than PP and gave dicarboxylic acids (HOOC(CH_2_)_*n*_COOH, where *n* = 2, 3, 4), as the main products as well as smaller amounts of monocarboxylic acids. Addition of Zr(OAc)_4_ to the Co/Mn/Br catalyst system improved the yields, attributed to the formation of an unspecified Co–Zr complex.^431^

**Scheme 50:**
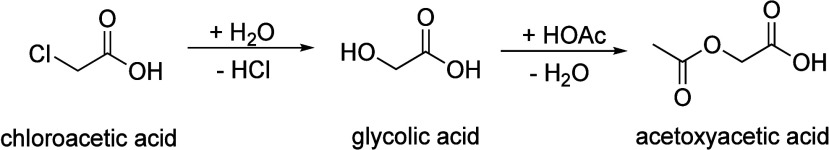
Hydrolysis of Chloroacetic Acid and Subsequent Condensation with Acetic Acid

PS was oxidized to benzoic acid in 88 % yield, accompanied by traces of benzil, using the Co/Mn/Zr/Br catalyst system. Br-free catalyst systems, such as Co/*N*-hydroxyphthalimide (NHPI), were also tested but gave much lower yields. For the oxidation of PVC, the Co/Mn/Br system produced a mixture of chloroacetic acid (35 mol%, relative to monomer subunits), acetoxyacetic acid (16 mol%) and glycolic acid (3.3 mol%). The last two were suggested to arise from reactions of chloroacetic acid (Scheme 50).

Oxidation of PVC can yield a range of undesirable byproducts, some of which are quite hazardous (and are not always acknowledged to be so). *Notably, chloroacetic acid is highly toxic.* Even HCl is problematic, and can cause equipment corrosion. Dechlorinating PVC prior to its oxidation may be safer.

**Scheme 51:**
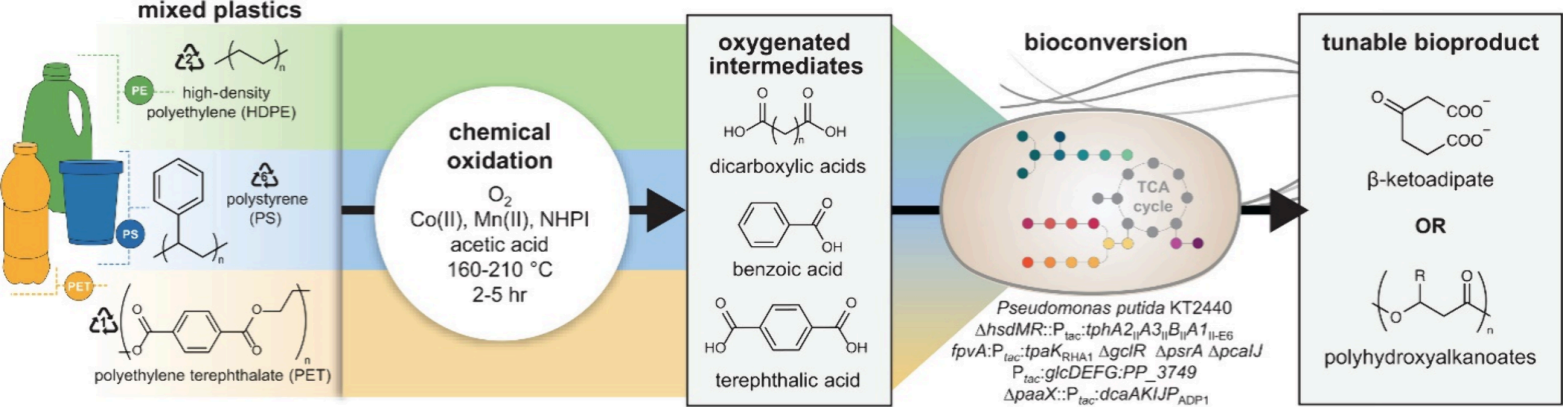
Two-Step Chemo-biocatalytic Strategy for Upcycling Mixed Plastic Waste via Initial Chemical Oxidation, Followed by Bioconversion of the Oxygenated Molecular Fragments Reproduced with permission from ref (434). Copyright 2022, AAAS.

*N*-hydroxyphthalimide (NHPI) can replace NaBr as the cocatalyst in Co/Mn-catalyzed oxidation, and is not corrosive to reactors in the way that bromine is.^433^ Co/Mn/NHPI in acetic acid was used to catalyze the oxidations of HDPE, PS, and PET, both individually and as a mixture, to small molecules under 8 bar O_2_, with 72 bar N_2_ added to suppress flammability (Scheme 51).^434^ Notably, when HDPE alone was oxidized, the process yielded dicarboxylic acids with carbon numbers ranging from C_4_ to C_22_, in approx. 34 mol% yield (relative to polymer carbon). PS was oxidized to benzoic acid in more than 60 mol% yield. The oxidized mixed plastic waste was used as a substrate for subsequent biological conversion with the bacterium *Pseudomonas putida*. Strain AW307 converted the oxygenates to β-ketoadipate, a monomer that can be used to make a polyamide, while strain AW164 gave polyhydroxyalkanoates (containing 3-hydroxydodecanoic and 3-hydroxydecanoic acids). These natural materials have potential applications as biodegradable polymers.

**Scheme 52:**
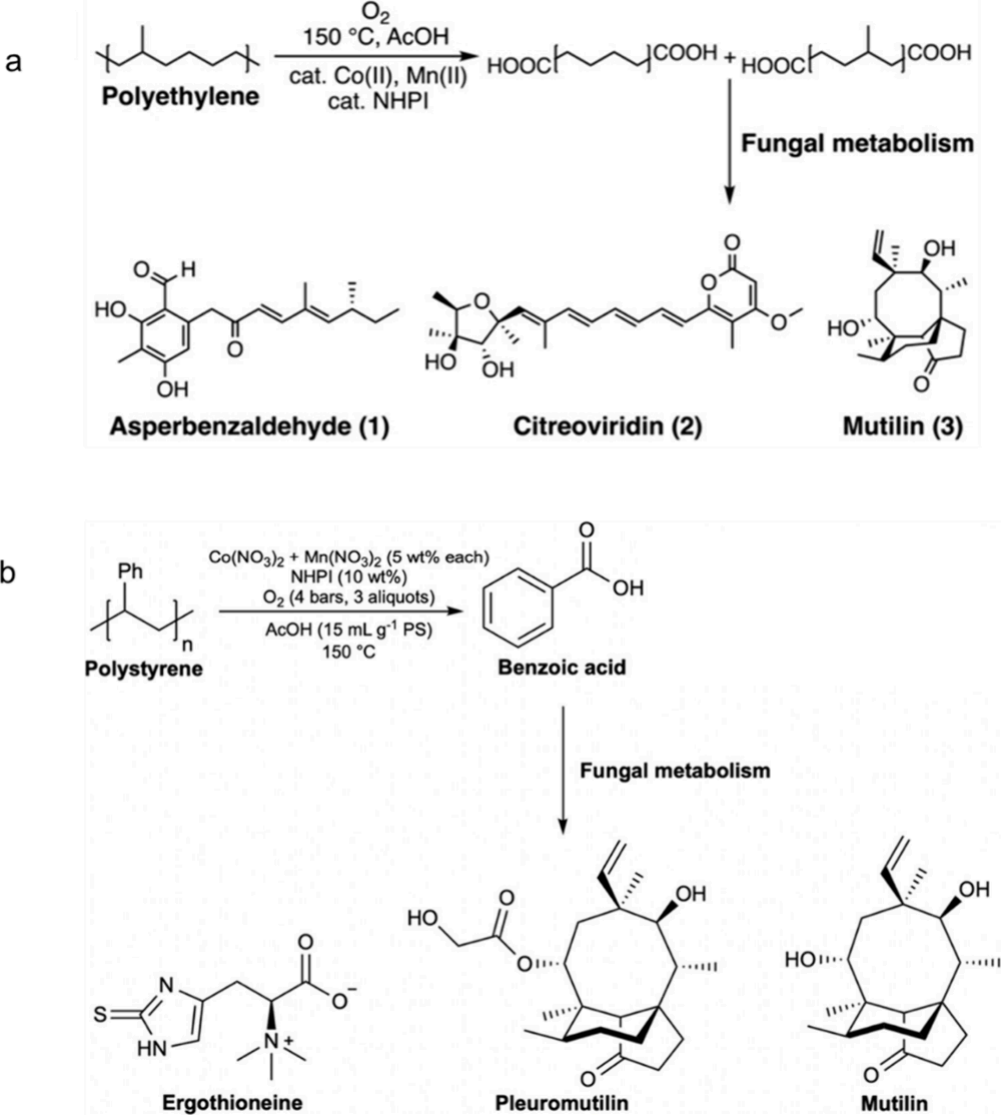
(a) Catalytic Oxidation of PE to Small-Molecule Dicarboxylic Acids; and (b) Catalytic Oxidation of PS to Benzoic Acid, Followed by Upgrading via Fungal Metabolism (a) Reproduced with permission from ref (435). Copyright 2022 Wiley. (b) Reproduced with permission from ref (436). Copyright 2023 American Chemical Society.

Dicarboxylic acids resulting from PE and benzoic acid derived from PS were converted by the fungus *Aspergillus nidulans* to metabolites with medicinal applications (Scheme 52).^435,436^ Although the potential value of these products is high, production volumes would be very small. Consequently, the impact on plastic waste valorization would likely be minimal.

**Scheme 53:**
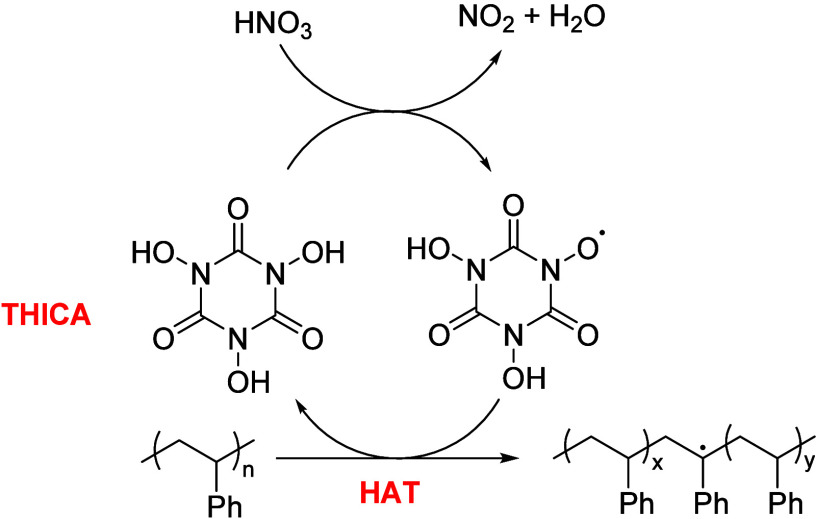
Proposed Initiation Step in PS Oxidation Catalyzed by THICA^437^

The organocatalyst NHPI and its derivatives can also catalyze the oxidative upcycling of PS to carboxylic acids, in the absence of a metal.^437^ When PS (104 mg, *M*_n_ = 140,000 g/mol, *M*_w_ = 230,000 g/mol), an organocatalyst (0.01 mmol), HNO_3_ (70 % v/v in H_2_O, 0.2 mL) and glacial acetic acid (2 mL) were heated at 120 °C in air for 24 h, the product mixture was dominated by benzoic acid and 4-nitrobenzoic acid. Among the organocatalysts tested, 4-F-NHPI and *N*,*N*′,*N*″-trihydroxyisocyanuric acid (THICA) gave the highest yields of benzoic acid (ca. 30 mol%, relative to mol styrene monomer) and 4-nitrobenzoic acid (ca. 10 mol%). The byproducts were formic acid and partially oxidized oligomers. In the proposed mechanism, HNO_3_ oxidizes THICA to an *N*-oxyl radical, which initiates the chain reaction via H atom transfer (HAT) from PS (Scheme 53). Various types of post-consumer PS were tested, and the reaction was scaled up to convert 10.4 g PS waste.

NHPI was also reported to be a mediator in the electrochemical oxidation of PS.^438^ NHPI is oxidized to the corresponding phthalimide-*N*-oxyl radical at the electrode surface. The radical abstracts a hydrogen atom from PS and regenerates NHPI. Subsequently, the radical on the PS chain reacts with O_2_ to initiate oxidative depolymerization. The products were monomers and dimers, mainly ketones, benzaldehyde and benzoic acid, as well as various products derived from NHPI. However, the benefits appear limited due to the low selectivity, when compared to alternative methods for PS oxidation.

#### 5.2.4 Catalytic Oxidation of Polyolefin Pyrolysis Oils

**Figure 69:**
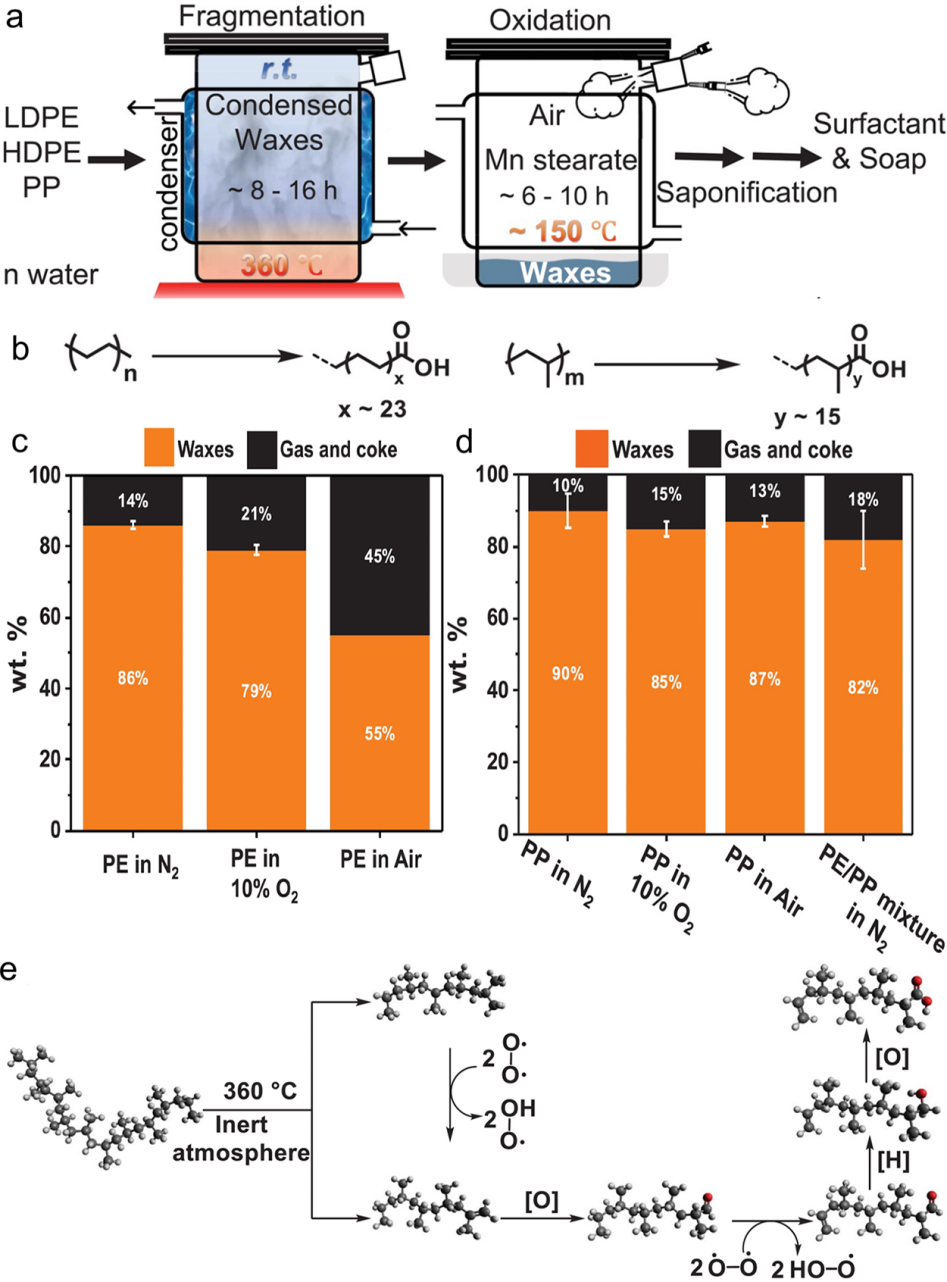
(a) Schematic of the two-step upcycling of commercial POs to long-chain organic acids. (b) Overall reactions, showing the conversion of PE and PP to long-chain organic acids; (c) product yields from PE “pyrolysis” in various atmospheres; (d) product yields from PP “pyrolysis” in various atmospheres, as well as from a waste PE/PP mixture; and (e) proposed mechanism for polymer oxidation. Adapted with permission from ref (439). Copyright 2023, AAAS.

Pyrolysis was combined with catalytic oxidation in a two-step process to convert POs to long-chain organic acids.^439^ The starting materials were PE (*M*_w_ = 97,000 g/mol, *Đ* = 2), PP (*M*_w_ = 158,000 g/mol, *Đ* = 4), and a mixture of post-consumer PO waste (25 wt% HDPE, 25 wt% LDPE, 25 wt% PP, 25 wt% unsorted PE/PP). In the first (pyrolysis) step, the solid was heated under N_2_, or in O_2_ (10 vol% in N_2_), or in air (note: only the reaction under N_2_ is technically pyrolysis). The temperature of the reactor base was set to 360 °C, while the reactor body was cooled, resulting in the lid being close to room temperature, Figure 69a. A strong temperature gradient was deemed important for obtaining hydrocarbon fragments of the desired molecular weight (i.e., waxes). In the case of PE, pyrolysis under N_2_ yielded a mixture of alkenes and alkanes, ranging from 300 to 1000 g/mol. Heating under 10 vol% O_2_ or in air also generated ketones, aldehydes, and esters, with molecular weights from 200 to 900 g/mol.

In a second reactor, the waxes were oxidized in flowing air in the presence of a Mn(II) stearate catalyst at 150 °C. Long-chain organic acids and a minor amount of long-chain organic esters were formed in this oxidation step; the esters were converted to the corresponding carboxylic acids by subsequent saponification. The overall reaction was therefore the conversion of PE to long-chain organic acids with molecular weights up to 700 g/mol (Figure 69b). Ketones and aldehydes were also formed.

Similar outcomes were observed for PP, with minor differences depending on the pyrolysis atmosphere (Figure 69c, d). A proposed mechanism, based on DFT simulations, is shown in Figure 69e. The oxidation of alkanes to carboxylic acids was suggested to proceed sequentially via alkene, aldehyde, and alcohol intermediates. However, alkanes can be oxidized directly to alkylperoxy radical intermediates (ROO^•^, see section 5.1.1.1), which can be further oxidized to carboxylic acids. Consequently, some of the proposed intermediates are unnecessary.

The upcycling of PO pyrolysis oil to long-chain organic alcohols was reported by a different multistep route. The process involves (1) thermal depolymerization to produce a partially unsaturated pyrolysis oil, followed by (2) catalytic alkene hydroformylation to generate aldehydes, and (3) catalytic aldehyde hydrogenation.^440^ Pyrolyses of virgin HDPE, PP, and LDPE, as well as recycled HDPE, were conducted in a fluidized bed at 500 °C, with a residence time of 20 s. The pyrolysis oils from post-consumer recycled HDPE were subjected to batch distillation under reduced pressure up to 165 °C, producing a light cut (C_<11_, 73 wt%, with 1-pentene and 1-hexene being the most abundant hydrocarbons) and a heavy cut (C_>10_, 27 wt%). According to 1D GC-FID, 2D GC×GC-FID, ^13^C NMR, and ^1^H-^13^C HSQC NMR analysis of the light cut, as well as NO ionization spectroscopy analysis of the heavy cut, the pyrolysis oils contained large amounts of alkenes (34–50 wt%), as well as alkanes (20–39 wt%), monoaromatics (11–23 wt%), alkadienes (6–13 wt%), and dicyclo-aromatics (<5 wt%), with carbon numbers ranging from C_5_ to C_40_. The hydrocarbon distribution varied with the PO feedstock: HDPE gave the most alkenes and alkadienes, while PP gave the least. Elemental analysis showed that the pyrolysis oil generated from the waste plastic also contained trace amounts of heteroatoms (N, O, Cl) and metals (Fe, Si, K, Ca, S, Zn, Cu, Ti, Al).

**Figure 70:**
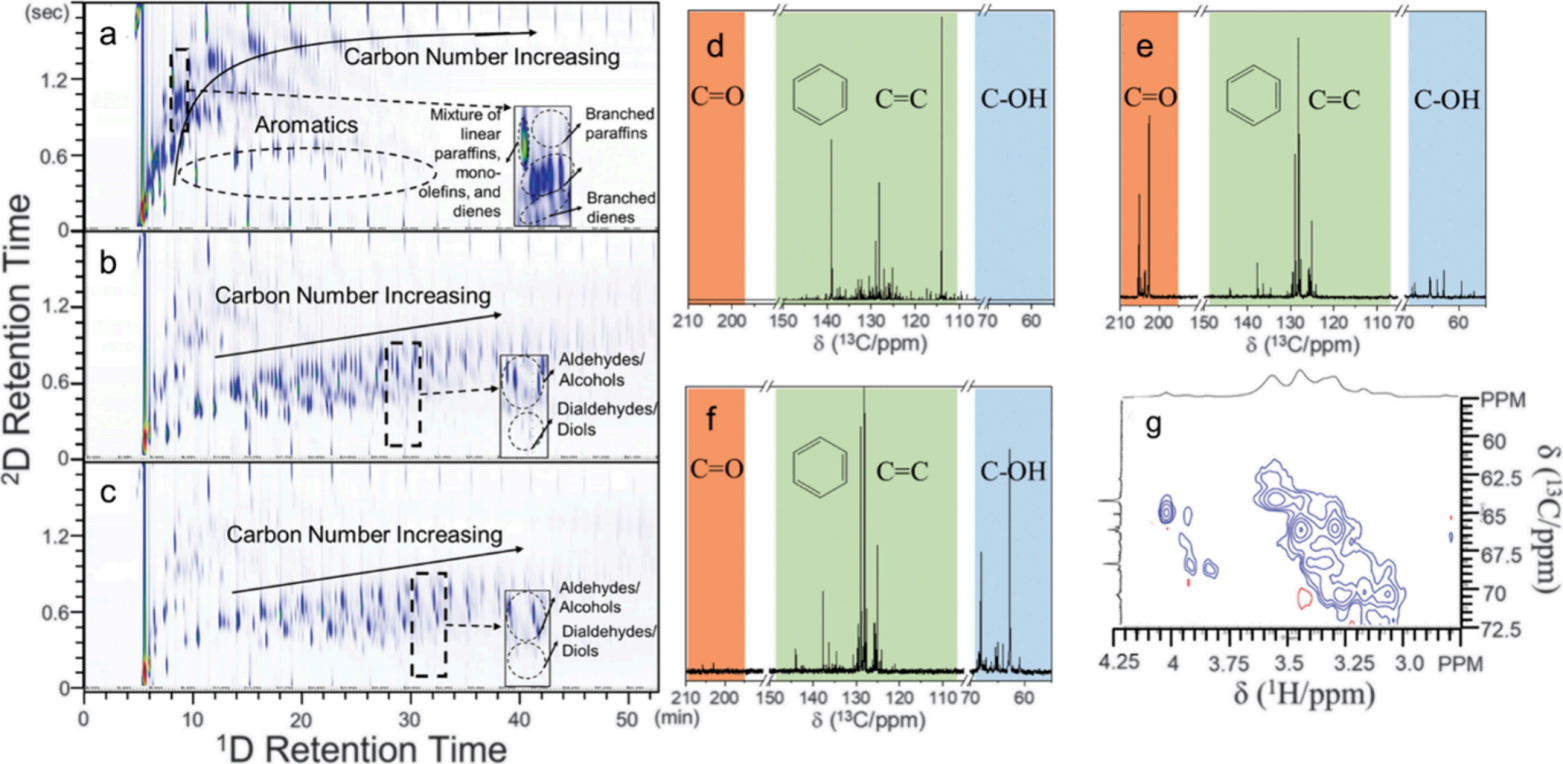
Comparison of 2D GC analyses and ^13^C NMR spectra for (a, d) HDPE pyrolysis oil (light cut); (b, e) after hydroformylation of the pyrolysis oil; and (c, f) after hydrogenation of the hydroformylated oil, as well as (g) ^1^H-^13^C HSQC NMR of the hydrogenated oil (blue and red represent signals for primary and secondary carbons, respectively). Reproduced with permission from ref (440). Copyright 2023, AAAS.

Hydroformylation of the pyrolysis oils (20–40 g) with syn gas (70 bar, containing equimolar CO and H_2_) was performed at 120 °C in a batch reactor with Co_2_(CO)_8_ as the catalyst (10 wt%). Light and heavy cut pyrolysis oils behaved similarly. Products from the light cut oil were analyzed by 2D GC×GC-FID, Figure 70a,b. Small changes in peak positions in the first dimension (^1^D) retention time suggest that hydroformylation led to only minor changes in carbon numbers. However, the formation of aldehydes caused new features to appear in the region characteristic of alcohols and aldehydes in the second dimension (^2^D). Most alkenes (ca. 90 %) were converted, resulting in aldehyde contents up to 60 wt%. The appearance of carbonyl peaks in the ^13^C NMR confirmed the formation of aldehydes (presumably, both mono- and dialdehydes), Figure 70d,e.

The hydroformylated oils were hydrogenated in a continuous process (50 sccm) at 100 °C, using a Ni/SiO_2_ catalyst (20 wt% Ni) under 78 bar H_2_. The Co hydroformylation catalyst had to be removed because it inhibited the hydrogenation catalyst. A shift in the 1D retention times (Figure 70c) implied alcohol (and dialcohol) formation, since alcohol retention times are longer than aldehyde retention times for the same carbon number. Most (>90 %) of the aldehydes were hydrogenated; ^13^C NMR peaks in the carbonyl region disappeared, and peaks in the alcohol region appeared (Figure 70f). ^1^H–^13^C HSQC NMR confirmed that most are primary alcohols (Figure 70g).

**Figure 71:**
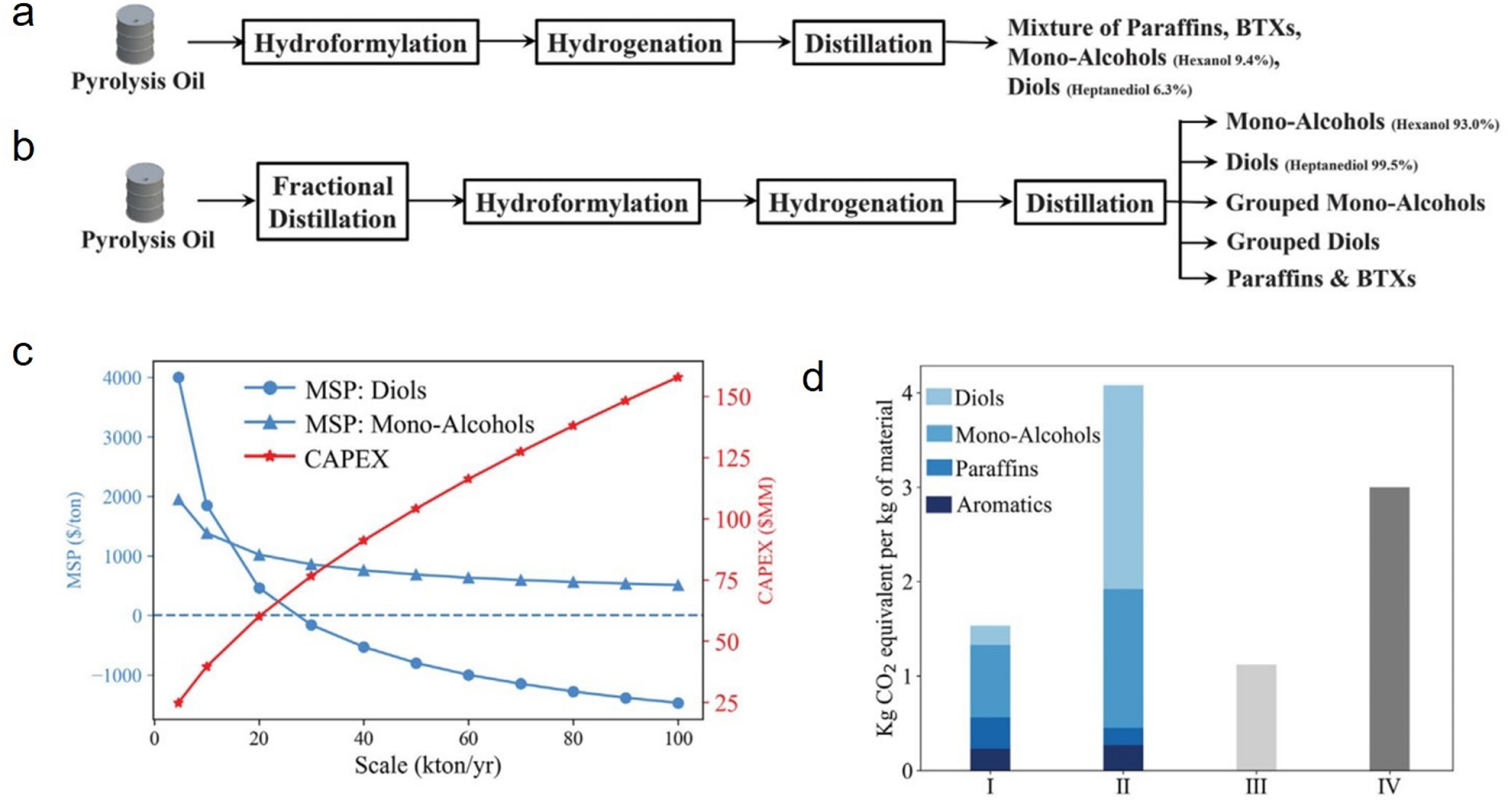
Comparison of proposed pyrolysis oil upgrading processes: (a) without, and (b) with separation prior to hydroformylation; (c) CAPEX and product MSPs analysis; (d) comparison of GHG emissions from (I) the proposed technology for converting 1 kg plastic to chemicals, (II) producing the same amount of chemicals using conventional technologies, (III) converting 1 kg plastic to alkenes by pyrolysis and steam cracking, and (IV) incineration of 1 kg plastic. Reproduced with permission from ref (440). Copyright 2023, AAAS.

Using the same approach, high purity monoalcohols (e.g., 1-hexanol, 91%) and diols (e.g., 1,7-heptanediol, 99.5%) were produced by distilling the pyrolysis oil, recovering different fractions, and performing hydroformylation-hydrogenation on each cut, Figure 71a,b. Estimated minimum selling prices (MSP) and capital expenditure (CAPEX) analyses were promising. The estimated GHG emissions, 1.6 kg CO_2_-equiv./kg plastic, were lower than traditional alcohol production methods, and lower than plastic incineration, Figure 71d. CO_2_ emissions for producing diols by this route were estimated to be 10× lower than by conventional routes starting from petroleum. The strategy should also be compatible with alkanes produced by plastic pyrolysis followed by steam cracking to alkenes.

### 5.3 Photocatalytic Oxidation of Polyolefins

To-date, most PO upcycling via catalytic photooxidation has focused on PS. The main product is benzoic acid, whose stability slows its overoxidation to CO_2_. There is no clear conclusion on whether tacticity affects the oxidation of PS, since most papers do not report tacticity. In contrast, photooxidation of the other major PO types (PE, PP, and PVC) leads readily to CO_2_, diminishing the effectiveness of the method. Other photooxidation products for these polymers include formic and acetic acids, generally in less than 20 % yield. In addition, HCl formed during PVC oxidation may poison catalysts. Many papers lack essential molecular weight data, with some papers reporting only *M*_w_ without *M*_n_ or *Đ*.

#### 5.3.1 Photocatalytic Oxidation of Polystyrene

**Scheme 54:**
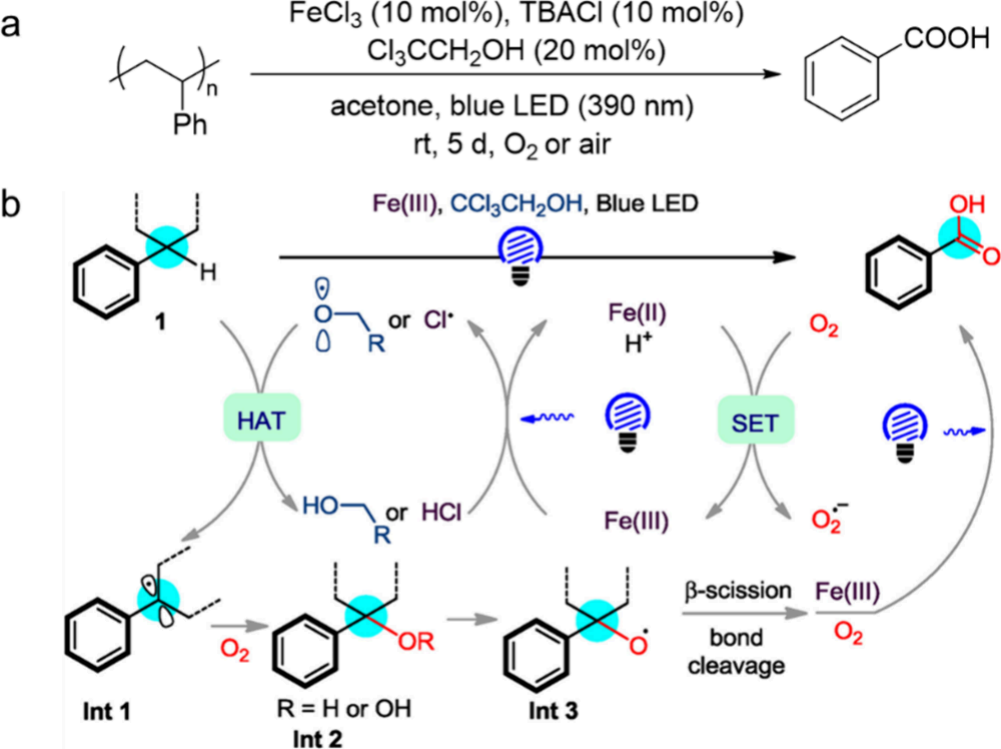
(a) Photooxidation of PS to Benzoic Acid; and (b) Proposed Mechanism Adapted with permission from ref (441). Copyright 2021, Wiley.

Inspired by the photocatalytic oxidation of alkylaromatics catalyzed by FeCl_3_, PS (*M*_w_ = 229,300 g/mol, *Đ* = 1.63) was converted to benzoic acid (Scheme 54a).^441^ The rate was very slow. After 5 d in air, 42 mg PS was converted to benzoic acid in 54 % yield (based on mol styrene monomer). The yield under 1 bar O_2_ was 67 %. Post-consumer PS foam also gave benzoic acid, in 64 % yield under 1 atm O_2_. In the proposed mechanism (Scheme 54b), the cocatalysts Cl_3_CCH_2_OH (20 mol%) and tetrabutylammonium chloride (TBACl, 10 mol%) generated alkoxy radicals^442^ and Cl radicals,^443,444^ respectively, in the presence of Fe(III) and blue LED light. These radicals abstracted hydrogen atoms from PS to form benzylic radicals. Subsequent proposed steps follow the autoxidation mechanism in Scheme 45. Eventually, the benzyloxy radical undergoes β-scission before being further oxidized to benzoic acid.

**Figure 72:**
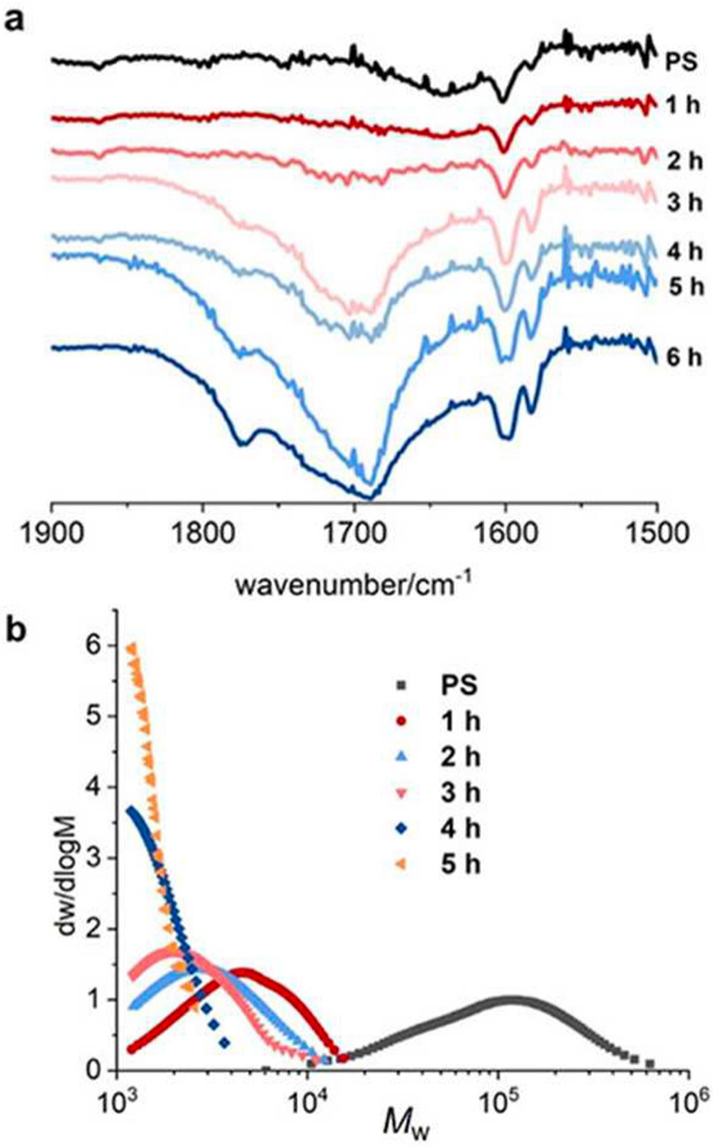
(a) IR spectra of PS recorded during 6 h of Fe-catalyzed photooxidation; and (b) GPC analysis of the oxidized PS recovered during the first 5 h of reaction. Reproduced with permission from ref (445). Copyright 2021, Wiley.

Photooxidation of PS to benzoic acid catalyzed by FeCl_2_ was also reported.^445^ Since FeCl_2_ is readily oxidized to FeCl_3_ under the reaction conditions (indeed, this reaction is invoked in the mechanism in Scheme 54), the active metal catalyst is likely the same in both cases. PS (52 mg, *M*_w_ = 145,000 g/mol) and FeCl_2_ (from 2 to 10 mol%, based on styrene monomer) were stirred in a mixed solvent CH_2_Cl_2_/CH_3_CN (3:2) or neat CH_3_CN under 1 bar O_2_ at ambient temperature while being irradiated with a 400 nm LED. In the first 6 h, only traces of benzoic acid were detected, although carbonyl groups were observed in the IR spectrum of the polymer residue (Figure 72a). During this period, *M*_w_ decreased to about 1,000 (Figure 72b), indicating extensive chain cleavage. Subsequently, the yield of benzoic acid increased significantly, reaching about 60 % after 66 h. CO_2_ was also observed as a product. When photooxidation was investigated with PS particles (*M*_w_ = 35,000 or 350,000 g/mol), syndiotactic PS (*M*_w_ = 222,000 g/mol), atactic PS (*M*_w_ = 202,000 g/mol), and post-consumer PS waste, such as foam board (*M*_w_ = 222,000 g/mol) and a cup (*M*_w_ = 166,000 g/mol), all yielded similar outcomes. The study was also extended to PS copolymers, including styrene-allyl alcohol, styrene-acrylonitrile, acrylonitrile-butadiene-styrene, and styrene-maleic anhydride. In each case, the yield of benzoic acid was slightly lower compared to the reaction of pure PS.

**Figure 73:**
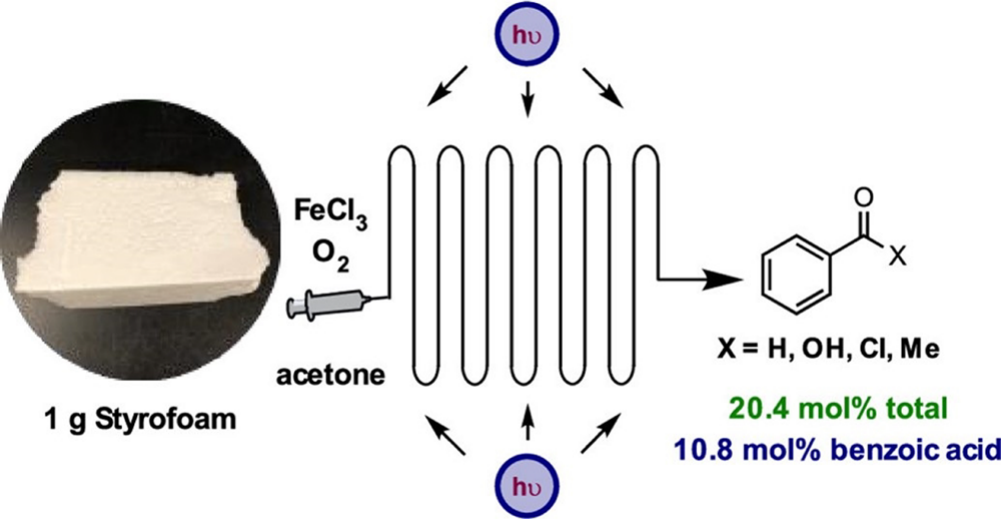
Schematic of PS photooxidation in flow. Reproduced with permission from ref (446). Copyright 2022, American Chemical Society.

Photooxidation of PS (20 mg, *M*_n_ = 89,000 g/mol, *Đ* = 6.33) was also reported to be catalyzed by FeCl_3_ (10 wt%) assisted by a white LED.^446^ After 20 h, PS was converted to a mixture of benzaldehyde, benzoic acid, benzoyl chloride, and acetophenone, in a total yield of about 10 mol%. Other products were oxidized oligomers (*M*_n_ = 800 g/mol, *Đ* = 1.91). Four types of commercial PS products, including a coffee cup lid and Styrofoam, were converted to small molecules under the same conditions. The yield was very low (2.2 %) for the coffee cup lid, likely because its black color (presumably carbon black, but referred to as black "dye") interfered with light absorption. Photooxidation of PS foam was achieved at gram scale in a flow reactor (Figure 73).

**Figure 74:**
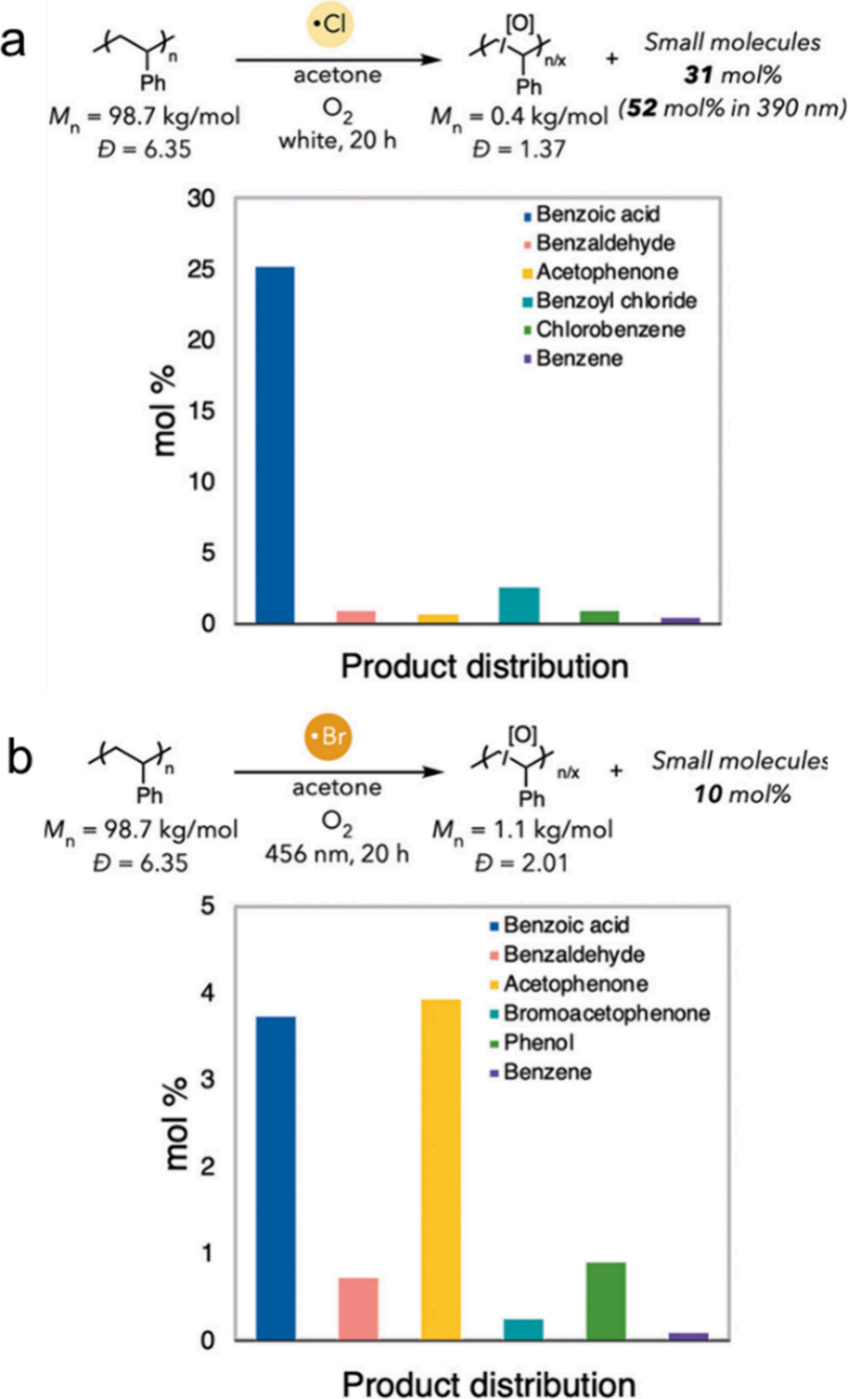
Product distributions from PS photooxidation catalyzed by (a) FeCl_3_; and (b) FeBr_3_. Reproduced with permission from ref (447). Copyright 2023, American Chemical Society.

Further investigation of the FeX_3_-catalyzed photooxidation of PS revealed that the product distribution was affected by the identity of the halide.^447^ With FeCl_3_, the major small-molecule product was benzoic acid (Figure 74a). Use of FeBr_3_ yielded nearly equivalent amounts of benzoic acid and acetophenone (Figure 74b), although the total yield of small molecule products was reduced, presumably due to the lower reactivity of Br^•^ relative to Cl^•^. The difference was suggested to originate in the selectivity of the H^•^ abstraction step, based on results with model compounds and bond dissociation energies (BDEs). The higher BDE of the H–Cl bond was suggested to allow Cl^•^ to abstract H^•^ from both secondary (2°) and tertiary (3°) C–H bonds in PS, while Br^•^ abstracted H^•^ only from weaker 3° C–H bonds. Since H^•^ abstraction at 3° C–H bonds near the chain end should produce acetophenone, Br^•^ radical was proposed to react preferentially near chain ends, presumably due to steric effects. However, product selectivities were compared at constant time, instead of constant conversion, and acetophenone can be further oxidized to benzoic acid. Therefore, it will be the major product even with the FeBr_3_ catalyst, provided the reaction time is sufficiently long.

**Figure 75:**
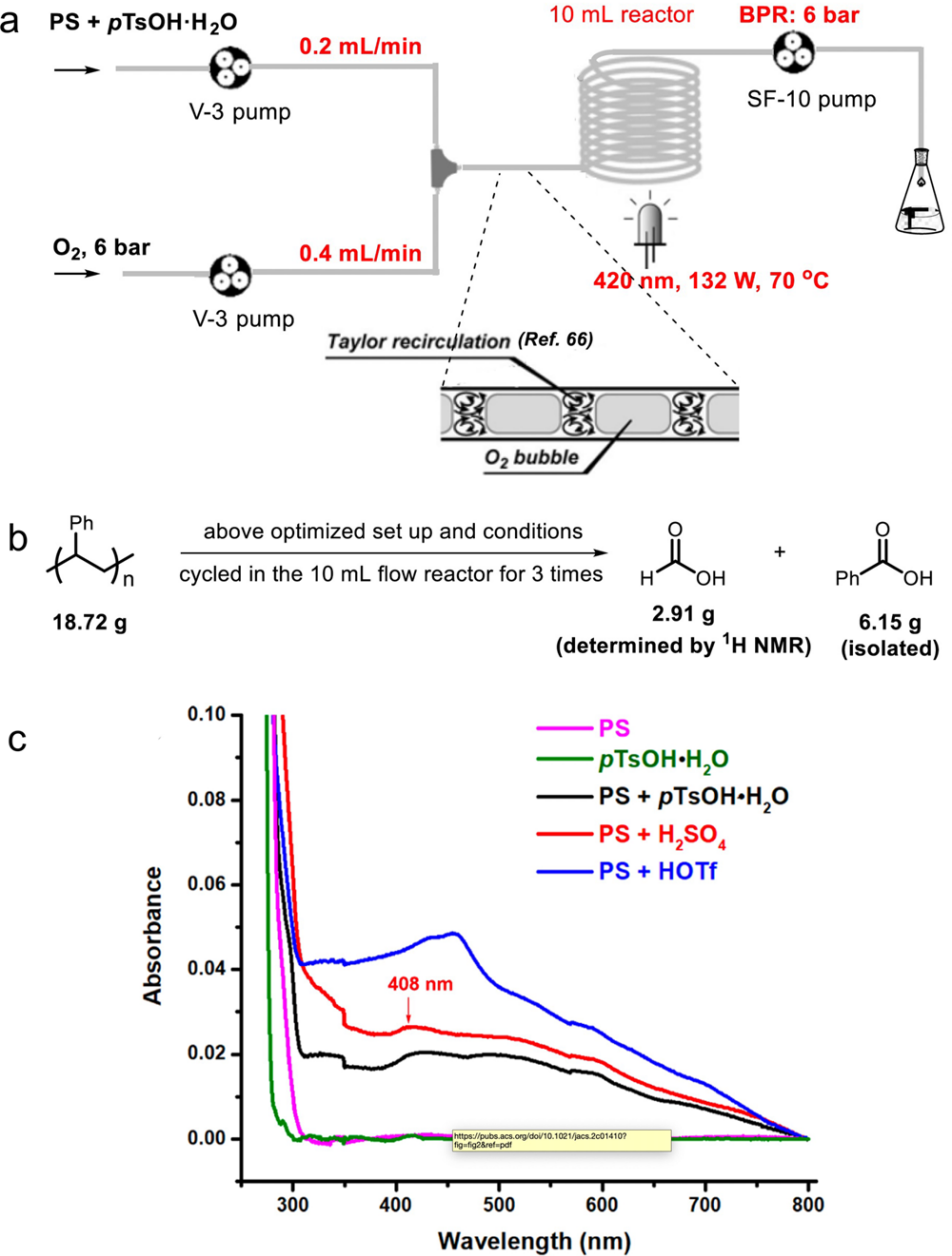
(a) Schematic for flow setup used in PS photooxidation; (b) scale-up of PS photooxidation; and (c) UV–vis spectra of PS-acid solutions. Adapted with permission from ref (448). Copyright 2022, American Chemical Society.

PS photooxidation appears to be catalyzed by simple Brønsted acids as well as transition metal catalysts. For example, *p*-toluenesulfonic acid (*p*-TsOH) was reported to catalyze the photooxidation of PS in benzene/MeCN (1:1), giving formic acid, benzoic acid, and acetophenone under 1 bar O_2_ after blue light irradiation at room temperature for 15 h.^448^ The main byproduct was partially oxidized PS with a lower molecular weight. Photooxidation was performed on PS samples of varying molecular weights, and with different PS waste materials (Table 11). The reaction was also conducted in a continuous flow system (1.04 g PS and 5 mol% *p*-TsOH in 5 mL solvent, flowing at 0.2 mL/min) at a slightly higher O_2_ pressure (6 bar) and temperature (70 °C) (Figure 75a). After a single pass, the effluent was recycled to the reactor to achieve a gram scale yield of oxidation products (Figure 75b). The nature of the active chromophore in the acid-catalyzed reaction was discussed. Solutions of PS with strong acids showed a broad absorption across the visible region, although no such absorption was observed for PS and *p*-TsOH separately (Figure 75c). An unspecified adduct between PS and *p*-TsOH was suggested to act as a photosensitizer.

**Table 11:**
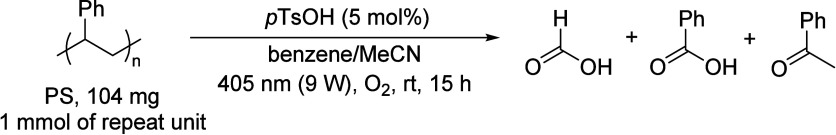
Products of Photocatalytic Oxidation of Various Types of PS and PS Waste^448^

	yields (%)^a^		
sample	formic acid	benzoic acid	benzophenone
PS (*M*_w_ = 192,000 g/mol)	67	50	2
PS (*M*_w_ = 35,000 g/mol)	57	38	4
PS (*M*_w_ = 800-5000 g/mol)	60	40	5
PS cup lid	62	41	2
yogurt container	58	40	2
PS packing foam pieces	64	48	2
PS foam waste	61	46	2
PS food box	62	44	2
laboratory weighing boat	61	43	2

a Reported yields are calculated based on styrene monomer units, making the yield of formic acid appear relatively high.

In another PS photooxidation method, fluorenone (20 mol% relative to styrene monomer) served as an organocatalyst.^449^ The reaction was conducted in ethyl acetate with 1 equiv. H_2_SO_4_ (relative to styrene monomer) under O_2_ at 50 °C. The solution was irradiated for 16 h using a blue LED. The small molecule products were benzoic acid (ca. 40 mol% yield) and other aromatics (acetophenone, ethyl benzoate, benzaldehyde, phenylglyoxylic acid, totaling about 20 mol% yield), with byproducts CO, CO_2_, and oligomers with molecular weights ranging from 200 to 800 g/mol. The method was applied to pure PS (*M*_w_ = 260,000 g/mol, *Đ* = 2.5 g/mol) and post-consumer PS foam (*M*_w_ = 250,000 g/mol, *Đ* = 4.2), achieving upcycling to benzoic acid at gram scale.

**Scheme 55:**
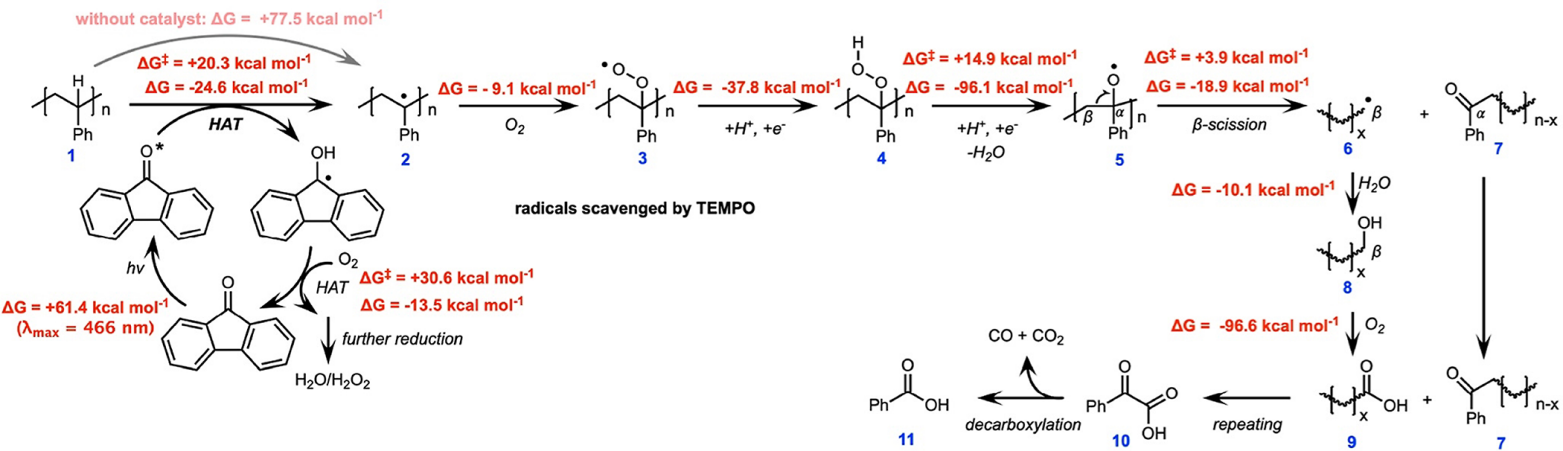
Proposed Mechanism for PS Photooxidation Catalyzed by Fluorenone Reproduced with permission from ref (449). Copyright 2022, American Chemical Society

In the proposed mechanism (Scheme 55), the reaction is initiated when the excited state of fluorenone abstracts H^•^ from PS to form a benzylic radical which is trapped by O_2_. Based on DFT calculations, H_2_SO_4_ promotes peroxide decomposition. A similar strategy for PS upcycling was reported using anthraquinone instead of fluorenone.^450^ In that case, less catalyst (5 mol%) was required and no strong Brønsted acid was present. PS (100 mg) was converted to benzoic acid in ca. 28% yield after 48 h. Curiously, when various post-consumer PS materials were tested, benzoic acid was produced in higher yields (30 - 60%) compared to the model PS, although there was no explanation for this difference in behavior.

**Scheme 56:**
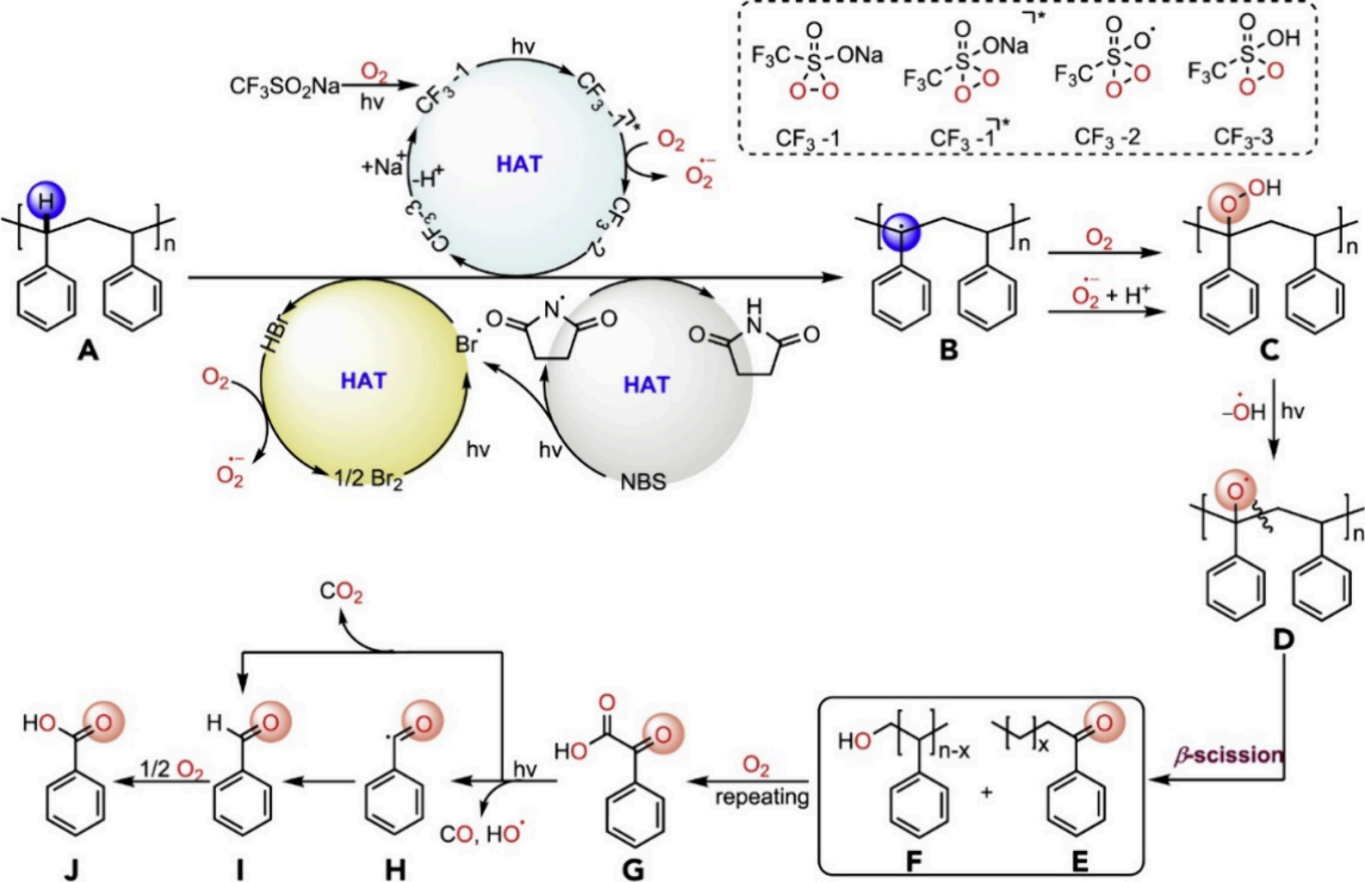
Proposed Mechanism for PS Oxidation Catalyzed by *N*-Bromosuccinamide Reproduced with permission from ref (451). Copyright 2022, Elsevier.

Another metal-free photocatalytic oxidation of PS to benzoic acid was reported.^451^ In the proposed H^•^ abstraction step, *N*-bromosuccinimide (NBS) generated Br^•^ and succinimide radicals, both of which can abstract H^•^ from a PS chain, initiating the chain reaction (Scheme 56). The addition of triflate enhanced the rate. Based on the appearance of a UV peak, it was proposed to react with O_2_ to form peroxide CF_3_-1, whose excited state reduces O_2_ to O_2_^–•^ (Scheme 56). However, O_2_^–•^ usually abstracts H^•^ from polymers to form polymeryl radicals R^•^ which are trapped by O_2_ (Section 5.1.1), rather than by O_2_^–•^ /HO_2_^•^.

**Figure 76:**
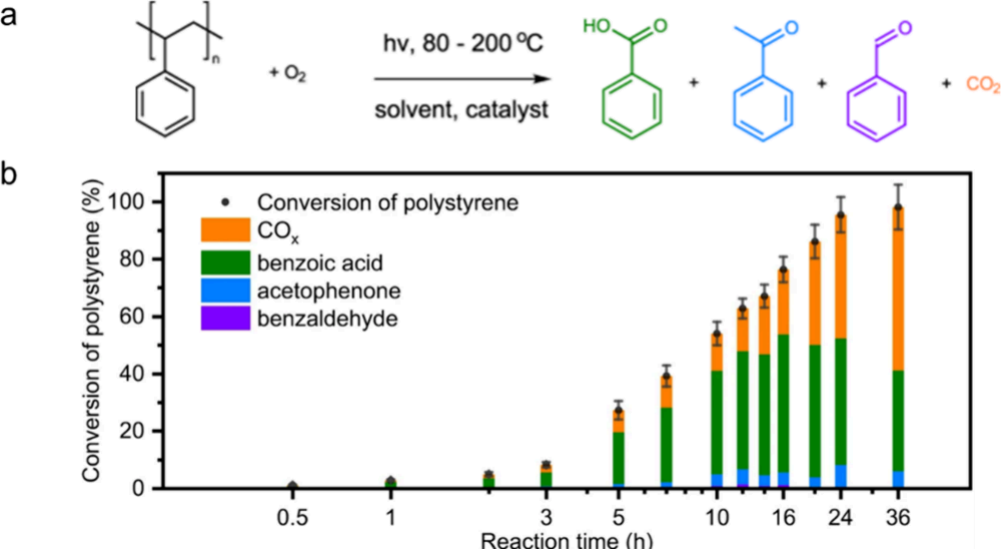
(a) PS oxidation to aromatic oxygenates, catalyzed by g-C_3_N_4_; and (b) evolution of the product distribution with time. Adapted with permission from ref (452). Copyright 2022, Springer Nature.

Graphitic carbon nitride (g-C_3_N_4_) can also serve as a photocatalyst for PS oxidation (Figure 76a).^452^ Aromatic oxygenates were obtained from PS (20 mg, *M*_w_ = 50,000 g/mol) in about 60 % yield (based on mol monomer subunits) when the reaction was carried out in CH_3_CN under 10 bar O_2_ at 150 °C for 24 h. CO_2_ and CO were byproducts. PS conversion surpassed 90 %, based on mol carbon recovered in the gas and liquid products relative to mol carbon in the PS starting material. Compared to the performance of other common photocatalysts, such as TiO_2_, ZnO, and ZnS, g-C_3_N_4_ resulted in the best balance between PS conversion and selectivity towards desired organic compounds. Longer reaction times and higher reaction temperatures gave undesirable over-oxidation. PS with molecular weights from 800 to 110,000 (*M*_w_), syndiotactic PS, PS pellets, and post-consumer PS cups all gave similar conversions and selectivities under these conditions. Notably, PS with a smaller molecular weight showed higher reactivity due to its better solubility.

Products were analyzed as a function of time. An unexplained lengthy induction period (3 h) preceded a rapid increase in yields. In the first 10 h, the molecular weight of the PS increased, attributed to the incorporation of oxygen and the formation of inter-chain cross-links, presumably as a result of radical-radical reactions. IR indicated the presence of carbonyl groups as well as hydroxyl groups associated with phenols and other alcohols. Benzoic acid was always the major small-molecule product; no dimers, trimers, or oligomers were detected. Based on these results, a mechanism was proposed in which PS is first oxidized without chain cleavage, followed by further oxidation leading to C–C bond cleavage to form hydroxylated alkylbenzenes, alkylphenones, and phenols. These intermediates are further oxidized to benzaldehyde, acetophenone and benzoic acid. Phenol was not found in the final products, suggesting it is readily oxidized to CO_2_.

Exfoliation of the g-C_3_N_4_ photocatalyst resulted in higher activity (inferred from the higher PS conversion). The effect was attributed to the higher surface, which also resulted in higher selectivity to benzoic acid.^453^ Process improvements further increased the yield of desired products. When the solution phase was removed after each hour of reaction to prevent overoxidation, the selectivity to organic products increased to 76 from 60 mol%. Partial oxidation and pyrolysis of PS in air at 220 °C for 40 h also promoted selectivity. A higher weight-hourly space velocity (WHSV) of the PS solution (0.3 mg/mL in MeCN) gave more benzaldehyde and acetophenone (51 and 31%, respectively), while a lower WHSV resulted in more benzoic acid (up to 74 %).

Organically-modified TiO_2_ was also reported to be effective as a photocatalyst for PS upcycling to benzoic acid.^454^ TiO_2_ was modified with potassium stearate or *N*,*N*-diethyl-3-(trimethoxysilyl)propan-1-amine (DTSPA). These materials showed stronger absorption in the visible region and higher photocurrent densities than unmodified TiO_2_ and gave higher yields. After 4 h irradiation with a 40 W LED (λ_max_ = 370 nm) under 1 bar O_2_ at room temperature, PS (10.4 mg, *M*_n_ = 103,400 g/mol, *M*_w_ = 250,700 g/mol) was converted to benzoic acid and trace acetophenone in a total yield of ca. 40 %. The byproducts were oxidized PS (*M*_n_ = 168,900 g/mol, *M*_w_ = 220,700 g/mol) and oligomers with *M*_n_ and *M*_w_ values of ca. 400. The slight increase in *M*_n_ for oxidized PS was caused by oxygen incorporation. No CO_2_ was detected. Various types of post-consumer PS waste gave benzoic acid in lower yields (18 to 40 %).

UO_2_^2+^ was investigated as a photocatalyst for PS oxidation to benzoic acid.^455^ A mixture of PS (0.48 mmol as monomer, 50 mg) and UO_2_(NO_3_)_2_ (5 mol%) in CH_2_Cl_2_ were irradiated with blue light under 1 bar O_2_ at room temperature for 72 h. Benzoic acid was formed in 46 % yield. Concentrated HCl improved the reactivity, possibly because the photocatalyst lifetime is pH-dependent. The acid may also help to dissolve PS. Two styrene-based copolymers, styrene-acrylonitrile (SAN) and acrylonitrilebutadiene-styrene (ABS), also gave benzoic acid in about 40 % yield based on mol styrene monomer.

#### 5.3.2 Photocatalytic Oxidation of Other Polyolefins

**Figure 77:**
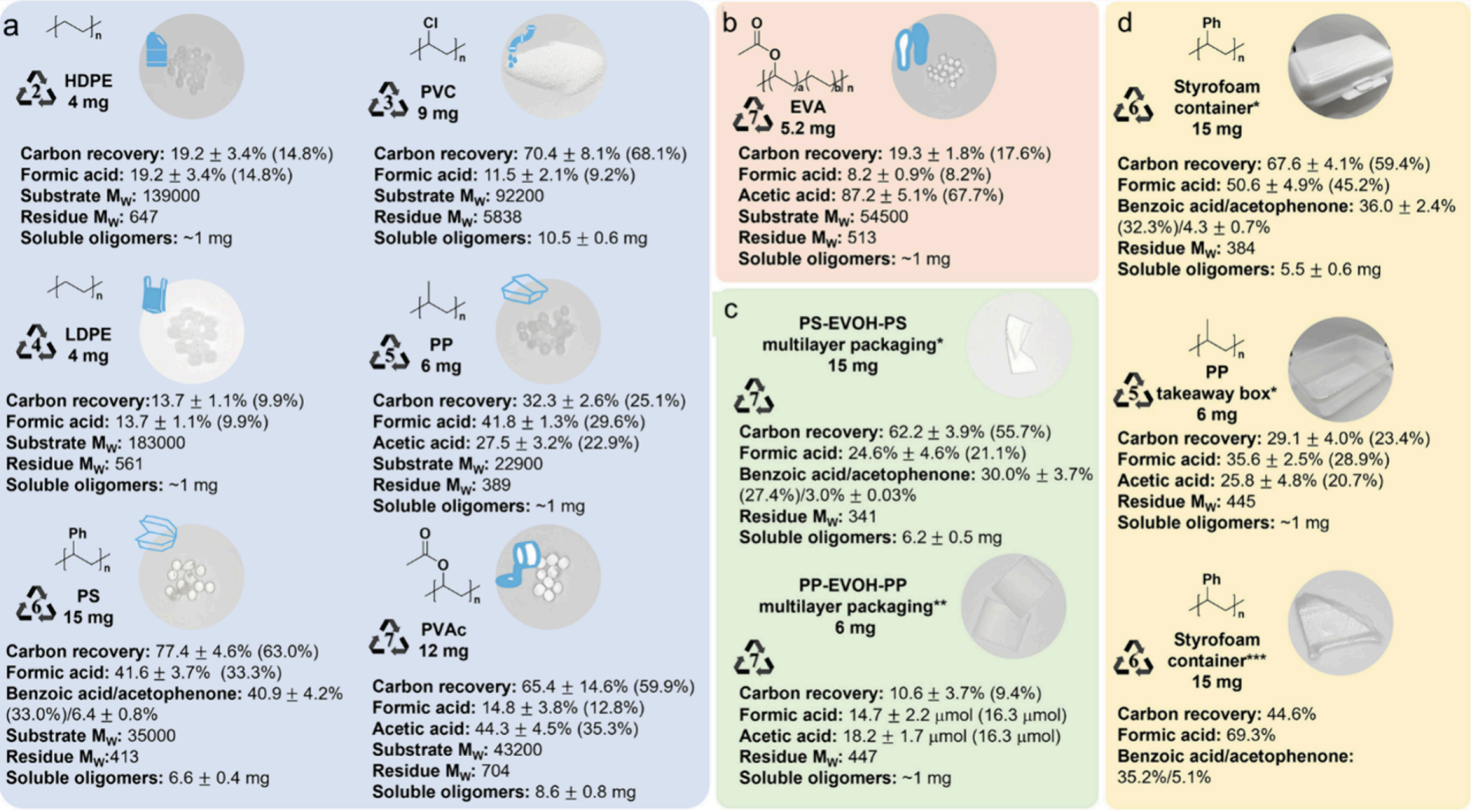
Outcomes for photooxidation of various types of plastics, catalyzed by V(O)(acac)_2_. Carbon recovery refers to the mol fraction of carbon present in the carboxylic acid products and/or soluble oligomeric products, relative to mol carbon in the original polymer. Reproduced with permission from ref (456). Copyright 2023, Elsevier.

Several commodity POs were converted to carboxylic acids via photooxidation catalyzed by V(O)(acac)_2_, with CO_2_ as the main byproduct.^456^ The catalyst (2 mol%, based on mol monomer, 0.02 M) was dissolved in CH_2_Cl_2_, and the solution was irradiated with a white LED (50 W) under O_2_ for 5 d. Although the reaction efficiency was low, the method appears versatile. Results for PET, PE, PVC, PP, and PS are summarized in Figure 77a. Poly(vinyl acetate) (PVAc), ethylene-vinyl acetate (EVA), multilayer packaging, and post-consumer PS and PP containers were also converted (Figure 77b–d). Low solubility polymers such as PE and PP were pre-heated with CH_2_Cl_2_ at 110 °C for 20 min to form a suspension. Post-consumer PS (1 g) was also converted in a flow reactor with a recycle stream. Benzoic acid and acetophenone were obtained in yields similar to those found in batch, but with a higher PS concentration (0.18 M) in the flow reactor compared to the batch reactor (0.02 M).

**Figure 78:**
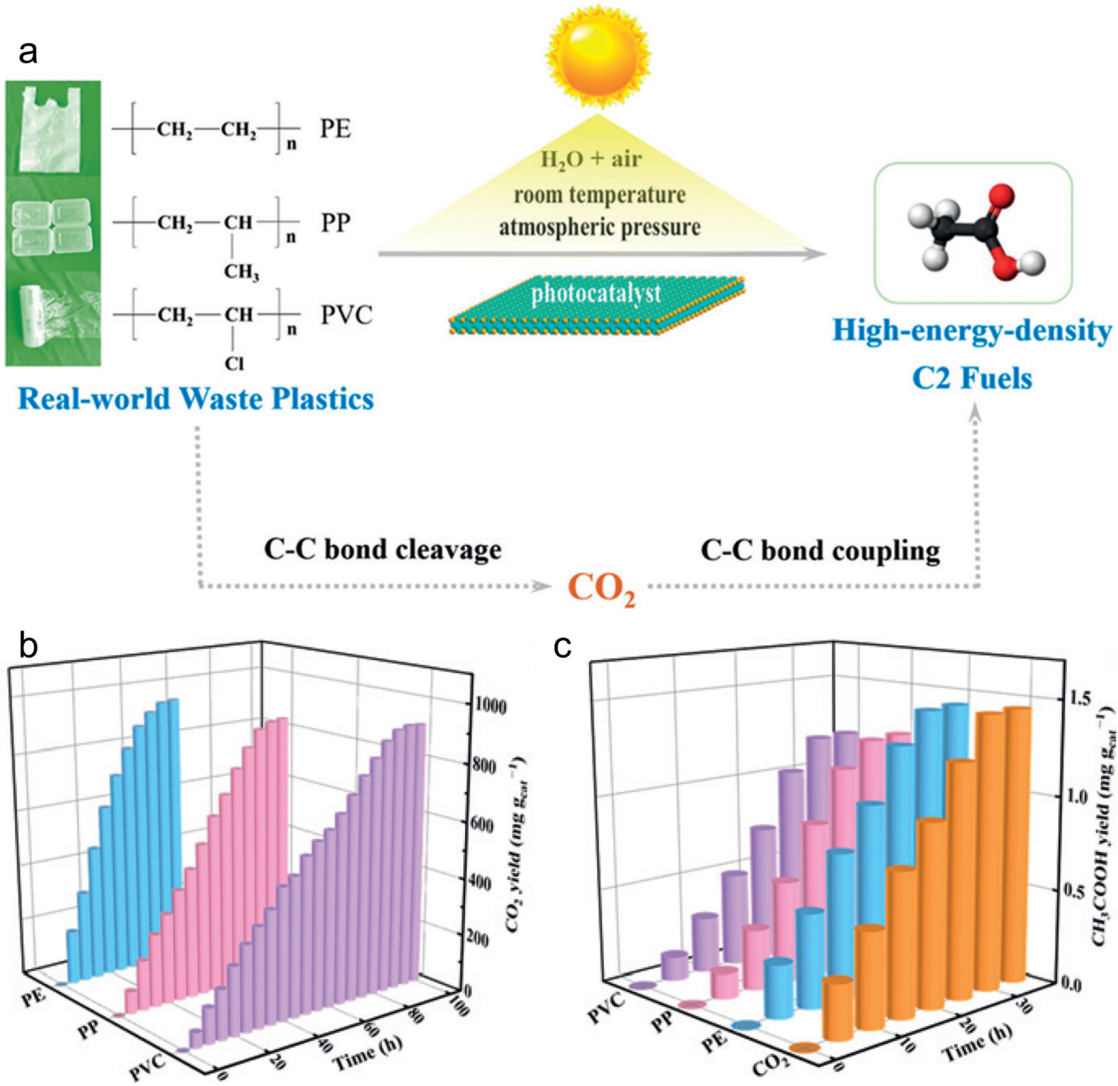
(a) Schematic of photo-oxidative conversion of PE, PP, and PVC to acetic acid; (b) CO_2_ yields; and (c) CH_3_COOH yields. Adapted with permission from ref (457). Copyright 2020, Wiley.

PE, PP and PVC were oxidized to acetic acid, using Nb_2_O_5_ as the photocatalyst (Figure 78a).^457^ PE or PP was immersed in water at room temperature under 1 bar air. After irradiation with a Xe lamp for 40 h (PE), 60 h (PP), or 90 h (PVC), the plastic was completely photodegraded. Although the major product was CO_2_, CH_3_COOH was also formed, in a very low yield (0.07 mg starting from 150 mg PE) (Figure 78b,c). The process was described as a route to “high density fuel”, but the tiny yield and the lack of precedent for the use of acetic acid as a fuel undercut these claims. The surprising proposed mechanism involved initial mineralization of the POs to CO_2_, then reduction of CO_2_ to acetic acid. Evidence was presented in the form of control reactions, including D-incorporation from D_2_O into the acetic acid, and photoreduction of CO_2_ to acetic acid. Given the complexity of the chain reactions in polymer photodegradation, facile isotopic exchange, and the very low yield of acetic acid, these control experiments are inconclusive.

**Figure 79:**
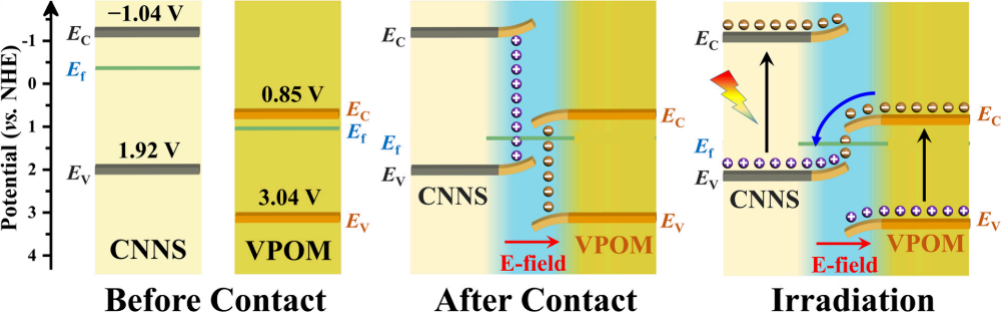
Proposed band structure of the VPOM/CNNS hybrid catalyst. Reproduced with permission from ref (458). Copyright 2022 Elsevier.

PE, PP and PVC were also converted to formic acid by photocatalytic oxidation in acetonitrile under O_2_.^458^ The photocatalyst was a combination of V-substituted phosphomolybdic acid (VPOM) and graphitic carbon nitride nanosheets (CNNS). No formic acid was detected when either catalyst component was used separately. In the presence of UV light, electron transfer from VPOM to CNNS was suggested to suppress electron-hole recombination and facilitate radical formation (Figure 79). Several waste POs, including PE bags, PP masks and PVC film, were oxidized in this way. However, the efficiency was low. After 36 h, the main product was CO_2_, and less than 7% of the carbon was found in the formic acid.

A few papers have reported the use of metal–organic frameworks (MOFs) as components of photocatalysts for PO upcycling to acetic acid. Ag_2_O/Fe-MOF was explored as a photocatalyst for PE upcycling,^459^ while ZnO/UiO66-NH_2_ and CDs/Zr-MOF (where CDs are carbon nanodots) were used as photocatalysts for PVC upcycling. The yields of acetic acid from PVC, up to 14 %, are relatively high for a PO photooxidation (other than PS).

### 5.4 Polyolefin Oxidation by H_2_O_2_

PO oxidation using H_2_O_2_ is usually conducted under thermal, rather than photochemical, conditions. At low temperatures, a catalyst is required due to the low intrinsic reactivity of H_2_O_2_ towards hydrocarbons. In the presence of a redox-active metal, H_2_O_2_ undergoes homolytic bond cleavage to generate hydroxyl and hydroperoxyl radicals, Scheme 47. They are reactive towards even the most inert POs.

**Figure 80:**
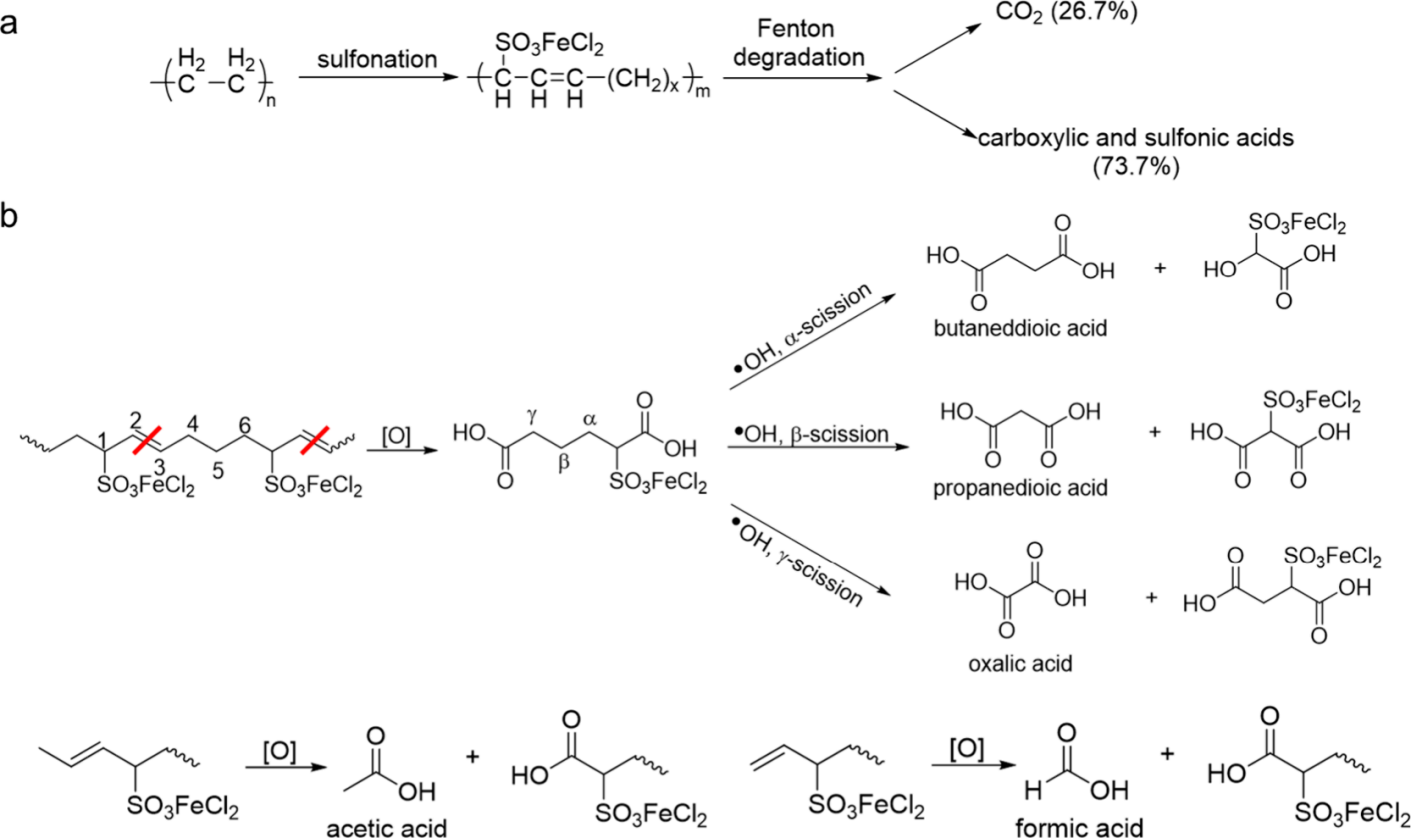
(a) Overall process for PE degradation to organic acids via an unsaturated sulfonated polymer, PESO_3_-Fe; and (b) proposed pathway for PESO_3_-Fe oxidation. Adapted with permission from ref (460). Copyright 2016, Wiley.

A two-step procedure involving PE sulfonation followed by Fenton oxidation resulted in the transformation of PE to short-chain organic acids.^460^ In the first step, a low molecular weight PE (*M*_n_ = 1,700 g/mol, *M*_w_ = 4,000 g/mol) was activated by its reaction (at 65 °C in CHCl_3_) with chlorosulfuric acid (ClSO_3_H), which installed sulfonate groups (−SO_3_^–^) adjacent to C=C bonds along the PE backbone (Figure 80a).^461^ Coordination of these sulfonate groups to Fe(III) resulted in the modified polymer PESO_3_-Fe, with approx. equimolar amounts of S and Fe. Oxidation of PESO_3_-Fe by H_2_O_2_ was complete in solution at room temperature in about 10 min. Commercial plastics, including LDPE carrier bags and HDPE bottles, were also converted to organic acids in about 90 % yield within 2 h. The products were mono- and dicarboxylic acids, as well as sulfonic acids, in 73 % overall yield. The most important carbon-based side-product was CO_2_ (27 mol% yield, based on mol carbon).

The proposed mechanism for oxidative cleavage of PESO_3_-Fe is shown in Figure 80b. First, C=C bonds are cleaved to give dicarboxylic acids of various chain lengths. These molecules undergo further cleavage at C–C bonds in the α-, β- or γ-positions relative to remaining sulfonate groups. When a C=C bond is located near the polymer chain end, acetic or formic acid is formed directly.

**Scheme 57:**
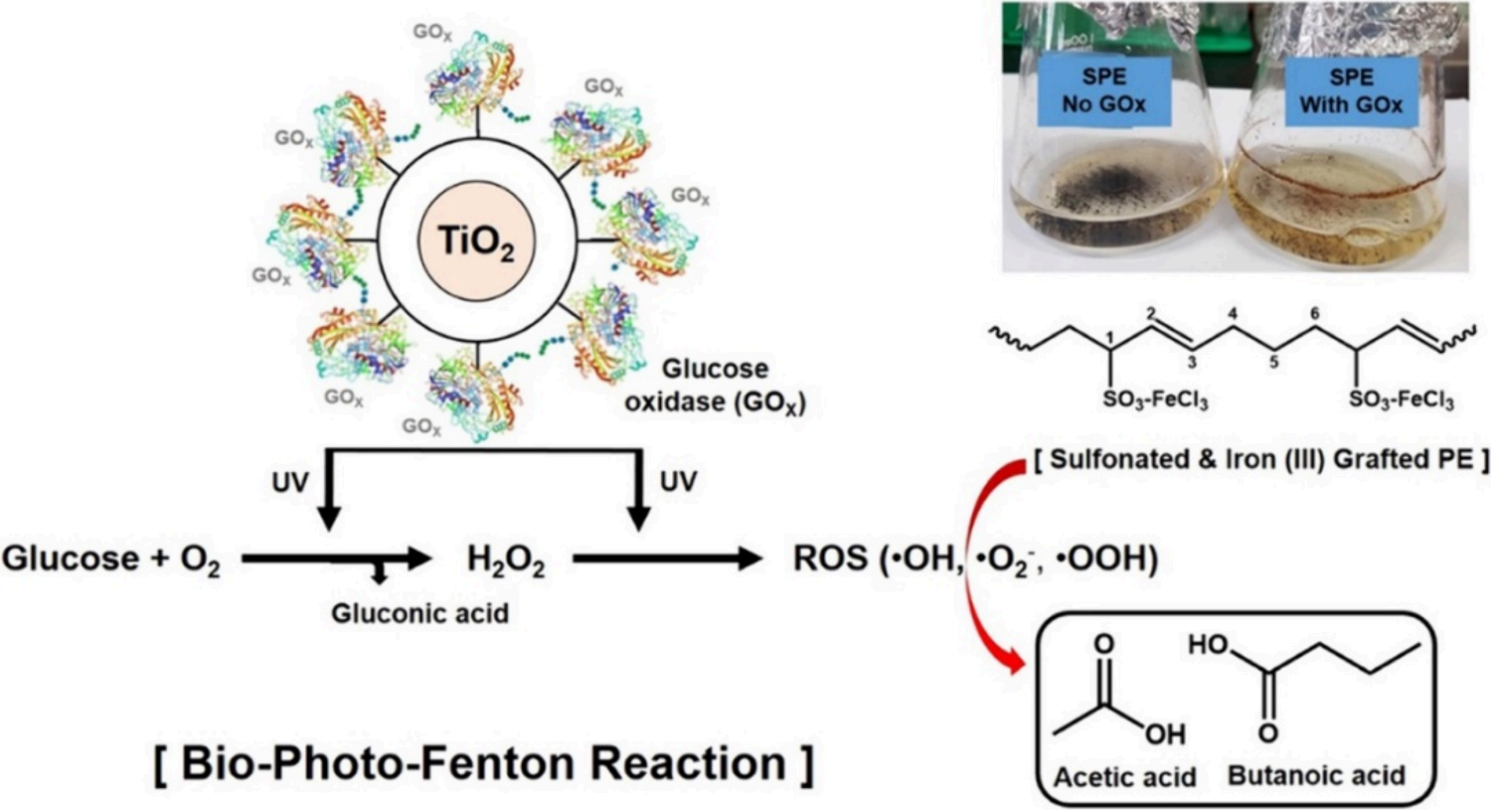
Conversion of a Sulfonated PE to Acetic and Butanoic Acids Reproduced with permission from ref (462). Copyright 2021, Elsevier.

A related strategy was used to convert a low molecular weight PE (*M*_n_ = 1700 g/mol, *M*_w_ = 4000 g/mol) to acetic acid by oxidizing PESO_3_-Fe with H_2_O_2_ generated *in situ* by the enzyme glucose oxidase (GOx) immobilized on TiO_2_ nanoparticles (TiO_2_-GOx), Scheme 57. The system was also irradiated with UV light, leading to its description as “bio-photo-Fenton”.^462^ The excited state TiO_2_ converted H_2_O_2_ to various reactive oxygen species (ROS), including OH radicals, which oxidized PESO_3_-Fe. This process was described as highly selective (>95%) towards acetic acid (although it was produced in only about 6% yield, based on carbon mass) and a small amount of butanoic acid. Nevertheless, the main product of the reaction was still CO_2_.

In another study, a low molecular weight PE (*M*_n_ = 2,800 g/mol, *M*_w_ = 5,900 g/mol) was converted via a Fenton process to a low molecular weight oxidized PE bearing alcohol, carboxylic acid, and methyl ester functional groups.^463^ A mixture of PE and Fe_2_O_3_ was ball-milled with an aqueous solution of H_2_O_2_ (30 wt%) for 90 min. About 1 g products was obtained from 1 g PE, suggesting that only trace CO_2_ formed. The mechanical forces associated with ball milling were believed to improve the contact between Fe_2_O_3_ and H_2_O_2_, favoring the generation of hydroxyl radicals.^464^ The product molecular weight depended on the reaction conditions. Adding more Fe_2_O_3_ and extending the milling time enhanced chain cleavage, generating products with lower molecular weights. Using 1 g H_2_O_2_ and 100 mg Fe_2_O_3_, the average molecular weight decreased to *M*_n_ = 300 g/mol, *M*_w_ = 2,600 g/mol. Scanning electron microscope (SEM) images and dynamic light scattering (DLS) analysis showed that the particle size of the residual PE decreased during the reaction.

### 5.5 C–H Bond Functionalization of Polyolefins

**Scheme 58:**
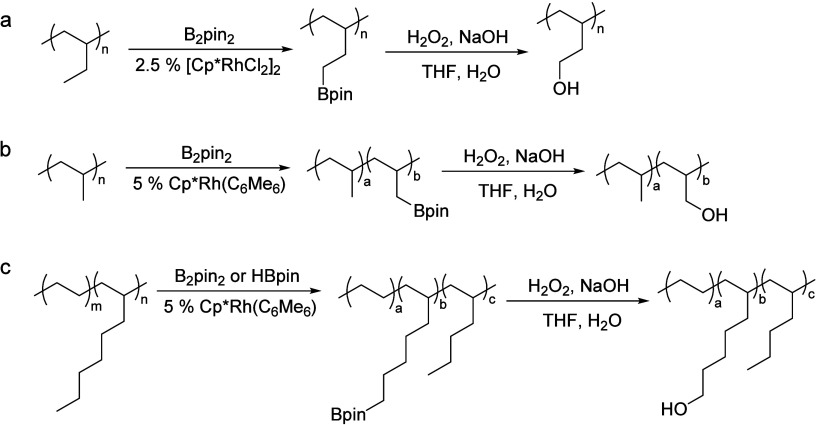
C–H Borylation and Subsequent Oxidation of Various Branched Polyolefins^465−467^

Inspired by alkane functionalization strategies, the C–H functionalization of poly(ethyl-ethylene) (PEE) was attempted using a molecular Rh catalyst.^465^ Borylation at the methyl groups of the ethyl side-chains required 36 h at 150 °C, then the borylated polymer was oxidized at room temperature using H_2_O_2_ to give PEE functionalized with hydroxyl groups (Scheme 58a). ^1^H NMR, ^13^C NMR, and IR confirmed the regioselective functionalization of CH_3_ groups, about 10 % of which were hydroxylated. Oxidation caused the molecular weight of PEE to increase slightly. For example, low molecular weight PEE-1 (*M*_n_ = 1,200 g/mol, *Đ* = 1.11) was converted to oxidized PEE-1 (*M*_n_ = 1,340 g/mol, *Đ* = 1.05), while high molecular weight PEE-2 (*M*_n_ = 37,000 g/mol, *Đ* = 1.06) was converted to oxidized PEE-2 (*M*_n_ = 38,900 g/mol, *Đ* = 1.28). Oxidation also resulted in an increase in the glass transition temperature (*T*_g_), from −42 to +11 °C, due to intermolecular interactions between OH groups on different PE chains.

The same strategy was applied to the oxidation of various PPs (aPP, *M*_n_ = 40,000 g/mol, *Đ* = 1.2 or *M*_n_ = 16,100 g/mol, *Đ* = 2.3; iPP, *M*_n_ = 17,600 g/mol, *Đ* = 2.1 or *M*_n_ = 66,800 g/mol, *Đ* = 3.8; sPP, *M*_n_ = 40,300 g/mol, *Đ* = 2.4 or *M*_n_ = 66,200 g/mol, *Đ* = 3.3), Scheme 58b,^466^ as well as an LLDPE (*M*_n_ = 112,000 g/mol, *Đ* = 1.9), Scheme 58c.^467^ A higher reaction temperature (200 °C) was required to borylate PP compared to the other two POs, presumably because the CH_3_ groups of PP close to the polymer backbone are more sterically hindered, decreasing the efficiency of C–H activation.

Other oxidants can be used to introduce oxygen into POs. These reactions are catalyzed by a variety of transition metal catalysts, giving polymers with various functional groups,^468−472^Table 12. The partially oxidized POs have mechanical properties that differ from the original POs. Incorporation of ca. 3 hydroxyl and carbonyl groups per 100 backbone carbons on PE chain increased adhesion between the polymer and aluminum by ca. 20×.^472^

**Table 12:** Catalytic Oxidative Functionalization of Various Polyolefins^468−472^

polymer	initial *M*_n_ [*Đ*]	oxidant^a^	catalyst^b^	final *M*_n_ (*Đ)*	functional groups^c^
PEP^d^	8,900 [1.1]	ozone	Mn(TDCPP)OAc	8,800 (1.1)	OH (0.3 – 1.1) CO (trace)
poly(1-butene)	151,000 [1.9]	ethyl diazoacetate	Tp^Br3^Cu(NCMe)	191,000 (1.8)	CO_2_Et (1.0)
PE	10,100 [8.7]	^m^CPBA	[Ni(Me_4_Phen)_3_]^+^	10,200 (8.3)	OH, Cl, CO, ester (total 2.0)
poly(isobutene)	18,100 [2.3]	^m^CPBA	Ru(TPFPP)(CO)	17,700 (2.1)	CO (0.6 - 2.3) OH, Cl (trace)
PE	25,900 [6.8]	2,6-dichloro-pyridine-*N*-oxide	Ru(TPFPP)(CO)	25,700 (6.9)	OH, CO (total 3.3)

a ^m^CPBA is meta-chloroperoxybenzoic acid.

b TDCPP is *meso*-tetra-2,6-dichlorophenylporphyrin; Tp^Br3^ is hydro-*tris*(3,4,5-tribromo-pyrazolyl)borate; phen is 1,10-phenanthroline; TPFPP is *tetrakis*-(pentafluorophenyl)porphyrin.

c Per 100 backbone carbons.

d PEP is poly(ethylene-*co*-propylene).

**Scheme 59:**
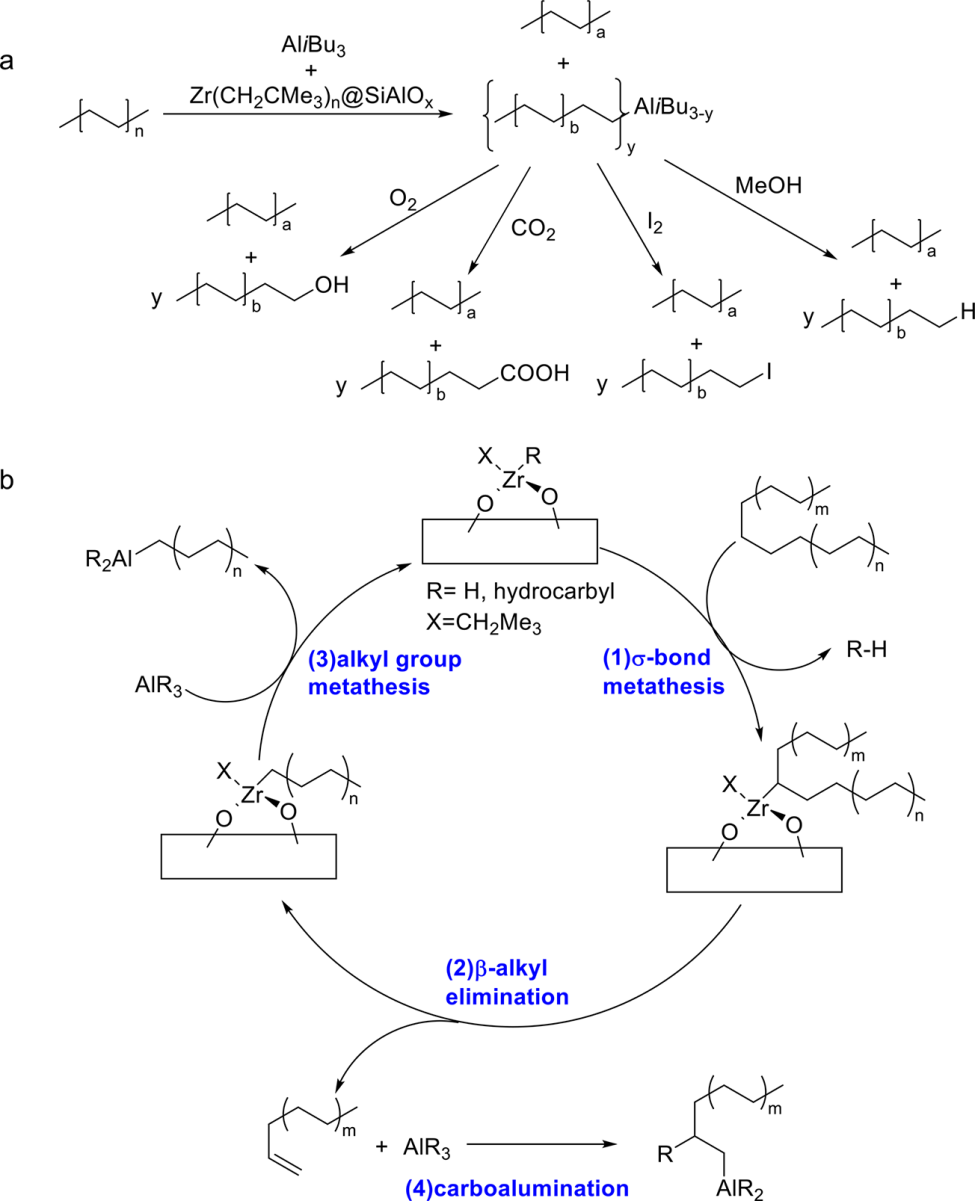
(a) Catalytic Conversion of PE to Functionalized Alkane Fragments, via Alkylaluminum Intermediates, and (b) Proposed Mechanism for PE Alumination, Catalyzed by a Supported Organozirconium Adapted with permission from ref (473). Copyright 2021, Elsevier.

Tetraneopentylzirconium (ZrNp_4_) supported on silica-alumina (Si/Al = 3:1) catalyzed the carboalumination of HDPE (*M*_w_ = 38,600 g/mol, *Đ* = 6.2) by Al^*i*^Bu_3_, Scheme 59a.^473^ The catalyst also promoted C–C bond cleavage via β-alkyl elimination, so that shorter hydrocarbon chains were produced (both alkenes and alkanes). The process required a stoichiometric amount of the expensive and air-sensitive alkylaluminum reagent. The aluminated products reacted readily with O_2_ in a subsequent step to form long-chain alcohols. The intermediates were also quenched with other reagents, for example, CO_2_, I_2_ and methanol, to give the corresponding carboxylic acids, iodoalkanes, and alkanes, respectively. Starting from 1 g HDPE, and adding fresh catalyst (0.11 g) and Al^*i*^Bu_3_ (120 μL) every 12 h, a total liquid yield of 84 % was eventually achieved after 36 h at 200 °C. The alkanes and mono-alcohols in the liquid products had carbon numbers from 20 to 50.

The proposed mechanism starts with polymer activation by σ-bond metathesis at a [ZrR] active site (Scheme 59b). β-Alkyl elimination produces an alkene, which undergoes carboalumination. Finally, alkyl group metathesis transfers the remaining polymer-derived ligand from Zr to Al. The process was also successfully applied to iPP (*M*_w_ = 117,600 g/mol, *Đ* = 3.2), giving similar results.

A similar PO alumination strategy was developed using Zr(O*t*Bu)_4_ supported on silica-alumina.^474^ Since this catalyst does not promote β-alkyl elimination, polymeryl ligands were transferred from Zr to Al directly. Therefore, PO alumination occurred with almost no change in carbon chain length. Starting from a low molecular weight PE (*M*_w_ =2,100 g/mol, *Đ* = 1.2), the product polymer had the same molecular weight and dispersity. Exposing the aluminated PE to air produced a PE functionalized with alcohol groups.

This process was also used to functionalize LDPE (*M*_w_ =8,100 g/mol, *Đ* = 4.7), LLDPE (*M*_w_ =15,500 g/mol, *Đ* = 4.7), iPP (*M*_w_ =57,400 g/mol, *Đ* = 6.8), and PS (*M*_w_ =40,600 g/mol, *Đ* = 2.0). After reaction, the molecular weight had increased by less than 10 % due to oxygen incorporation, indicating a low extent of oxidation (less than three hydroxyl groups per chain, on average).

### 5.6 Conclusions and Perspectives

Both thermal catalytic oxidation and photocatalytic oxidation are potential approaches for upcycling POs. Early research on thermal catalytic oxidation focused on identifying functional groups without considering the potential commercial value of the resulting oxygenated products. Subsequent studies have partly addressed this oversight. Several processes have been developed to convert POs to low molecular weight dicarboxylic acids at relatively low reaction temperatures (≤200 °C). Potential applications for these acids have been described, e.g., as monomers for polyesters, and as substrates for biological conversion. Other oxygenates are also desirable targets, but it remains a challenge to obtain partial oxidation products like aldehydes and ketones selectively.

Compared to thermal catalytic oxidation, photocatalytic oxidation is conducted at even milder reaction temperatures, most commonly at room temperature. However, the rates are usually very low, involving reactions of very small amounts of polymer and often requiring several days to go to completion. The low solubility of POs under these conditions is surely a limiting factor. The quantum efficiency and energy cost of providing the UV light are an important, but often overlooked, consideration, making the operating temperature a misleading proxy for the energy requirement of the reaction. Low yields and over-oxidation present other significant challenges. Many reports focus on PS photooxidation for this reason, since the stability of benzoic acid makes it resist further oxidation to CO_2_. At this time, few papers have explored photocatalytic oxidative upcycling of other types of POs, such as PE and PP, although they represent a much larger fraction of plastic waste. The yields of formic or acetic acids from these polymers are typically less than 20 mol%, with CO_2_ being the major product (undermining the goals of polymer upcycling and carbon circularity). Suppressing over-oxidation in larger scale reactions with higher quantum efficiencies would be a major breakthrough for photocatalytic PO upcycling.

C–H functionalization of POs to create new types of higher value polymers is another strategy that is starting to attract attention, although the primary focus of many of these studies has not generally been polymer upcycling. Achieving functionalization without reducing molecular weight is usually the goal, although it can be difficult to suppress chain cleavage under oxidizing conditions. A combination of C–H functionalization with C–C bond cleavage could represent new opportunities in PO upcycling, by creating routes to small and intermediate molecular weight hydrocarbons whose functional groups impart higher value.

## 6 General Conclusions, Challenges, and Outlook

### 6.1 Thermodynamic Considerations in Polyolefin Upcycling

#### 6.1.1 Enthalpy Cost

**Figure 81:**
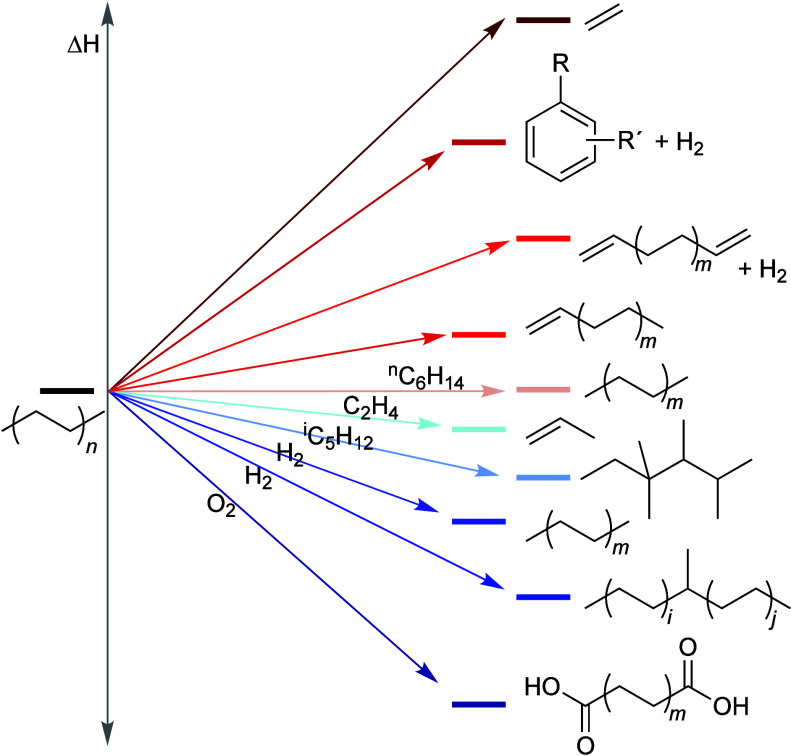
Targets of PE upcycling vary in their enthalpy cost. Coreagent-free strategies (complete depolymerization to ethylene, and partial depolymerization to other alkenes/alkadienes, as well as aromatization) are endothermic, while many strategies that involve a coreagent are exothermic.

Enthalpy differences between a PO such as PE and the various types of depolymerization products derived from it vary widely. Figure 81 provides a qualitative summary. Since values for the thermodynamic properties of many PO upcycling products (especially those with large carbon numbers) are not available, computational assessments are needed. Still, the sheer number of individual compounds makes conventional approaches prohibitively expensive in many cases, so general contribution models and/or individual model compounds have been used to obtain approximate results.^475^ (A similar concern applies to conventional microkinetic models, which would have to incorporate thousands of steps. They can be handled by lumping similar reactions.)^254,476^ Data science tools and machine learning may be used to good effect here, and new approaches to model these macromolecular reactions realistically may be powerful.

Based on this qualitative thermodynamic assessment, the upcycling strategies described in this review can be classified into three general categories. The first is coreagent-free. It includes processes like catalytic cracking and hydrogen redistribution to a mixture of alkanes and aromatics (represented by the red arrows in Figure 81). These approaches simply reorganize the carbon and hydrogen atoms of the PO without the need for additional inputs.

The second general approach involves the use of a coreagent such as H_2_, alkanes, alkenes, O_2_, or H_2_O_2_ (represented by the blue arrows in Figure 81). These processes tend to be much more favorable thermodynamically. However, the energy required to make the coreagent (e.g., H_2_, ethylene, alkanes) may be significant, and cannot be neglected in the overall thermodynamic assessment. The third approach is depolymerization by alkane metathesis, which requires a light alkane as a coreagent, but is nearly thermoneutral. General thermodynamic features of these strategies are described below.

#### 6.1.2 Coreagent-Free Depolymerization Strategies

**Figure 82:**
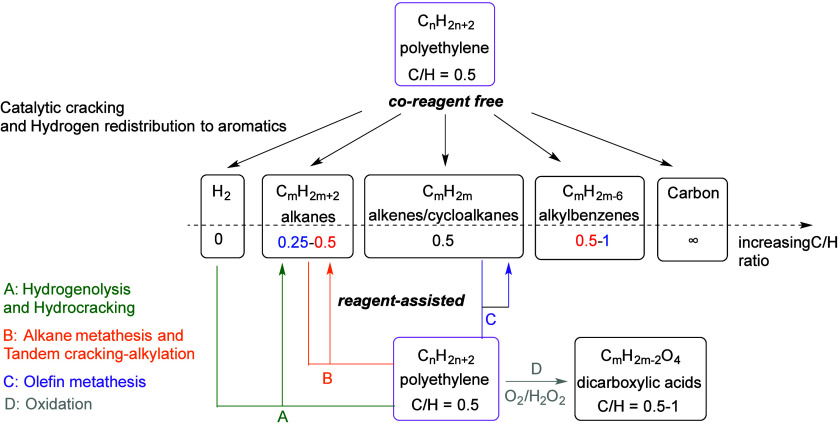
Catalytic strategies for PE upcycling, organized by C/H ratio: coreagent-free (upper grey arrows) and reagent-assisted (lower colored arrows; colored lines indicate coreagents). C/H ratios are shown for PE and for each product category. For products where the ratio varies depending on the carbon number C_*m*_, the limit for the smallest possible carbon number is shown in blue; the limit as C_*m*_ approaches infinity is shown in red.

Obviously no added reagents are *required* for a perfectly atom-efficient depolymerization to olefins (low pressure H_2_ may be present in the reactor, not as a stoichiometric reactant, but for the purpose of extending catalyst lifetime). The C/H ratio of 0.5 in PE is the same as its monomer (ethylene) and other olefins, Figure 82. However, typical bond dissociation energies for C–C and C=C bonds are 347 and 614 kJ/mol, respectively, and since depolymerization converts two C–C bonds to one C=C bond, it is strongly enthalpically disfavored. However, breaking a PE chain into smaller fragments supplies an entropic driving force. Therefore, depolymerization to smaller olefins can be achieved at higher temperatures, although selectivity suffers as side-reactions become important.

PE depolymerization to longer-chain alkenes (ethylene oligomers) is less energy intensive than depolymerization to ethylene because fewer C–C bonds are broken. Furthermore, the product alkenes have higher intrinsic value than the original monomers. New methods for selective partial depolymerizations are desirable. Hydrogen redistribution reactions can also target other hydrocarbon product mixtures, including alkadienes and aromatics. When alkylbenzenes are a major product (C/H = 0.5–1.0), alkanes (C/H = 0.25–0.5) and/or H_2_ are formed as co-products, incurring separation costs to recover the aromatic components. The addition of CO_2_ to consume H_2_ by RWGS can help to make aromatization thermodynamically more favorable, improving the yield of alkylaromatics.^330,331^

All coreagent-free processes are challenged to target specific molecular weights, because chain cleavage is mostly random along an undifferentiated PO backbone. Consequently, there is no intrinsic kinetic or thermodynamic selectivity in terms of molecular weight. Some applications (e.g., fuels, lubricants, solvents, surfactants) tolerate a range of molecular weights, but uses of depolymerization products as chemical building blocks generally depend on selectivity. The energy requirement (and accompanying cost) of the separations must be considered in assessing the overall value of an upcycling process. A broader array of much more effective strategies (for some rare examples, see sections 2.4.3, 3.3.4, and 3.6.3.1) to control product distributions and target specific molecular weights is needed.

#### 6.1.3 Coreagent-Assisted Depolymerization Strategies

Coreagents can increase the thermodynamic driving force for depolymerization, relative to coreagent-free strategies. Hydrogenolysis produces *n*-alkanes; hydrocracking and tandem cracking-alkylation yield mostly *iso*-alkanes; tandem isomerization/ethenolysis produces propylene; and partial oxidation results in dicarboxylic acids as well as other oxygenates. Hydrogenolysis, hydrocracking, alkylation and oxidation are all exothermic, while alkane metathesis is nearly thermoneutral. Thus, depolymerization can proceed with considerably less energy input (although the energy needed to produce the coreagent(s) may be considerable, and must be included in the overall energy footprint). As expected, activation barriers are generally smaller for these thermodynamically favorable reactions, allowing them to proceed at lower temperatures and with less coke formation than the coreagent-free processes. Reactions that incorporate even more types of small molecules into the polymer fragments and generate new types of products await discovery.

The economic viability of all of these processes will depend on the price and availability of the coreagent at scale (which is very large for waste POs). Furthermore, although coreagent-assisted strategies may be highly selective in terms of a single *type* of product (e.g., *n*-alkanes), they usually generate a distribution of molecular weights within that product type category, making products suitable for applications that do not require a specific molecular weight, or else incurring similar separation costs as the coreagent-free strategies. In addition, the separation (and presumably recycle) of the coreagent must be considered. For example, the cross-metathesis of PE with *n*-hexane requires a large excess of the light alkane (indeed, in a seminal example, the majority of the mass of liquid products was derived from the coreagent).^343^

### 6.2 Opportunities in Catalyst and Reactor Design

The investigation of PO depolymerization mechanisms, and the use of this information to inform catalyst and reactor design, can be facilitated by studies of small-molecule hydrocarbon model compounds. Many well-known mechanisms (e.g., hydrogenolysis, cracking, metathesis) appear to translate well from the molecular to the macromolecular regime. For example, the metal/acid balance (MAB) influences the rate and product selectivity in PO hydrocracking in ways that are analogous to behaviors in small molecule reactions.^195,219^ However, there are significant differences between small molecule and macromolecular chemistries that are often under-appreciated by the catalysis community, and caution is warranted when extrapolating insights. Some examples are described below.

#### 6.2.1 Mitigating Mass Transfer Limitations

PO melts have much higher viscosities, and individual polymer chains have much lower mobilities, relative to smaller molecules. Consequently, efficient mixing can be hard to achieve in depolymerizations, and mass transfer effects must be evaluated. For heterogeneous catalysts with active sites located within their micro/mesopore structures, the ability of longer chains to access sites that are not on or near the external surfaces on the timescale of the reaction is not guaranteed. The slow diffusion of macromolecules between different types of active sites in a tandem reaction may also be problematic, except when the overall depolymerization is intrinsically slow (as it often was in early reports). Large pore and/or hierarchical catalyst structures may be more effective as catalysts in controlling the proximity of active sites and the access of reaction intermediates to them. Similar to processes involving solid biomass, pretreatment steps to reduce PO molecular weight prior to selective catalytic depolymerization may be useful.

#### 6.2.2 Mitigating Heat Transfer Limitations

Another consequence of the high melt viscosity and low thermal conductivity of POs is that heat may be unevenly distributed in the reactor. This feature has long made the design of alkene polymerization processes very challenging. Similarly, PO depolymerization outcomes may show a strong (and often unacknowledged) dependence on reactor size and configuration, as well as the amount of polymer in the reactor. Wherever possible, experiments should be conducted multiple times *and at more than one scale/in more than one reactor*, to explore reproducibility and validate comparisons.^56,57^ Catalysts responsive to microwave heating may be helpful in providing heating at or near the active sites.^310,477^ New catalytic reactor designs, including melt extruders designed for polymer processing,^13^ may be needed to better manage heat and mass transport issues for macromolecular transformations.

#### 6.2.3 Solvent Effects

The use of a solvent can dramatically improve mass as well as heat transfer. However, many POs are highly insoluble, and therefore require the use of very large amounts of solvent. Furthermore, the low reactivity of POs can make it difficult to choose a solvent that will not participate in the reaction. Solvent choice can also have a profound effect on the outcome of the reaction via its influence on polymer conformation.^125^ These effects remain largely unexplored, but they represent opportunities for future investigation.

During PO depolymerization, the formation of liquid-range hydrocarbons supplies the reaction with a PO-derived solvent. A rarely-acknowledged consequence is that mass and heat transport limitations are likely to vary considerably over the course of a reaction, complicating efforts to measure and compare intrinsic catalytic PO upcycling performance.

### 6.3 Considerations for Economically Viable and Environmentally Responsible Upcycling

#### 6.3.1 Purification of Waste Polyolefin Feedstocks

In commercial uses, a PO is not a single material but a group of materials. Even POs with the same nominal composition (e.g., (CH_2_)_*n*_ for polyethylene) and product category (e.g., LDPE) are manufactured in a large number of grades and formulations, each with a characteristic molecular weight and molecular weight distribution, type and frequency of branching, and where relevant, stereochemistry and stereoregularity. Each of these characteristics can impact the efficiency of a depolymerization process and, potentially, the product distribution. For example, PE branching appears to enhance the rates of C–C bond scission via hydrocracking and oxidation, but impedes the rates of metathesis and hydrogenolysis.

Post-consumer waste plastic contains not only multiple PO types, but even when separated, multiple grades within each type. Upcycling processes will eventually need to deal with this inherent feedstock variability. Industry-standard density separation generates PO streams contaminated by polymers containing various heteroatoms (e.g., Cl, O, N), at variable levels. For example, multilayer materials contain both POs and heteroatom-containing polymers. Furthermore, most commercial plastics contain significant amounts of organic additives (currently manufactured at approx. 10 wt% of total plastic production) as well as inorganic fillers, which are incorporated to modify polymer properties such as flexibility, flammability, and durability.^289^ Functional groups found in these additives include ethers, phthalates, phosphates, amines, benzophenones, etc., for which depolymerization catalysts have different levels of tolerance. For example, chlorine tends to bind strongly to metal sites, while amines poison acid sites. Transition metal hydrides used in hydrogenolysis and alkene metathesis catalysts can be extremely sensitive to poisoning by polar compounds, including common feed impurities such as H_2_O and O_2_.

The energy requirements (closely related to the cost) for plastic separation and/or purification (e.g., by selective dissolution of polymers),^478^ including the stripping of additives,^290^ will need to be taken into account in designing economically viable catalytic depolymerization processes. Alternatively, information about the impact of additives on catalytic upcycling could be used by resin manufacturers to design future plastics and the products made from them with a view to facilitating their depolymerization.

#### 6.3.2 Product Separation

Occasionally, POs can be converted to a single molecular product with high selectivity. For example, PE can be converted almost exclusively to propylene via its reaction with excess ethylene (although separation of ethylene and propylene in the product stream is still required), described in section 4.3.4.^344,396^ Several papers report that PS can be oxidized selectively to benzoic acid (see section 5.3.1). In contrast, most depolymerization approaches generate hydrocarbon mixtures that are difficult and costly to separate into individual molecular products. For example, catalytic cracking forms complex mixtures of alkanes, alkenes, cycloalkanes, and aromatics.^191^ While catalytic hydrocracking generates a simpler product mixture containing “only” *iso*-alkanes, the number of possible isomers is typically very large. Catalytic hydrogenolysis to *n*-alkanes is more selective in this respect, since the molecular weight distribution is the only source of product complexity.

More selective processes with lower product separation needs can be achieved with more complex catalyst and/or reactor designs. However, the cost of a more involved catalytic process must be compared to the cost of separation. Some catalyst architectures alter product distributions by affecting the retention times of intermediates. For example, changing the dimensions of a zeolite crystal alters the time spent by alkene cracking products in the micropores. This parameter was tuned to promote selectivity in the catalytic cracking of PE or PP to either light alkenes (section 3.3.4) or BTX aromatics (section 3.6.3.1).^42,209^ In a similar strategy, coating silica-supported Pt nanoparticles with a mesoporous silica shell led to a narrowing of the alkane molecular weight distribution in PE hydrogenolysis, compared to the same catalyst without the shell (section 2.4.3).^146^ Finally, product mixtures may be simplified by biological funneling via a specific metabolic pathway towards a single compound or material (section 5.2.3).^434^

#### 6.3.3 Catalyst Separation and Recycle

In many reports of batch catalytic PO depolymerizations, high catalyst loadings (total mass, including and usually dominated by the catalyst support) are needed to achieve high conversion to low molecular weight products. Catalyst recovery and reuse are sometimes, but not always, investigated, and the total amount of polymer converted even in multiple batch cycles is almost always far lower than needed for a commercial process. Simple catalysts (e.g., supported metal nanoparticles) can be recovered with the insoluble organics and may be regenerated by simple calcination, followed by reduction. (We note that polymers with inorganic fillers pose a different problem for catalyst recovery, since inorganic deposits are usually not removed by calcination, and require pretreatment filtration instead.) More complex catalyst architectures may provide higher activity and selectivity in their pristine state, but may not be robust enough to survive the mechanical and chemical degradation expected during long-term use and during regeneration. Some types of catalysts (e.g., first-row transition metals, supported or unsupported organometallic compounds) may be more susceptible to poisoning (see section 3.3.5) and are inherently more difficult to recover/recycle.

Some tandem catalytic approaches described in this review are elegant and efficient strategies, but when two distinct catalysts are used for different steps in a one-pot process, they present unique challenges for catalyst regeneration. If the catalysts are intimately mixed in the reactor, they may be impossible to separate and regenerate. Single-component, multi-site, or multi-functional catalysts for tandem processes are a possible solution.

#### 6.3.4 Roles for Technoeconomic Analysis and Life Cycle Assessment

The complexity of catalytic PO upcycling presents enormous challenges to the development of new catalysts and new processes. In fundamental research, there is generally no “too expensive” disqualification. However, resolving environmental end-of-life issues with plastic waste urgently requires decisions about which strategies to pursue, based on includes factors other than chemical feasibility. Preliminary techno-economic analysis (TEA) and life-cycle assessment (LCA) can be used to evaluate the economic and environmental impacts, respectively, of potential PO upcycling processes.^479^ All steps, including the collection, separation, and purification of the plastic waste, catalyst synthesis, recovery, and regeneration, solvent recovery and recycling, product separation, and disposal of solid residues, need to be considered.^480^

It is important to acknowledge the large and unavoidable uncertainties associated with such preliminary analyses, in the absence of a complete process design and scaleup information. Using preliminary TEA and LCA results as marketing tools to promote bench-scale upcycling results to journal readers is not helpful. Instead, these techniques should be viewed as tools to evaluate the differential impacts of different process components and parameters, thereby guiding and prioritizing targets for process improvement.^291,292^ A lack of standardization in LCA methodologies, definitions, and choices of system boundaries, still hampers comparisons.^481^ Analyses should also acknowledge that while targeting higher value products may make a techno-economic analysis look more appealing, the potential market is much smaller for these products. LCA may also make a process targeting lower volume upcycling products look attractive, but the contributions of such processes to mitigating plastic waste are necessarily minimally impactful. There are also strict regulatory constraints on certain types of targets (e.g., those intended for use in the food or health care sectors), since trace impurities from the waste plastic can be carried along in the upcycling process.

### 6.4 Outlook for Polyolefin Upcycling

Today’s POs still retain a major cost advantage over bio-based and biodegradable alternatives, and a meta-analysis of LCAs suggested that they may have smaller overall environmental footprints (e.g., GHG emissions, water use) in their production as well.^481−483^ While eliminating some non-essential uses of POs (e.g., superfluous packaging) is an essential part of an overall plastic waste reduction strategy, their intrinsic advantages are likely to remain relevant for some time. Consequently, it is essential to develop better strategies to deal responsibly with the end-of-life phase for POs.

Our ability to use POs as future chemical feedstocks is likely to depend on the availability of low cost, renewable coreagents, such as H_2_, ethylene, and light alkanes. The processes are likely to be thermal, since photochemical efficiencies are extremely low and electrochemical approaches are hampered (possibly irredeemably) by extremely low PO solubilities in electrolyte-containing solvents near room temperature. While making high value products can result in an attractive TEA, any products that are valuable enough to justify this expense can probably be made more efficiently from feedstocks that are much more tractable than waste plastics. Likewise, converting waste POs to functionalized polymers will have to compete with on-purpose manufacturing of such polymers. The significant variability and contamination that comes with using a waste material as feedstock is likely to make adoption of such recycled materials challenging. The highly engineered nature and performance specifications for polymer materials seems likely to make on-purpose polymer synthesis from monomers more attractive for the foreseeable future.

The current technology for recovering value from waste POs is thermal cracking to alkenes and other basic chemical feedstocks.^484^ It remains to be seen whether our chemical ingenuity can overcome the barriers to creating more energy efficient and economically attractive uses for our waste plastics. If this proves possible, we can confidently predict that catalysis will be an important part of the solution.
